# Oral regimens for rifampin-resistant, fluoroquinolone-susceptible tuberculosis

**DOI:** 10.1056/NEJMoa2400327

**Published:** 2025-01-30

**Authors:** Lorenzo Guglielmetti, Uzma Khan, Gustavo E. Velásquez, Maelenn Gouillou, Amanzhan Abubakirov, Elisabeth Baudin, Elmira Berikova, Catherine Berry, Maryline Bonnet, Matteo Cellamare, Vijay Chavan, Vivian Cox, Zhanna Dakenova, Bouke Catherine de Jong, Gabriella Ferlazzo, Aydarkhan Karabayev, Ohanna Kirakosyan, Nana Kiria, Mikanda Kunda, Nathalie Lachenal, Leonid Lecca, Helen McIlleron, Ilaria Motta, Sergio Mucching Toscano, Hebah Mushtaque, Payam Nahid, Lawrence Oyewusi, Samiran Panda, Sandip Patil, Patrick P.J. Phillips, Jimena Ruiz, Naseem Salahuddin, Epifanio Sanchez Garavito, Kwonjune J. Seung, Eduardo Ticona, Lorenzo Trippa, Dante E. Vargas Vasquez, Sean Wasserman, Michael L. Rich, Francis Varaine, Carole D. Mitnick

**Affiliations:** 1 Médecins Sans Frontières, Paris (L.G., F.V.); 2 Sorbonne Université, National Institute of Health and Medical Research (INSERM), U1135, Centre d’Immunologie Et Des Maladies Infectieuses, Paris (L.G.); 3 Assistance Publique Hôpitaux de Paris (APHP), Groupe Hospitalier Universitaire Sorbonne Université, Hôpital Pitié Salpêtrière, Centre National De Référence Des Mycobactéries Et De La Résistance Des Mycobactéries Aux Antituberculeux, Paris (L.G.); 4 Interactive Development and Research, Singapore, Singapore (U.K.); 5 McGill University, Epidemiology, Biostatistics and Occupational Health, Montreal (U.K.); 6 UCSF Center for Tuberculosis, University of California San Francisco, San Francisco (G.E.V., P.N., P.P.J.P); 7 Division of HIV, Infectious Diseases, and Global Medicine, University of California San Francisco, San Francisco (G.E.V.); 8 Epicentre, Paris (M.G., E.B.); 9 National Scientific Center of Phthisiopulmonology, Almaty (A.A., E.Be.); 10 Médecins Sans Frontières, London (C.B., I.M.); 11 Translational Research on HIV and Endemic and Emerging Infectious Diseases, Montpellier Université de Montpellier, Montpellier, Institut de recherche pour le développement, Montpellier, National Institute of Health and Medical Research (INSERM), Montpellier (M.B.); 12 MedStar Health Research Institute, Washington D.C. (M.C.); 13 Médecins Sans Frontières, Mumbai (V.C.); 14 Centre for Infectious Disease Epidemiology and Research, University of Cape Town, Cape Town (V.Co.); 15 City Center of Phthisiopulmonology, Astana (Z.D.); 16 Institute of Tropical Medicine, Antwerp (B. C. dJ); 17 Médecins Sans Frontières, Geneva (G.F., N.L.); 18 Center of Phthisiopulmonology of Almaty Health Department, Almaty (A.K.); 19 Médecins Sans Frontières, Yerevan (O.K.); 20 National Center for Tuberculosis and Lung Diseases, Tbilisi (N.K.); 21 Partners In Health, Maseru (M.K.); 22 Socios En Salud Sucursal Peru, Lima (L.L., S.M-T., J.R., E.S-G., D.E.V-V.); 23 Global Health and Social Medicine, Harvard Medical School (L.L., K.J.S., M.L.R., C.D.M.); 24 Department of Medicine, University of Cape Town, Cape Town (H.Mc.); 25 Medical Research Council Clinical Trials Unit at University College London, London (I.M.); 26 The Indus Hospital and Health Network, Karachi (H.M., N.S.); 27 Jhpiego Lesotho, Maseru (L.O.); 28 Indian Council of Medical Research Headquarters - New Delhi, New Delhi (S.P.); 29 Indian Council of Medical Research-National AIDS Research Institute, Pune (S.Pat.); 30 Hospital Nacional Sergio E. Bernales, Centro de Investigacion en Enfermedades Neumologicas, Lima (E.S-G.); 31 Partners In Health, Boston (K.J.S., M.L.R., C.D.M.); 32 Division of Global Health Equity, Brigham and Women’s Hospital, Boston (K.J.S., M.L.R., C.D.M.); 33 Hospital Nacional Dos de Mayo, Lima (E.R.T); 34 Universidad Nacional Mayor de San Marcos, Lima (E.R.T.); 35 Dana-Farber Cancer Institute, Department of Biostatistics and Computational Biology, Boston (L.T.); 36 Harvard University T.H. Chan School of Public Health, Boston (L.T.); 37 Hospital Nacional Hipólito Unanue, Lima (D.E.V-V.); 38 St George’s University of London Institute for Infection and Immunity, London (S.W.); 39 Wellcome Centre for Infectious Diseases Research in Africa, Institute of Infectious Disease and Molecular Medicine, Cape Town (S.W.)

## Abstract

For decades, poor treatment options and low-quality evidence plagued care for multidrug/rifampin-resistant tuberculosis (MDR/RR-TB). The advent of new anti-TB drugs and enhanced funding now permit randomized controlled trials of shortened, all-oral treatment for MDR/RR-TB.

**Methods:**

endTB is an international, open-label, Phase 3 non-inferiority, randomized, controlled clinical trial to compare five 9-month all-oral regimens including bedaquiline (B), delamanid (D), linezolid (L), levofloxacin (Lfx) or moxifloxacin (M), clofazimine (C) and pyrazinamide (Z), to the standard (control) for treatment of fluoroquinolone-susceptible RR-TB. Participants were randomized to 9BLMZ, 9BCLLfxZ, 9BDLLfxZ, 9DCLLfxZ, 9DCMZ and control using Bayesian response-adaptive randomization. The primary outcome was favorable outcome at week 73 defined by two negative sputum culture results or by favorable bacteriologic, clinical, and radiologic evolution. The non-inferiority margin was 12 percentage points.

**Results:**

Of 754 randomized patients, 699 and 562 were included in the modified intention to treat (mITT) and per-protocol (PP) analyses, respectively. In mITT, the control had 80.7% favorable outcomes and 9BCLLfxZ (Risk Difference [RD]: 9.8% [95%CI: 0.9, 18.7]), 9BLMZ (RD: 8.3% [95%CI: -0.8, 17.4]), 9BDLLfxZ (RD: 4.6% [95%CI: -4.9, 14.1]), and 9DCMZ (RD: 2.5% [95%CI: -7.5, 12.5]) were non-inferior. Relative treatment efficacy was similar in the PP population with the exception of 9DCMZ. The proportion of participants experiencing grade 3 or higher adverse events was similar across the regimens. Grade 3 or higher hepatotoxicity occurred in 11.7% overall and in 7.1% of the control.

**Conclusions:**

Consistent results across all analyses support the non-inferior efficacy of these three all oral shortened regimens. The endTB trial increases treatment options for MDR/RR-TB.

(Funded by Unitaid and others; ClinicalTrials.gov: NCT02754765)

Tuberculosis resistant to rifampin (RR-TB), a key anti-tuberculosis drug, is a major global health threat. According to the World Health Organization (WHO), 410,000 people become sick with RR-TB annually. Only 40% are diagnosed and treated, 65% of them successfully.^1^ Historically, poor response was largely due to the suboptimal 18-to 24-month regimens, which included injected aminoglycosides/polypeptides and caused substantial toxicity.^2^ Regimens were devised based on expert opinion and pooled analyses of observational studies because no evidence was available from contemporary randomized, controlled clinical trials.^3,4^ In 2016-2017, hope of improved evidence and treatment emerged with the launch of endTB and two other multi-country, randomized, controlled trials to examine whether shorter, all-oral regimens of 6- or 9-months duration could safely and efficaciously treat multidrug-resistant (MDR)/RR-TB in adults and adolescents. The STREAM 2 study examined a 9-month, 7-drug bedaquiline-containing regimen.^5^ TB-PRACTECAL studied three 6-month regimens comprising a nucleus of bedaquiline, linezolid, and pretomanid.^6^ The endTB (Evaluating Newly Approved Drugs for Multidrug-resistant Tuberculosis) trial (ClinicalTrials.gov identifier NCT02754765), reported here, evaluated the efficacy and safety of five 9-month, all-oral treatment regimens compared to the evolving standard of care for fluoroquinolone-susceptible RR-TB. This Phase III clinical trial used Bayesian response-adaptive randomization^7,8^ to improve the use of newer (bedaquiline and delamanid) and repurposed (clofazimine and linezolid) drugs and offer alternatives for patient-centered care.

## Methods

### Design and oversight

endTB is an international, multicenter, open-label Phase III, non-inferiority trial conducted by the endTB consortium (see Supplement). Full design (Figure S1) and implementation details are published^9^ and available at NEJM.org. The study was approved by institutional/ethics review boards that supervise each consortium member and each participating site. All participants provided written informed consent.

The distribution of study responsibilities across the team and additional oversight are described in the supplement (Section 2.2; Tables S1-S5). All authors vouch for the accuracy and completeness of the data and for the fidelity of the trial to the protocol (available at nejm.org). The Consolidated Standards of Reporting Trials extension for adaptive design trials guided this trial report.^10^

### Participants

Individuals aged 15 years or older who had fluoroquinolone-susceptible, pulmonary RR-TB confirmed by WHO-endorsed rapid tests were enrolled at 12 sites, which were run by endTB partners (Table S2), in Georgia, India, Kazakhstan, Lesotho, Pakistan, Peru, and South Africa, with the goal of ensuring representativeness (Table S6). Inclusion was irrespective of human immunodeficiency virus (HIV) serostatus and CD4 lymphocyte count. The trial excluded persons with baseline: pregnancy; elevated liver enzymes; uncorrectable electrolyte disorders; QT interval corrected by the Fridericia formula (QTcF) ≥450 msec; resistance or prior exposure (≥30 days) to bedaquiline, delamanid, clofazimine, or linezolid; and ≥15 days treatment with any second-line anti-tuberculosis drug during the current TB episode.^9^ The Supplement details baseline eligibility criteria and study retention of participants who became pregnant.

### Randomization and treatment

Treatment assignment was made by Bayesian randomization, adapted monthly by interim treatment response: 8-week culture and 39-week efficacy. Details have been previously published.^7,8^ Assignment occurred through a centralized interactive randomization system.

Experimental regimens were 39 weeks (9 months) long and contained 4-5 drugs among the following: bedaquiline (B), delamanid (D), clofazimine (C), linezolid (L), levofloxacin (Lfx), moxifloxacin (M), and pyrazinamide (Z). Regimen combinations were: 9BLMZ, 9BCLLfxZ, 9BDLLfxZ, 9DCLLfxZ, and 9DCMZ (Figure S2, Table S7). Control group regimens reflected WHO Guidelines in effect during the trial (Table S8).^4,11,12^ Treatment was administered 7 days/week, 6 under direct observation. In linezolid-containing experimental arms, linezolid dose was decreased at Week 16 or sooner if necessary to reduce toxicity. (Figure S2 and Table S7).

### Procedures

Clinical, safety, and mycobacteriologic assessments occurred weekly until week 12, every 4 weeks until week 47, and every 6-8 weeks thereafter (Table S9). Standardized mycobacteriology tests were performed in designated, quality-controlled, trial-site laboratories; the Institute of Tropical Medicine supported site laboratories and performed additional testing. Procedures included smear microscopy and culture in Mycobacteria Growth Indicator Tube (MGIT) system at all laboratories and on solid Löwenstein-Jensen media at all laboratories except in South Africa. Phenotypic drug susceptibility (DST) testing was performed in MGIT for at least rifampin and fluoroquinolones. DST for bedaquiline, clofazimine, delamanid, and linezolid were gradually introduced.

### Outcomes

Favorable outcome at week 73 was the primary efficacy endpoint. It was established by the absence of an unfavorable outcome and either 1) two consecutive, negative cultures (one between weeks 65 and 73); or 2) favorable bacteriological, radiological, and clinical evolution. Unfavorable outcomes were: death (from any cause); replacement/addition of one drug in the experimental arms or two drugs in the control arm; or initiation of new RR-TB treatment (Supplement 2.6.2).

Maximum follow-up was 104 weeks. Study follow-up ended when the final participant reached 73 weeks post-randomization. Favorable outcome at week 104 was a secondary endpoint (Supplement 2.6.3). Outcomes were adjudicated by the Clinical Advisory Committee (Supplement 2.6.4).

Safety outcomes were Grade 3 or higher adverse events (AEs), serious AEs (SAEs), death, discontinuation of at least one study drug due to AEs, and AEs of special interest (AESIs) defined as Grade 3 or higher: hepatotoxicity, hematologic toxicity, optic neuritis, peripheral neuropathy, or QTcF prolongation, all by week 73 (Supplement 2.6.5). AEs could be established by lab values alone and were graded by the site investigators according to the standardized MSF Pharmacovigilance Unit Severity Scale (available at NEJM.org).

### Analysis populations

Modified intention-to-treat (mITT) and per-protocol (PP) were co-primary analysis populations. The mITT population included all randomized participants who took at least one dose of study treatment (safety population) and who had a pre-randomization culture positive for *M. tuberculosis*. It excluded participants with baseline phenotypic resistance to bedaquiline, clofazimine, delamanid, any fluoroquinolone, and/or linezolid. The PP population retained participants from the mITT population who: 1) completed a protocol-consistent course of treatment (80% of expected doses taken within 120% of the regimen duration) or did not because of treatment failure or death; and 2) received less than 7 days of prohibited concomitant medication(s) and of study drug(s) not prescribed according to protocol. Other analysis populations are described in the Supplement.

### Statistical Analysis

Sample size assumptions included: week 73 favorable outcomes in 75% of participants in experimental groups, 70% of participants in the control, and relapse in 10%; 11% ineligible for mITT and 10% more ineligible for PP. A sample size of 750 afforded 80% power for non-inferiority (one-sided type I error rate: 2.5%) of 3 experimental regimens in the mITT and 2 in the PP populations. The non-inferiority margin was set at -12% because the control was designed to perform better than other recent standards.^13,14^ Slightly worse efficacy of experimental regimens was considered an acceptable tradeoff to achieve the shortening and pill-burden-reduction benefits of the experimental regimens. Lastly, three recent TB treatment trials used the 12% margin.^6,15,16^

The efficacy analysis relied on the absolute between-group difference in the percentages of participants with favorable outcome at Week 73. To sequence regimen comparisons, we used a hierarchical-testing approach (Supplement 2.7). Non-inferiority in the mITT population was established if the lower bound of the 95% confidence interval [CI] around the difference exceeded -12%. In this report, PP analyses provide complementary information but are not used for formal testing of a non-inferiority comparison. Risk differences were estimated using a binomial regression model (generalized linear model for a binomial outcome with an identity link function).

The primary analysis was unadjusted. Secondary analyses explored confounding by prespecified covariates (Supplement 2.7). Cox regression was used to estimate crude hazard ratios for time from randomization to unfavorable outcome and 95% confidence intervals (CI) for each experimental group. Schoenfeld residuals were used to test the proportional hazards assumption. Adjusted, subgroup, sensitivity, and post-hoc efficacy analyses are described in the Supplement (2.7). We estimated the frequency of death, SAEs, AESIs and AEs of Grade 3 or higher by group. For Grade 3 or higher AEs, we also estimated the frequency of events related to a study drug. All analyses were performed in Stata version 17.0.

## Results

### Trial populations and baseline characteristics

**Figure 1:**
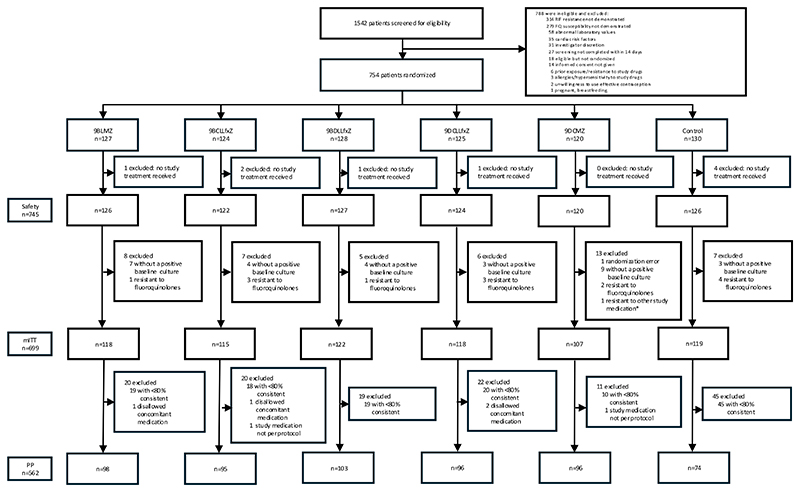
Participant flow diagram of study arms and analysis populations RIF denotes rifampin, FQ fluoroquinolone, mITT modified-intention-to-treat, PP per protocol; *bedaquiline, clofazimine, delamanid and/or linezolid

Between February 2017 and October 2021, 1542 individuals underwent screening and 754 were randomized. Nine participants were excluded from the safety population (N=745) and 46 from the mITT population, which comprised 699 participants. The PP population included 562 participants (Figures 1 and S4).

Overall, 264 (37.8%) participants were female. Median age was 32.0 years, 25 (3.6%) were less than 18 years of age; 98 (14.0%) were living with HIV, 568 (81.3%) had sputum smear results graded 1+ or above, and 57.1% had cavitation on chest radiograph. Baseline demographic and clinical characteristics are provided in Tables 1 and S10-S11 and stratified by country in S12. Modest variability in TB severity/history is observed by arm; expected differences in comorbidities (HIV, diabetes, hepatitis C) occur by country.

Control group regimens contained at least five drugs at start in 118 (99.2%) participants. Most (114, 95.8%) were longer regimens and 97 (81.5%) conformed to WHO 2022 recommendations^17^ (Tables S13-S14).

### Efficacy results

**Figure 2:**
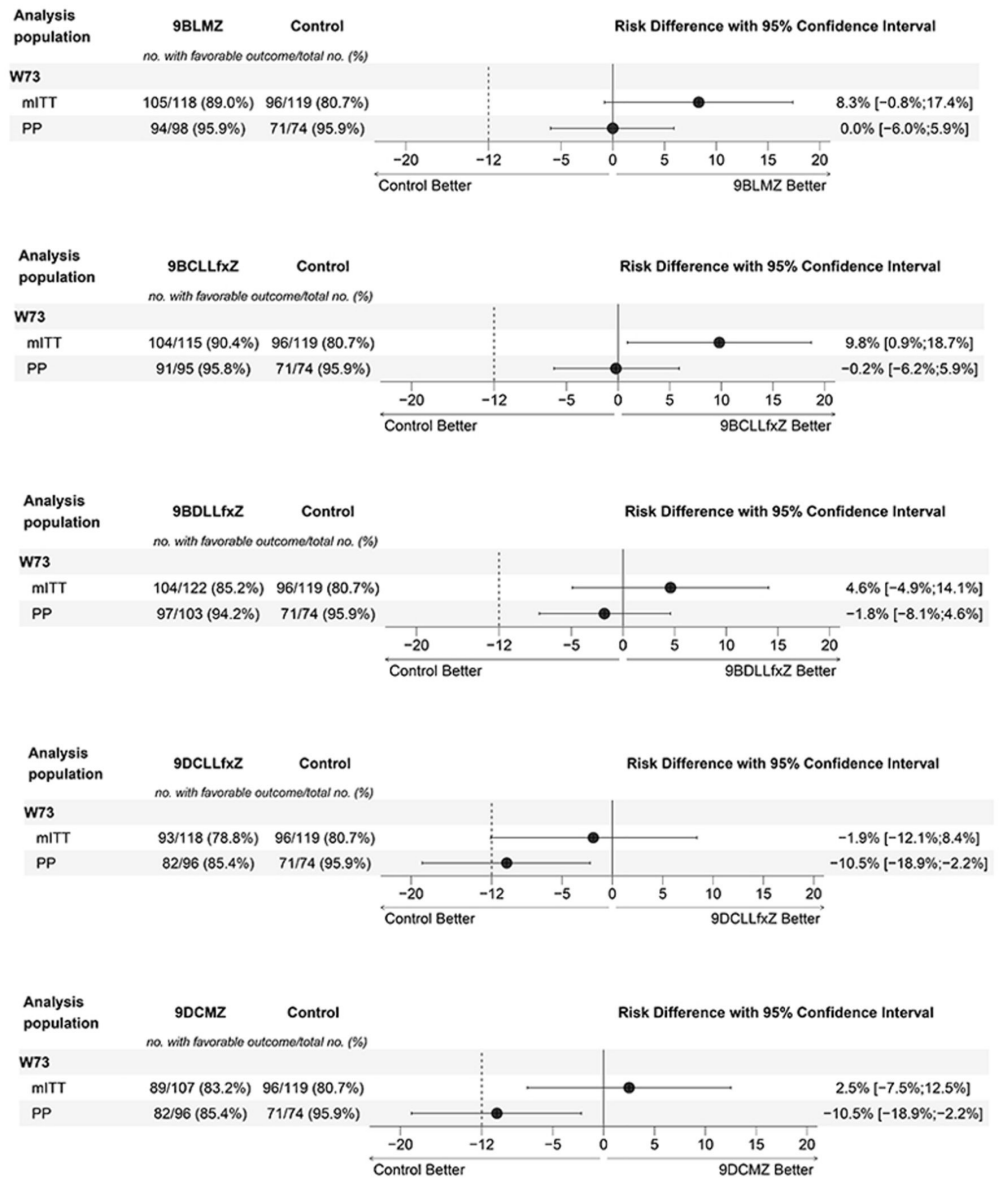
Forest plots of primary efficacy analyses at Week 73, by experimental group versus control Shows the results of the primary efficacy analysis in the modified-intention-to treat and complementary analysis in the per-protocol analysis populations (a. 9BLMZ vs. control, b. 9BCLLfxZ vs. control, c. 9BDLLfxZ vs. control, d. 9DLLfxZ vs. control, e. 9DCMZ vs. control). The noninferiority margin of -12 percentage points is designated by the dashed vertical line. Participants were classified as having a favorable outcome at week 73 if one of the following was true: 1) their last two culture results were negative and were taken from sputum samples collected on separate visits, the latest between Week 65 and Week 73; 2) the last culture result (from a sputum sample collected between Weeks 65 and 73) was negative and either there was no other post-baseline culture result or the penultimate culture result was positive due to laboratory cross contamination; and bacteriological, radiological and clinical evolution is favorable; or 3) there was no culture result from a sputum sample collected between Week 65 and Week 73 or the result of that culture was positive due to laboratory cross contamination, and the most recent culture result was negative, and bacteriological, radiological and clinical evolution was favorable. The modified-intention-to treat population included randomized participants with culture-positive, FQ-susceptible and MDR/RR-TB whose isolated M. tuberculosis strains were not determined to be resistant to bedaquiline, clofazimine, delamanid, fluoroquinolone, or linezolid. Participants who did not have a pre-treatment sputum culture positive for M. tuberculosis were also excluded from the modified-intention-to treat population. The per-protocol population was the modified-intention-to treat population excluding participants who, for reasons other than treatment failure or death, do not complete a protocol-consistent course of treatment. A protocol-consistent course of treatment was 80% of expected doses taken within 120% of the intended regimen duration. Participants who received more than 7 days of either a prohibited concomitant medication or a study drug not prescribed according to protocol were also excluded from the per-protocol population. Confidence interval widths have not been adjusted for multiplicity and should not be used in place of hypothesis testing.

In the primary, unadjusted outcome analysis of the control group, favorable outcomes occurred in 80.7% (95% CI, 72.4 to 87.3) in the mITT and in 95.9% (95%CI, 88.6 to 99.2) in the PP populations. Ordered comparison revealed that four experimental groups (9BCLLfxZ, 9BLMZ, 9BDLLfxZ, and 9DCMZ) were non-inferior to the control in the mITT population. Risk differences (RD) were: 9.8% (95%CI, 0.9 to 18.7) for 9BCLLfxZ, 8.3% (95%CI, -0.8 to 17.4) for 9BLMZ, 4.6% (95%CI, -4.9 to 14.1) for 9BDLLfxZ, and 2.5% (95%CI, -7.5 to 12.5) for 9DCMZ (Table 2, Figure 2). 9DCLLfxZ was not non-inferior in the mITT population. PP analyses supported these findings, except for 9DCMZ (Table S15, Figures S5a-S5e).

Unfavorable outcomes due to positive culture occurred in 4.1% of the full cohort, 7.5% of the DCMZ group, and in 10.2% of the 9DCLLfxZ group (Table 2). Loss to follow-up and consent withdrawal were more frequent in the control group than in experimental regimens. Overall, recurrence occurred in 3 (0.4%) participants. Efficacy outcomes were similar at secondary endpoints, week 39 (Tables S16-S17) and week 104 (Tables S18-S19, Figures S5a-S5e), in adjusted analyses (Tables S20-S25), and in sensitivity analyses (Tables S26-S28).

Overall, treatment effects at 73 weeks did not differ importantly in subgroup analyses in the mITT population. Possible exceptions for some groups include: country, prior exposure to second-line anti-TB drugs, cavitation, HIV coinfection, and low body mass index. Outcomes generally improved, while relative treatment effect did not change meaningfully, over the study period (Figures S6a-S6e). In the 9BCLLfxZ group, time to unfavorable outcome was longer than in the control (hazard ratio=0.48 [95% CI: 0.23-0.98]) (Figures S7a-S7e).

### Safety results

We report the number of participants in the safety population who experienced at least one of each safety event by Week 73 after randomization. The number with at least one Grade 3 or higher AE ranged from 54.8% (9BLMZ) to 61.4% (9BDLLfxZ) in experimental groups and was 62.7% in the control. SAE frequency was similar across groups: it ranged from 13.1% in 9BCLLfxZ to 16.7% in 9DCMZ and in the control. Overall, death from any cause occurred in 15 (2.0%) participants by 73 weeks (Table 3) and in 18 participants (2.4%) by 104 weeks; frequency was similar across treatment groups. No deaths were considered related to study drugs by the investigators (Table S29).

Among all Grade 3 or higher AEs and SAEs, 313/901 (34.7%) and 54/174 (31.0%), respectively were classified as related by the investigator to study drugs. At least one AESI was reported in 23.9% of all participants: the most frequent, hepatotoxicity, occurred in 7.1% of the control and its frequency in experimental groups ranged from 6.3% in 9BDLLfxZ to 18.3% in 9BLMZ. Hematologic toxicity occurred in 10.3% of control participants; in experimental groups, it ranged from 7.4% (9BCLLfxZ) to 10.5% (9DCLLfxZ). Peripheral neuropathy occurred in 4.8% in control and ranged from 2.4% (9DCLLfxZ) to 7.1% (9BDLLfxZ) in experimental groups. QTcF interval prolongation occurred exclusively in groups 9DCMZ (4.2%) and 9BCLLfxZ (3.3%). Other safety details, including drug discontinuations, are reported in Tables S30-S37. Ten (1.3%) participants became pregnant during study participation (Table S38).

## Discussion

Consistent results across all analyses support the non-inferior efficacy of three regimens (9BLMZ, 9BCLLfxZ, and 9BDLLfxZ) compared to the standard of care. These three regimens each produced favorable outcomes in more than 85% of participants at week 73; this represents an improvement over global averages and is comparable to trial results with the BPaLM regimen (88%).^1,6^

Death was uncommon despite the substantial burden of comorbidities and cavitary disease. Grade 3 or higher AEs were frequent across all groups, but typically considered by the site investigator as unrelated to study drugs. Although the study was not powered for statistical comparison of safety outcomes, we observed some patterns. Grade 3 or higher hepatoxicity was more common in experimental groups, except in 9BDLLfxZ, than in the control. Pyrazinamide, included in all experimental groups and almost 50% of the control regimens, can cause elevated liver enzymes, as can bedaquiline, fluoroquinolones, and linezolid; this can be aggravated by alcohol use and active hepatitis B or C infection, which were present in the cohort.^18–20^ Linezolid-related toxicities were generally less frequent in experimental groups than in the control and this may reflect a safety benefit of routinely lowering the weekly dose of linezolid at 16 weeks or earlier.^21–24^ QTcF intervals of >500 ms were infrequent and occurred only in groups containing clofazimine and a second major QT prolonger, bedaquiline or moxifloxacin. These results are consistent with emerging evidence about the safety of bedaquiline in combination with other QT-prolonging anti-TB drugs.^6,25,26^

Bayesian response-adaptive randomization permitted identification of multiple non-inferior TB regimens in a single study. Randomization was ultimately relatively balanced because experimental regimens performed similarly to the control on the interim endpoints used to adjust probabilities. Improved surrogate markers for treatment response will enhance efficiency of adaptive trials in TB.^27,28^

There are several limitations. Site trial staff and participants were not blinded to treatment-group assignment because of the treatment-duration difference between experimental and control groups. To mitigate risks of bias, we concealed treatment assignment and randomization probabilities from laboratory staff and central investigators. Bayesian adaptation and analysis for DSMB reports were performed by unblinded statisticians. During the trial enrollment period, WHO Guidelines changed twice. We incorporated these updates into trial guidance on the composition of control group regimens. The impact on regimen composition was modest because initial trial guidance had already been well aligned with newer WHO recommendations, to which 81.5% of control regimens conformed.^17^

Strengths of the trial include its randomized, internally, concurrently controlled design, which is essential to high certainty of evidence for guidance.^29^ Other strengths include the consistency of the findings across populations, endpoints, and analyses. Moreover, the control group performance, 80.7% favorable outcome, was better than that reported in other recent studies.^5,6,30–32^ That this improved standard could discriminate among well-performing regimens provides confidence in the efficacy of those found to be non-inferior. High retention of participants—including in the control group—and completeness of study data indicate high-quality implementation. The trial included adolescents and retained pregnant women. The population was heterogeneous, representing 4 continents, a range of TB disease severity, and substantial burdens of important comorbidities, all contributing to generalizability of study results to the broader population of people affected by MDR/RR-TB (Table S6).

These findings support the use of three new, all-oral, shorter MDR/RR-TB regimens in addition to BPaLM. In August 2024, WHO endorsed the use of these 3 regimens over the longer all-oral regimen.^33^ BPaLM was recommended by WHO in 2022 for use in non-pregnant people of 14 years of age or older.^17^ 9BLMZ, 9BCLLfxZ, and 9BDLLfxZ could be used in nearly all adults, children, and pregnant women with fluoroquinolone-susceptible MDR/RR-TB; all drugs in the endTB regimens have pediatric formulations and are recommended regardless of age.^34,35^ Findings are also relevant to pregnant women: all drugs included in the endTB regimens are considered to be acceptable for use during pregnancy.^17,36^ Two bedaquiline-sparing regimens (9DCMZ and 9DCLLfxZ) were examined: the overall assessment of these regimens does not support their use compared to a control that commonly contained bedaquiline. Favorable outcome frequency was higher than that reported in a recent trial testing a 9-month regimen containing neither bedaquiline nor clofazimine.^31^ However, among the groups receiving 9DCMZ and 9DCLLfxZ, unfavorable outcomes due to positive culture or recurrence were more frequent than in other regimens. Development of efficacious, shortened bedaquiline-sparing regimens requires further research.

Several implementation considerations arise. First, integrating endTB regimens allows simplification of country drug formularies while retaining a range of treatment options. Regimens may be selected according to individual patient characteristics and preferences; they offer alternatives for drug intolerances, interactions, contraindications, resistance, and stock-outs. Second, further development of—and access to—rapid, reliable, resistance testing is essential both to optimize patient selection for these regimens and to detect emergence of resistance.^37,38^ Finally, this study underscores the need for diligent monitoring of liver enzymes and of linezolid associated toxicities. Hepatotoxicity is a known risk with many anti-TB drugs, including pyrazinamide, a component of all endTB regimens.^17,19,35^ QT interval prolongation monitoring could be optimized through risk-based strategies, for example, intensifying monitoring in persons receiving multiple QT-prolonging drugs or with arrhythmia risk factors.^39,40^

The results of the endTB trial improve prospects for effective, simple, all-oral, and patient-centered treatment for adults and children with MDR/RR-TB.

Disclosure forms provided by the authors are available with the full text of this article at NEJM.org.

## Supplementary Material

Supplement

## Figures and Tables

**Table 1:** Baseline characteristics of participants in the mITT population

Characteristic	9BLMZ (N = 118)	9BCLLfxZ (N = 115)	9BDLLfxZ (N = 122)	9DCLLfxZ (N = 118)	9DCMZ (N = 107)	Control (N = 119)	Total (N = 699)
Female sex – no. (%)	41 (34.7%)	37 (32.2%)	55 (45.1%)	38 (32.2%)	45 (42.1%)	48 (40.3%)	264 (37.8%)
Median age, years (IQR) [range]	31.0 (25.0;41.0) [15.0;69.0]	38.0 (26.0;50.0) [15.0;70.0]	32.0 (22.0;45.0) [15.0;70.0]	30.5 (22.0;41.0) [15.0;69.0]	32.0 (24.0;46.0) [15.0;71.0]	31.0 (22.0;42.0) [15.0;70.0]	32.0 (23.0;44.0) [15.0;71.0]
Study country – no. (%)							
Georgia	2 (1.7%)	2 (1.7%)	1 (0.8%)	3 (2.5%)	1 (0.9%)	3 (2.5%)	12 (1.7%)
India	8 (6.8%)	4 (3.5%)	3 (2.5%)	3 (2.5%)	1 (0.9%)	4 (3.4%)	23 (3.3%)
Kazakhstan	30 (25.4%)	35 (30.4%)	33 (27.0%)	22 (18.6%)	24 (22.4%)	23 (19.3%)	167 (24.0%)
Lesotho	14 (11.9%)	11 (9.6%)	15 (12.3%)	11 (9.3%)	14 (13.1%)	12 (10.1%)	77 (11.0%)
Pakistan	18 (15.3%)	16 (13.9%)	13 (10.7%)	11 (9.3%)	16 (15.0%)	18 (15.1%)	92 (13.2%)
Peru	38 (32.2%)	39 (33.9%)	49 (40.2%)	54 (45.8%)	45 (42.1%)	51 (42.9%)	276 (39.5%)
South Africa	8 (6.8%)	8 (7.0%)	8 (6.6%)	14 (11.9%)	6 (5.6%)	8 (6.7%)	52 (7.4%)
Median body mass index (kg/m^2^) (IQR)	19.9 (17.5;22.1)	20.0 (18.4;23.6)	20.9 (18.8;22.8)	20.6 (18.1;23.6)	19.9 (17.9;22.2)	20.8 (17.6;23.0)	20.4 (18.0;22.8)
ECOG – no. (n/%)							
0	42 (35.6%)	35 (30.4%)	51 (41.8%)	47 (39.8%)	35 (32.7%)	43 (36.1%)	253 (36.2%)
1	55 (46.6%)	62 (53.9%)	53 (43.4%)	54 (45.8%)	53 (49.5%)	63 (52.9%)	340 (48.6%)
2	17 (14.4%)	15 (13.0%)	12 (9.8%)	16 (13.6%)	17 (15.9%)	11 (9.2%)	88 (12.6%)
3	4 (3.4%)	3 (2.6%)	6 (4.9%)	1 (0.9%)	2 (1.9%)	2 (1.7%)	18 (2.6%)
HIV, positive – no. (%)	15 (12.7%)	14 (12.2%)	17 (13.9%)	18 (15.3%)	15 (14.0%)	19 (16.0%)	98 (14.0%)
Median CD4 count among HIV-positive participants^†^ (IQR)	170.5 (41.0;505.0)	190 (85.0;377.0)	314.5 (157.0;478.5)	328.5 (170.5;579.5)	404.0 (143.0;643.0)	269.0 (83.0;443.0)	296.0 (118.0;497.0) (N=91)
Antiretroviral treatment among HIV-positive participants	12 (80.0%)	9 (64.3%)	10 (58.8%)	14 (77.8%)	11 (73.3%)	12 (63.2%)	68 (69.4%)
Hepatitis B, HbsAg positive – no. (%)	3 (2.5%)	3 (2.6%)	0 (0.0%)	2 (1.7%)	4 (3.7%)	4 (3.4%)	16 (2.3%)
Hepatitis C, positive – no. (%)	5 (4.2%)	5 (4.3%)	3 (2.5%)	4 (3.4%)	3 (2.8%)	6 (5.0%)	26 (3.7%)
Diabetes* – no. (%)	19 (16.1%)	19 (16.5%)	20 (16.4%)	16 (13.6%)	16 (15.0%)	15 (12.6%)	105 (15.0%)
Smear result – no. (%)							
Negative/Scanty	20 (16.9%)	19 (16.5%)	31 (25.4%)	24 (20.3%)	18 (16.8%)	19 (16.0%)	131 (18.7%)
1-2+	57 (48.3%)	59 (51.3%)	58 (47.5%)	49 (41.5%)	43 (40.2%)	52 (43.7%)	318 (45.5%)
3+	41 (34.7%)	37 (32.2%)	33 (27.0%)	45 (38.1%)	46 (43.0%)	48 (40.3%)	250 (35.8%)
Cavitation‡ – no. (%)	68 (57.6%)	69 (60.0%)	73 (59.8%)	53 (44.9%)	61 (57.0%)	75 (63.0%)	399 (57.1%)
Extent of TB disease^£§^ – no. (%)							
Limited	21 (17.8%)	14 (12.2%)	18 (14.8%)	23 (19.5%)	20 (18.7%)	18 (15.1%)	114 (16.3%)
Moderate	70 (59.3%)	77 (67.0%)	77 (63.1%)	67 (56.8%)	64 (59.8%)	71 (59.7%)	426 (60.9%)
Extensive	27 (22.9%)	24 (20.9%)	26 (21.3%)	25 (21.2%)	23 (21.5%)	29 (24.4%)	154 (22.0%)
Prior exposure to TB treatment^#^ - no. (%)							
None	76 (64.4%)	67 (58.3%)	78 (63.9%)	80 (67.8%)	72 (67.3%)	74 (62.2%)	447 (63.9%)
First-line drugs only	20 (16.9%)	23 (20.0%)	27 (22.1%)	25 (21.2%)	23 (21.5%)	31 (26.1%)	149 (21.3%)
Other drugs	15 (12.7%)	19 (16.5%)	15 (12.3%)	7 (5.9%)	11 (10.3%)	11 (9.2%)	78 (11.2%)
Pyrazinamide resistance^§&^ – no. (%)	57 (48.3%)	63 (54.8%)	66 (54.1%)	66 (55.9%)	66 (61.7%)	59 (49.6%)	377 (53.9%)
Second-line injectable resistance°^§&^ – no. (%)	14 (11.9%)	18 (15.7%)	15 (12.3%)	13 (11.0%)	14 (13.1%)	16 (13.4%)	90 (12.9%)

The modified-intention-to treat population included randomized participants with culture-positive, FQ-susceptible and MDR/RR-TB whose isolated *M. tuberculosis* strains were not determined to be resistant to bedaquiline, clofazimine, delamanid, fluoroquinolone, or linezolid. Participants who did not have a pre-treatment sputum culture positive for *M. tuberculosis* were also excluded from the modified-intention-to treat population. ECOG denotes eastern cooperative oncology group performance status, HIV human immunodeficiency virus, TB tuberculosis, HBsAg hepatitis surface antigen, and IQR interquartile range;

* Data on diabetes were missing for one participant;

† data on CD4 count were unknown for 7 participants;

‡ data on cavitation were unknown for 5 participants;

£ extent of TB disease was classified by Study Investigators, as follows: limited = presence of lesions with slight to moderate density, but no cavitations, not exceeding the size of the apex of the lung; moderate = lesions present in one or both lungs, not exceeding a) scattered lesions of slight to moderate density that involve the total volume of one lung or partially involve both lungs, b) dense, confluent lesions that extend up to one third of the volume of one lung, and c) cavitation with a diameter of < 4 cm in any single cavity; extensive = lesions that are more extended than those defined as moderate;

§ data on extent of TB disease, pyrazinamide, and second-line injectable resistance were unknown for 5 participants;

# data on previous exposure to TB treatment were unknown for 25 participants;

& phenotypic drug susceptibility testing; ° second-line injectables are amikacin, capreomycin, and kanamycin.

**Table 2:** Primary efficacy outcomes at Week 73 in the modified-intention-to-treat population

Outcome	9BLMZ (N = 118)	9BCLLfxZ (N = 115)	9BDLLfxZ (N = 122)	9DCLLfxZ (N = 118)	9DCMZ (N = 107)	Control (N = 119)	Total (N = 699)
**Favorable**							
Participants - no. (%)	105 (89.0%)	104 (90.4%)	104 (85.2%)	93 (78.8%)	89 (83.2%)	96 (80.7%)	591 (84.5%)
Absolute difference from control (%, 95% CI)	8.3% (-0.8%;17.4%)	9.8% (0.9%;18.7%)	4.6% (-4.9%;14.1%)	-1.9% (-12.1%;8.4%)	2.5% (-7.5%;12.5%)		
Participants with negative culture results, Week 65 and 73 – no. (%)	102 (86.4%)	100 (87.0%)	102 (83.6%)	90 (76.3%)	87 (81.3%)	91 (76.5%)	572 (81.8%)
Participants with favorable bacteriological, clinical and radiological evolution^^^ – no. (%)	3 (2.5%)	4 (3.5%)	2 (1.6%)	3 (2.5%)	2 (1.9%)	5 (4.2%)	19 (2.7%)
**Unfavorable**							
Participants – no. (%)	13 (11.0%)	11 (9.6%)	18 (14.8%)	25 (21.2%)	18 (16.8%)	23 (19.3%)	108 (15.5%)
Death, all cause – no. (%)^£^	2 (1.7%)	1 (0.9%)	3 (2.5%)	3 (2.5%)	2 (1.9%)	2 (1.7%)	13 (1.9%)
Participants with positive culture results^*^ – no. (%)	1 (0.8%)	3 (2.6%)	4 (3.3%)	12 (10.2%)	8 (7.5%)	1 (0.8%)	29 (4.1%)
Participants with recurrence^§^ – no. (%)	0 (0.0%)	0 (0.0%)	0 (0.0%)	1 (0.8%)	2 (1.9%)	0 (0.0%)	3 (0.4%)
Participants with permanent treatment discontinuation due to adverse event – no. (%)	3 (2.5%)	3 (2.6%)	1 (0.8%)	1 (0.8%)	1 (0.9%)	2 (1.7%)	11 (1.6%)
Participants with poor treatment adherence/lost to follow-up – no. (%)	3 (2.5%)	2 (1.7%)	3 (2.5%)	3 (2.5%)	4 (3.7%)	8 (6.7%)	23 (3.3%)
Participants who withdrew consent – no. (%)	1 (0.8%)	1 (0.9%)	4 (3.3%)	3 (2.5%)	0 (0.0%)	7 (5.9%)	16 (2.3%)
Participants with other unfavorable outcome^#^ – no. (%)	3 (2.5%)	1 (0.9%)	3 (2.5%)	2 (1.7%)	1 (0.9%)	3 (2.5%)	13 (1.9%)

The modified-intention-to treat population included randomized participants with culture-positive, FQ-susceptible and MDR/RR-TB whose isolated *M. tuberculosis* strains were not determined to be resistant to bedaquiline, clofazimine, delamanid, fluoroquinolone, or linezolid. Participants who did not have a pre-treatment sputum culture positive for *M. tuberculosis* were also excluded from the modified-intention-to treat population.

^ participants without culture results between Week 65 and Week 73;

£ 13 mITT participants experienced death as a treatment outcome, 1 participant in the safety population who was excluded from the mITT population also experienced death. 1 participant in the mITT population was assigned positive culture result as unfavorable outcome at 73 weeks and later died.

* participants who permanently discontinued treatment because of a positive sputum culture at Week 16 or later or who had a positive sputum culture between Week 65 and Week 73;

§ participants who, after treatment completion, had a positive sputum culture or started a new treatment regimen;

# participants with other unfavorable outcome: not assessable after completing treatment (n=6), investigator’s judgement (n=4), pregnancy or breastfeeding (n=2), use of prohibited concomitant medication (n=1). Confidence interval widths have not been adjusted for multiplicity and should not be used in place of hypothesis testing.

**Table 3:** Safety analysis at Week 73 in the Safety population

	9BLMZ (N = 126)	9BCLLfxZ (N = 122)	9BDLLfxZ (N = 127)	9DCLLfxZ (N = 124)	9DCMZ (N = 120)	Control (N = 126)	Total (N = 745)
Participants with any adverse event – no. (%)	126 (100.0%)	122 (100.0%)	127 (100.0%)	124 (100.0%)	120 (100.0%)	125 (99.2%)	744 (99.9%)
Grade 3 or higher adverse events							
Participants with ≥1 event– no. (%)	69 (54.8%)	68 (55.7%)	78 (61.4%)	75 (60.5%)	72 (60.0%)	79 (62.7%)	441 (59.2%)
No. of events	136	166	144	148	148	163	901
No. of events related to study drug(s) (% of all events)^^^	49 (36.3%)	57 (34.3%)	56 (38.9%)	58 (40.2%)	37 (25.0%)	56 (34.4%)	313 (34.7%)
Serious adverse events							
Participants with ≥1 event– no. (%)	18 (14.3%)	16 (13.1%)	20 (15.8%)	18 (14.5%)	20 (16.7%)	21 (16.7%)	113 (15.2%)
No. of events	26	29	30	26	31	32	174
No. of events related to study drug(s) (% of all events)^^^	7 (26.9%)	11 (37.9%)	11 (36.7%)	11 (42.3%)	6 (19.4%)	8 (25.0%)	54 (31.0%)
Death from any cause – no. (%)	3 (2.4%)	1 (0.8%)	3 (2.4%)	4 (3.2%)	2 (1.7%)	2 (1.6%)	15 (2.0%)
Adverse event of special interest							
Participants with ≥1 event– no. (%)	35 (27.8%)	33 (27.1%)	25 (19.7%)	33 (26.6%)	26 (21.7%)	26 (20.6%)	178 (23.9%)
Participants with any Grade 3-4 increase in ALT or AST– no. (%)	23 (18.3%)	17 (13.9%)	8 (6.3%)	18 (14.5%)	12 (10.0%)	9 (7.1%)	87 (11.7%)
Participants with any Grade 3-4 leukopenia, anemia, or thrombocytopenia – no. (%)	11 (8.7%)	9 (7.4%)	10 (7.9%)	13 (10.5%)	9 (7.5%)	13 (10.3%)	65 (8.7%)
Participants with any Grade 3-4 peripheral neuropathy – no. (%)	4 (3.2%)	5 (4.1%)	9 (7.1%)	3 (2.4%)	3 (2.5%)	6 (4.8%)	30 (4.0%)
Participants with any Grade 3-4 optic neuritis – no. (%)	0 (0.0%)	1 (0.8%)	0 (0.0%)	1 (0.8%)	0 (0.0%)	2 (1.6%)	4 (0.5%)
Participants with any Grade 3-4 QT corrected^*^ interval prolonged – no. (%)	0 (0.0%)	4 (3.3%)	0 (0.0%)	0 (0.0%)	5 (4.2%)	0 (0.0%)	9 (1.2%)
Participants with permanent discontinuation of any drug due to adverse event – no. (%)	26 (20.6%)	32 (26.2%)	35 (27.6%)	29 (23.4%)	19 (15.8%)	51 (40.5%)	192 (25.8%)

Safety population is all randomized participants who had received at least one dose of study treatment.

^ related is defined as at least a reasonable possibility to be caused by one or more drugs in the regimen;

* QT interval corrected according to the Fridericia formula. ALT denotes alanine transaminase, AST aspartate aminotransferase
