# Supplementary material for: Oral regimens for rifampin-resistant, fluoroquinolone-susceptible tuberculosis
Source: N Engl J Med. Author manuscript; Available in PMC 2025 Jan 30. (PMC7617355; doi:10.1056/NEJMoa2400327)
Supplement: Supplement [file EMS202805-supplement-Supplement.pdf]

Table of Contents

1 List of endTB Collaborators/Contributors (and study sites) 4

2 Methods 6

2.1 Objectives 6

2.1.1 Primary Objective 6

2.1.2 Secondary Objectives 6

2.1.3 Exploratory Objectives 6

2.2 Design and oversight 6

2.3 Participants & Sites 7

2.3.1 Full Inclusion & Exclusion Criteria 8

2.3.2 Retention of pregnant women in the endTB clinical trial 9

2.4 Randomization and treatment 9

2.4.1 Bayesian response-adaptive randomization and blinding 9

2.4.2 Treatment 11

2.4.3 Central management of study products 13

2.5 Procedures 14

2.6 Follow-up and Outcomes 15

2.6.1 Follow-up 15

2.6.2 Primary Efficacy Outcome 15

2.6.3 Secondary Efficacy Outcome 16

2.6.4 Evaluation of the bacteriological, radiographic and clinical evolution 17

2.6.5 Safety Assessment 17

2.7 Sample Size Assumptions and Analysis 18

2.7.1 Secondary analysis populations 18

2.7.2 Pre-specified adjusted, subgroup, sensitivity, and post-hoc adjusted analyses 18

2.7.3 Accounting for Multiplicity 19

3 Results 20

3.1.1 Efficacy 20

3.1.2 Safety 21

4 References 94

List of Figures

Figure S1. endTB Trial Design 22

Figure S2. Composition of endTB trial regimens 23

Figure S3. Allocation ratio through recruitment 24

Figure S4. CONSORT Group allocation during fixed and adaptive periods 25

Figure S5a. Primary and Secondary efficacy analyses of endTB Trial Regimen 9BLMZ vs Control at Week 73 and Week 104 26

Figure S5b. Primary and Secondary efficacy analyses of endTB Trial Regimen 9BCLLfxZ vs Control at Week 73 and Week 104 26

Figure S5c. Primary and Secondary efficacy analyses of endTB Trial Regimen 9BDLLfxZ vs Control at Week 73 and Week 104 27

Figure S5d. Primary and Secondary efficacy analyses of endTB Trial Regimen 9DCLLfxZ vs Control at Week 73 and Week 104 27
Figure S5e. Primary and Secondary efficacy analyses of endTB Trial Regimen 9DCMZ vs Control at Week 73 and Week 104 28
Figure S6a. Subgroup Analysis of Efficacy of endTB Trial Regimen 9BLMZ vs Control at Week 73 (mITT population) 29
Figure S6b. Subgroup Analysis of Efficacy of endTB Trial Regimen 9BCLLfxZ vs Control at Week 73 (mITT Population) 30
Figure S6c. Subgroup Analysis of Efficacy of endTB Trial Regimen 9BDLLfxZ vs Control at Week 73 (mITT Population) 31
Figure S6d. Subgroup Analysis of Efficacy of endTB Trial Regimen 9DCLLfxZ vs Control at Week 73 (mITT Population) 32
Figure S6e. Subgroup Analysis of Efficacy of endTB Trial Regimen 9DCMZ vs Control at Week 73 (mITT Population) 33
Figure S7a. Kaplan Meier Curve of Time to Unfavorable Outcome by Week 73: endTB Experimental Regimen 9BLMZ vs. Control (mITT population) 34
Figure S7b. Kaplan Meier Curve of Time to Unfavorable Outcome by Week 73: endTB Experimental Regimen 9BCLLfxZ vs. Control (mITT population) 35
Figure S7c. Kaplan Meier Curve of Time to Unfavorable Outcome by Week 73: endTB Experimental Regimen 9BDLLfxZ vs. Control (mITT population) 36
Figure S7d. Kaplan Meier Curve of Time to Unfavorable Outcome by Week 73: endTB Experimental Regimen 9DCLLfxZ vs. Control (mITT population) 37
Figure S7e. Kaplan Meier Curve of Time to Unfavorable Outcome by Week 73: endTB Experimental Regimen 9DCMZ vs. Control (mITT population) 38

List of Tables

Table S1. endTB Trial Protocol Writing Committee 39
Table S2. endTB Implementing Partners by Country 40
Table S3. endTB Data Safety and Monitoring Board 40
Table S4. endTB Scientific Advisory Committee 40
Table S5. Global TB Community Advisory Board 41
Table S6. Representativeness of Study Participants 41
Table S7. Weight-based dosing and frequency of administration of endTB trial study drugs (experimental arms) 43
Table S8. Weight-based dosing and frequency of administration of endTB trial study drugs (control arm) 44
Table S9. endTB trial Schedule of Events 45
Table S10. Other Baseline Characteristics of mITT Population 48
Table S11. Baseline phenotypic DST results – mITT population (N=699) 50
Table S12. Baseline characteristics of participants by country (mITT Population) 51
Table S13. endTB Trial Control Regimen composition and duration at initiation of treatment, by country (mITT Population) 53
Table S14. Distribution of endTB Control Group Regimens by WHO Guideline Period and Conformity with Guidelines 56
Table S15. Primary efficacy outcomes at Week 73 in the per-protocol population 57
Table S16. Unadjusted Analyses of Efficacy of endTB Trial Regimens at 39 Weeks (mITT Population) 58

Table S17. Unadjusted Analyses of Efficacy of endTB Trial Regimens at 39 Weeks (PP Population) 58
Table S18. Unadjusted Analyses of Efficacy of endTB Trial Regimens at 104 Weeks (mITT Population) 59
Table S19. Unadjusted Analyses of Efficacy of endTB Trial Regimens at 104 Weeks (PP Population) 59
Table S20. Adjusted Analyses of Efficacy of endTB Trial Regimens at 73 Weeks (mITT Population)........... 60
Table S21. Adjusted Analyses of Efficacy of endTB Trial Regimens at 73 Weeks (PP Population).............. 61
Table S22. Adjusted (pre-specified) Analyses of Efficacy of endTB Trial Regimens at 39 Weeks (mITT Population) 61
Table S23. Adjusted (pre-specified) Analyses of Efficacy of endTB Trial Regimens at 39 Weeks (PP Population) 62
Table S24. Adjusted (pre-specified) Analyses of Efficacy of endTB Trial Regimens at 104 Weeks (mITT Population) 62
Table S25. Adjusted (pre-specified) Analyses of Efficacy of endTB Trial Regimens at 104 Weeks (PP Population) 63
Table S26. Sensitivity Analyses of Efficacy of endTB Trial Regimens at 73 Weeks (Assessable, all-culture mITT, all-DST mITT, and Assessable Reclassified populations) 64
Table S27. Post-hoc sensitivity analysis of efficacy of endTB Trial Regimens at 73 Weeks (mITT population): reclassification of W73 treatment outcome of participants in control arm with longer regimen with addition or change of one or more investigational drug in their regimen as unfavourable outcome (as in experimental arms)........ 65
Table S28. Sensitivity Analyses of Efficacy of endTB Trial Regimens at 104 Weeks (Assessable, all-culture mITT, all-DST mITT, and Assessable Reclassified populations) 65
Table S29. Line Listing of Deaths in endTB Trial by Week 104 (Safety population) 67
Table S30. Number (%) of participants with adverse events (AEs) by Week 73 (Safety Population) 68
Table S31. Number (%) of participants experiencing adverse events (AEs) of grade 3 or higher by SOC and PT by Week 73 (Safety Population) 69
Table S32. Number (%) of participants experiencing adverse events (AEs) of grade 3 or higher by SOC and PT by Week 104 (Safety Population) 75
Table S33. Number (%) of participants experiencing serious adverse events (SAEs) by SOC and PT by Week 73 (Safety Population) 82
Table S34. Number (%) of participants experiencing serious adverse events (SAEs) by SOC and PT by Week 104 (Safety Population) 86
Table S35. Number (%) of participants with adverse events of special interest (AESI) of grade 3 or higher and time to onset by Week 73 (Safety population) 90
Table S36. Adverse events of special interest (AESI) of grade 3 or higher and time to onset by Week 104 (Safety population) 91
Table S37. Frequency of endTB trial Participants Experiencing Adverse Events Leading to (and Time to) Permanent Discontinuation of at Least One Study Drug by Treatment Group by Week 73 (Safety Population) 92
Table S38. Participant pregnancies, exposure information, and treatment decision during the endTB Trial 93

1 List of endTB Collaborators/Contributors (and study sites)

Aundh Chest Hospital, India (study site): Gurpreet Arora, Valay Ashara, Sonali Bhattacharjee, Rakshanda Borkar, Namita Chincholikar, Nikita Choudhary, Praveen Davuluri, Neha Desai, Dipika, Sunil Dhongade, Drishti Dulani, Sheetal Ghule, Nilma Hirani, Prakash Hurali, Reeana Irani, Kiran Jadhav, Pritesh Jadhav, Radhika Jadhav, Nidhin Joseph, Akshay Joshi, Pranav Joshi, Akhilraj K, Sagar K, Madhavi Kamble, Rashmi Kate, Tanvi Keer, Madhuri S Kenche, Harsha Khandare, Pranesha Koli, Apurva Kulkarni, Sagar Kumar Patra, Abhishek Kuwar, Prakash Lande, Nana Mane, Priya Masalge, Priya Mishra, Rishabh Mitkari, Ajit More, Pracheta Munot, Ramya Murgesh, Sagar Muthe, Nupur Nakashe, Dwaragesh Natarajan, Sharad Paril, Supriya Patil, Vaishali Patil, Vaijayanti Patwardhan, Vivek Posture, Anil Pradeshi, Vanya Prasad, Bhavya Pundir, Yogesh Randhit, Michael Reckart, Pradnya Samant, Hanif Shaikh, Ashutosh Sharma, Neela Sharma, Sharda Sharma, Nilesh Shinde, Suvarna Shinde, Gauri Shingare, Abhay Shulka, Aniket Shriwas, Lokesh Sonawane, Urmila Sudhindra, Dipali Suryavanshi, Akshay Thakare, Danashree Udare, Gayatri Vaidya, Rani Wagh

City Centre of Phthisiopulmonology, Kazakhstan (study site): Lyazzat Isenova, Gulbakhar Konigratbayeva, Gulnar Nurtayeva, Nurlan Zekeshov

Epicentre, France **:** Romain Chenu, Souna Garba, Daiane Tozzi, Francis-Jaudel Yuya-Septoh

Harvard Medical School, USA*:* Eva Chang, Julia Coit, Jacquelyn-My Do, Allison LaHood, Kelsey O'Brien, Enoma Okunbor, Elna Osso

Hospital Nacional Dos de Mayo, Peru (study site): Victor Chávez, Piero Larco, Daniel Mendoza, Christian Robles, Angelo Rojas, Janet Salazar, Gabriela Santos, Juan Sosa, Cesar Ticona

Hospital Nacional Hipólito Unanue, Peru (study site): Shirley Carranza, Diego Delgado, Cemir Galarza, Gladys Murga, Elizabeth Ramos

Hospital Nacional Sergio Bernales, Peru (study site): Kelly Cantaro, Andrely Huerta, Jonathan Maldonado, Roberto Nuñez, Katherine Panduro, Ruth Rafael

The Indus Hospital & Health Network, Pakistan (study site): Amaan Abdul, Sara Abedin, Eraj Afreen, Sohail Ahmed, Ubaid Ahmed, Jinsar Ali Shah, Azka Ashraf, Faisal Aslam, Zahida Bashir, Saba Fayyaz, Kiren Hafeez, Meherunissa Hamid, Basmah Hassan, Muzamil Hussain, Muhammad Irfan, Maria Omar, Shafiqua Parveen, Sharoon Sunail, Muhammad Farooq, Batool Mazhar, Sana Munir, Neelofer Sonia, Muhammad Yaseen, Muhammad Zubair, Fatima Zehra, Iqra Zulfiqar

Institute of Chest Diseases, Pakistan (study site): Tazeen Abbasi, Ghulam Akbar Abro, Mushtaque Ahmed, Nazir Ahmed, Muhammad Hammad Ali, Nazakat Ali, Rao Danish, Ayesha Farhat, Sunaina Gill, Muhammad Hafeez, Ghulam Hussain, Habib Inayat, Sylvia Johnson, Farees Kamal, Adil Kamran, Sanjeet Kumar, Mahveer Maheshwari, Shahid Mamsa, Sumera Massey, Asif Mehmood, Aaisha Memon, Ayaz Ali Mirani, Shafaque Naz, Tanzeel Rafique Qureshi, Yash Roop Moazzam Sheikh, Muhammad Rafi Siddiqui, Bhom Singh, Mustajab Soomro

Institute of Tropical Medicine, Belgium: Elisa Ardizzoni, Leen Rigouts, Praha Rupasinghe

Interactive Development and Research, Pakistan: Annum Aftab, Saman Ahmed, Muhammad Adnan Alamgir, Ayaz Ali, Azhar Ali, Shahzaib Ahmed Ali, Sakhawat Ali, Sanam Altaf, Samreen Amjad, Mahnoor Sahiba Arshad, Sadia Ausim, Abdul Basit, Haroon Ezik, Hira Hussain, Sibtain Hyder, Shahzad Inayat, Aleeza Janmohammed, Komal Jaseem, Aqsa Jawed, Lamis Maniar, Pardeep Kumar, Susheel Kumar, Reema Latif, Sara Liaqat, Muhammad Ammar Nasser, Muhammad Shakeel, Shahzaib Shaikh, Anique Siddiqui, Jetmal Singh, Koushalia Sivan, Momal Taimoor, Nabeel Zia

National Center for Tuberculosis and Lung Diseases, Georgia (study site): Tinatin Amirejibi, Rusudan Aspindzelashvili, Zaza Avaliani, Nino Bablishvili, Mariam Bichiashvili, Teona Bichashvili, Lasha Darchia, Marika Eristavi, Marine

Gachechiladze, Irine Keinashvili, Nino Kiria, Nani Kristesiashvili, Valerie Marechal, Darejan Meskhishvili, Lali Mikiashvili, Lia Trapaidze, Dali Zhizhilashvili, Maia Zhorzholiani

National Center for Tuberculosis Problems, Kazakhstan (study site): Inessa Li, Merei Otepbergenova

National Scientific Center of Phthisiopulmonology, Kazakhstan (study site): Gaukhar Abildayeva, Sagit Bektasov, Orazbayeva Maryash, Elena Telegina, Lyailya Tursynbekova

Partners In Health Kazakhstan, Kazakhstan: Zhanelya Amanzholova, Perizat Bektassova, Anel Belgozhanova, Nazerke Birimkulova, Nurgul Dyusebayeva, Nilay Erkut, Arnur Gusmanov, Banu Kassenova, Aigerim Kassym, Kanat Khazhidinov, Tatyana Lee, Azhar Magzumova, Moldir Mussabayeva, Gulnar Omarova, Nadezhda Ryapolova, Lyazzat Sagyndykova, Asel Serekbay, Assel Stambekova, Gulmira Tanatarova, Zinatdin Uaisov, Zhanel Zhantuarova, Gulnara Zhumakairova

Partners In Health, USA: Nataliya Arlyapova, Joshua Bogus, Meredith Cain, Merida Carmona, Clare Flanagan, Sarah McAnaw, Alyssa Scharff, Sonya Soni, Megan Striplin

Partners In Health Lesotho, Botsabelo MDR-TB Hospital, Lesotho (study site): Seyfu Abebe, Johnson Alakaye, Atang Bulane, Michael Custodio, Precious Hajison, Malefetsane Hetsa, David Holtzman, Matlotliso Khesa, Makatleho Lethola, Talime Lebitsa, Mpho Mahamo, Joalane Makaka, Mokenyakenya Matoko, Stephane Mpinda, Polo Mohoang, Daniel Monyaesa, Patrick Nkundayirazo, Mabohlokoa Ntsane (Sesomo Mohale), Seabata Ntsibane, Sebakeng Phate, Mathemba Radebe (Retsepile Tlali), Moliehi Rakhetsi, Tello Ranoosi, Meseret Tamirat, Mahooe Thokoana

Partners In Health Peru (Socios En Salud), Peru: Ericka Alegre, Christian Aguilar, Lourdes Armuto, Nadia Barreda, Maria Barreto, Stephany Cabrera, Roger Calderon, Milagros Castro, Jelina Chavez, Laura Chavez, Haydee Cheje, Lesli Cori, Daniel Dávalos, Silvana de la Gala, Karen Delgadillo, Hannely Diaz, Ximena Flores, Jimmy Galarza, Dalicxa García, Fanny Garcia, Miriam Gaspar, Roselena Godos, Pamela Gomez, Luz Gonzales, Lizsery Guerrero, Sadith Inga, Judith Jasaycucho, Ana Pro Martinez, Raquel Molina, Raquel Mugruza, Hansel Mundaca, Merilyn Nuahan, Kevin Ore, Liliana Panduro, Olga Peña, Sara Perea, Cynthia Pinedo, Yhomay Ponce, Claudia Quinte, Maritza Quiñones, Alicia Ramos, Elmer Ramirez, Rosina Reynoso, Cynthia Reyes, Jessica Reyes, Vanessa Riccio, Bryan Robles, Karen Rojas, Oswaldo Sanabria, Maria Saravia, Susana Saravia, Liz Senador, Jose Soberon, Edith Soncco, Maribel Soto, Catherine Suarez, Milagros Suarez, Veronica Suarez, Erika Torpoco, Oscar Torres, Claudia Torrez, Gabriela Tunque, Aida Ugarte, Gissela Vaderrama, William Valdivia, Yesica Valdivia, Isabel Valverde, Cynthia Vargas, Janet Vargas, Elvia Vasquez, Juan Veliz, Dioselinda Villa, Sheyla Villafuerte, Geraldine Villar, Stephanie Villegas, Milagros Wong, Rosa Yataco

Médecins Sans Frontières Khayelitsha, South Africa (study site): Neide Cossa, Stobdan Kalon, Christine Knel, Nopinky Matinse, Vuyokazi Mbanjwa, Axole Ndayi, Tasanya Nomaliso, Nelisiwe Ntali, Tabassum Rashid, Allan Taylor, Hamza van der Ross, Brenda Wright

Médecins Sans Frontières OCG, Switzerland **:** Sylvine Coutisson

Médecins Sans Frontières OCP, France : Melchior Atger, Sandra Nadia Baya, Marwa Bekhiet, Veronique Boissière, Roberta Caboclo, Marhaba Chaudhry, Sandrine Cloez, Sandra Collin, Céline Delifer, Shana Desmaisons, Vanessa Ducher, Cathy Hewison, Mohamed Ibrahim, Kristen Lebeau, Merry Mazmanian, Rada Mirzayeva, Mathilde Moreau, Monica Moschioni, Audrey Pâquet, Christophe Perrin, Laura Pichon, Jeanne Roussel, Martina Scotton

University of California San Francisco, USA **:** Pearl Sun

2 Methods

2.1 Objectives

2.1.1 Primary Objective

The primary objective is to assess whether the efficacy of experimental regimens at Week 73 is non-inferior to that of the control.

2.1.2 Secondary Objectives

1. To compare the efficacy of experimental regimens at Week 104 to that of the control
2. To compare the frequency of and time to early treatment response (culture conversion) in experimental regimens to that of the control
3. To compare the efficacy of experimental regimens at Week 39 to that of the control
4. To compare, at Week 73 and Week 104, the proportion of patients who experienced failure or relapse in the experimental arms to that in the control arm
5. To compare, at Week 73 and Week 104, the proportion of patients who died of any cause in the experimental arms to that in the control arm
6. To compare, at Week 73 and Week 104, the proportion of patients who experience grade 3 or higher AEs or SAEs of any grade in the experimental arms to that in the control arm
7. To compare, at Week 73 and Week 104, the proportion of patients who experience AESIs in experimental regimens to that in the control arm

2.1.3 Exploratory Objectives

1. To compare safety and efficacy endpoints across experimental arms, e.g., arms containing bedaquiline vs. delamanid (regimen 2 vs. 4); arms containing clofazimine & levofloxacin vs. arms containing moxifloxacin (regimen 2 vs. 1); arm containing bedaquiline and delamanid vs. arms containing one of the drugs and clofazimine (regimen 3 vs. 4 and regimen 3 vs. 2)
2. To evaluate conversion endpoints as potential surrogate markers for unfavorable outcome
3. To compare the activity of regimens between patients with strains that are resistant to important drugs in those regimens (e.g., pyrazinamide) and patients with strains that are sensitive
4. To estimate the frequency of drug resistance amplification among treatment failures and relapses occurring by Week 104
5. To compare treatment adherence in the experimental arms to that in the control and across regimens
6. To compare the frequency of and time to severe linezolid-related toxicity between linezolid dose-reduction strategies.
7. To compare, at Week 73 and Week 104, efficacy endpoints across linezolid dose-reduction strategies: 300 mg daily or 600 mg thrice weekly.

2.2 Design and oversight

The endTB protocol committee members (Table S2) designed the trial. The endTB protocol and statistical analysis plan are available at NEJM.org. The endTB consortium comprises Médecins Sans Frontières (MSF), Partners In Health (PIH), and Interactive Research and Development (IRD). Supervising institutional/ethical review boards were at Harvard Medical School (HMS), IRD, Institute of Tropical Medicine (ITM), and MSF. The data were collected by the site investigators and study teams. Data were analyzed at Epicentre and validated for the primary efficacy endpoint at the University of California, San Francisco (UCSF). The MSF Pharmacovigilance (PV) Unit provided support for standardized recording, reporting, grading and classification of adverse events. Safety data collection and transmission to the PV unit was the responsibility of the Site Principal Investigators and Site Co-Investigators. Adverse events were graded by the Site Principal Investigators and Site Co-Investigators according to the MSF Pharmacovigilance Unit Severity Scale. Site Principal Investigators and Site Co-Investigators were trained and received regular refresher trainings on the use of the

Severity Scale and on definitions and reporting of SAE/AESI to ensure harmonization across countries and sites. The PV Unit performed the medical review of each SAE and AESI reports. They evaluated each adverse event-investigational medicinal product pair: the expectedness and the Sponsor's assessment of the causality. Cases considered as potential Suspected Unexpected Serious Adverse Reactions (SUSAR) were directed to the Medical Review Board (MRB). The MRB comprised international DR-TB experts appointed by the PV Unit to review and confirm all potential SUSARs and assess the overall impact on participants. The PV Unit also provided support to the Clinical Advisory Committee (CAC) for safety-related eligibility questions, case management, medical monitoring, and permanent treatment discontinuation.

Other Committees

Data Safety and Monitoring Board (DSMB)
The endTB DSMB comprised individuals with expertise relevant to the endTB trial (Table S3). They met semi-annually to review safety and efficacy data, as well as protocol deviations. They reviewed criteria for stopping rules. They also received interim, quarterly reports on AESIs and reviewed safety halting rules. Their service was voluntary.

Scientific Advisory Committee (SAC)
The SAC was consulted on study design and important modifications throughout the study. They reviewed the statistical analysis plan. They received at least annual updates on study progress and changes. They remained blinded to treatment assignment and only saw summary data occasionally in the context of questions about possible modifications to design or reporting. Their composition is presented in Table S4. They also served as the SAC for TB-PRACTECAL. Their service was voluntary.

Global TB Community Advisory Board (TB-CAB)
The TB-CAB was consulted on study design and important modifications throughout the study. They reviewed study dissemination materials. They received at least annual updates on study progress and changes. They remained blinded to treatment assignment and did not see study data until after database lock when preliminary results were shared. Their composition is presented in Table S5. This is a pre-existing CAB and was not composed specifically for endTB. They were not compensated by endTB for their service.

endTB Laboratory Advisory Group (LAG)
The endTB LAG provided scientific oversight of issues that involved research on specimens and/or isolates collected from participants. They advised on the relevance and scientific validity of laboratory procedures and potential ancillary laboratory studies. They remained blinded to treatment assignment and did not see study data until after database lock when preliminary results were shared publicly. Their service was voluntary.

LAG members: Martina Casenghi (Elizabeth Glaser Pediatric AIDS Foundation), Daniella Cirillo (San Raffaele Scientific Institute), Ted Cohen (Yale School of Public Health), Claudia Denkinger (University Hospital Heidelberg), Kathleen Eisenach (TB or NOT TB Consulting), Megan Murray (Harvard Medical School).

endTB Clinical Advisory Committee (CAC)
The Clinical Advisory Committee (CAC) provided support to site investigators for the management of adverse events and possible treatment failure. They also adjudicated efficacy endpoints (see 2.6.4). As unblinded team members, they did not participate in discussions of the protocol or analysis. CAC members were: Catherine Berry (Médecins Sans Frontières), Jennifer Furin (Harvard Medical School), Ohanna Kirakosyan (Médecins Sans Frontières), Ilaria Motta (Médecins Sans Frontières).

2.3 Participants & Sites

Male or female patients with pulmonary TB who were age 15 and older and who had (risk factors for or) documented rifampin-resistant TB but not known fluoroquinolone-resistant TB were eligible to be consented and screened. Prospective participants were identified in inpatient or outpatient facilities in the study catchment areas. Patients who agreed to be evaluated for the study were referred to study staff for consent and screening.

Country and sites were selected for participation based on results of formal site capacity potential assessments, involvement of an endTB consortium partner in MDR care in the country, and inclusion of a heterogenous, representative study population. endTB participants are representative of the global population affected by TB, especially MDR/RR-TB. Table S1 uses TB notification data reported to WHO (1), estimates by WHO (2), and an individual patient data meta-analysis (IPD-MA) (3) to describe the broader population affected by TB and MDR/RR-TB globally. Inclusion of study participants from 7 countries across 5 WHO regions ensured representative heterogeneity of the study population. All the countries in which the endTB trial enrolled are themselves high-MDR/RR-TB burden countries (India, Kazakhstan, Pakistan, Peru, South Africa) or in regions where burden is high (Lesotho in southern Africa, Georgia in eastern Europe/central Asia). TB, including MDR/RR-TB, disproportionately affects the young: the median age of the endTB cohort was 32 (IQR: 23-44) similar to that in the most recent IPD-MA of MDR/RR-TB: 34 (24-45) years of age. More males than females are generally treated for MDR-TB as is the case in the endTB cohort. HIV is a comorbidity of variable prevalence by region; coinfection occurs in more than 50% in southern Africa; it's rarer (6.3%) globally. In endTB, 14% of the full cohort was HIV coinfected; >50% in the southern African sites and <5% elsewhere. In light of the similarities between endTB participants and the affected population, the results are likely generalizable.

2.3.1 Full Inclusion & Exclusion Criteria

Inclusion Criteria

1. Has documented pulmonary tuberculosis due to strains of *M. tuberculosis* resistant to rifampin and susceptible to fluoroquinolones, diagnosed by validated rapid molecular test;
2. Is ≥ 15 years of age;
3. Is willing to use contraception: pre-menopausal women or women whose last menstrual period was within the preceding year, who have not been sterilized, must agree to use contraception unless their partner has had a vasectomy; men who have not had a vasectomy must agree to use condoms;
4. Provides informed consent for study participation; additionally, a legal representative of patients considered minor per local laws should also provide consent;
5. Lives in a dwelling that can be located by study staff and expects to remain in the area for the duration of the study.

Exclusion Criteria

1. Has known allergies or hypersensitivity to any of the investigational drugs;
2. Is known to be pregnant or is unwilling or unable to stop breast-feeding an infant;
3. Is unable to comply with treatment or follow-up schedule;
4. Has any condition (social or medical) which, in the opinion of the site principal investigator, would make study participant unsafe;
5. a. Has had exposure (intake of the drug for 30 days or more) in the past five years to bedaquiline, delamanid, linezolid, or clofazimine, or has proven or likely resistance to bedaquiline, delamanid, linezolid, or clofazimine (e.g., household contact of a DR-TB index case who died or experienced treatment failure after treatment containing bedaquiline, delamanid, linezolid, or clofazimine or had resistance to one of the listed drugs); exposure to other anti-TB drugs is not a reason for exclusion.
   b. Has received second-line drugs for 15 days or more prior to screening visit date in the current MDR/RR-TB treatment episode. Exceptions include: (1) patients whose treatment has failed according to the WHO 2013 definition and who are being considered for a new treatment regimen; (2) patients starting a new treatment regimen after having been "lost to follow-up" according to the WHO 2013 definition and, (3) patients in whom treatment failure is suspected (but not confirmed according to WHO 2013 definition), who are being considered for a new treatment regimen, and for whom the Clinical Advisory Committee (CAC) consultation establishes eligibility.
6. Has one or more of the following:
   - Hemoglobin ≤ 7.9 g/dL;
   - Uncorrectable electrolytes disorders: o Total Calcium < 7.0 mg/dL (1.75 mmol/L);
     - Potassium < 3.0 mEq/L (3.0 mmol/L) or ≥6.0 mEq/L (6.0 mmol/L);

        - Magnesium < 0.9 mEq/L (0.45 mmol/L);
    - Serum creatinine > 3 x ULN;
    - Aspartate Aminotransferase (AST) or Alanine Aminotransferase (ALT) ≥ 3 x ULN;
    - Total bilirubin ≥ 3 x ULN;
    - Unless otherwise specified, Grade 4 result as defined by the MSF Severity Scale on any of the screening laboratory tests.
7. Has cardiac risk factors defined as:
    - An arithmetic average of the two ECGs with highest QTcF intervals of greater than or equal to 450 ms. Retesting to reassess eligibility will be allowed using an unscheduled visit during the screening phase;
    - Evidence of ventricular pre-excitation (e.g., Wolff Parkinson White syndrome);
    - Electrocardiographic evidence of either:
        - Complete left bundle branch block or right bundle branch block; OR
        - Incomplete left bundle branch block or right bundle branch block and QRS complex duration greater than or equal to 120 msec on at least one ECG;
    - Having a pacemaker implant;
    - Congestive heart failure;
    - Evidence of second- or third-degree heart block;
    - Bradycardia as defined by sinus rate less than 50 bpm;
    - Personal or family history of Long QT Syndrome;
    - Personal history of arrhythmic cardiac disease with the exception of sinus arrhythmia;
    - Personal history of syncope (i.e. cardiac syncope not including syncope due to vasovagal or epileptic causes).
8. Concurrent participation in another trial of any medication used or being studied for TB treatment, as defined in cited documents.
9. Is taking any medication that is contraindicated with the medicines in the trial regimen which cannot be stopped (with or without replacement) or requires a wash-out period longer than 2 weeks.

2.3.2 Retention of pregnant women in the endTB clinical trial

Women who became pregnant during study participation and whose pregnancy was not terminated could remain on study treatment if all of the following conditions were met:

- The clinical trial insurance policy subscribed in the country covered pregnant participants, including damage to and loss of the fetus;
- The local authorities and ethics committee(s) approved;
- The participant was at least 18 years of age;
- The Site PI considered that the expected benefits of continued treatment outweighed the risks of ongoing fetal exposure;
- The CAC reviewed the Site PI's recommendation
- The participant was informed of the therapeutic options, potential risks and expected benefits and sperate specific consent was obtained.

2.4 Randomization and treatment

2.4.1 Bayesian response-adaptive randomization and blinding

The endTB trial employed Bayesian response-adaptive randomization because of a possible efficiency advantage in studies with more than one effective regimen (4). Since endTB was exploring 5 experimental regimens and aimed to identify all of those that are effective, a design that permitted doing this as efficiently as possible was chosen. Randomization was not stratified by country or by any patient characteristic. With the 6 arms, and 7 countries selected to promote heterogeneity, stratification would have resulted in substantial increase in sample size.

A run-in period of fixed, balanced randomization across the 6 treatment groups was planned for the first 180 participants. We used a single randomization list, which was generated in Stata by the trial statistician and uploaded to the central Web-based system (Venn Life Sciences, Dublin, Ireland, v2.5), which site staff used to assign treatment group. Bayesian, responsive-adaptive randomization started after 185 patients were randomized (see Figure S3) and used blocks of varying

size. The adaptations were binding. The probability of assignment to each experimental arm then varied according to interim and end-of-treatment outcomes of participants progressing through the trial. The probability of assignment to the control arm was also controlled to approximately match the sample size of the highest enrolling experimental arm. The final sample size proportions were unknown prior to completion of randomization since they depended on the outcomes observed during the trial and changed over time. It was necessary to estimate such proportions anytime a new randomization list was generated.

Data used for adaptations (every 4 weeks):

- Week 8 culture results: culture negativity (TS8)
- Treatment outcome at Week 39: favorable outcome (TS39)

The algorithm used TS8 and TS39 to compute the posterior probability of non-inferiority to the control arm. The randomization probabilities favored allocation to the most promising regimens. For the control, the randomization probability was defined to approximately match the experimental arm with the highest number of enrolled patients.

TS73 (Favorable outcomes at week 73) was used to assess futility and/or superiority per pre-specified thresholds. Stopping an arm early would have been considered if the posterior probability of a regimen being non-inferior to the control at Week 73 fell below a threshold of 5%. Conversely, if the posterior probability was at least 95% that a regimen was superior to the control at Week 73, stopping for efficacy would have been considered. Results (whether or not the thresholds were met) were shared with data safety and monitoring board (DSMB). Had either threshold been met, the protocol specified that DSMB could further review safety and efficacy data and make a (non-binding) recommendation to stop an arm or the whole study. Neither threshold was ever met.

For TS8, any positive culture was dominant. The culture result was coded as follows:

- positive if either/both of 2 collected samples was positive
- negative if no positive and one sample was negative
- contaminated if at least one sample was contaminated and no sample was positive or negative culture
- missing, unable to produce sputum – if not able to produce sputum for either sample and there was no positive or negative culture
  missing sputum – not collected for other reasons

For combined results across 2 visits (e.g., Week 39 treatment outcome used Week 36 and Week 39 culture results), for each visit: the first sample collected was dominant if positive or negative; otherwise, the second collected sample was used; the result was contaminated in the absence of positive or negative cultures in the visit samples if at least one of them was contaminated. Then, the results of the two visits were combined as described below (for TS39 and imputation):

- positive if W36 (first in series) or W39 (second in series) was positive
- negative: if no positive and ≥1 negative
- contaminated: at least one contaminated and the other is contaminated or missing
- missing, unable to produce sputum – if not able to produce sputum for any sample at week 36 or week 39 and no positive or negative culture
- missing sputum – not collected for other reasons

Imputation was performed for TS8 and TS39 when culture results were contaminated or missing, unable to produce sputum. In addition, TS8 was imputed when TS8 result was missing and TS39 was not missing. We imputed missing Week 8 culture results to combined results at weeks 2 and 4. We imputed W36-39 culture result to combined results at weeks 32 and 36.

A Bayesian statistician updated the priors and the resulting randomization probabilities and communicated the new list each month to VennLife (the central interactive web/voice randomization system), from which site staff continued to assign treatment group. No central or site staff were informed of the randomization probabilities. The Bayesian statistician had no contact with trial participants or involvement in eligibility assessment or protocol decision-making. He had no involvement in analysis of study results, which was performed by the study statistician.

All other central study team members with protocol-decision making authority and responsibility for the analysis were blinded to treatment assignment. Mycobacteriology laboratory staff were also blinded to treatment assignment.

2.4.2 Treatment

Experimental arm treatment was prescribed for 9 months (39 weeks), 7 days/week. Weight-based dosing is in Table S6. One drug could be discontinued from experimental arm treatments with participants still considered to be on study treatment. No drugs could be added or substituted in experimental arms. Discontinuation of more than one drug or replacement/addition of any drugs in the experimental arms resulted in study treatment discontinuation (see *Permanent Discontinuation of Study Drug/Treatment*).

2.4.2.1 Experimental arm duration and combination

Duration

At the time the endTB trial was designed, recommended regimens were 18-24 months. Mouse models supported the idea that some combinations could permit regimen shortening. In 2016 WHO approved a shortened (9-11 month) 7-drug regimen for treatment of selected MDR-TB patients, those with limited resistance beyond isoniazid and rifampin. (5) The encouraging clinical results, combined with emerging animal and human data about the new and repurposed drugs, engendered confidence that regimens containing fewer drugs might also be effective at 9 months in populations with *M. tuberculosis* isolates resistant to more drugs. Shorter regimens were suggested to be possible by mouse models. But enthusiasm for going even shorter was dampened by the recent experience of "overly optimistic translation of the output from these [murine] studies into expectations of a 2-month treatment-shortening effect" in fluoroquinolone-containing regimens for drug-susceptible TB. (6) At the time of study planning in 2015-2016, in the absence of clinical data indicating the viability of regimens shorter than 9 months, we opted for 9 months duration.

Drug combinations

A number of preclinical studies had been performed prior to endTB design which lent support to the study combinations. (7) These studies suggested shortening potential of selected combinations by demonstrating effects on relapse-free survival, as well as on lung CFU counts during treatment. Combination studies focused primarily on newer anti-TB drug classes, bedaquiline (B) and nitroimidazoles [delamanid (D), pretomanid, TBA-354] or oxazolidinones [linezolid (L), sutezolid, posizolid]. These drugs were explored in combination with existing drugs, most often pyrazinamide (Z), moxifloxacin (M), and rifapentine and in some cases with other second-line oral and injectable agents.

Bedaquiline-pyrazinamide combinations containing, as a third drug one of the following: moxifloxacin, linezolid, rifapentine, or clofazimine all demonstrated bactericidal activity at least equal to or greater than the standard first-line regimen on fully susceptible strains. (7) The endTB regimens 9BLMZ, 9BCLLfxZ and 9BDLLfxZ built on this active nucleus of bedaquiline and pyrazinamide by adding a fluoroquinolone, levofloxacin (Lfx) or moxifloxacin, and a second drug with anti-TB activity. In mice, several 3-drug regimens (including BMZ, BZL, BZC) prevented all relapse after 4 months of treatment and at least 93% of relapse after 3 months of treatment. In the case of BZC, there was no relapse after only 2 months of treatment. (7)

endTB regimens 9BDLLfxZ, 9DCLLfxZ and 9DCMZ contain delamanid. At the time of study design, delamanid was selected as the nitroimidazole for several reasons. Although few studies had performed head-to-head comparisons between delamanid and pretomanid, available murine and *in vitro* data indicated that: Delamanid was more potent than pretomanid, and approximately equally potent to TBA-354, which ultimately was not developed. (8) And, selection for delamanid-resistant mutants occurred less frequently than selection for pretomanid-resistant mutants. (9) Additionally, clinical development of delamanid was more advanced than for pretomanid and TBA-354, having achieved conditional approval by a stringent regulatory authority, at the time the decision was made. Lastly, other trials were examining the role of pretomanid in shorter, all-oral regimens.

Animal data existed for the use of the other nitroimidazoles in combination. Results from studies containing a different class member could not be directly extrapolated to delamanid. Nevertheless, since delamanid was thought to be more active and less prone to selection for resistant mutants than pretomanid (and similar to TBA-354). (8, 9)

These studies were considered to offer complementary information for selection of regimens that adhere to the principles described above. Tasneen and colleagues showed consistent shortening potential in mice combining a nitroimidazole and an oxazolidinone. This combination was included in endTB regimens 9BDLLfxZ and 9DCLLfxZ. Linezolid, already approved for other indications, was registered in many countries with important MDR-TB problems, and available for inclusion in the trial regimens, unlike the other oxazolidinones.

Regimen 9BLMZ contains 3 drugs with bactericidal activity and 3 with sterilizing activity (bedaquiline, linezolid, pyrazinamide). The 2 major QT-interval prolongers in the regimen are bedaquiline and moxifloxacin. That this four-drug regimen represented a viable option for shortened treatment was supported by previously described mouse studies. (7) One of the 3-drug regimens that prevented all relapse after 4 months of treatment and at least 93% of relapse after 3 months of treatment is the nucleus of this regimen—bedaquiline, moxifloxacin, pyrazinamide—to which linezolid is added.

Regimen 9BCLLfxZ changes regimen 9BLMZ by replacing moxifloxacin with levofloxacin (to reduce the QT liability) and clofazimine. It starts with the nucleus of bedaquiline, pyrazinamide, and clofazimine, which resulted in no relapse after only 2 months of treatment in the mouse. (7) To this, the regimen adds linezolid and levofloxacin. It contains at least 2 drugs with bactericidal activity (bedaquiline, levofloxacin, and linezolid) as well as at least 3 (beyond pyrazinamide) with some sterilizing activity (bedaquiline, linezolid, and clofazimine). The two major QT-interval prolongers are clofazimine and bedaquiline. Levofloxacin was substituted for moxifloxacin to reduce the QT liability.

Regimen 9BDLLfxZ is the only regimen to contain both newly approved drugs, bedaquiline and delamanid. Like the others, it boasts at least 2 drugs with limited population exposure with both bactericidal and sterilizing activity. It has 1 major (bedaquiline) and 2 minor (delamanid and levofloxacin) QT-interval prolongers. No clinical data were available on the co-administration of bedaquiline and delamanid, though the DELIBERATE study later reported on PK and provisional safety and efficacy of the combination. (10) The efficacy of a bedaquiline-nitroimidazole (pretomanid and TBA-354) combination was well supported in mice. (9)

Regimen 9DCLLfxZ contains 3 drugs with some bactericidal activity (delamanid, linezolid, and levofloxacin) as well as 3 (+PZA) with treatment-shortening benefits (delamanid, linezolid and clofazimine). This 5-drug regimen contains 1 major (clofazimine) and 2 minor (delamanid and levofloxacin) QT-interval prolongers.

Regimen 9DCMZ offers 2 drugs with some bactericidal activity (delamanid moxifloxacin) as well as 2 (+PZA) with treatment-shortening benefits (delamanid and clofazimine). One of two four-drug, moxifloxacin-containing regimens, we anticipated a reduced toxicity profile compared to the five-drug regimens. It does, however, contain 2 major (clofazimine and moxifloxacin) and 1 minor (delamanid) QT-interval prolongers.

Full rationale is provided in the Background Information and Scientific Rationale section of the Protocol.

2.4.2.2 Control arm composition

For the control arm, regimen composition was guided by a study SOP, which in turn reflected current WHO guidance and local variations; these included the possibility of using of 9-month, all-oral “modified Bangladesh” regimens in India and South Africa in those whose prior exposure and known susceptibility were consistent with recommendations for its use. Otherwise, the longer regimen was used. No drugs could be added or substituted to the shorter, control regimen; one drug could be added or substituted to the longer regimen. The differences were because the longer, conventional regimen was an individualized regimen in which changes were expected. The experimental regimens and the modified Bangladesh regimens were standardized regimens; deviation from standardized regimens is considered to be relevant to their evaluation.

Control regimen treatment guidance was changed twice, in conformity with WHO Guideline updates. When new guidance took effect, starting 18 Dec 2018, endTB trial study procedures were updated according to the following principles:

1. Participants on treatment for less than one month: initiate new regimen constructed according to WHO 2018 recommendations
2. Participants on treatment for more than one moth with good treatment response: replace any injectables (amikacin, capreomycin, or kanamycin) with a Group A drug whenever possible. If Group A and B drugs were not appropriate, replace injectables kanamycin and capreomycin with amikacin.
3. Participants on treatment for more than on month with signs of slow or no treatment response: consider restarting a new treatment according to current recommendations

On 31 March 2020, further recommended changes were made according to the following principles for the shorter, all-oral MDR/RR-TB regimen:

1. Participants at any point of treatment with signs of good treatment response: if still taking an injectable, consider replacing amikacin with bedaquiline whenever possible.
2. Participants at any point of treatment with signs of slow or no treatment response: consider restarting a new conventional treatment according to 2020 WHO recommendations (taking note that resistance may have been developed to some of the drugs used in the regimen).

Changes within a class—fluoroquinolone, thioamide, or aminoglycoside/polypeptide—did not count as replacements.

As noted, the shorter MDR/RR-TB regimen was only offered as the control in countries where it was being used as the choice in the National TB Program for MDR/RR-TB.

Weight-based drug dosing was prescribed as recommended by WHO recommendations (see Table S8).

2.4.3 Central management of study products

MSF-Logistique was responsible for study product procurement and transportation. MSF-Logistique is a non-for-profit association under French 1901 law based in Bordeaux, France. It operates in compliance with the WHO Model Quality Assurance System for procurement agencies. MSF-Logistique is an approved "pharmaceutical establishment" by the French Ministry of Health since 1999. In 2007, MSF-Logistic obtained the certificate of Good Distribution Practices in its capacity as wholesale pharmaceutical establishment for humanitarian projects. It is also certified by the European Commission as international humanitarian procurement center.

Central procurement

To ensure standardization, study products were centrally procured. Below is the list of products centrally procured:

- **List of Investigational Products (IPs) procured**

IPs: amikacin, bedaquiline, clofazimine, cycloserine, capreomycin, delamanid, ethambutol, ethionamide, isoniazid, imipenem/cilastatin and amoxicillin/clavulanic acid, kanamycin, linezolid, para-aminosalicylic acid (PAS), prothionamide, pyrazinamide and terizidone

- **List of ancillary medicines procured**

Ancillary medicines to treat side effects: amitriptyline, beclometasone inhaler, biperiden, carbamazepine, chlorphenamine, clotrimazole vaginal tablet, diazepam, epoietin alfa syringe, fluoxetine, haloperidol, ibuprofen, levothyroxine, loperamide, magnesium oxide tablet & vial for injection, magnesium sulfate injectable, metoclopramide, miconazol cream, omeprazole, ondasetron, paracetamol, potassium chloride, prednisolone, promethazine, pyridoxine, risperidone, salbutamol inhaler, sodium chloride IV fluid, valproate sodium, water for injection.

- **Single-sourcing for IPs**

To ensure a steady supply and avoid bias throughout the whole duration of the trial, only a single manufacturer for each investigational product was selected. MSF-Logistic secured procurement agreements with the selected manufacturers for the duration of the clinical trial. The list of manufacturers for each IP is below.

Description	Manufacturer
AMIKACIN sulfate, eq. 250 mg/ml base, 2 ml, amp.	Medochemie
BEDAQUILINE, 100 mg, tab	Janssen
CAPREOMYCIN sulfate, eq. 1 g base, powder, vial	Vianex
CLOFAZIMINE, 100 mg, soft caps.	Novartis
CYCLOSERINE, 250 mg, caps.	MacLeods
DELAMANID, 50mg, tab., blister	Otsuka
ETHAMBUTOL hydrochloride (E), eq. 400 mg base, tab. blister	MacLeods
ETHIONAMIDE, 250mg tab	MacLeods
ISONIAZID,100 mg, breakable tab., blister	MacLeods
KANAMYCIN SULFATE, eq.to 1 g base, powder, vial	PanPharma
LEVOFLOXACINE hémihydrate, éq. 250 mg base, tab	Hetero
LEVOFLOXACINE hémihydrate, éq. 500 mg base, tab	MacLeods
LINEZOLID, 600 mg, coated tab.	Hetero
MOXIFLOXACIN hydrochloride, eq 400 mg base, tab.	Hetero
PARA-AMINOSALICYLIC acid (PAS),del.rel.gran, 4g, sach.(25°C)	Jacobus
PROTHIONAMIDE, 250 mg, tab.	Microlabs
PYRAZINAMIDE, 400 mg, tab., blister	MacLeods

- **Documentation provided to get import permit**

Certificate of Analysis (CoA), valid Certificate of Pharmaceutical Product (CoPP), Good Manufacturing Practices (GMP) certificate, artwork, package insert/patient information leaflet (PIL), Summary of product Characteristics (SmPC).

- **Continuous supply**

Continuous supply throughout the trial. A specific rotating stockpile for the trial was in place at MSF-Logistique warehouse to avoid any shortage.

Good Clinical Practice-compliant transportation

MSF-Logistique was responsible for ensuring appropriate transportation conditions for the drugs supplied to countries participating in the endTB Clinical Trial. All drug packages were labeled with specific endTB clinical trial stickers.

To be GCP compliant, the supplies were maintained at temperature between 15-25°C (or 2-8°C for Epoetin alpha only) all along the transport. Each packing box contained a data logger that started tracking as the parcel left MSF-Logistique warehouse until the box was opened in the central pharmacy of the destination country to demonstrate the compliance with the defined transport conditions.

Transport and assurance were provided up to the port of entry and required documentation supplied for the customs clearance (cargo manifest, packing list, invoices, certificate of donation, etc.).

Details of drug procurement and transportation are available in the endTB trial Pharmacy Manual.

2.5 Procedures

Study visits entailed: physical examination, symptom assessment, adverse event assessment, electrocardiograms, and blood and sputum (at least two per visit) sample collection at designated study facilities at each site. Treatment delivery/administration and sputum specimen collection could also occur in participants' homes or other place acceptable to participants and staff. Other study procedures could occur at these places or through remote visits during the COVID-19 pandemic or other in-country emergencies that limited movement of participants and staff. Hematology and biochemistry analyses were performed at designated clinical laboratories. Unscheduled laboratory tests were performed in other clinical laboratories as needed for participant safety. Phenotypic drug susceptibility testing was performed in

Mycobacteria Growth Indicator Tube (MGIT) at baseline and on late positive cultures at Week 16 or later at designated microbiology laboratories at each study site. Specific tests are described in more detail below. Adverse events were reviewed at every study visit, with investigators soliciting reports of signs or symptoms; daily treatment support visits provided another opportunity to ask participants about any new or worsening conditions. If reported, treatment supporters communicated the information to investigators and facilitated referrals for evaluation.

Sputum Specimen Testing

At least 2 sputa (spot or early morning) were collected at each visit at which sputum collection was indicated. When cultures were indicated, at least one was performed in MGIT; the other was performed in Löwenstein-Jensen medium except at the study lab in South Africa (National Institute for Communicable Diseases, which tested for both South Africa and Lesotho sites). During implementation of the endTB trial, this lab did not perform culture *M. tuberculosis* in Löwenstein-Jensen and therefore both cultures were performed in MGIT.

Recording and Central Overreading of ECGs

At screening and baseline visits (Visits 1 and 2), three ECGs were taken five minutes apart for each participant and recorded. At subsequent study visits (Visit 3-24; 27, and Early Termination, if applicable), two ECGs were taken five minutes apart and recorded. At screening and baseline, the ECG with the longest QT interval was sent to Clario (formerly eResearch Technologies) for central reading. For follow-up visit ECGs (Visits 3, 5, 7, 10, 15, 17, 19, 21, and 27), one ECG of best quality was sent for routine central reading. Clario overreads were also available for ECGs taken at unscheduled visits, as needed for safety.

2.6 Follow-up and Outcomes

2.6.1 Follow-up

Trial participation in all arms lasted until at least Week 73 and up to Week 104. Not all participants would have a study visit after Week 73. Sites conducted as many visits as possible until the end of the Week 73 window of the last participant randomized into the study. This “hybrid” follow-up option was chosen to balance the benefit of 2-year follow-up to capture data on relapse and the benefit of reporting trial results sooner (11).

2.6.2 Primary Efficacy Outcome

The primary efficacy outcome was the proportion of participants with favorable outcome at Week 73.

A participant’s outcome was classified as favorable at Week 73 if the outcome as not classified as unfavorable, and one of the following was true:

- The last two culture results are negative. These two cultures must be taken from sputum samples collected on separate visits, the latest between Week 65 and Week 73;
- The last culture result (from a sputum sample collected between Weeks 65 and 73) is negative; and either there is no other post-baseline culture result or the penultimate culture result is positive due to laboratory cross contamination; and bacteriological, radiological and clinical evolution is favorable;
- There is no culture result from a sputum sample collected between Week 65 and Week 73 or the result of that culture is positive due to laboratory cross contamination; and the most recent culture result is negative; and bacteriological, radiological and clinical evolution is favorable.

A participant’s outcome was classified as unfavorable at Week 73 in case of any of the following:

1. Replacement or addition of one or more investigational drugs in an experimental arm or in the control arm if using the shortened regimen (failure);
2. Replacement or addition of two or more investigational drugs in the control arm if using the conventional regimen (failure);
3. Initiation of a new MDR-TB treatment regimen after the end of the allocated study regimen and before Week 73 (recurrence);
4. Death from any cause;

5. At least one of the last two cultures, the latest being from a sputum sample collected between Week 65 and Week 73, is positive in the absence of evidence of laboratory cross contamination (failure/recurrence);
6. The last culture result (from a sputum sample collected between Week 65 and Week 73) is negative; AND
   - there is no other post-baseline culture result or the penultimate culture is positive due to laboratory cross contamination; and bacteriological, radiological or clinical evolution is unfavorable (failure/recurrence);
7. There is no culture result from a sputum sample collected between Week 65 and Week 73 or it is positive due to laboratory cross contamination; AND
   - the most recent culture is negative; and bacteriological, radiological or clinical evolution is unfavorable (failure/recurrence); or
   - the most recent culture result is positive in the absence of laboratory cross contamination
8. The outcome is not assessable because there is no culture result from a sputum sample collected between Week 65 and Week 73 or it is positive due to laboratory cross contamination; AND
   - there is no other post-baseline culture result or the most recent culture is positive due to laboratory cross contamination; or,
   - the most recent culture is negative and bacteriological, radiological and clinical evolution is not assessable;
9. Previously classified as unfavorable in the present study[i]

2.6.3 Secondary Efficacy Outcome

The secondary efficacy outcome was the proportion of participants with favorable outcome at Week 104.

A participant's outcome was classified as favorable at Week 104 if the outcome was not classified as unfavorable, and one of the following was true:

- The last two cultures are negative. These two cultures must be from sputum samples collected on separate visits, the latest between Week 97 and Week 104;
- The last culture result (from a sputum sample collected between Week 97 and Week 104) is negative; and either there is no other post-baseline culture result or the penultimate culture result is positive due to laboratory cross contamination; and bacteriological, radiological and clinical evolution is favorable;
- There is no culture result from a sputum sample collected between Week 97 and Week 104 or the result of that culture is positive due to laboratory cross contamination; and the most recent culture result is negative; and bacteriological, radiological and clinical evolution is favorable.

A participant's outcome was classified as unfavorable at Week 104 in case of any of the following:

1. Replacement or addition of one or more investigational drugs in the experimental arm or in the control arm if using the shortened regimen (failure);
2. Replacement or addition of two or more investigational drugs in the control arm if using the conventional regimen (failure);
3. Initiation of a new MDR-TB treatment regimen after the end of the allocated study regimen and before Week 104 (recurrence);
4. Death from any cause;
5. At least one of the last two cultures, the latest being from a sputum sample collected between Week 97 and Week 104, is positive in the absence of evidence of laboratory cross contamination (failure/recurrence);
6. The last culture result (from a sputum sample collected between Week 97 and Week 104) is negative; ***AND***
   - there is no other post-baseline culture result or the penultimate culture is positive due to laboratory cross contamination; and bacteriological, radiological or clinical evolution is unfavorable (failure/recurrence).

---

[i] Exception: A participant whose outcome was unfavorable because it was unassessable at Week 39 is eligible for re-evaluation at Week 73.

7. There is no culture result from a sputum sample collected between Week 97 and Week 104 or it is positive due to laboratory cross contamination; ***AND***
   - the most recent culture is negative; and bacteriological, radiological or clinical evolution is unfavorable (failure/recurrence);
8. There is no culture result from a sputum sample collected between Week 97 and Week 104 or it is positive due to laboratory cross contamination; ***AND***
   - there is no other post-baseline culture or it is positive; or,
   - the most recent culture is negative and bacteriological, radiological and clinical evolution is not assessable.
9. Previously classified as unfavorable in the present study;[ii]
10. Loss to follow-up[iii]

2.6.4 Evaluation of the bacteriological, radiographic and clinical evolution

The bacteriological, radiographic and clinical evolution was evaluated whenever necessary, that is when culture results were not available during the specified time period to allow culture-based outcome assignment. Briefly, evolution was evaluated as follows: bacteriological evolution entailed reviewing all sputum culture results and assessing the presence of acquired drug resistance; radiographic evolution entailed comparing the most recent chest X-ray to baseline with regards to the presence/size of lung cavities and the extension and/or new appearance of lung lesions; clinical evolution entailed comparing the most recent clinical evaluation to baseline with regards to performance status (measured with the Eastern Cooperative Oncology Group [ECOG] scale), body weight, and TB-related respiratory signs/symptoms (cough, hemoptysis, thoracic pain, dyspnea) and constitutional signs (fever, weight loss, night sweats or lack of appetite). The detailed procedure was described in a study SOP. All outcomes assigned using the evaluation of the bacteriological, radiographic and clinical evolution were reviewed through the event adjudication process (below).

Event Adjudication

In order to ensure the accuracy of the treatment outcome endpoints in the study and limit the variability across sites, an event adjudication process was established by which the CAC reviewed and validated the treatment outcomes assigned by the site investigators. An automated assignment algorithm, corresponding to the treatment outcome definitions, was programmed in Stata. During event adjudication, the CAC reviewed the treatment outcomes that were discordant between the site investigator and the algorithm as well as all those that had been established based on bacteriological, radiological and clinical evolution. Discrepancies between the treatment outcome emerging from CAC validation and site-investigator-assigned treatment outcome were discussed with site investigators. If the discrepancy was determined to be due to data incorrectly entered into the database or the site investigator changed their opinion to agree with the algorithm, data was modified, and the final outcome was reported as concordant.

2.6.5 Safety Assessment

Severity grading

The MSF Severity Scale was derived primarily from Division of Microbiology and Infectious Disease Adult Toxicity Table (Nov 2007) and Common Terminology Criteria for Adverse Events (v.4.03 14-Jun-2010).

Adverse Events of Special Interest

Adverse Events of Special Interest (AESI) could be serious or non-serious and were defined as a clinically important event that had the potential to be causally associated with a study drug and needed to be carefully monitored by the Sponsor. For the endTB trial, pre-determined AESI were grade 3 or higher of the following: leukopenia, anemia, thrombocytopenia,

---

[ii] Exception: a participant whose outcome is unfavorable because it is unassessable at Week 73 is eligible for re-evaluation at Week 104.

[iii] Loss to follow up is defined as participants for whom study staff have no information and whom they have been unable to contact for more than 14 weeks before last study visit per study schedule.

prolonged QT (average QTcF >500 ms or >60 ms change from baseline together with signs/symptoms of serious arrhythmia), optic neuritis, peripheral neuropathy, or hepatic toxicity.

Permanent Discontinuation of Study Drug/Treatment (see also Treatment)
Permanent discontinuation of study drug(s) was advised if a participant insisted on stopping study treatment. Additionally, permanent discontinuation of study drug(s) was considered on a case-by-case basis depending on the following circumstances: a) requirement for prohibited concomitant medications; b) any condition (social or medical) which, in the opinion of the site PI, made the study participant unsafe; c) indications of treatment failure including positive culture at Week 16 or later; or d) enrolled participant became pregnant (see *Retention in study of pregnant women* in Inclusion/Exclusion section). Permanent discontinuation of study drug(s) could also occur if a grade 3 or higher adverse event was considered related to the study drug(s) and did not resolve within 14 days. Any permanent discontinuation of a drug or treatment was discussed with the CAC and classification of permanent discontinuation required prior discussion with, and input from, the CAC.

2.7 Sample Size Assumptions and Analysis

We elected a 12% non-inferiority margin for several reasons. First, because the control included new and repurposed drugs and was expected to perform better than the global standard of care and better than the control arms in the pivotal trials of delamanid and bedaquiline.(12, 13) Second, the shortening and pill-burden benefits of the experimental regimens were substantial. Therefore, slightly worse efficacy of experimental regimens over the higher standard could be acceptable. Lastly, three recent treatment trials for drug-susceptible and drug-resistant TB—STAND, TRUNCATE and TB-PRACTECAL—used the 12% margin. (14-16)

2.7.1 Secondary analysis populations

All-culture mITT includes participants without pre-randomization cultures positive for *M. tuberculosis*. All-DST mITT includes participants with isolates resistant to bedaquiline, clofazimine, delamanid, fluoroquinolone, and/or linezolid. The assessable population includes participants from the PP population whose outcomes were classified as favorable or unfavorable and excludes those whose outcomes were unfavorable because they were not assessable. Also excluded from the assessable population are participants who experienced voluntary withdrawal, loss to follow up (LTFU), or confirmed reinfection. A fourth analysis population was created for sensitivity analyses. This “Assessable Reclassified” population is defined as the assessable population in which outcomes of “LTFU” or “Unfavorable because unassessable” are assigned the outcome that had been assigned at Week 39. Distinct populations were created for analyses of Week 73 and Week 104 endpoints.

2.7.2 Pre-specified adjusted, subgroup, sensitivity, and post-hoc adjusted analyses

Poisson regression model with robust standard errors was used for adjusted and subgroup analyses in light of convergence failures with binomial regression models. Adjusted estimate of risk difference with corresponding 95% confidence interval was calculated. We prespecified the following covariates for consideration for adjustment: country, BMI, pyrazinamide resistance, injectable resistance, age, sex, presence of each comorbidity at baseline or during study participation (including HIV, hepatitis B and C, diabetes, SARS-Cov-2 infection and COVID-19), smear result, lung cavitation presence at baseline, prior exposure to TB treatment, extent of disease (limited/moderate/extensive, based on chest X-ray at baseline) were defined *a priori* as covariates for consideration. Any covariate that was significant (p<0.10) in the univariate analysis was introduced into a multivariable model. We further prespecified that selection for inclusion in the final model would be made through the data-driven process of backward elimination; only variables significant at p<0.05 were retained in the final model.

Subgroup analyses
Sub-group analyses of the primary efficacy endpoint were performed (in both mITT and PP populations) for regimens that were shown to be non-inferior. Subgroups were established on the following baseline characteristics:

- Country (Georgia, India, Kazakhstan, Lesotho, Pakistan, Peru, South Africa)
- Presence of comorbidities (presence/absence of: HIV, hepatitis C, diabetes, and SARS-CoV-2 infection/COVID-19)
- BMI (<18.5 kg/m2, 18.5-24.9 kg/m2, ≥25 kg/m2)
- Pyrazinamide resistance (sensitive/resistant)
- Age (<18, 18-45, ≥45)

- Sex (male/female)
- Smear result (at screening) (positive/negative)
- Cavitation (presence/absence)
- Prior exposure to TB treatment (none/first line only/other)
- WHO recommendations implemented (by implementation date).

Sensitivity analyses
The primary unadjusted and adjusted efficacy analyses were performed for week 73 outcomes:
- in the following populations: 1) assessable, 2) all-culture mITT, and 3) all-DST mITT populations.
- in the assessable population reclassifying outcomes of "LTFU" or "unfavorable because unassessable" as the outcome that had been assigned at Week 39

In addition, as a post-hoc sensitivity analysis we examined the effect on 73-week outcomes of increasing the strictness of the definition for regimen change as a reason for unfavorable outcome as applied to the longer regimens in the control arm. The protocol specified that unfavorable outcome was declared for experimental arm (and shorter-regimen control-arm) participants who had ≥1 drug added and for longer-regimen, control-arm participants who had ≥2 drugs added. In the sensitivity analysis, we assigned unfavorable outcome to ALL participants, experimental and control (longer and shorter regimens) who had ≥1 drug added.

We did not perform the following planned subgroup and sensitivity analyses:
- Subgroup analysis by SARS-CoV-2 infection/COVID-19. Although we adapted data collection (when the protocol was amended) to capture information on SARS-CoV-2 infections, we did not record information that permitted discernment of the timing of the infection relative to study participation (i.e., pre-enrollment, at screening/baseline or on-treatment) and the type of test used (antibody, antigen, or RT-PCR). Since the observed number with COVID-19/SARS-CoV-2 infections was relatively small, and because all other characteristics used for subgroup analyses were baseline characteristics, we thought a stratified analysis would be very difficult to interpret, if not misleading.
- excluding participants from the control arm who received a shortened regimen, because the proportion did not exceed the pre-defined threshold of 10%
- using multiple imputation to impute missing data after examination of the missing data pattern, because the proportion did not exceed the pre-defined threshold of 20%. Data were missing for only 0.7% of observations.

Post-hoc adjusted analyses
Because there was some variability in distribution of covariates observed, we performed a second set of adjusted analyses to evaluate confounding. Covariates included in the multivariable model were pyrazinamide resistance, HIV status, sputum smear microscopy grade, and cavitation on chest X-ray.

2.7.3 Accounting for Multiplicity
We prespecified the hierarchical testing or fixed-sequence approach. For the present report, the regimen with the highest proportion of favorable outcomes in the mITT population was compared to the control first; if the lower bound of the 95% confidence interval exceeded the non-inferiority margin (-12%), then a comparison between the control and the experimental regimen having the second highest proportion of favorable outcomes in the mITT population was performed. Comparisons were planned to continue until all regimens were evaluated in sequence in the mITT and then in the PP populations or until the lower bound of the 95% confidence interval around an estimate did not exceed -12% in one of the sequenced comparisons, whichever came first. Results of hypothesis testing of non-inferiority in the PP population are not reported. Regimens that met the threshold in mITT, with supporting information in the PP, were considered to be non-inferior to the control. All comparisons were done at the full alpha level. As such, confidence interval widths have not been adjusted for multiplicity and should not be used in place of hypothesis testing for secondary or sensitivity analyses.

3 Results

First patient, first visit occurred on 22 February 2017; last patient, last visit was completed on 08 April 2023 when the last participant completed 73 weeks of post-randomization follow-up.

Among the 1542 people screened because they had tuberculosis and risk factors for resistance, more than half (788) were ineligible for enrollment. The largest number was excluded because resistance to rifampin was not demonstrated (314) or because susceptibility to fluoroquinolones was not established (279). The endTB regimens are only appropriate for TB patients with known or likely resistance to rifampin and susceptibility to fluoroquinolones. For that reason, exclusion of this group does not represent a source of bias. The remainder of ineligible participants (195, 25% of those ineligible) met a range of exclusion criteria, most commonly safety reasons (58 for abnormal laboratory values and 35 for cardiac risk factors).

Baseline lab/safety measures reported in Table S10 reveal expected variability across arms. Baseline characteristics are stratified by country in Table S12. Prevalence of: HIV is higher and diabetes is lower in southern Africa; hepatitis C is higher in eastern Europe and Central Asia; cavitation is higher in Kazakhstan and Peru (which together represent nearly 65% of the study population). BMI was lower in south Asia. These differences were expected and support the generalizability of the study results.

We report the distribution of the use of individual drugs and regimens in the control group by country (Table S13). Small differences can be observed in the use or choice of injectables, delamanid, and ethionamide/prothionamide. However, almost two-thirds (77 out of 119) of the participants received regimens containing bedaquiline, clofazimine, linezolid, and levofloxacin and another 5 (4.2%) received regimens containing bedaquiline, clofazimine, linezolid, and moxifloxacin. That a large number of participants received at least 3 Group A and 1 Group B drugs reinforces both the strength of the control arm and its relative uniformity. Changes over time can be observed in Table S14. Control group participants received regimens containing an injectable agent during only the first WHO Guideline period (prior to 18 Dec 2018). These 22 participants (18.5% of all control group participants) were the only ones to receive regimens that were not compliant with 2022 Guidelines. (See *Treatment*)

3.1.1 Efficacy

Adjusted analyses
Prespecified adjusted analyses supported the primary results. The adjusted risk difference (aRD) for 9BCLLfxZ relative to the control was 9.5% (95%CI, 0.4 to 18.6) in mITT and 0.3% (95%CI, -6.1% to 6.7%) in the PP population. aRD for 9BLMZ and 9BDLLfxZ in the mITT population were 8.8% (95%CI, -0.6 to 18.2) and 3.9% (95%CI, -5.8 to 13.6) respectively. In the PP population, these estimates were 0.1% (95%CI, -6.2 to 6.4) and -2.9% (95%CI, -10.2 to 4.4) respectively. Relative to the control in the mITT population, 9DCMZ had an aRD of 1.9% (95%CI, -8.4 to 12.3) and in the PP population aRD of -10.8% (95%CI, -19.3 to -2.2) (Figures S5a-S5e and Table S16-S17).

Cox proportional hazards models
There was no evidence that the proportional hazard assumption was violated for the time from randomization to unfavorable outcome. The crude hazard ratios for time from randomization to unfavorable outcome and 95% confidence intervals for each experimental group are shown in figures S7a-S7e. They generally support the findings from the primary analysis.

Sensitivity analyses of favorable outcomes at week 73
Table S18 reports results of pre-specified sensitivity analyses in additional analysis populations. Table S19 shows results of the post-hoc sensitivity analysis (recommended by a reviewer) of the stricter regimen-change rule as applied to the longer regimen in the control arm. Considering as unfavorable any change of ≥1 drug (instead of the pre-specified ≥2 drugs) in longer regimens in the control arm results in poorer observed performance of the control arm: % favorable outcome declines from 80.7% to 68.1% in the mITT population. The primary results are supported for endTB regimens 9BLMZ, 9BCLLfxZ, 9BDLLfxZ, and 9DCMZ. Using this approach, the performance of endTB regimen 9DCLLfxZ improves relative to the control (RD: 10.7%; 95% CI: -0.4%;21.9%).

3.1.2 Safety

Deaths

The most common causes of death were disease progression (5 participants) and sepsis (3 participants) [Table S36]. Two participants were found dead at home: sudden death could not be excluded although it was considered unlikely by study investigators and medical review committee.

Permanent Drug Stops due to adverse events

Permanent drug stops due to AEs occurred in 40.5% of participants in the control and in between 15.8% (9DCMZ) and 27.6% (9BDLLfxZ) in the experimental groups (Table S37). The most frequently stopped drugs were pyrazinamide (108 [14.5%] participants at median 92 [IQR: 57.5-184.5] days post randomization) and linezolid (66 [8.9%] participants at median 164 [IQR: 112-225] days post randomization).

Figure S1. endTB Trial Design

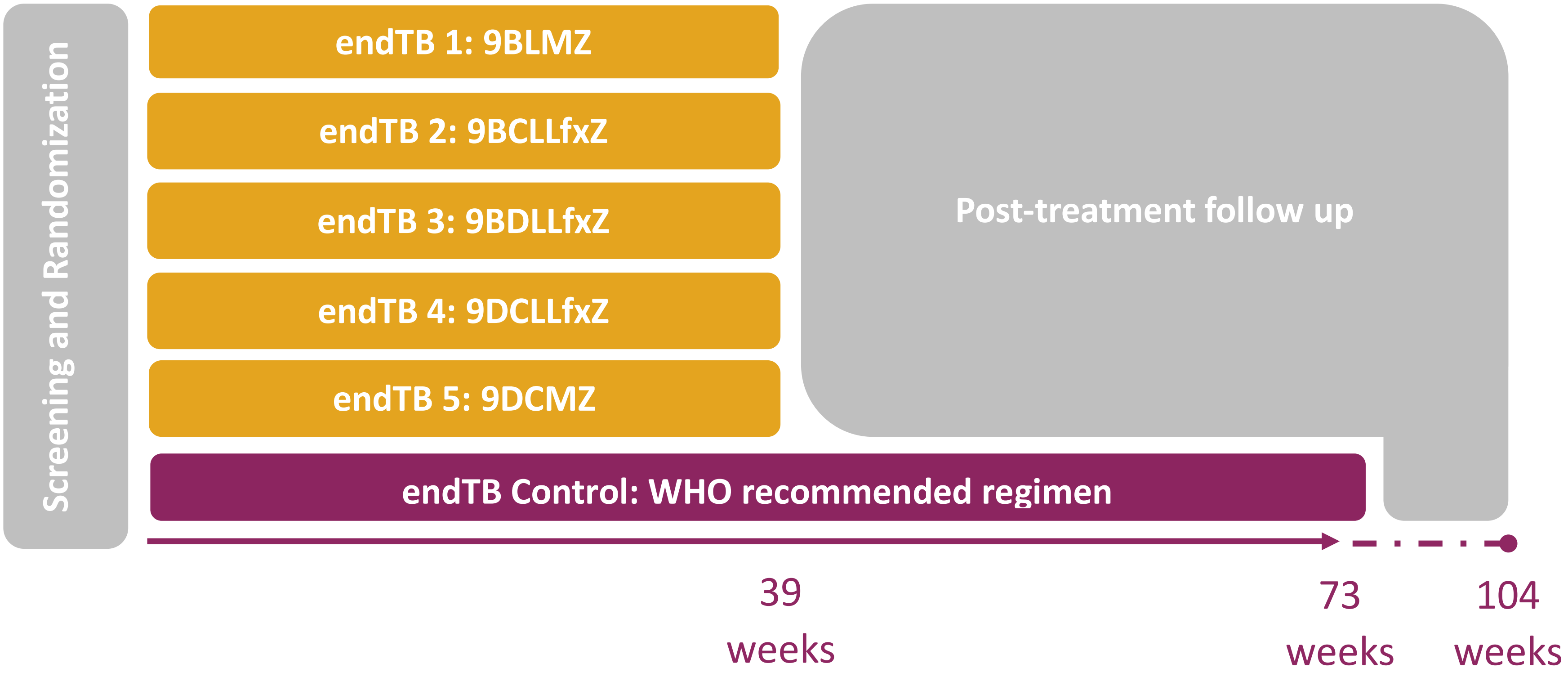

Figure S2. Composition of endTB trial regimens

Trial regimens	Bedaquiline	Delamanid	Clofazimine	Linezolid	Fluoroquinolone	Pyrazinamide
**9BLMZ**	B			L	M	Z
**9BCLLfxZ**	B		C	L	Lfx	Z
**9BDLLfxZ**	B	D		L	Lfx	Z
**9DCLLfxZ**		D	C	L	Lfx	Z
**9DCMZ**		D	C		M	Z
**Control**	Standard of care for the treatment of rifampin-resistant and fluoroquinolone-susceptible tuberculosis. Composed according to latest World Health Organization guidelines, as they evolved during the trial. This group included mostly participants treated with the 18-month conventional regimen.					

B denotes bedaquiline, L linezolid, M moxifloxacin, Z pyrazinamide, C clofazimine, Lfx levofloxacin, D delamanid

Figure S3. Allocation ratio through recruitment

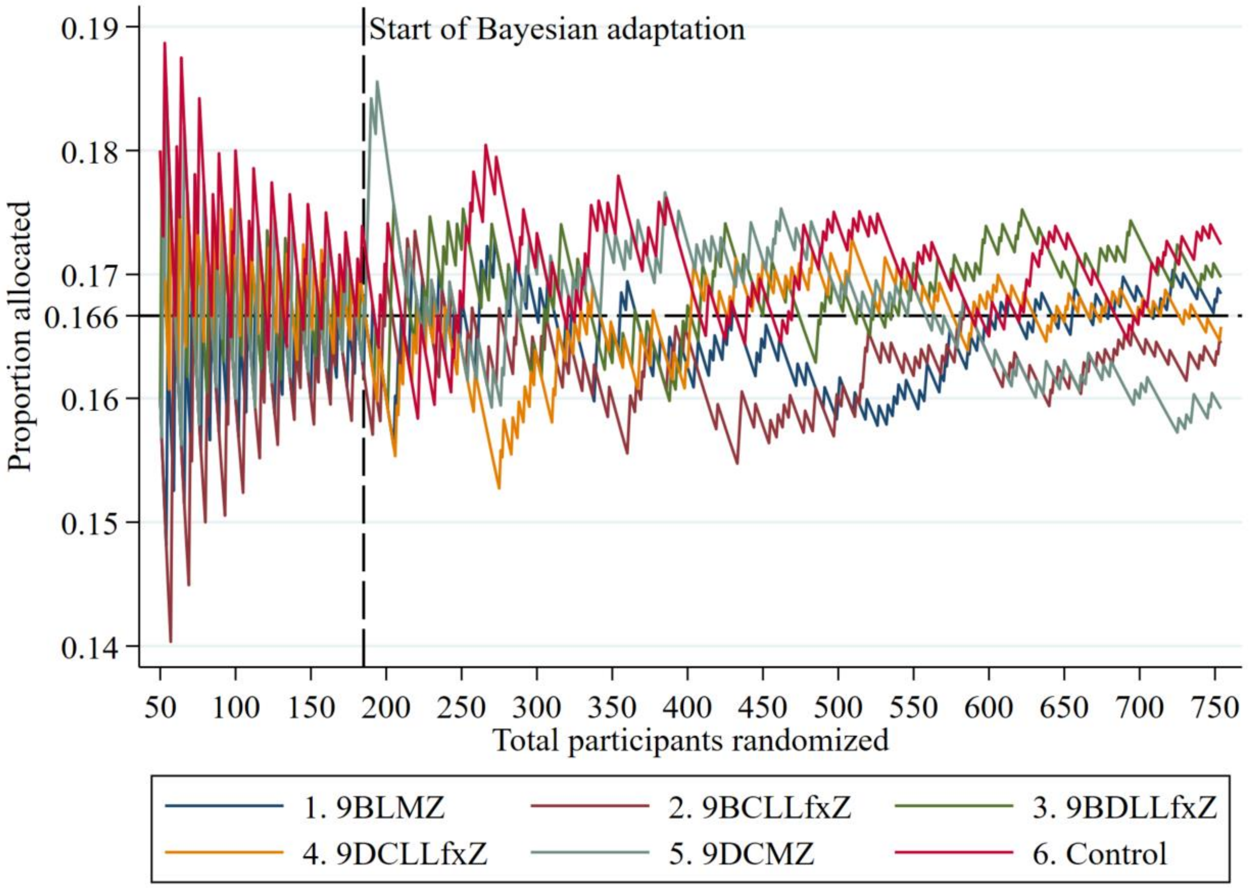

Figure S4. CONSORT Group allocation during fixed and adaptive periods

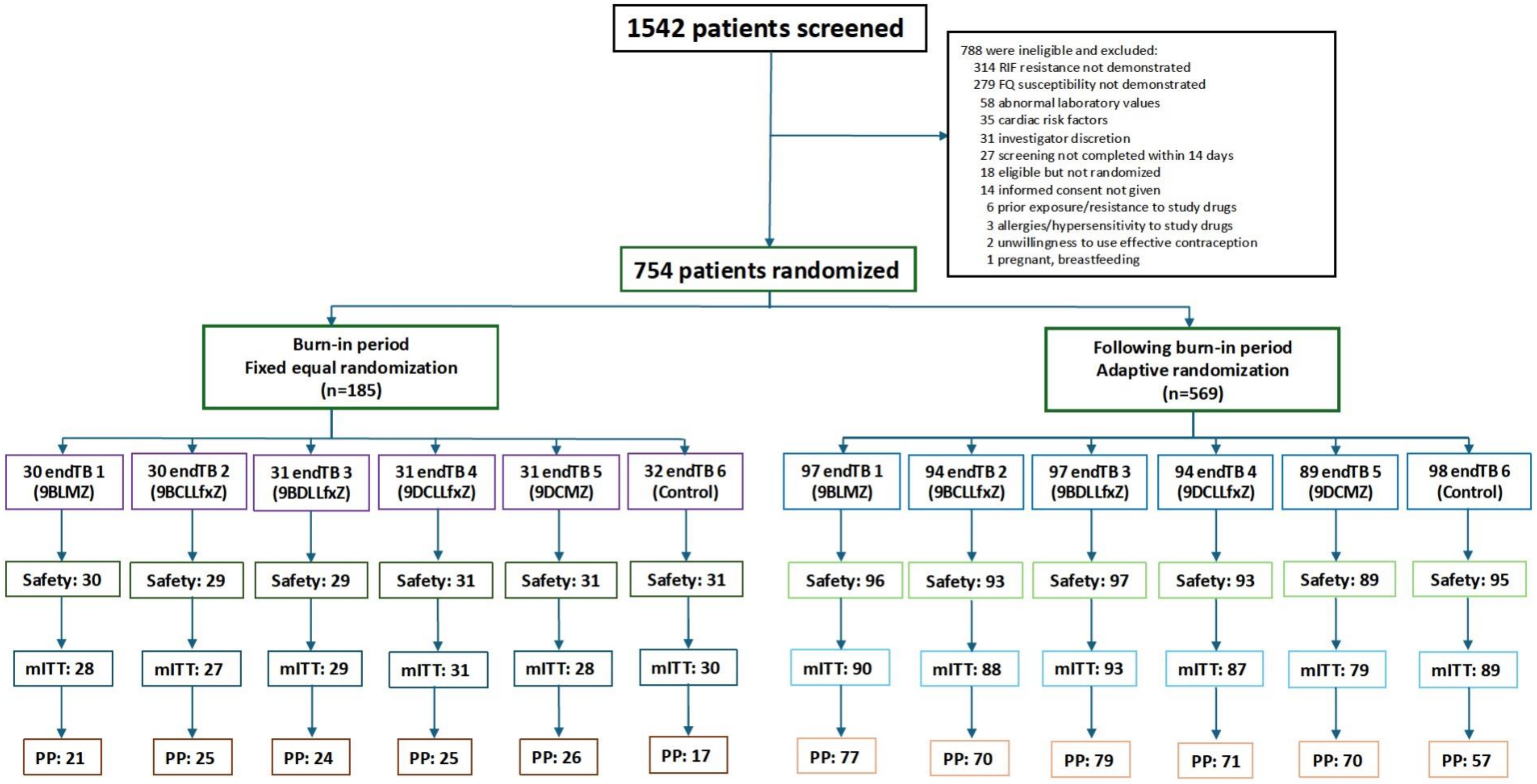

RIF denotes rifampin, FQ fluoroquinolone, mITT modified-intention-to-treat, PP per protocol; B=bedaquiline, C=clofazimine, D=delamanid, L=linezolid, Lfx=levofloxacin, M=moxifloxacin, Z=pyrazinamide;

Figure S5a. Primary and Secondary efficacy analyses of endTB Trial Regimen 9BLMZ vs Control at Week 73 and Week 104

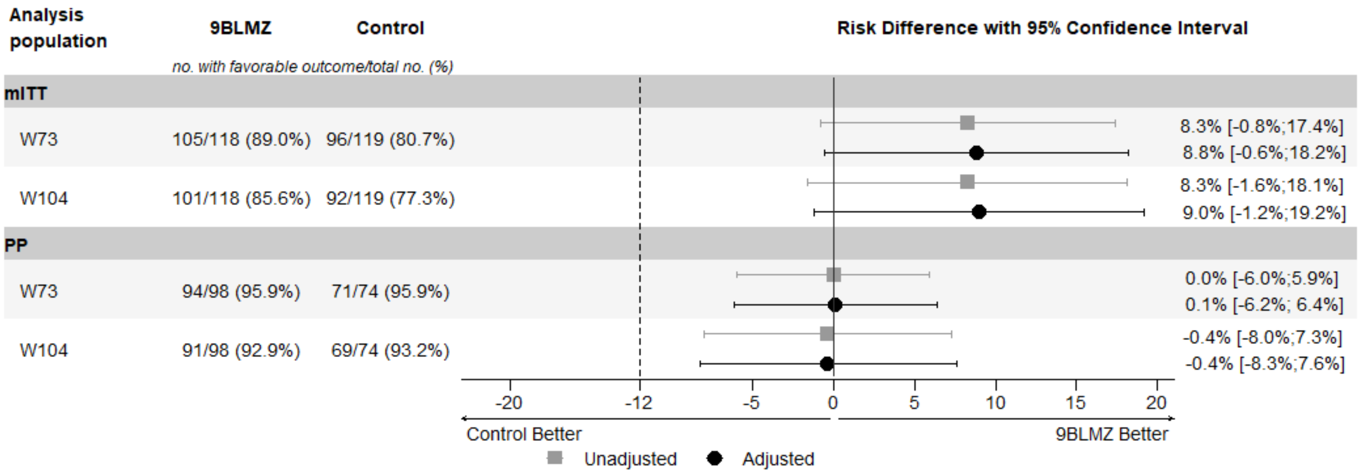

Note: Confidence intervals widths have not been adjusted for multiplicity and the intervals should not be used in place of hypothesis testing for secondary outcomes (Week 104). They are only presented for precision purposes.
Data-driven backwards selection among pre-specified covariates resulted in mITT (modified-intention-to-treat) model at weeks 73 and 104 adjusted for: hepatitis C, extent of disease; and PP (per-protocol) analyses at weeks 73 and 104 adjusted for: Age, BMI.

Figure S5b. Primary and Secondary efficacy analyses of endTB Trial Regimen 9BCLLfxZ vs Control at Week 73 and Week 104

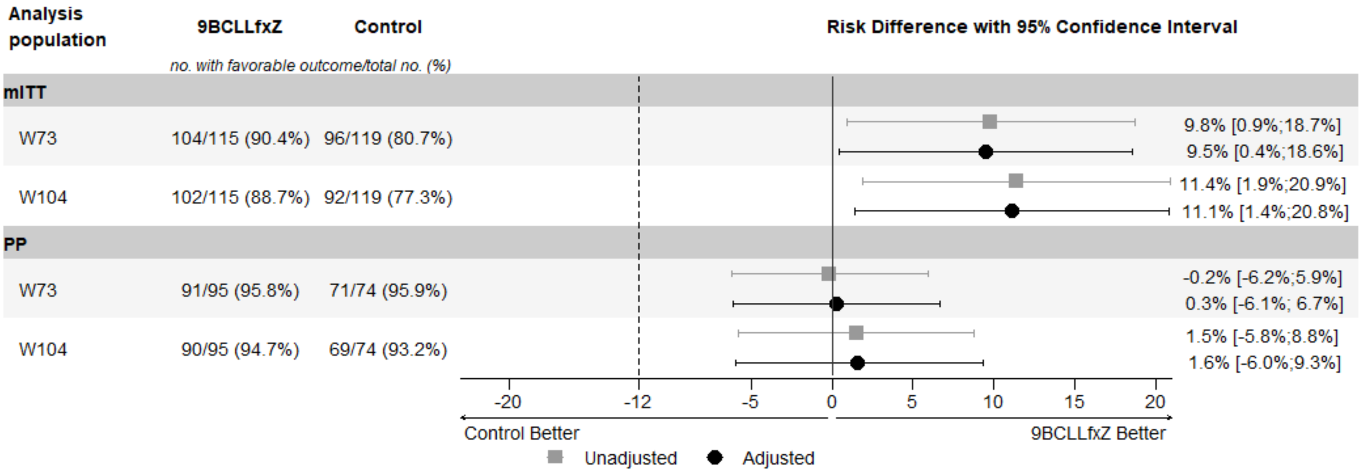

Note: Confidence intervals widths have not been adjusted for multiplicity and the intervals should not be used in place of hypothesis testing for secondary outcomes (Week 104). They are only presented for precision purposes.
Data-driven backwards selection among pre-specified covariates resulted in mITT (modified-intention-to-treat) model at weeks 73 and 104 adjusted for: hepatitis C, extent of disease; and PP (per-protocol) analyses at weeks 73 and 104 adjusted for: Age, BMI.

Figure S5c. Primary and Secondary efficacy analyses of endTB Trial Regimen 9BDLLfxZ vs Control at Week 73 and Week 104

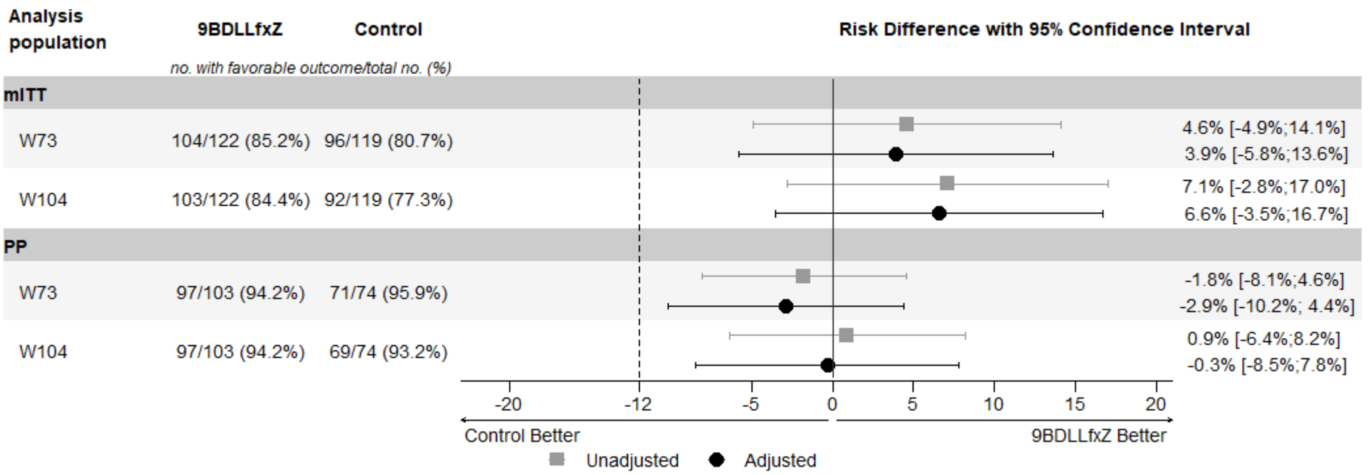

Note: Confidence intervals widths have not been adjusted for multiplicity and the intervals should not be used in place of hypothesis testing for secondary outcomes (Week 104). They are only presented for precision purposes.
Data-driven backwards selection among pre-specified covariates resulted in mITT (modified-intention-to-treat) model at weeks 73 and 104 adjusted for: hepatitis C, extent of disease; and PP (per-protocol) analyses at weeks 73 and 104 adjusted for: Age, BMI.

Figure S5d. Primary and Secondary efficacy analyses of endTB Trial Regimen 9DCLLfxZ vs Control at Week 73 and Week 104

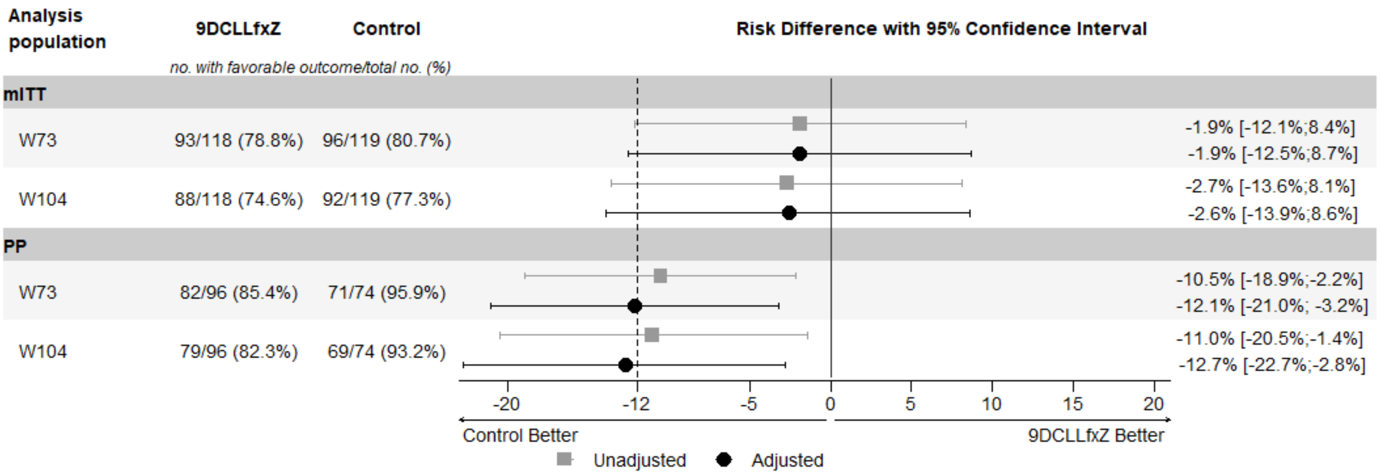

Note: Confidence intervals widths have not been adjusted for multiplicity and the intervals should not be used in place of hypothesis testing for secondary outcomes (Week 104). They are only presented for precision purposes.
Data-driven backwards selection among pre-specified covariates resulted in mITT (modified-intention-to-treat) model at weeks 73 and 104 adjusted for: hepatitis C, extent of disease; and PP (per-protocol) analyses at weeks 73 and 104 adjusted for: Age, BMI.

Figure S5e. Primary and Secondary efficacy analyses of endTB Trial Regimen 9DCMZ vs Control at Week 73 and Week 104

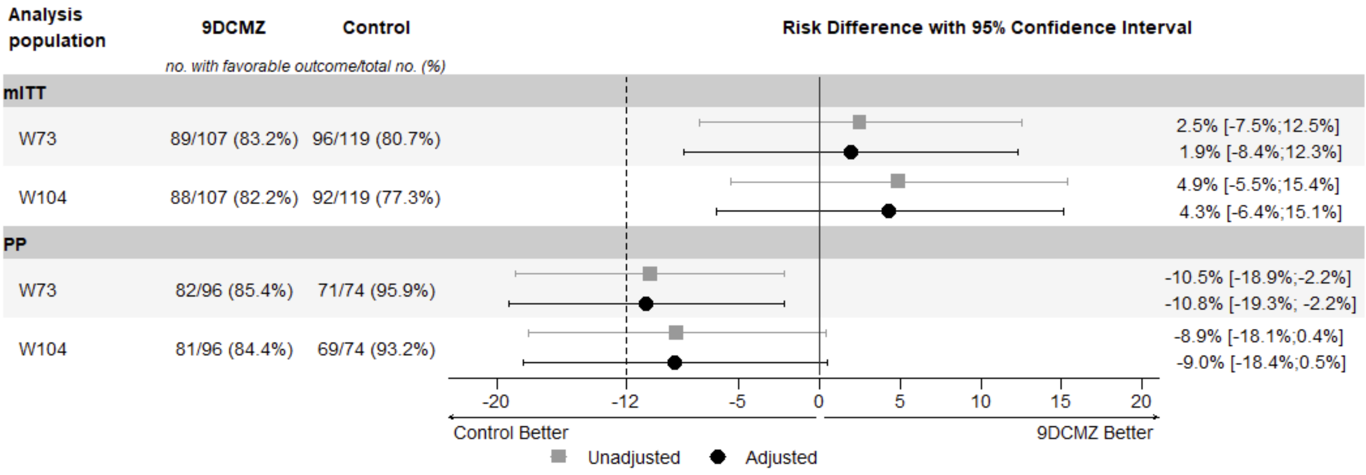

Note: Confidence intervals widths have not been adjusted for multiplicity and the intervals should not be used in place of hypothesis testing for secondary outcomes (Week 104). They are only presented for precision purposes.
Data-driven backwards selection among pre-specified covariates resulted in mITT (modified-intention-to-treat) model at weeks 73 and 104 adjusted for: hepatitis C, extent of disease; and PP (per-protocol) analyses at weeks 73 and 104 adjusted for: Age, BMI.

Figure S6a. Subgroup Analysis of Efficacy of endTB Trial Regimen 9BLMZ vs Control at Week 73 (mITT population)

	9BLMZ	Control	RD (95% CI)
**mITT**	**105/118 (89.0%)**	**96/119 (80.7%)**	**8.3% [-0.8%;17.4%]**
**Country**			
Georgia	1/2 (50.0%)	2/3 (66.7%)	-16.7% [-114.4%;81.1%]
India	7/8 (87.5%)	3/4 (75.0%)	12.5% [-37.9%;62.9%]
Kazakhstan	27/30 (90.0%)	19/23 (82.6%)	7.4% [-11.6%;26.4%]
Lesotho	14/14 (100.0%)	12/12 (100.0%)	–
Pakistan	17/18 (94.4%)	17/18 (94.4%)	0.0% [-15.2%;15.2%]
Peru	35/38 (92.1%)	36/51 (70.6%)	21.5% [6.3%;36.8%]
South Africa	4/8 (50.0%)	7/8 (87.5%)	-37.5% [-80.4%;5.4%]
**HIV**			
Negative	91/103 (88.3%)	79/100 (79.0%)	9.3% [-0.8%;19.5%]
Positive	14/15 (93.3%)	17/19 (89.5%)	3.9% [-15.1%;22.8%]
**Hepatitis C**			
Negative	102/113 (90.3%)	93/113 (82.3%)	8.0% [-1.0%;16.9%]
Positive	3/5 (60.0%)	3/6 (50.0%)	10.0% [-51.6%;71.6%]
**Diabetes**			
No	86/99 (86.9%)	86/104 (82.7%)	4.2% [-5.7%;14.1%]
Yes	19/19 (100.0%)	10/15 (66.7%)	33.3% [9.1%;57.5%]
**BMI**			
<18.5	36/41 (87.8%)	35/39 (89.7%)	-1.9% [-15.8%;12.0%]
18.5-24.9	57/65 (87.7%)	47/62 (75.8%)	11.9% [-1.5%;25.3%]
≥25	12/12 (100.0%)	14/18 (77.8%)	22.2% [2.7%;41.8%]
**Sex**			
Female	36/41 (87.8%)	42/48 (87.5%)	0.3% [-13.5%;14.1%]
Male	69/77 (89.6%)	54/71 (76.1%)	13.6% [1.5%;25.6%]
**Age**			
<18	1/2 (50.0%)	5/6 (83.3%)	-33.3% [-114.0%;47.3%]
18-44	84/93 (90.3%)	75/91 (82.4%)	7.9% [-2.0%;17.8%]
≥45	20/23 (87.0%)	16/22 (72.7%)	14.2% [-9.2%;37.6%]
**Pyrazinamide**			
Sensitive	57/61 (93.4%)	44/57 (77.2%)	16.2% [3.7%;28.8%]
Resistant	48/57 (84.2%)	49/59 (83.1%)	1.2% [-12.4%;14.7%]
**Smear results**			
Negative/scanty	16/20 (80.0%)	13/19 (68.4%)	11.6% [-16.1%;39.2%]
1-2+	54/57 (94.7%)	43/52 (82.7%)	12.0% [0.2%;23.9%]
3+	35/41 (85.4%)	40/48 (83.3%)	2.0% [-13.2%;17.2%]
**Cavitation**			
Absence	43/50 (86.0%)	31/43 (72.1%)	13.9% [-2.7%;30.5%]
Presence	62/68 (91.2%)	64/75 (85.3%)	5.8% [-4.7%;16.3%]
**Prior exposure TB treatment**			
None	68/76 (89.5%)	59/74 (79.7%)	9.7% [-1.8%;21.2%]
First line only	20/20 (100.0%)	24/31 (77.4%)	22.6% [7.7%;37.4%]
Second line	10/15 (66.7%)	10/11 (90.9%)	-24.2% [-54.1%;5.6%]
**WHO recommendations**			
<18/12/2018	24/30 (80.0%)	22/31 (71.0%)	9.0% [-12.6%;30.7%]
18/12/2018-31/03/2020	37/40 (92.5%)	32/40 (80.0%)	12.5% [-2.4%;27.4%]
≥ 01/04/2020	44/48 (91.7%)	42/48 (87.5%)	4.2% [-8.1%;16.4%]

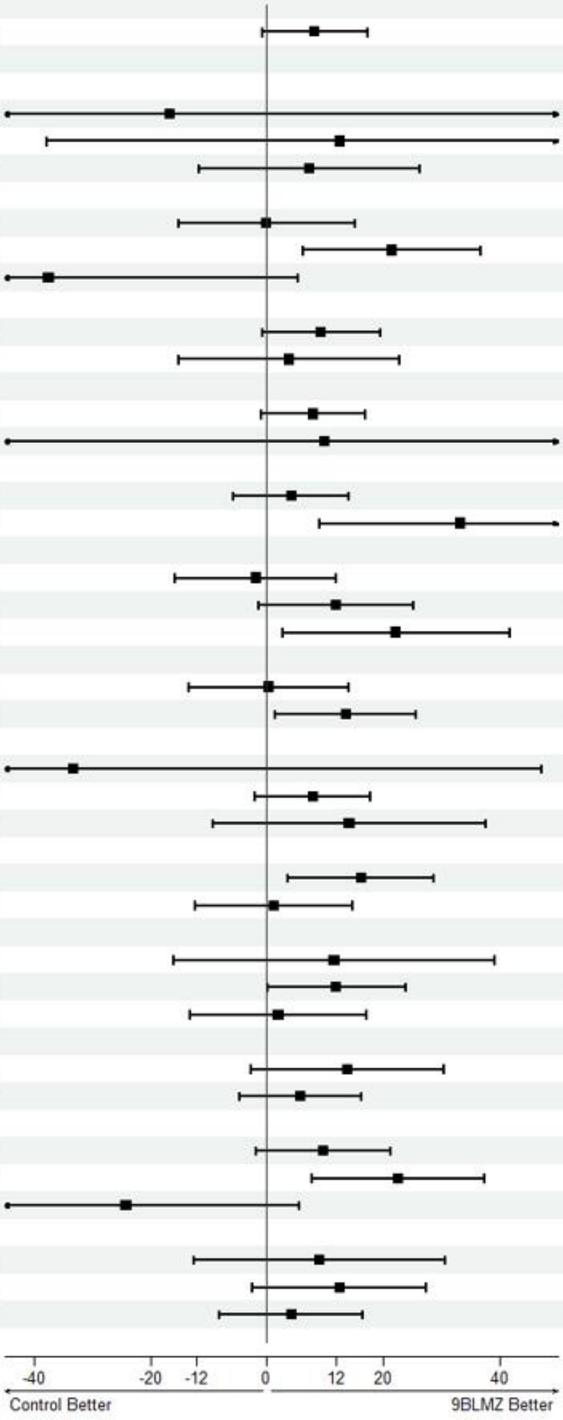

Note: confidence intervals widths have not been adjusted for multiplicity and the intervals should not be used in place of hypothesis testing. They are only presented for precision purposes.

Figure S6b. Subgroup Analysis of Efficacy of endTB Trial Regimen 9BCLLfxZ vs Control at Week 73 (mITT Population)

	9BCLLfxZ	Control	RD (95% CI)
**mITT**	**104/115 (90.4%)**	**96/119 (80.7%)**	**9.8% [0.9%;18.7%]**
**Country**			
Georgia	2/2 (100.0%)	2/3 (66.7%)	33.3% [-26.3%;93.0%]
India	4/4 (100.0%)	3/4 (75.0%)	25.0% [-20.4%;70.4%]
Kazakhstan	31/35 (88.6%)	19/23 (82.6%)	6.0% [-12.9%;24.9%]
Lesotho	11/11 (100.0%)	12/12 (100.0%)	
Pakistan	15/16 (93.8%)	17/18 (94.4%)	-0.7% [-16.8%;15.4%]
Peru	37/39 (94.9%)	36/51 (70.6%)	24.3% [9.9%;38.7%]
South Africa	4/8 (50.0%)	7/8 (87.5%)	-37.5% [-80.4%;5.4%]
**HIV**			
Negative	90/101 (89.1%)	79/100 (79.0%)	10.1% [0.1%;20.2%]
Positive	14/14 (100.0%)	17/19 (89.5%)	10.5% [-3.5%;24.5%]
**Hepatitis C**			
Negative	101/110 (91.8%)	93/113 (82.3%)	9.5% [0.8%;18.2%]
Positive	3/5 (60.0%)	3/6 (50.0%)	10.0% [-51.6%;71.6%]
**Diabetes**			
No	86/96 (89.6%)	86/104 (82.7%)	6.9% [-2.6%;16.4%]
Yes	18/19 (94.7%)	10/15 (66.7%)	28.1% [1.8%;54.3%]
**BMI**			
<18.5	27/33 (81.8%)	35/39 (89.7%)	-7.9% [-24.3%;8.4%]
18.5-24.9	57/61 (93.4%)	47/62 (75.8%)	17.6% [5.2%;30.0%]
≥25	20/21 (95.2%)	14/18 (77.8%)	17.5% [-4.1%;39.0%]
**Sex**			
Female	34/37 (91.9%)	42/48 (87.5%)	4.4% [-8.5%;17.3%]
Male	70/78 (89.7%)	54/71 (76.1%)	13.7% [1.7%;25.7%]
**Age**			
<18	3/3 (100.0%)	5/6 (83.3%)	16.7% [-15.0%;48.3%]
18-44	65/71 (91.5%)	75/91 (82.4%)	9.1% [-1.1%;19.3%]
≥45	36/41 (87.8%)	16/22 (72.7%)	15.1% [-6.2%;36.4%]
**Pyrazinamide**			
Sensitive	48/52 (92.3%)	44/57 (77.2%)	15.1% [2.0%;28.3%]
Resistant	56/63 (88.9%)	49/59 (83.1%)	5.8% [-6.5%;18.2%]
**Smear results**			
Negative/scanty	18/19 (94.7%)	13/19 (68.4%)	26.3% [2.8%;49.8%]
1-2+	55/59 (93.2%)	43/52 (82.7%)	10.5% [-1.6%;22.7%]
3+	31/37 (83.8%)	40/48 (83.3%)	0.5% [-15.5%;16.4%]
**Cavitation**			
Absence	43/46 (93.5%)	31/43 (72.1%)	21.4% [6.1%;36.7%]
Presence	61/69 (88.4%)	64/75 (85.3%)	3.1% [-8.0%;14.1%]
**Prior exposure TB treatment**			
None	62/67 (92.5%)	59/74 (79.7%)	12.8% [1.7%;24.0%]
First line only	20/23 (87.0%)	24/31 (77.4%)	9.5% [-10.8%;29.9%]
Second line	18/19 (94.7%)	10/11 (90.9%)	3.8% [-16.2%;23.9%]
**WHO recommendations**			
<18/12/2018	24/28 (85.7%)	22/31 (71.0%)	14.7% [-6.0%;35.5%]
18/12/2018-31/03/2020	35/37 (94.6%)	32/40 (80.0%)	14.6% [0.0%;29.1%]
≥ 01/04/2020	45/50 (90.0%)	42/48 (87.5%)	2.5% [-10.1%;15.1%]

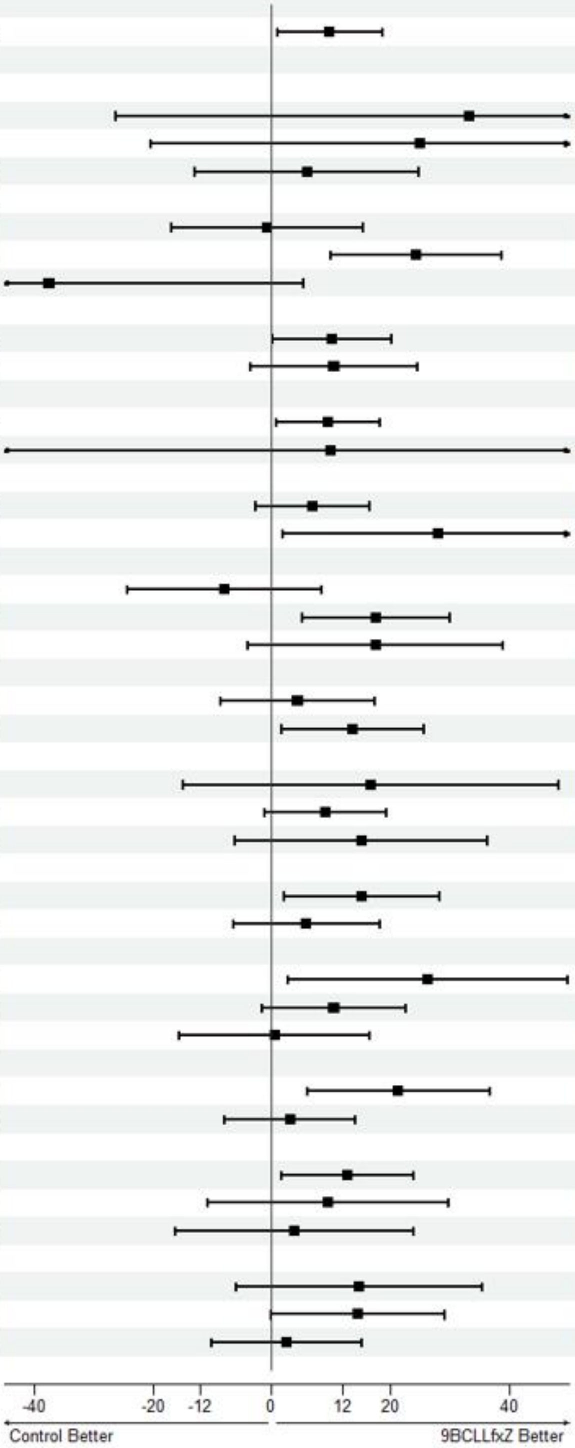

Note: confidence intervals widths have not been adjusted for multiplicity and the intervals should not be used in place of hypothesis testing. They are only presented for precision purposes.

Figure S6c. Subgroup Analysis of Efficacy of endTB Trial Regimen 9BDLLfxZ vs Control at Week 73 (mITT Population

	9BDLLfxZ	Control	RD (95% CI)
**mITT**	**104/122 (85.2%)**	**96/119 (80.7%)**	**4.6% [-4.9%;14.1%]**
**Country**			
Georgia	1/1 (100.0%)	2/3 (66.7%)	33.3% [-28.3%;94.9%]
India	2/3 (66.7%)	3/4 (75.0%)	-8.3% [-82.0%;65.3%]
Kazakhstan	29/33 (87.9%)	19/23 (82.6%)	5.3% [-14.0%;24.5%]
Lesotho	11/15 (73.3%)	12/12 (100.0%)	-26.7% [-49.5%;-3.9%]
Pakistan	10/13 (76.9%)	17/18 (94.4%)	-17.5% [-43.2%;8.1%]
Peru	44/49 (89.8%)	36/51 (70.6%)	19.2% [4.0%;34.4%]
South Africa	7/8 (87.5%)	7/8 (87.5%)	0.0% [-33.5%;33.5%]
**HIV**			
Negative	92/105 (87.6%)	79/100 (79.0%)	8.6% [-1.6%;18.8%]
Positive	12/17 (70.6%)	17/19 (89.5%)	-18.9% [-44.9%;7.2%]
**Hepatitis C**			
Negative	102/119 (85.7%)	93/113 (82.3%)	3.4% [-6.0%;12.9%]
Positive	2/3 (66.7%)	3/6 (50.0%)	16.7% [-54.1%;87.4%]
**Diabetes**			
No	87/101 (86.1%)	86/104 (82.7%)	3.4% [-6.5%;13.4%]
Yes	16/20 (80.0%)	10/15 (66.7%)	13.3% [-16.7%;43.4%]
**BMI**			
<18.5	20/28 (71.4%)	35/39 (89.7%)	-18.3% [-37.7%;1.1%]
18.5-24.9	72/81 (88.9%)	47/62 (75.8%)	13.1% [0.4%;25.8%]
≥25	12/13 (92.3%)	14/18 (77.8%)	14.5% [-9.9%;39.0%]
**Sex**			
Female	46/55 (83.6%)	42/48 (87.5%)	-3.9% [-17.5%;9.7%]
Male	58/67 (86.6%)	54/71 (76.1%)	10.5% [-2.4%;23.4%]
**Age**			
<18	5/5 (100.0%)	5/6 (83.3%)	16.7% [-14.6%;47.9%]
18-44	71/86 (82.6%)	75/91 (82.4%)	0.1% [-11.1%;11.4%]
≥45	28/31 (90.3%)	16/22 (72.7%)	17.6% [-3.9%;39.1%]
**Pyrazinamide**			
Sensitive	50/56 (89.3%)	44/57 (77.2%)	12.1% [-1.5%;25.7%]
Resistant	54/66 (81.8%)	49/59 (83.1%)	-1.2% [-14.6%;12.2%]
**Smear results**			
Negative/scanty	28/31 (90.3%)	13/19 (68.4%)	21.9% [-1.7%;45.5%]
1-2+	47/58 (81.0%)	43/52 (82.7%)	-1.7% [-16.1%;12.8%]
3+	29/33 (87.9%)	40/48 (83.3%)	4.5% [-10.9%;20.0%]
**Cavitation**			
Absence	38/48 (79.2%)	31/43 (72.1%)	7.1% [-10.7%;24.8%]
Presence	65/73 (89.0%)	64/75 (85.3%)	3.7% [-7.1%;14.5%]
**Prior exposure TB treatment**			
None	70/78 (89.7%)	59/74 (79.7%)	10.0% [-1.4%;21.4%]
First line only	22/27 (81.5%)	24/31 (77.4%)	4.1% [-16.9%;25.0%]
Second line	11/15 (73.3%)	10/11 (90.9%)	-17.6% [-46.2%;11.1%]
**WHO recommendations**			
<18/12/2018	25/30 (83.3%)	22/31 (71.0%)	12.4% [-8.6%;33.4%]
18/12/2018-31/03/2020	37/42 (88.1%)	32/40 (80.0%)	8.1% [-7.8%;24.0%]
≥ 01/04/2020	42/50 (84.0%)	42/48 (87.5%)	-3.5% [-17.4%;10.4%]

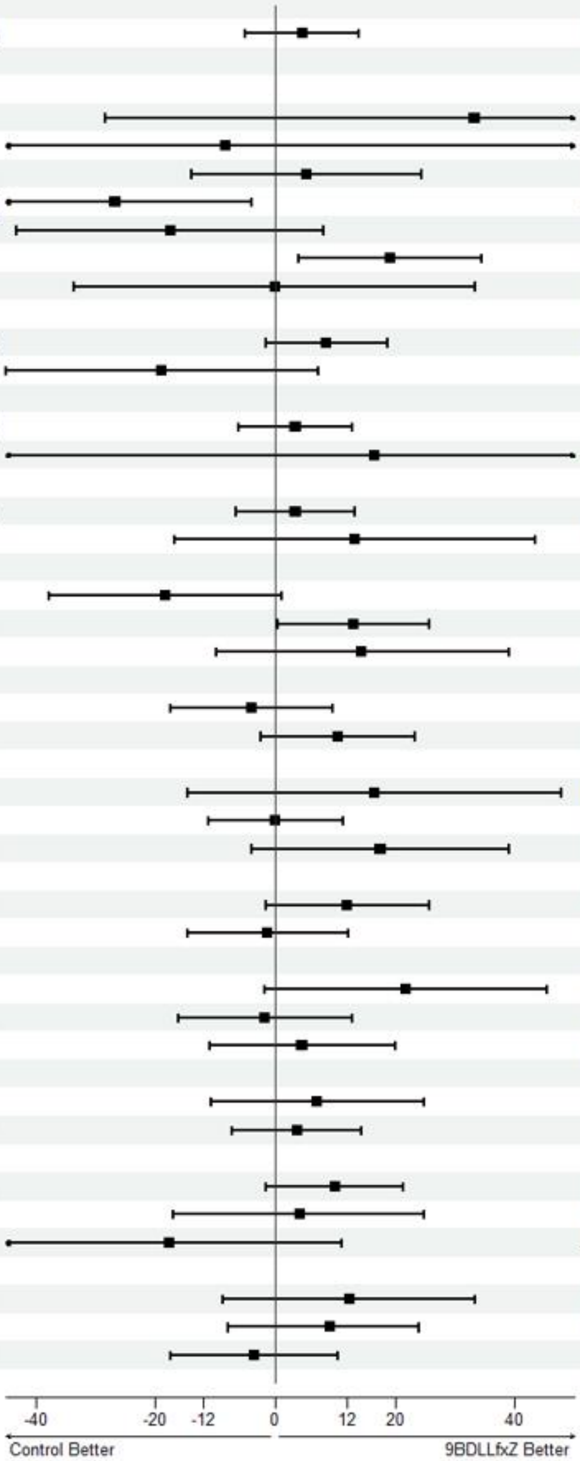

Note: confidence intervals widths have not been adjusted for multiplicity and the intervals should not be used in place of hypothesis testing. They are only presented for precision purposes.

Figure S6d. Subgroup Analysis of Efficacy of endTB Trial Regimen 9DCLLfxZ vs Control at Week 73 (mITT Population)

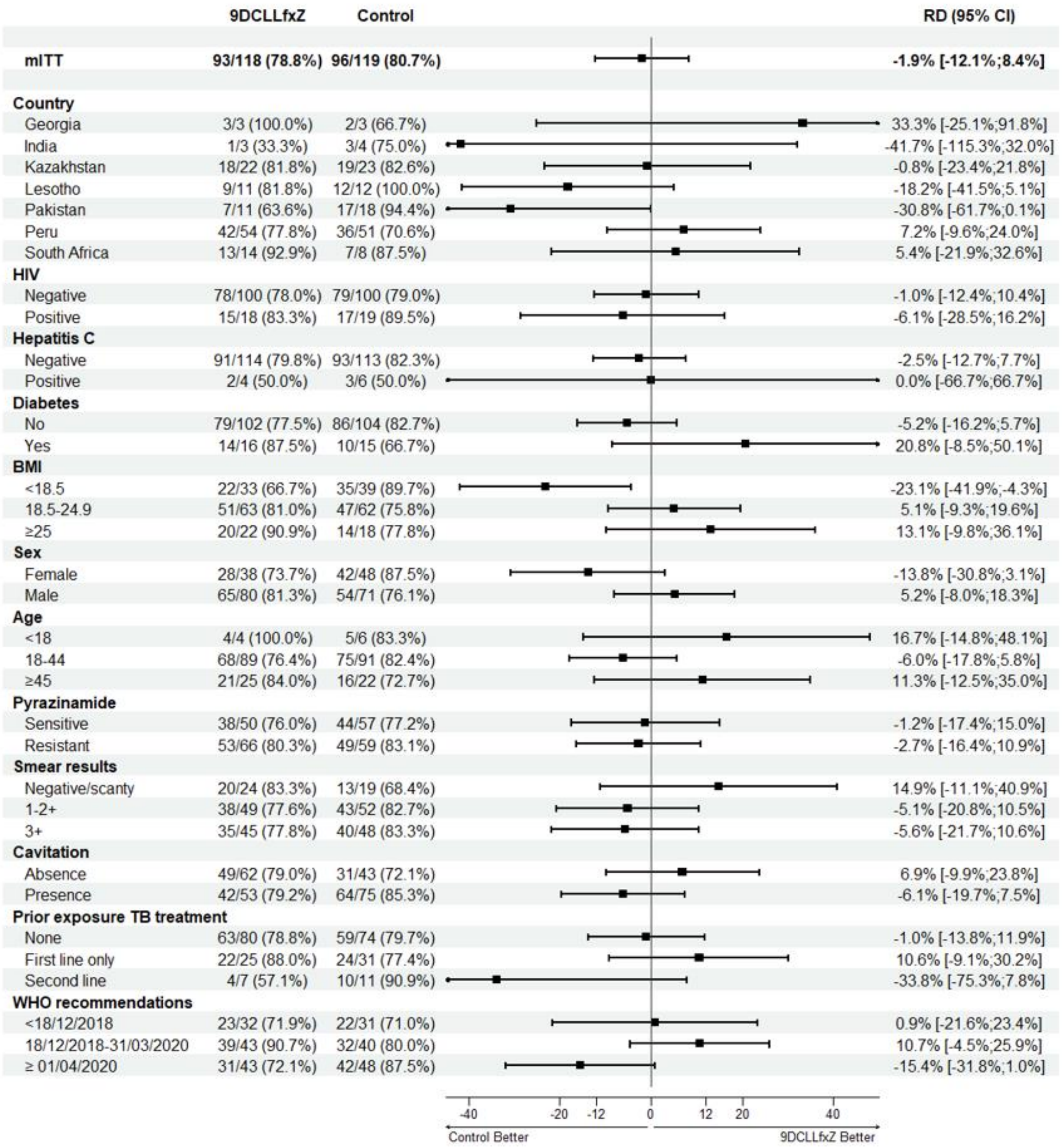

Note: confidence intervals widths have not been adjusted for multiplicity and the intervals should not be used in place of hypothesis testing. They are only presented for precision purposes.

Figure S6e. Subgroup Analysis of Efficacy of endTB Trial Regimen 9DCMZ vs Control at Week 73 (mITT Population)

	9DCMZ	Control	RD (95% CI)
**mITT**	**89/107 (83.2%)**	**96/119 (80.7%)**	**2.5% [−7.5%;12.5%]**
**Country**			
Georgia	1/1 (100.0%)	2/3 (66.7%)	33.3% [−28.3%;94.9%]
India	1/1 (100.0%)	3/4 (75.0%)	25.0% [−22.4%;72.4%]
Kazakhstan	20/24 (83.3%)	19/23 (82.6%)	0.7% [−21.0%;22.5%]
Lesotho	13/14 (92.9%)	12/12 (100.0%)	−7.1% [−20.9%;6.6%]
Pakistan	14/16 (87.5%)	17/18 (94.4%)	−6.9% [−26.6%;12.7%]
Peru	36/45 (80.0%)	36/51 (70.6%)	9.4% [−7.8%;26.6%]
South Africa	4/6 (66.7%)	7/8 (87.5%)	−20.8% [−66.6%;25.0%]
**HIV**			
Negative	78/92 (84.8%)	79/100 (79.0%)	5.8% [−5.1%;16.7%]
Positive	11/15 (73.3%)	17/19 (89.5%)	−16.1% [−42.8%;10.5%]
**Hepatitis C**			
Negative	87/104 (83.7%)	93/113 (82.3%)	1.4% [−8.7%;11.4%]
Positive	2/3 (66.7%)	3/6 (50.0%)	16.7% [−54.1%;87.4%]
**Diabetes**			
No	77/91 (84.6%)	86/104 (82.7%)	1.9% [−8.5%;12.3%]
Yes	12/16 (75.0%)	10/15 (66.7%)	8.3% [−24.1%;40.8%]
**BMI**			
<18.5	29/36 (80.6%)	35/39 (89.7%)	−9.2% [−25.4%;7.0%]
18.5−24.9	47/57 (82.5%)	47/62 (75.8%)	6.6% [−7.9%;21.2%]
=25	13/14 (92.9%)	14/18 (77.8%)	15.1% [−8.8%;38.9%]
**Sex**			
Female	42/45 (93.3%)	42/48 (87.5%)	5.8% [−6.1%;17.8%]
Male	47/62 (75.8%)	54/71 (76.1%)	−0.2% [−14.9%;14.4%]
**Age**			
<18	5/5 (100.0%)	5/6 (83.3%)	16.7% [−14.6%;47.9%]
18−44	62/73 (84.9%)	75/91 (82.4%)	2.5% [−8.9%;13.9%]
=45	22/29 (75.9%)	16/22 (72.7%)	3.1% [−21.4%;27.6%]
**Pyrazinamide**			
Sensitive	36/41 (87.8%)	44/57 (77.2%)	10.6% [−4.3%;25.5%]
Resistant	53/66 (80.3%)	49/59 (83.1%)	−2.7% [−16.4%;10.9%]
**Smear results**			
Negative/scanty	16/18 (88.9%)	13/19 (68.4%)	20.5% [−5.3%;46.3%]
1−2+	38/43 (88.4%)	43/52 (82.7%)	5.7% [−8.4%;19.8%]
3+	35/46 (76.1%)	40/48 (83.3%)	−7.2% [−23.6%;9.1%]
**Cavitation**			
Absence	42/46 (91.3%)	31/43 (72.1%)	19.2% [3.4%;35.0%]
Presence	47/61 (77.0%)	64/75 (85.3%)	−8.3% [−21.6%;5.0%]
**Prior exposure TB treatment**			
None	60/72 (83.3%)	59/74 (79.7%)	3.6% [−9.0%;16.2%]
First line only	18/23 (78.3%)	24/31 (77.4%)	0.8% [−21.7%;23.4%]
Second line	11/11 (100.0%)	10/11 (90.9%)	9.1% [−8.3%;26.5%]
**WHO recommendations**			
<18/12/2018	25/33 (75.8%)	22/31 (71.0%)	4.8% [−17.0%;26.6%]
18/12/2018−31/03/2020	33/40 (82.5%)	32/40 (80.0%)	2.5% [−14.7%;19.7%]
= 01/04/2020	31/34 (91.2%)	42/48 (87.5%)	3.7% [−9.8%;17.1%]

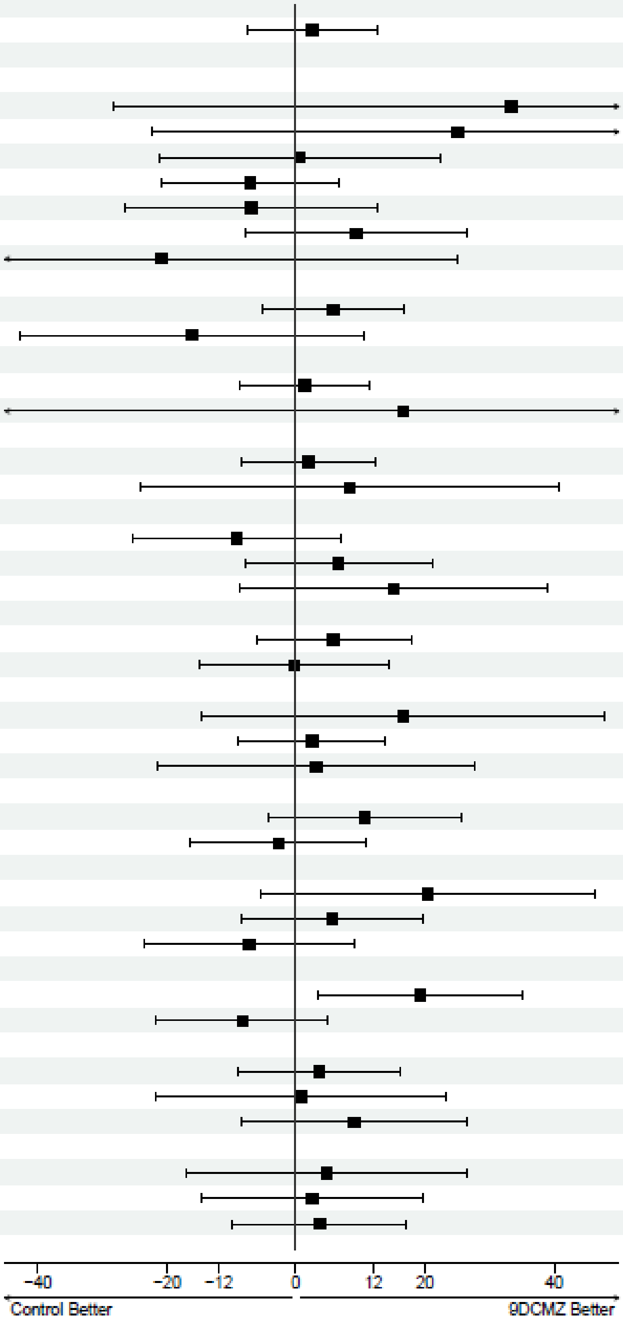

Figure S7a. Kaplan Meier Curve of Time to Unfavorable Outcome by Week 73: endTB Experimental Regimen 9BLMZ vs. Control (mITT population)

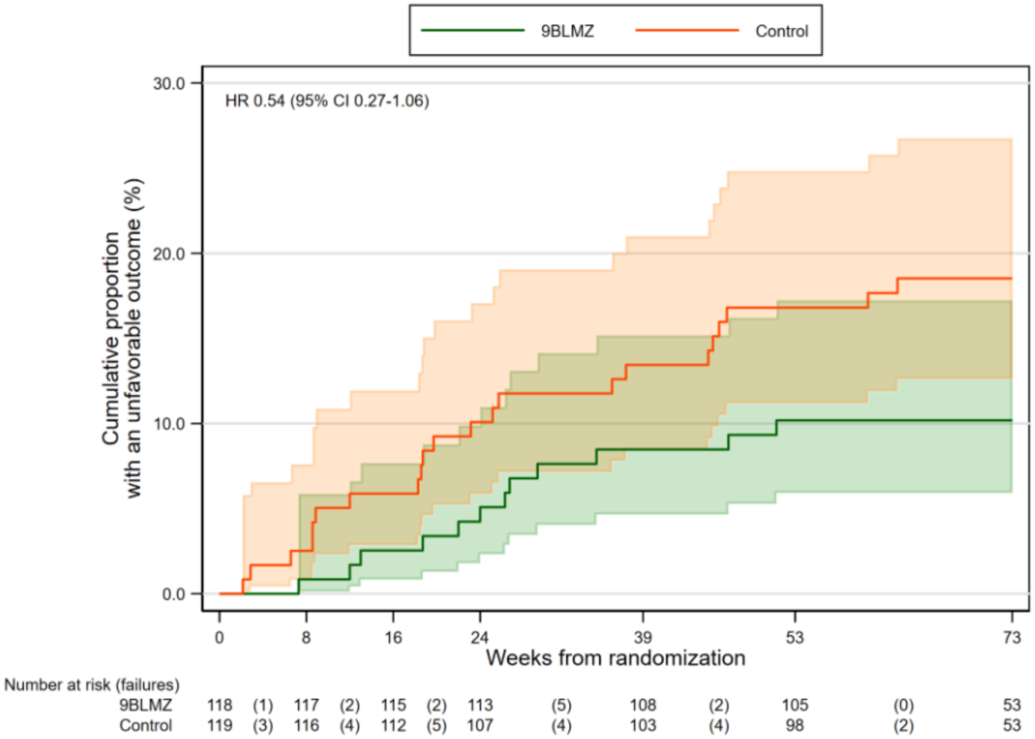

Shaded areas are 95% CI around estimates; Control: orange, Experimental: light green, Control and Experimental: overlapped color. Confidence intervals widths have not been adjusted for multiplicity and the intervals should not be used in place of hypothesis testing for secondary outcomes. They are only presented for precision purposes.

Figure S7b. Kaplan Meier Curve of Time to Unfavorable Outcome by Week 73: endTB Experimental Regimen 9BCLLfxZ vs. Control (mITT population)

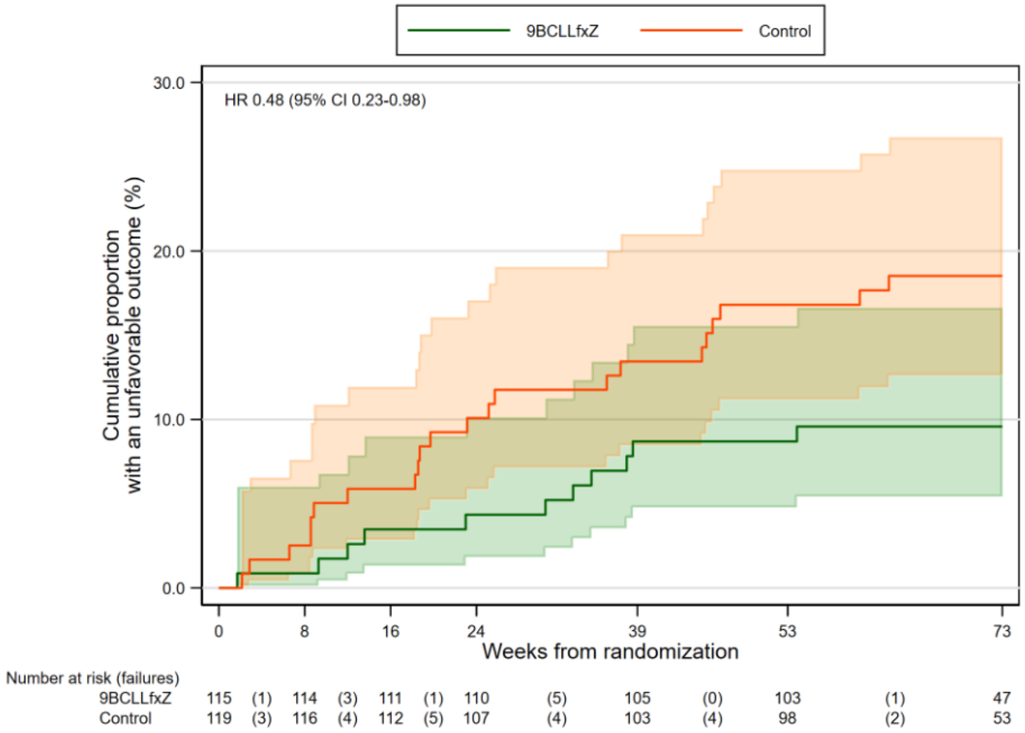

Shaded areas are 95% CI around estimates; Control: orange, Experimental: light green, Control and Experimental: overlapped color. Confidence intervals widths have not been adjusted for multiplicity and the intervals should not be used in place of hypothesis testing for secondary outcomes. They are only presented for precision purposes.

Figure S7c. Kaplan Meier Curve of Time to Unfavorable Outcome by Week 73: endTB Experimental Regimen 9BDLLfxZ vs. Control (mITT population)

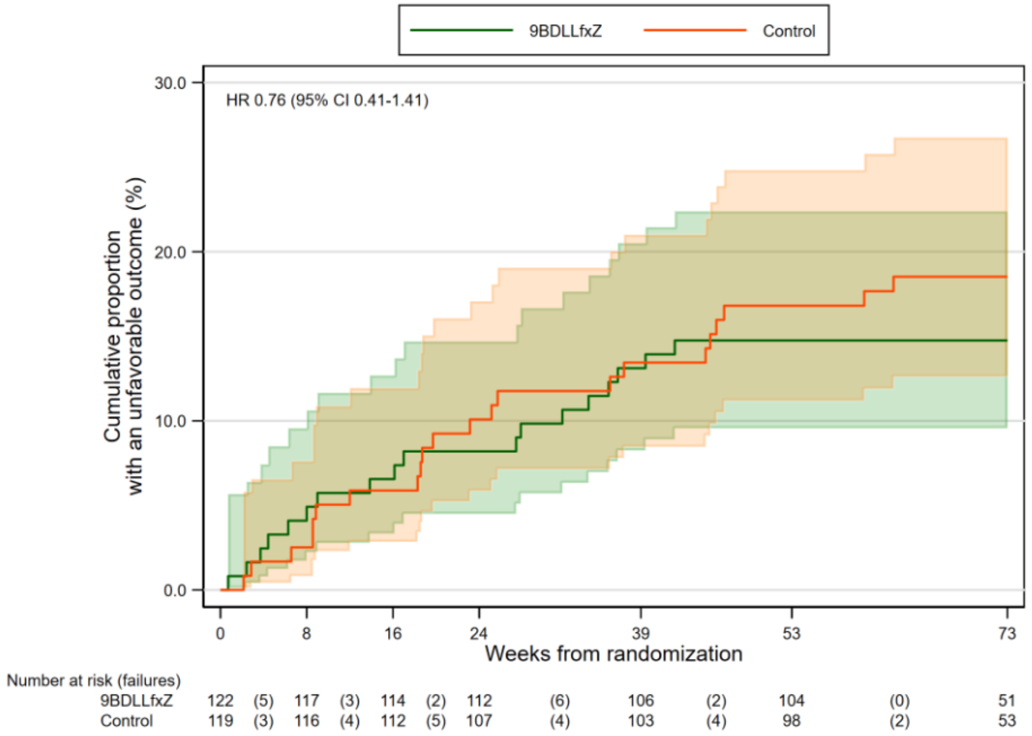

Shaded areas are 95% CI around estimates; Control: orange, Experimental: light green, Control and Experimental: overlapped color. Confidence intervals widths have not been adjusted for multiplicity and the intervals should not be used in place of hypothesis testing for secondary outcomes. They are only presented for precision purposes.

Figure S7d. Kaplan Meier Curve of Time to Unfavorable Outcome by Week 73: endTB Experimental Regimen 9DCLLfxZ vs. Control (mITT population)

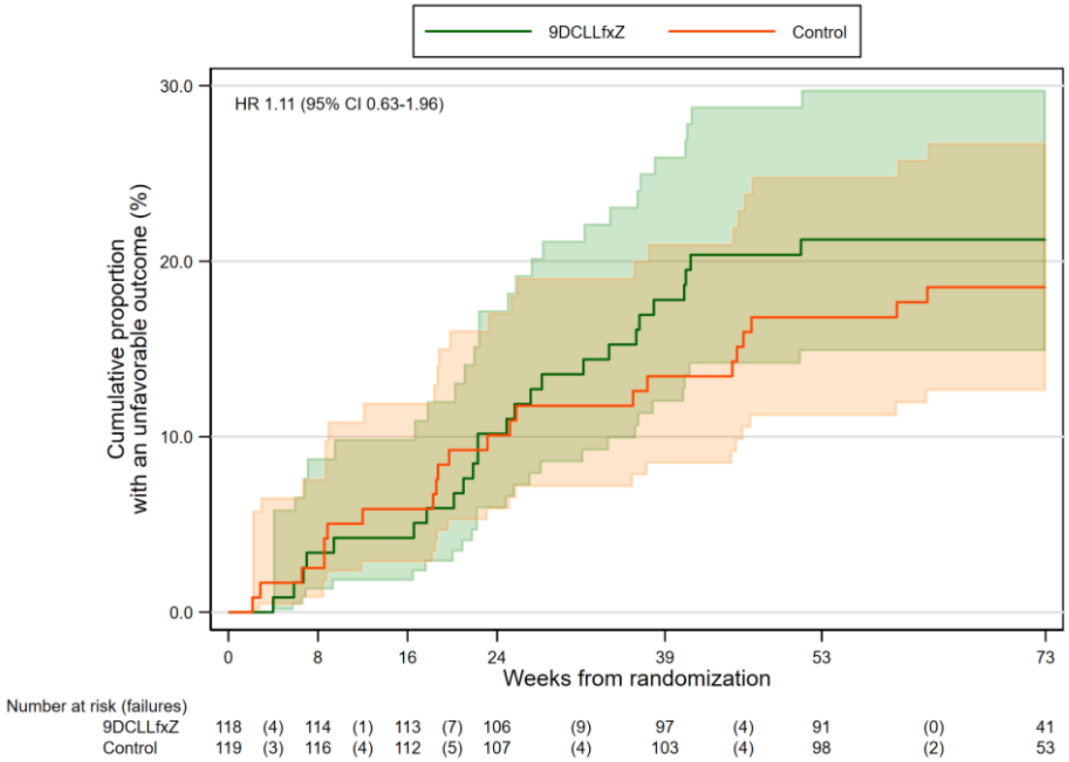

Shaded areas are 95% CI around estimates; Control: orange, Experimental: light green, Control and Experimental: overlapped color. Confidence intervals widths have not been adjusted for multiplicity and the intervals should not be used in place of hypothesis testing for secondary outcomes. They are only presented for precision purposes.

Figure S7e. Kaplan Meier Curve of Time to Unfavorable Outcome by Week 73: endTB Experimental Regimen 9DCMZ vs. Control (mITT population)

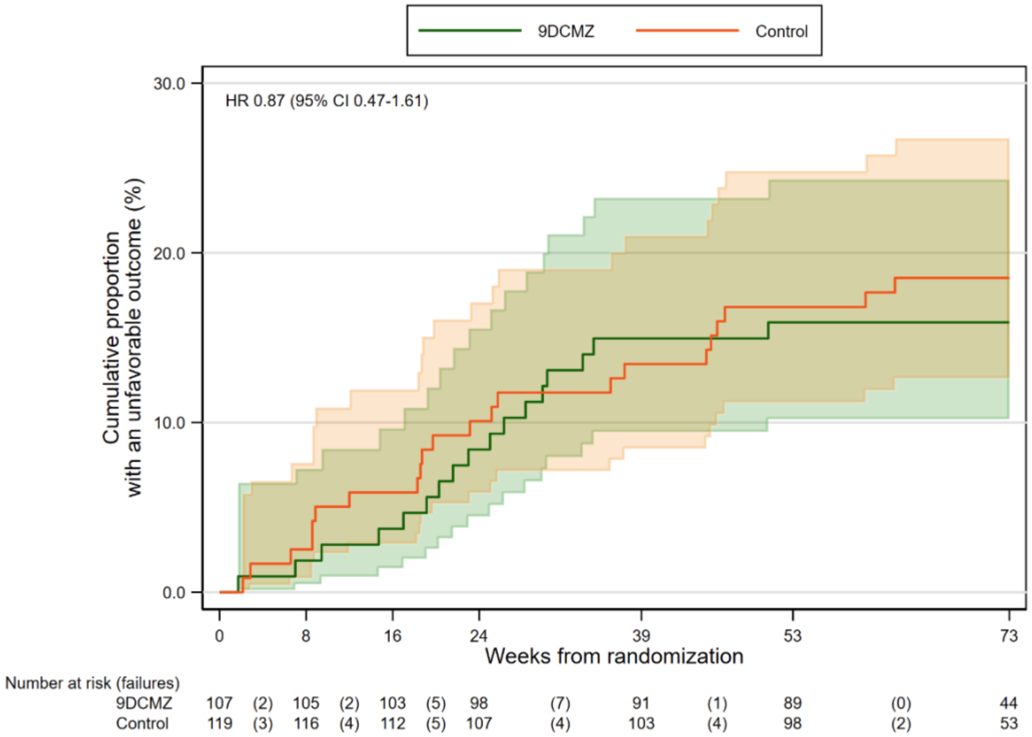

Shaded areas are 95% CI around estimates; Control: orange; Experimental: light green; Control and Experimental: overlapped color. Confidence intervals widths have not been adjusted for multiplicity and the intervals should not be used in place of hypothesis testing for secondary outcomes. They are only presented for precision purposes.

Table S1. endTB Trial Protocol Writing Committee

Name	Role	Institution
Elisabeth Baudin	Trial Statistician	Epicentre, France
Elmira Berikova	Site representative Kazakhstan	National Scientific Center of Phthisiopulmonology, Kazakhstan
Maryline Bonnet	TB Epidemiologist	Translational Research on HIV and Endemic and Emerging Infectious Diseases, France
Bouke de Jong	Mycobacteriologist	Institute of Tropical Medicine, Belgium
Gabriella Ferlazzo	MDR-TB Clinician	Médecins Sans Frontières, France
Lorenzo Guglielmetti	MDR-TB Clinician	Médecins Sans Frontières, France
Uzma Khan	MDR-TB Clinician	Interactive Research and Development, Singapore
Nana Kiria	Site representative Georgia	National Center for Tuberculosis and Lung Diseases, Georgia
Nathalie Lachenal	PV expert	Médecins Sans Frontières, Switzerland
Leonid Lecca	Site representative Peru	Socios En Salud Sucursal Perú, Peru
Helen McIlleron	Pharmacologist	University of Cape Town, South Africa
Carole Mitnick	TB Epidemiologist	Harvard Medical School, USA
Lawrence Oyewusi	Site representative Lesotho	Partners In Health, Lesotho
Samiran Panda	Site representative India	Indian Council of Medical Research-National AIDS Research Center, India
Patrick Phillips	TB Statistician	University of California San Francisco, USA
Michael Rich	MDR-TB Clinician	Partners In Health, USA
Nassem Salahuddin	Site representative Pakistan	The Indus Hospital and Health Network, Pakistan
Kwonjune Seung	MDR-TB Clinician	Partners In Health, USA
Oleksandr Telnov	MDR-TB Clinician	Hôpitaux Universitaires de Genève, Switzerland
Lorenzo Trippa	Bayesian Statistician	Dana-Farber Cancer Institute, USA
Francis Varaine	MDR-TB Expert	Médecins Sans Frontières, France
Gustavo Velásquez	MDR-TB Clinician	University of California San Francisco, USA
Sean Wasserman	Site representative South Africa	Institute for Infection and Immunity at St. George's, United Kingdom
Peter Zimetbaum	Electrophysiologist	Beth Israel Deaconess Medical Center, USA

Table S2. endTB Implementing Partners by Country

Country	Implementing Partners
Georgia	National Center for Tuberculosis and Lung Diseases
India	Doctors Without Borders India<br>Aundh Chest Hospital
Kazakhstan	Partners In Health Kazakhstan<br>National Scientific Center of Phthisiopulmonology<br>National Center for Tuberculosis Problems<br>City Centre of Phthisiopulmonology
Lesotho	Partners In Health Lesotho<br>Botsabelo MDR-TB Hospital
Pakistan	Interactive Research and Development<br>The Indus Hospital & Health Network<br>Institute of Chest Disease
Peru	Socios En Salud (Partners In Health Peru)<br>Hospital Nacional Hipólito Unanue<br>Hospital Nacional Sergio Bernales<br>Hospital Nacional Dos de Mayo
South Africa	Médecins Sans Frontières Belgium<br>Town 2 Community Day Centre

Table S3. endTB Data Safety and Monitoring Board

Name	Institution
D. Stephen Coad	Queen Mary University of London, United Kingdom
Kelly Dooley	Vanderbilt University, United States
Mathilde Fréchet-Jachym	Sanatorium, Centre Hospitalier de Bligny, France
Michael Hoelscher	Ludwig Maximilians Universitat, Germany
Kevin Monahan	Boston University, United States

Table S4. endTB Scientific Advisory Committee

Name	Institution
Kathleen Eisenach	TB or NOT TB Consulting, United States
Robert Horsburgh	Boston University, United States
Christoph Lange	Research Center Borstel, Germany
Christian Lienhardt	National Institutes of Health, United States
Graeme Meintjes	University of Cape Town, South Africa
Eric Nuermberger	Johns Hopkins University School of Medicine, United States
Andrew Nunn	University College London, United Kingdom

Table S5. Global TB Community Advisory Board

Name	Institution	Country
Albert S. Makone	Shiloah Zimbabwe	Zimbabwe
Ani Herna Sari	Perhimpunan Organisasi Pasien TB (POP-TB) and Perkumpulan Rekat Surabaya	Indonesia
Diptendu Bhattacharya	Survivors Against TB	India
Gloria Kerubo Moses	Amref Health Africa	Kenya
Jimmy Galarza Castillo	University of Texas Health [formerly Socios En Salud, Peru]	USA
Jonathan Stillo	TB Europe Coalition; Eurasian Community for Access to Treatment; Wayne State University	Romania; Moldova; USA
Kseniia Schenina	TB People, TB Europe Coalition	Russia
Ketholelie Angami	Access to Rights and Knowledge Foundation; Nagaland Users' Network	India
Lindsay McKenna	Treatment Action Group	USA
Oxana Rucsineanu	Society of Moldova against Tuberculosis (SMIT); TB Patients Association in Moldova	Moldova
Paran Sarmita Winarni	Pejuang Tangguh; Youth Movement Against TB Indonesia	Indonesia
Patrick Agbassi	VillageReach	Ivory Coast
Rosa Herrera	Americas TB Coalition; Instituto de Servicios de Salud Publica del Estado de Baja California	Mexico
Sergiy Kondratyuk	International Treatment Preparedness Coalition	Ukraine
Wim Vandevelde	Global Network for and by People Living with HIV; European AIDS Treatment Group; AfroCAB	South Africa; Belgium

Table S6. Representativeness of Study Participants

Characteristic	Description Globally	Description endTB
Disease, problem, or condition under investigation	Distribution of characteristics in general population of people with notified TB (7.5 million) or MDR/RR-TB in 2022 (1)	Distribution of characteristics in endTB mITT study population (N=699)<br>N (%) unless otherwise specified
Sex	Among global notified TB cases: 55% male (2)	435 (62.2%) male
Age	From MDR/RR IPD-MA:<br>Median (IQR) age: 34 (24-45)<br><br>From WHO TB notification tables (2022):<br>92% >15+ years<br>4.4% >15-19 years<br>47% 20-44 years<br>41% >=45 years	Median (IQR) age: 32 (23,44)<br><br><br><br>25(3.6%) 15-17 years<br>503 (72.0%) 18-45 years<br>171 (24.5%) >45 years
Race or ethnic group	To our knowledge, there are no well-established biological links between race or ethnic group and the risk of developing MDR/RR-TB.	There is significant racial and ethnic diversity, linked to the geographic diversity described below.
Geography	Of the 30 countries with the highest burden of TB, 17 are in Africa (including Lesotho, S. Africa), 1 is in S. America, 2 are in S. Asia (India, Pakistan).<br><br>Of the 30 countries with the highest burden of MDR/RR-TB: 1 is in S. America (Peru), 9 are in E. Europe/Central Asia (including Kazakhstan), and 8 are in Africa (including S. Africa). MDR incidence is increasing in South-East Asia and the Americas.	Trial participants were from Africa (Lesotho, South Africa: 18.5%), S. Asia (India, Pakistan: 16.5%), E. Europe/Central Asia (Georgia, Kazakhstan: 25.6%), and South America (Peru: 39.5%).
Considerations related to HIV status	6.3% of TB patients are coinfected with HIV globally, >50% in parts of southern Africa.<br><br>In the 2021 individual patient meta-analysis of MDR-TB treatment outcomes used to inform WHO 2022 Guidelines, 14% of included individuals were people living with HIV. (3)	14.0% HIV positive (57.7% South Africa, 71.4% Lesotho); <5% other countries

Table S7. Weight-based dosing and frequency of administration of endTB trial study drugs (experimental arms)

**Drug***	**Weight Band (kg)**					
	**24-30**	**>30-35**	**>35-45**	**>45-55**	**>55-70**	**>70**
**Bedaquiline (B)**	200 mg QD for 2 weeks** followed by 100 mg 3x/week	400 mg QD x 2 weeks*** followed by 200 mg 3x/week				
**Delamanid (D)**	50 mg BID	100 mg BID				
**Moxifloxacin (M)**	400 mg					
**Levofloxacin (Lfx)**	750 mg		1000 mg			
**Linezolid (L)********	300 mg QD until Week 16 (followed by 300 mg QD or 600 mg 3x/week)	600 mg QD until Week 16 (followed by 300 mg QD or 600 mg 3x/week)				
**Clofazimine (C)**	100 mg					
**Pyrazinamide (Z)**	800 mg		1200 mg	1600 mg		2000 mg

***** Dosing was once a day unless otherwise indicated.
****** The intensive phase of bedaquiline treatment for the 24-30 kg weight band comprised 2 weeks of 200 mg QD. If the participant was already receiving bedaquiline treatment, bedaquiline within the experimental regimen was to be dosed as a continuation of that treatment (i.e., only remaining doses of the 2-week intensive phase were to be administered and the intensive phase was not to be restarted if it had already been completed at time of study treatment initiation).
*** The intensive phase of bedaquiline treatment for participants weighing more than 30 kg comprised 2 weeks of 400 mg QD. If the participant was already receiving bedaquiline treatment, bedaquiline within the experimental regimen was to be dosed as a continuation of that treatment (i.e., only remaining doses of the 2-week intensive phase were to be administered and the intensive phase was not to be restarted if it had already been completed at time of study treatment initiation).
**** Linezolid dosing was routinely modified at Week 16 or sooner if necessary to reduce toxicity related to linezolid. The modification entailed either decreased (300 mg daily) or intermittent (600 mg 3x/week) dosing.

Table S8. Weight-based dosing and frequency of administration of endTB trial study drugs (control arm)

<table>
<tr><th rowspan="2">Drug*</th><th colspan="6">Weight Band (kg)</th></tr>
<tr><th>24-30</th><th>>30-35</th><th>>35-45</th><th>>45-55</th><th>>55-70</th><th>>70</th></tr>
<tr><td>Levofloxacin (Lfx)</td><td>500 mg</td><td colspan="2">750 mg</td><td colspan="3">1000 mg</td></tr>
<tr><td>Moxifloxacin (M)</td><td colspan="6">400 mg</td></tr>
<tr><td>Bedaquiline (B)</td><td>200 mg daily for 2 weeks, followed by 100 mg 3 times/week</td><td colspan="5">400 mg daily for 2 weeks, followed by 200 mg 3 times/week</td></tr>
<tr><td>Linezolid (L)</td><td>300 mg</td><td colspan="5">600 mg</td></tr>
<tr><td>Clofazimine (C)</td><td colspan="6">100mg</td></tr>
<tr><td>Cycloserine (Cs)/ Terizidone (Tzd)</td><td colspan="3">500 mg</td><td colspan="3">750 mg</td></tr>
<tr><td>Ethambutol (E)</td><td>400 or 600 mg</td><td colspan="2">800 mg</td><td colspan="3">1200 mg</td></tr>
<tr><td>Delamanid (D)</td><td>50 mg BID</td><td colspan="5">100 mg BID</td></tr>
<tr><td>Pyrazinamide (Z)</td><td>1000 mg</td><td>1200 mg</td><td colspan="3">1600 mg</td><td>2000 mg</td></tr>
<tr><td>Amikacin (Am)</td><td>500 mg</td><td>625 mg</td><td>750 mg</td><td>750 or 1000 mg</td><td colspan="2">1000 mg</td></tr>
<tr><td>Ethionamide (Eto)/ Prothionamide (Pto)</td><td colspan="3">500 mg</td><td colspan="2">750 mg</td><td>1000 mg</td></tr>
<tr><td>Para-aminosalicylic acid (PAS)</td><td>3 to 3.5 g BID</td><td colspan="4">4 g BID</td><td>4 or 6 g BID</td></tr>
<tr><td>Isoniazid (H)</td><td>150 mg</td><td>200 mg</td><td colspan="4">300 mg</td></tr>
<tr><td>High-dose isoniazid (H^H)</td><td>400 mg</td><td colspan="2">450 mg</td><td colspan="3">600 mg</td></tr>
</table>

* Dosing was once a day unless otherwise indicated.

Table S9. endTB trial Schedule of Events

		Treatment																				Follow-Up												
	Screening	Baseline	W1	W2	W3	W4	W5	W6	W7	W8	W9	W 10	W 11	W 12	W 16	W 20	W 24	W 28	W 32	W 36	W39	W43	W47	W53	W59	W65	W73	W81	W89	W97	W 104	Sub total (minimal count)	Early Termination	Post-termination follow-up
Visit	1	2	3	4	5	6	7	8	9	10	11	12	13	14	15	16	17	18	19	20	21	22	23	24	25	26	27	28	29	30	31			
Window Period			+/- 2	+/- 2	+/- 2	+/- 2	+/- 2	+/- 2	+/- 2	+/- 2[#]	+/- 2	+/- 2	+/- 2	+/- 2	+/-7	+/- 7	+/- 7	+/- 7	+/- 7	+/- 7	+/- 14	+/- 14	+/- 14	+/- 14	+/- 14	+/- 14	+/- 30	+/- 14	+/- 14	+/- 14	+/- 30			
Eligibility																																		
Subject Consent	X	X																														2		
Demographics	X	X																														2		
Medical History	X																															1		
Inclusion/ Exclusion	X	X																														2		
Clinical Evaluation																																		
Vital Signs	X	X	X	X	X	X	X	X	X	X	X	X	X	X	X	X	X	X	X	X	X	X	X	X	X	X	X	X	X	X	X	31	X	X
Interval Medical History		X	X	X	X	X	X	X	X	X	X	X	X	X	X	X	X	X	X	X	X	X	X	X	X	X	X	X	X	X	X	30	X	X
Physical Exam	X	X	X	X	X	X	X	X	X	X	X	X	X	X	X	X	X	X	X	X	X	X	X	X	X	X	X	X	X	X	X	31	X	X
TB Symptom Assessment		X	X	X	X	X	X	X	X	X	X	X	X	X	X	X	X	X	X	X	X	X	X	X	X	X	X	X	X	X	X	30	X	X
ECOG Assessment		X																			X						X				X	4	X	X
Neurologic Exam		X				X				X				X	X	X	X	X	X	X	X	X	X				X				X	15	X	
Ophthalmologic Exam		X				X				X				X	X	X	X	X	X	X	X	X	X				X				X	15	X	
Mental Health Assessment		X																									X				X	3	X	
Treatment																																		
Randomization		X*													X**																	(1)		
Treatment Compliance			X	X	X	X	X	X	X	X	X	X	X	X	X	X	X	X	X	X	X	X[C]	X[C]	X[C]	X[C]	X[C]	X[C]	X[C]	X[C]	X[C]	X[C]	19	X[C]	
Adherence Counseling		X	X	X	X	X	X	X	X	X	X	X	X	X	X	X	X	X	X	X	X	X[C]	X[C]	X[C]	X[C]	X[C]	X[C]	X[C]	X[C]	X[C]		20		
Concomitant Medication Review	X	X	X	X	X	X	X	X	X	X	X	X	X	X	X	X	X	X	X	X	X	X	X	X	X	X	X	X	X	X	X	31	X	X
AE Review			X	X	X	X	X	X	X	X	X	X	X	X	X	X	X	X	X	X	X	X	X	X	X	X	X	X	X	X	X	29	X	X
Laboratory Testing																																		
CBC	X[1]					X				X				X	X	X	X	X	X	X	X	X	X								X	14	X	
Total Ca++, K+, Mg++	X[1]					X				X				X	X	X	X	X	X	X	X	X	X									13	X	

	Screening	Baseline	W1	W2	W3	W4	W5	W6	W7	W8	W 9	W 10	W 11	W 12	W 16	W 20	W 24	W 28	W 32	W 36	W39	W 43	W 47	W 53	W 59	W 65	W 73	W 81	W 89	W 97	W 104	Sub total (minimal count)	Early Termination	Post-termination follow-up
Visit	1	2	3	4	5	6	7	8	9	10	11	12	13	14	15	16	17	18	19	20	21	22	23	24	25	26	27	28	29	30	31			
Window Period			+/- 2	+/- 2	+/- 2	+/- 2	+/- 2	+/- 2	+/- 2	+/- 2[#]	+/- 2	+/- 2	+/- 2	+/- 2	+/- 7	+/- 7	+/- 7	+/- 7	+/- 7	+/- 7	+/- 14	+/- 14	+/- 14	+/- 14	+/- 14	+/- 14	+/- 30	+/- 14	+/- 14	+/- 14	+/- 30			
Creatinine	X[1]					X				X				X	X	X	X	X	X	X	X	X	X									13	X	
AST and ALT[2]	X[1]					X				X				X	X	X	X	X	X	X	X	X	X									13	X	
Total and Direct Bilirubin	X[1]					X				X				X	X	X	X	X	X	X	X	X	X									13	X	
Albumin	X[1]																															1		
TSH	X[3]	X[8]															X						X									3	X	
HbA1c	X[4]	X[8]															X[5]						X[5]				X[5]					(1)	X[5, 17]	X[5, 17]
HIV test	X[6]																															1		
CD4 count and viral load (in HIV+)	X[7]	X[8]															X[9]						X[9]				X[9]					(0)	X[9,17]	X[9,17]
Hepatitis B serology (anti-HBc total and HbsAg)	X	X[8]																														1		
Hepatitis C Serology: HCVAb	X	X[8]																														1		
Pregnancy test[11]	X	X[10]																														(2)	X	
Sputum Specimen Testing																																		
Smear Microscopy	X			X		X				X				X	X	X	X	X	X	X	X	X	X	X	X	X	X	X	X	X	X	22	X	X
Culture (LJ & MGIT)	X			X		X				X				X	X	X	X	X	X	X	X	X	X	X	X	X	X	X	X	X	X	22	X	X
Rapid Molecular Test for RIF Resistance	X[12]	X[8]																														1		
Rapid molecular test for FQ resistance	X[12]	X[8]													X[13]																	(1)		
DST (conventional 1st and 2nd line)	X														X[13,14]																	(1)	X[17]	X[17]
DST (new drugs)	X[14]														X[14]																	(0)	X[17]	X[17]
Genotyping[14]	X														X																	(0)	X[17]	X[17]
Specimen Storage	X[15]														X[15]																	(1)	X	
Special Assays or Procedures																																		
Audiometry		X				X				X				X	X	X	X	X	X	X	X	X	X				X				X	15	X	
ECG[1]	X[1]	X	X	X	X	X	X	X	X	X	X	X	X	X	X	X	X	X	X	X	X	X	X	X			X					25	X	
Chest X-ray		X[16]								X											X						X				X	5	X[17]	X[17]

Legend: endTB Trial Schedule of Events

#: W8 visit window was to occur within 8 weeks (+/-2 days) of study treatment initiation.

*: Randomization for treatment regimen assignment.

**: Only for participants receiving linezolid, randomization to linezolid dose reduction strategy could occur at W16 or earlier (concomitant to dose reduction decision).

C: Only for participants still on prescription.

1. Test with abnormal result at screening visit could be repeated within screening window.
2. ALT/AST tests were to be symptom-driven after Week 47.
3. Optionally, documented results of TSH (performed less than 4 weeks prior to screening visit date) could substitute for screening/baseline test. Investigators were encouraged to repeat TSH if they deemed it useful for patient management or to have a reliable pre-treatment result.
4. Optionally, documented results of HbA1c (performed less than 3 months prior to screening visit date) could substitute for screening/baseline test. Investigators were encouraged to repeat HbA1c if they deemed it useful for patient management or to have a reliable pre-treatment result.
5. HbA1c test (or 2 fasting blood glucose tests if HbA1c could not be done) to be repeated every 6 months only if abnormal at screening/baseline.
6. HIV serology was to be offered, unless patient was known to be positive or a documented negative result was available from less than one month before screening visit date.
7. CD4 count and HIV viral load were performed if the patient was known or found to be HIV positive. Optionally, documented results of CD4 count (performed less than six months prior to screening visit date) and viral load (performed less than 4 weeks prior to screening visit date) could substitute for screening test. Investigators were encouraged to repeat CD4 count/viral load if they deemed it useful for patient management or to have a reliable pre-treatment result.
8. Completed only if not done at screening.
9. CD4 count and HIV viral load were monitored every 6 months in HIV-positive patients.
10. Serum pregnancy test at baseline visit could be repeated if the blood specimen had not been drawn within 72 hours prior to treatment start.
11. Pregnancy tests could be performed on urine or serum samples after baseline visit until the end of study treatment.
12. Rapid molecular test results for RIF and FQ resistance, performed at designated study lab not more than 3 weeks prior to screening visit date had to be available before randomization.
13. Done locally on first positive culture or any culture reversion at or after Week 16.
14. Done at ITM on first positive culture or any culture reversion at or after Week 16 and on corresponding screening/baseline strain.
15. Stored the screening/baseline sample and isolate from the corresponding culture. When possible, stored samples and isolates from subsequent positive cultures for study (and future research) purposes.
16. Was not necessary if previous adequate imaging results within 3 weeks prior to baseline visit date were available.
17. Could be indicated, based on availability of previous results.

Table S10. Other Baseline Characteristics of mITT Population

Baseline characteristics	9BLMZ (n = 118)	9BCLLfxZ (n = 115)	9BDLLfxZ (n = 122)	9DCLLfxZ (n = 118)	9DCMZ (n = 107)	Control (n = 119)	Total (N = 699)
**Baseline QTcF* (ms)**							
Median [IQR]	400.5 [384.0;415.0]	399.5 [387.5;416.5]	398.0 [382.5;420.0]	401.3 [380.0;417.0]	399.0 [383.0;415.5]	397.5 [376.5;417.0]	399.5 [383.0;417.5]
Min-Max	340.0 - 449.0	340.0 - 448.0	344.0 - 448.0	335.5 - 448.0	340.0 - 444.5	348.0 - 443.0	335.5 - 449.0
**Sensory neuropathy**							
Normal	88 (74.6%)	77 (67.0%)	101 (82.8%)	97 (82.2%)	75 (70.1%)	93 (78.2%)	531 (76.0%)
Grade 1	17 (14.4%)	21 (18.3%)	14 (11.5%)	6 (5.1%)	9 (8.4%)	15 (12.6%)	82 (11.7%)
Grade 2	6 (5.1%)	15 (13.0%)	3 (2.5%)	12 (10.2%)	17 (15.9%)	4 (3.4%)	57 (8.1%)
Grade 3	7 (5.9%)	2 (1.7%)	4 (3.3%)	3 (2.5%)	6 (5.6%)	7 (5.9%)	29 (4.2%)
**Visual acuity**							
Normal	69 (58.5%)	51 (44.4%)	54 (44.3%)	68 (57.6%)	57 (53.3%)	62 (52.5%)	361 (51.7%)
Grade 1	23 (19.5%)	28 (24.4%)	42 (34.4%)	24 (20.3%)	24 (22.4%)	32 (27.1%)	173 (24.8%)
Grade 2	2 (1.7%)	7 (6.1%)	6 (4.9%)	4 (3.4%)	3 (2.8%)	6 (5.1%)	28 (4.0%)
Grade 3	22 (18.6%)	26 (22.6%)	18 (14.8%)	19 (16.1%)	18 (16.8%)	17 (14.4%)	120 (17.2%)
Grade 4	1 (0.9%)	3 (2.6%)	2 (1.6%)	3 (2.5%)	5 (4.7%)	1 (0.9%)	15 (2.1%)
**Complete blood cell count**							
**WBC count (10^9/L)**							
Median [IQR]	8.2 [6.5;10.9]	8.7 [7.2;11.3]	8.6 [6.7;11.0]	8.3 [6.4;10.0]	8.2 [6.2;10.4]	8.6 [7.0;10.6]	8.5 [6.7;10.8]
Min-Max	3.1 - 23.5	2.9 - 18.5	3.0 - 17.8	3.5 - 17.3	2.5 - 19.9	2.1 - 16.6	2.1 - 23.5
**Neutrophils (10^9/L)**							
Median [IQR]	5.8 [4.5;7.7]	6.2 [5.0;8.5]	6.1 [4.4;8.0]	5.5 [4.0;7.2]	5.7 [4.1;7.5]	6.0 [4.6;7.5]	5.8 [4.5;7.9]
Min-Max	1.8 - 20.7	1.1 - 15.9	1.2 - 15.6	1.8 - 14.5	0.9 - 16.5	0.9 - 14.1	0.9 - 20.7
**Lymphocytes (10^9/L)**							
Median [IQR]	1.6 [1.3;2.1]	1.6 [1.3;2.1]	1.7 [1.3;2.1]	1.6 [1.3;2.2]	1.6 [1.3;2.1]	1.6 [1.2;2.1]	1.6 [1.3;2.1]
Min-Max	0.3 - 12.0	0.4 - 4.7	0.5 - 3.9	0.3 - 3.9	0.3 - 5.4	0.5 - 6.2	0.3 - 12.0
**Eosinophils (%)**							
Median [IQR]	0.2 [0.1;0.2]	0.2 [0.1;0.3]	0.2 [0.1;0.3]	0.2 [0.1;0.3]	0.1 [0.1;0.2]	0.1 [0.1;0.3]	0.2 [0.1;0.3]
Min-Max	0.0 - 3.0	0.0 - 1.5	0.0 - 4.0	0.0 - 0.9	0.0 - 1.3	0.0 - 2.8	0.0 - 4.0
**Hemoglobin (g/dL)**							
Median [IQR]	12.2 [10.6;13.9]	12.7 [11.0;14.1]	12.3 [11.0;13.4]	12.6 [11.2;14.3]	12.5 [11.0;13.6]	12.4 [10.7;13.7]	12.5 [10.9;13.8]
Min-Max	8.1 - 16.2	8.2 - 17.1	8.1 - 17.9	8.3 - 17.1	8.6 - 16.3	8.1 - 16.6	8.1 - 17.9
**Hematocrit (L/L)**							
Median [IQR]	37.6 [33.8;41.7]	37.4 [34.6;41.1]	37.0 [34.0;40.8]	38.1 [34.6;42.0]	37.6 [34.0;40.0]	37.8 [33.8;41.2]	37.6 [34.0;41.0]
Min-Max	25.1 - 49.5	26.0 - 48.0	25.0 - 56.4	25.7 - 50.8	26.7 - 53.1	23.7 - 50.0	23.7 - 56.4

Baseline characteristics	9BLMZ (n = 118)	9BCLLfxZ (n = 115)	9BDLLfxZ (n = 122)	9DCLLfxZ (n = 118)	9DCMZ (n = 107)	Control (n = 119)	Total (N = 699)
**Platelet count (10^9/L)**							
Median [IQR]	409.0 [295.0;538.0]	387.0 [299.0;474.0]	406.0 [304.0;480.0]	359.0 [279.0;443.0]	377.0 [284.0;487.0]	369.0 [299.0;483.0]	383.0 [295.0;480.0]
Min-Max	146.0 - 854.0	80.0 - 810.0	121.0 - 729.0	140.0 - 747.0	131.0 - 1166.0	30.0 - 856.0	30.0 - 1166.0
**RBC (10^{12}/L)**							
Median [IQR]	4.6 [4.1;4.9]	4.5 [4.2;4.8]	4.5 [4.1;4.9]	4.5 [4.1;4.9]	4.4 [4.1;4.8]	4.5 [4.1;4.9]	4.5 [4.1;4.9]
Min-Max	2.8 - 5.8	2.8 - 6.0	3.0 - 6.4	2.8 - 6.0	2.8 - 6.5	2.3 - 5.9	2.3 - 6.5
**Biochemistry**							
**ALT (U/L)**							
Median [IQR]	19.0 [12.0;36.9]	18.0 [11.0;27.0]	18.0 [12.0; 27.0]	22.0 [13.0;29.0]	16.5 [11.0;25.0]	18.0 [11.0;28.0]	19.0 [12.0;29.0]
Min-Max	2.4-81.0	2.9-115.0	2.6-103.0	3.5-103.0	1.5-104.0	5.0-123.0	1.5-123.0
**AST (U/L)**							
Median [IQR]	22.0 [15.3;31.0]	20.0 [15.2;28.9]	22.0 [15.0;29.2]	21.9 [17.0;30.6]	21.8 [14.0;29.0]	21.0 [15.0;30.0]	21.4 [15.0;30.0]
Min-Max	5.0-96.0	4.0-85.0	3.0-96.4	2.0-125.0	2.0-111.0	2.0-79.3	2.0-125.0
**Bilirubin (mg/dL)**							
Median [IQR]	0.40 [0.23;0.53]	0.40 [0.29;0.60]	0.30 [0.20;0.48]	0.38 [0.23;0.60]	0.35 [0.28;0.60]	0.38 [0.29;0.60]	0.38 [0.24;0.60]
Min-Max	0.14-1.40	0.00-1.60	0.08-1.18	0.15-2.09	0.13-1.40	0.11-1.10	0.0-2.10

*median of the highest QT intervals corrected according to the Fridericia formula

Table S11. Baseline phenotypic DST results – mITT population (N=699)

Drug resistance	endTB1 (9BLMZ) N resistant/N with result (%)	endTB2 (9BCLLfxZ) N resistant/N with result (%)	endTB3 (9BDLLfxZ) N resistant/N with result (%)	endTB4 (9DCLLfxZ) N resistant/N with result (%)	endTB5 (9DCMZ) N resistant/N with result (%)	endTB6 (Control) N resistant/N with result (%)	Total N resistant/N with result (%)
Total mITT	118	115	122	118	107	119	699
Rifampicin	102/118 (86.4)	104/115 (90.4)	110/122 (90.2)	103/116 (88.8)	95/107 (88.8)	105/116 (90.5)	619/694 (89.2)
Isoniazid	93/118 (78.8)	107/115 (93.0)	109/122 (89.3)	102/116 (87.9)	92/107 (86.0)	99/116 (85.3)	602/694 (86.7)
Streptomycin	57/78 (73.1)	64/80 (80.0)	60/85 (70.6)	54/79 (68.3)	49/71 (69.0)	61/81 (75.3)	345/474 (72.8)
Pyrazinamide	57/118 (48.3)	63/115 (54.8)	66/122 (54.1)	66/116 (56.9)	66/107 (61.7)	59/116 (50.9)	377/694 (54.3)
Ethambutol	50/96 (52.1)	47/96 (49.0)	49/99 (49.5)	47/91 (51.6)	48/87 (55.2)	43/96 (44.8)	284/565 (50.3)
PAS	6/40 (15.0)	13/41 (31.7)	7/50 (14.0)	13/57 (22.8)	9/46 (19.6)	17/54 (31.5)	65/288 (22.6)
Ethionamide	19/56 (33.9)	10/55 (18.2)	12/62 (19.3)	12/66 (18.2)	14/61 (23.0)	9/66 (13.6)	76/366 (20.8)
Injectable (any)	14/118 (11.9)	18/115 (15.6)	15/122 (12.3)	13/116 (11.2))	14/107 (13.1)	16/116 (13.8)	90/694 (13.0)
Kanamycin	12/100 (12.0)	12/99 (12.1)	10/109 (9.2)	9/105 (8.6)	11/91 (12.1)	10/101 (9.9)	64/605 (10.6)
Capreomycin	6/100 (6.0)	10/98 (10.2)	8/109 (7.3)	9/105 (8.6)	5/91 (5.5)	12/101 (11.9)	50/604 (8.3)
Amikacin	2/54 (3.7)	4/53 (7.6)	3/58 (5.2)	0/48 (0.0)	3/42 (7.1)	2/52 (3.8)	14/307 (4.6)
Fluoroquinolone (any)#	0/118 (0.0)	0/115 (0.0)	0/122 (0.0)	0/116 (0.0)	0/107 (0.0)	0/116 (0.0)	0/694 (0.0)
Moxifloxacin 0.25µg/mL	0/107 (0.0)	0/101 (0.0)	0/110 (0.0)	0/98 (0.0)	0/90 (0.0)	0/101 (0.0)	0/607 (0.0)
Moxifloxacin 1µg/mL	0/90 (0.0)	0/85 (0.0)	0/95 (0.0)	0/86 (0.0)	0/79 (0.0)	0/88 (0.0)	0/523 (0.0)
Levofloxacin 1µg/mL	0/90 (0.0)	0/85 (0.0)	0/97 (0.0)	0/87 (0.0)	0/80 (0.0)	0/88 (0.0)	0/527 (0.0)
Ofloxacin 2 µg/mL	0/40 (0.0)	0/42 (0.0)	0/52 (0.0)	0/58 (0.0)	0/47 (0.0)	0/54 (0.0)	0/293 (0.0)

Fluoroquinolones tested, and the concentrations at which they are tested, have been updated during the trial in accordance with WHO guidance. For this reason, results are not uniformly available across members of the drug class and concentrations tested.

Table S12. Baseline characteristics of participants by country (mITT Population)

Baseline characteristics	Georgia	India	Kazakhstan	Lesotho	Peru	Pakistan	South Africa	Total
**N (%)**	12 (1.7%)	23 (3.3%)	167 (24.0%)	77 (11.1%)	276 (39.5%)	92 (13.2%)	52 (7.5%)	699 (100.0%)
**Sex**								
Male	9 (75.0%)	9 (39.1%)	104 (62.3%)	42 (54.5%)	190 (68.8%)	49 (53.3%)	32 (61.5%)	435 (62.2%)
Female	3 (25.0%)	14 (60.9%)	63 (37.7%)	35 (45.5%)	86 (31.2%)	43 (46.7%)	20 (38.5%)	264 (37.8%)
**Age**	34.0 [25.5; 38.5]	25.0 [19.0; 41.0]	34.0 [26.0; 45.0]	39.0 [31.0; 48.0]	27.0 [21.0; 44.0]	36.0 [24.5; 45.5]	32.5 [28.0; 41.0]	32.0 [23.0; 44.0]
**BMI**	19.4 [18.6; 21.1]	17.8 [15.9; 20.9]	20.5 [19.0; 22.3]	19.0 [17.5; 21.7]	21.4 [19.4; 24.1]	17.2 [15.6; 20.0]	20.2 [17.7; 22.8]	20.4 [18.0; 22.8]
**ECOG grade**								
0	5 (41.7%)	11 (47.8%)	67 (40.1%)	29 (37.7%)	40 (14.5%)	59 (64.1%)	42 (80.8%)	253 (36.2%)
1	7 (58.3%)	10 (43.5%)	70 (41.9%)	32 (41.6%)	186 (67.4%)	26 (28.3%)	9 (17.3%)	340 (48.6%)
2	0 (0.0%)	2 (8.7%)	17 (10.2%)	15 (19.5%)	47 (17.0%)	6 (6.5%)	1 (1.9%)	88 (12.6%)
3	0 (0.0%)	0 (0.0%)	13 (7.8%)	1 (1.3%)	3 (1.1%)	1 (1.1%)	0 (0.0%)	18 (2.6%)
**HIV test result at baseline**								
Positive	0 (0.0%)	0 (0.0%)	8 (4.8%)	55 (71.4%)	4 (1.4%)	1 (1.1%)	30 (57.7%)	98 (14%)
Negative	12 (100.0%)	23 (100.0%)	159 (95.2%)	22 (28.6%)	272 (98.6%)	91 (98.9%)	22 (42.3%)	601 (86%)
**CD4 cell count**	-	-	228.0 [116.0; 523.5]	329.0 [134.0; 499.5]	767.5 [241.0; 1,294.0]	-	212.0 [80.0; 473.0]	296.0 [118.0; 497.0]
**Antiretroviral treatment at baseline**								
No	0 (0.0%)	0 (0.0%)	5 (62.5%)	10 (18.2%)	1 (25.0%)	1 (100.0%)	13 (43.3%)	30 (30.6%)
Yes	0 (0.0%)	0 (0.0%)	3 (37.5%)	45 (81.8%)	3 (75.0%)	0 (0.0%)	17 (56.7%)	68 (69.4%)
**HBsAg positive at baseline**								
Positive	0 (0.0%)	0 (0.0%)	3 (1.8%)	6 (7.9%)	1 (0.4%)	3 (3.3%)	3 (5.8%)	16 (2.3%)
Negative	12 (100.0%)	23 (100.0%)	164 (98.2%)	71 (92.2%)	275 (99.6%)	89 (96.7%)	49 (94.2%)	683 (97.7%)
**Hepatitis C at baseline**								
Positive	2 (16.7%)	0 (0.0%)	15 (9.0%)	1 (1.3%)	0 (0.0%)	8 (8.7%)	0 (0.0%)	26 (3.7%)
Negative	10 (83.3%)	23 (100.0%)	152 (91.0%)	76 (98.7%)	276 (100.0%)	84 (91.3%)	52 (100.0%)	673 (96.3%)
Unknown	0 (0.0%)	0 (0.0%)	0 (0.0%)	1 (1.3%)	0 (0.0%)	0 (0.0%)	0 (0.0%)	1 (0.1%)
**Diabetes**								
No	10 (83.3%)	18 (78.3%)	144 (86.2%)	68 (88.3%)	227 (82.2%)	75 (81.5%)	51 (98.1%)	593 (84.8%)
Yes	2 (16.7%)	5 (21.7%)	23 (13.8%)	8 (10.4%)	49 (17.8%)	17 (18.5%)	1 (1.9%)	105 (15.0%)
Unknown	0 (0.0%)	0 (0.0%)	0 (0.0%)	1 (1.3%)	0 (0.0%)	0 (0.0%)	0 (0.0%)	1 (0.1%)
**Smear result (screening)**								
Negative/Scanty	2 (16.7%)	8 (34.8%)	41 (24.6%)	4 (5.2%)	56 (20.3%)	8 (8.7%)	12 (23.1%)	131 (18.7%)
1-2+	6 (50.0%)	13 (56.5%)	101 (60.5%)	31 (40.3%)	98 (35.5%)	48 (52.2%)	21 (40.4%)	318 (45.5%)

Baseline characteristics	Georgia	India	Kazakhstan	Lesotho	Peru	Pakistan	South Africa	Total
3+	4 (33.3%)	2 (8.7%)	25 (15.0%)	42 (54.5%)	122 (44.2%)	36 (39.1%)	19 (36.5%)	250 (35.8%)
**Cavitation**								
No	3 (25.0%)	14 (60.9%)	62 (37.1%)	39 (50.7%)	101 (36.6%)	53 (57.6%)	23 (44.3%)	295 (42.3%)
Yes	9 (75.0%)	9 (39.1%)	105 (62.9%)	35 (45.4%)	175 (63.4%)	39 (42.4%)	27 (51.9%)	399 (57.2%)
Unknown	0 (0.0%)	0 (0.0%)	0 (0.0%)	3 (3.9%)	0 (0.0%)	0 (0.0%)	2 (3.8%)	3 (0.4%)
**Extent of disease**								
Limited	4 (33.3%)	7 (30.4%)	17 (10.2%)	24 (31.2%)	38 (13.8%)	13 (14.1%)	11 (21.2%)	114 (16.3%)
Moderate	2 (16.7%)	14 (60.9%)	116 (69.5%)	26 (33.8%)	169 (61.2%)	68 (73.9%)	31 (59.6%)	426 (60.9%)
Extensive	6 (50.0%)	2 (8.7%)	34 (20.4%)	24 (31.2%)	69 (25.0%)	11 (12.0%)	8 (15.4%)	154 (22.0%)
Unknown	0 (0.0%)	0 (0.0%)	0 (0.0%)	3 (3.9%)	0 (0.0%)	0 (0.0%)	2 (3.8%)	5 (0.7%)
**Prior exposure to TB treatment**								
None	7 (58.3%)	15 (65.2%)	113 (67.7%)	40 (51.9%)	200 (72.5%)	52 (56.5%)	20 (38.5%)	447 (63.9%)
First line only	5 (41.7%)	5 (21.7%)	2 (1.2%)	36 (46.8%)	44 (15.9%)	35 (38.0%)	22 (42.3%)	149 (21.3%)
Other	0 (0.0%)	2 (8.7%)	49 (29.3%)	0 (0.0%)	16 (5.8%)	3 (3.3%)	8 (15.4%)	78 (11.2%)
Unknown	0 (0.0%)	1 (4.3%)	3 (1.8%)	1 (1.3%)	16 (5.8%)	2 (2.2%)	2 (3.8%)	25 (3.6%)
**Pyrazinamide resistance**								
Sensitive	6 (50.0%)	9 (39.1%)	58 (35.2%)	45 (58.4%)	94 (34.1%)	66 (74.2%)	39 (75.0%)	317 (45.7%)
Resistant	6 (50.0%)	14 (60.9%)	107 (64.8%)	32 (41.6%)	182 (65.9%)	23 (25.8%)	13 (25.0%)	377 (54.3%)
**Injectable resistance**								
Sensitive	8 (66.7%)	18 (78.3%)	134 (81.2%)	74 (96.1%)	231 (83.7%)	88 (98.9%)	51 (98.1%)	604 (87.0%)
Resistant	4 (33.3%)	5 (21.7%)	31 (18.8%)	3 (3.9%)	45 (16.3%)	1 (1.1%)	1 (1.9%)	90 (13.0%)

Table S13. endTB Trial Control Regimen composition and duration at initiation of treatment, by country (mITT Population)

	Georgia	India	Kazakhstan	Lesotho	Peru	Pakistan	South Africa	Total
N (%)	3 (2.5%)	4 (3.4%)	23 (19.3%)	12 (10.1%)	51 (42.9%)	18 (15.1%)	8 (6.7%)	119 (100.0%)
**Number of drugs in regimen**								
4	0 (0.0%)	0 (0.0%)	0 (0.0%)	0 (0.0%)	0 (0.0%)	1 (5.6%)	0 (0.0%)	1 (0.8%)
5	2 (66.7%)	1 (25.0%)	16 (69.6%)	8 (66.7%)	39 (76.5%)	16 (88.9%)	5 (62.5%)	87 (73.1%)
6	1 (33.3%)	0 (0.0%)	5 (21.7%)	4 (33.3%)	12 (23.5%)	0 (0.0%)	2 (25.0%)	24 (20.2%)
7	0 (0.0%)	3 (75.0%)	2 (8.7%)	0 (0.0%)	0 (0.0%)	1 (5.6%)	1 (12.5%)	7 (5.9%)
**Drugs**								
WHO Group A								
Bedaquiline (B)	0 (0.0%)	4 (100.0%)	18 (78.3%)	9 (75.0%)	39 (76.5%)	18 (100.0%)	8 (100.0%)	96 (80.7%)
Levofloxacin (Lfx)	2 (66.7%)	4 (100.0%)	22 (95.7%)	12 (100.0%)	51 (100.0%)	14 (77.8%)	8 (100.0%)	113 (95.0%)
Moxifloxacin (M)	1 (33.3%)	0 (0.0%)	1 (4.3%)	0 (0.0%)	0 (0.0%)	4 (22.2%)	0 (0.0%)	6 (5.0%)
Linezolid (L)	0 (0.0%)	1 (25.0%)	18 (78.3%)	7 (58.3%)	39 (76.5%)	17 (94.4%)	4 (50.0%)	86 (72.3%)
WHO Group B								
Clofazimine (Cfz)	0 (0.0%)	4 (100.0%)	18 (78.3%)	10 (83.3%)	36 (70.6%)	18 (100.0%)	8 (100.0%)	94 (79.0%)
Cycloserine/Terizidone (Cs-Tzd)	3 (100.0%)	1 (25.0%)	21 (91.3%)	7 (58.3%)	35 (68.6%)	14 (77.8%)	4 (50.0%)	85 (71.4%)
WHO Group C								
Ethambutol (E)	0 (0.0%)	3 (75.0%)	4 (17.4%)	0 (0.0%)	12 (23.5%)	1 (5.6%)	1 (12.5%)	21 (17.6%)
Isoniazid (H)	0 (0.0%)	3 (75.0%)	0 (0.0%)	0 (0.0%)	0 (0.0%)	1 (5.6%)	2 (25.0%)	6 (5.0%)
Pyrazinamide (Z)	2 (66.7%)	3 (75.0%)	8 (34.8%)	4 (33.3%)	31 (60.8%)	1 (5.6%)	6 (75.0%)	55 (46.2%)
Delamanid (D)	2 (66.7%)	0 (0.0%)	1 (4.3%)	7 (58.3%)	0 (0.0%)	2 (11.1%)	0 (0.0%)	12 (10.1%)
Ethionamide/Prothionamide (Eto-Pto)	2 (66.7%)	3 (75.0%)	5 (21.7%)	4 (33.3%)	12 (23.5%)	1 (5.6%)	3 (37.5%)	30 (25.2%)
Para-aminosalacylic acid (PAS)	1 (33.3%)	0 (0.0%)	3 (13.0%)	2 (16.7%)	0 (0.0%)	0 (0.0%)	0 (0.0%)	6 (5.0%)
Capreomycin (CM)	3 (100.0%)	0 (0.0%)	5 (21.7%)	0 (0.0%)	5 (9.8%)	0 (0.0%)	0 (0.0%)	13 (10.9%)
Kanamycin (KM)	0 (0.0%)	0 (0.0%)	0 (0.0%)	2 (16.7%)	7 (13.7%)	0 (0.0%)	0 (0.0%)	9 (7.6%)
**Regimen**								
B/Cfz/E/Eto-Pto/H/Lfx/Z	0 (0.0%)	3 (75.0%)	0 (0.0%)	0 (0.0%)	0 (0.0%)	1 (5.6%)	1 (12.5%)	5 (4.2%)
B/Cfz/Cs-Tzd/L/M	0 (0.0%)	0 (0.0%)	0 (0.0%)	0 (0.0%)	0 (0.0%)	2 (11.1%)	0 (0.0%)	2 (1.7%)
B/Cfz/Cs-Tzd/L/Lfx	0 (0.0%)	1 (25.0%)	14 (60.9%)	3 (25.0%)	20 (39.2%)	12 (66.7%)	2 (25.0%)	52 (43.7%)
B/Cfz/Cs-Tzd/D/Lfx	0 (0.0%)	0 (0.0%)	0 (0.0%)	2 (16.7%)	0 (0.0%)	0 (0.0%)	0 (0.0%)	2 (1.7%)
B/Cfz/D/L/Lfx	0 (0.0%)	0 (0.0%)	1 (4.4%)	3 (25.0%)	0 (0.0%)	0 (0.0%)	0 (0.0%)	4 (3.4%)
B/Cfz/D/L/M	0 (0.0%)	0 (0.0%)	0 (0.0%)	0 (0.0%)	0 (0.0%)	2 (11.1%)	0 (0.0%)	2 (1.7%)
B/Cfz/Eto-Pto/Lfx/Z	0 (0.0%)	0 (0.0%)	0 (0.0%)	0 (0.0%)	0 (0.0%)	0 (0.0%)	2 (25.0%)	2 (1.7%)
B/Cfz/H/Lfx/Z	0 (0.0%)	0 (0.0%)	0 (0.0%)	0 (0.0%)	0 (0.0%)	0 (0.0%)	1 (12.5%)	1 (0.8%)

	Georgia	India	Kazakhstan	Lesotho	Peru	Pakistan	South Africa	Total
B/Cfz/L/Lfx/Z	0 (0.0%)	0 (0.0%)	1 (4.4%)	0 (0.0%)	16 (31.4%)	0 (0.0%)	0 (0.0%)	17 (14.3%)
B/Cfz/L/Lfx	0 (0.0%)	0 (0.0%)	0 (0.0%)	0 (0.0%)	0 (0.0%)	1 (5.6%)	0 (0.0%)	1 (0.8%)
B/Cfz/Cs-Tzd/L/M/Z	0 (0.0%)	0 (0.0%)	1 (4.4%)	0 (0.0%)	0 (0.0%)	0 (0.0%)	0 (0.0%)	1 (0.8%)
B/Cfz/Cs-Tzd/L/Lfx/Z	0 (0.0%)	0 (0.0%)	1 (4.4%)	0 (0.0%)	0 (0.0%)	0 (0.0%)	2 (25.0%)	3 (2.5%)
B/Cfz/D/Eto-Pto/Lfx/Z	0 (0.0%)	0 (0.0%)	0 (0.0%)	1 (8.3%)	0 (0.0%)	0 (0.0%)	0 (0.0%)	1 (0.8%)
B/Cs-Tzd/L/Lfx/Z	0 (0.0%)	0 (0.0%)	0 (0.0%)	0 (0.0%)	3 (5.9%)	0 (0.0%)	0 (0.0%)	3 (2.5%)
CM/Cs-Tzd/D/Lfx/PAS	1 (33.3%)	0 (0.0%)	0 (0.0%)	0 (0.0%)	0 (0.0%)	0 (0.0%)	0 (0.0%)	1 (0.8%)
CM/Cs-Tzd/Eto-Pto/M/Z	1 (33.3%)	0 (0.0%)	0 (0.0%)	0 (0.0%)	0 (0.0%)	0 (0.0%)	0 (0.0%)	1 (0.8%)
CM/Cs-Tzd/D/Eto-Pto/Lfx/Z	1 (33.3%)	0 (0.0%)	0 (0.0%)	0 (0.0%)	0 (0.0%)	0 (0.0%)	0 (0.0%)	1 (0.8%)
CM/Cs-Tzd/E/Eto-Pto/Lfx/Z	0 (0.0%)	0 (0.0%)	2 (8.7%)	0 (0.0%)	5 (9.8%)	0 (0.0%)	0 (0.0%)	7 (5.9%)
CM/Cs-Tzd/E/Eto-Pto/Lfx/PAS/Z	0 (0.0%)	0 (0.0%)	2 (8.7%)	0 (0.0%)	0 (0.0%)	0 (0.0%)	0 (0.0%)	2 (1.7%)
CM/Cs-Tzd/Eto-Pto/Lfx/PAS/Z	0 (0.0%)	0 (0.0%)	1 (4.4%)	0 (0.0%)	0 (0.0%)	0 (0.0%)	0 (0.0%)	1 (0.8%)
Cs-Tzd/Eto-Pto/KM/Lfx/PAS /Z	0 (0.0%)	0 (0.0%)	0 (0.0%)	2 (16.7%)	0 (0.0%)	0 (0.0%)	0 (0.0%)	2 (1.7%)
Cs-Tzd/D/Eto-Pto/L/Lfx/Z	0 (0.0%)	0 (0.0%)	0 (0.0%)	1 (8.3%)	0 (0.0%)	0 (0.0%)	0 (0.0%)	1 (0.8%)
Cs-Tzd/E/Eto-Pto/KM/Lfx /Z	0 (0.0%)	0 (0.0%)	0 (0.0%)	0 (0.0%)	7 (13.7%)	0 (0.0%)	0 (0.0%)	7 (5.9%)
**Other regimen descriptors**								
Any 2nd line injectable*	3 (100.0%)	0 (0.0%)	5 (21.7%)	2 (16.7%)	12 (23.5%)	0 (0.0%)	0 (0.0%)	22 (18.5%)
Shorter 9-12 month regimen^	0 (0.0%)	3 (75.0%)	0 (0.0%)	0 (0.0%)	0 (0.0%)	1 (5.6%)	1 (12.5%)	5 (4.2%)
Treatment duration-longer regimen (weeks) **								
N	2	1	18	10	34	17	6	88
Mean (SD)	89.9 (4.0)	62.7	80.0 (5.3)	80.6 (0.6)	79.5 (5.2)	78.4 (1.1)	73.2 (2.1)	79.1 (5.0)
Median [IQR]	89.9 [87.0;92.7]	62.7 [62.7;62.7]	78.3 [78.1;79.0]	80.8 [80.1;81.0]	78.0 [78.0;79.7]	78.0 [78.0;78.3]	72.0 [71.9;74.9]	78.1 [78.0;80.0]
Min-Max	87.0 - 92.7	62.7 - 62.7	71.1 - 95.9	79.1 - 81.3	72.0 - 100.6	78.0 - 82.7	71.9 - 76.9	62.7 - 100.6
**Duration by drug**								
Levofloxacin	n=1<br>87.0<br>[87.0; 87.0]	n=1<br>62.7<br>[62.7; 62.7]	n=17<br>78.1<br>[71.1; 79.0]	n=10<br>80.6<br>[80.0; 80.9]	n=34<br>78.0<br>[78.0; 79.7]	n=13<br>78.0<br>[78.0; 78.3]	n=6<br>72.0<br>[71.9; 74.9]	n=82<br>78.0<br>[76.9; 80.0]
Bedaquiline	n=0<br>-	n=1<br>62.7<br>[62.7; 62.7]	n=17<br>38.7<br>[25.9; 78.1]	n=7<br>79.1<br>[4.9; 80.7]	n=28<br>71.8<br>[53.6; 78.0]	n=17<br>77.9<br>[77.9; 78.0]	n=6<br>52.1<br>[27.6; 71.9]	n=76<br>71.8<br>[51.9; 78.0]
Clofazimine	n=0<br>-	n=1<br>62.7<br>[62.7; 62.7]	n=16<br>78.1<br>[68.0; 78.3]	n=8<br>80.8<br>[80.3; 80.9]	n=24<br>78.0<br>[75.5; 78.0]	n=17<br>78.0<br>[78.0; 78.3]	n=6<br>72.0<br>[71.9; 74.9]	n=72<br>78.0<br>[76.1; 78.4]
Linezolid	n=2<br>67.1<br>[60.6; 73.6]	n=1<br>62.6<br>[62.6; 62.6]	n=17<br>78.1<br>[64.0; 78.3]	n=6<br>66.9<br>[31.6; 80.0]	n=27<br>78.0<br>[76.0; 78.0]	n=17<br>78.0<br>[78.0; 78.3]	n=4<br>21.0<br>[16.4; 48.6]	n=74<br>78.0<br>[67.0; 78.1]

	Georgia	India	Kazakhstan	Lesotho	Peru	Pakistan	South Africa	Total
Cycloserine/Terizidone	n=2	n=1	n=18	n=6	n=23	n=14	n=4	n=68
	89.9	33.6	78.2	80.9	78.0	78.0	71.9	78.0
	[87.0; 92.7]	[33.6; 33.6]	[58.4; 79.0]	[80.0; 81.1]	[76.0; 80.0]	[54.0; 78.0]	[50.9; 72.0]	[71.5; 80.0]
Pyrazinamide	n=2	n=0	n=4	n=4	n=21	n=0	n=4	n=35
	89.9	-	57.8	44.3	78.0	-	55.5	78.0
	[87.0; 92.7]		[32.9; 87.2]	[7.8; 80.9]	[78.0; 79.7]		[22.1; 74.4]	[37.0; 80.6]
Ethionamide/Prothionamide	n=2	n=0	n=3	n=4	n=7	n=0	n=2	n=18
	34.2	-	67.3	80.4	79.1	-	75.9	78.4
	[13.4; 55.0]		[7.7; 87.3]	[48.1; 81.0]	[78.0; 80.0]		[74.9; 76.9]	[67.3; 80.0]
Ethambutol	n=0	n=0	n=3	n=0	n=7	n=0	n=0	n=10
	-	-	12.6	-	80.0	-	-	79.2
			[2.6; 20.6]		[78.7; 93.3]			[20.6; 82.3]
Capreomycin	n=2	n=0	n=3	n=0	n=5	n=0	n=0	n=10
	35.4	-	31.9	-	16.3	-	-	29.8
	[32.1; 38.7]		[28.7; 37.0]		[16.0; 23.0]			[16.3; 32.1]
Delamanid	n=1	n=0	n=2	n=10	n=0	n=3	n=0	n=16
	51.7	-	64.3	79.6	-	78.0	-	78.0
	[51.7; 51.7]		[50.3; 78.3]	[66.9; 80.7]		[42.4; 78.0]		[51.9; 80.4]
Kanamycin	n=0	n=0	n=0	n=2	n=4	n=0	n=0	n=6
				19.1	27.9		-	25.6
				[14.3; 23.9]	[18.7; 32.1]			[14.3; 28.4]
Moxifloxacin	n=1	n=0	n=2	n=0	n=0	n=4	n=0	n=7
	92.7	-	39.4	-	-	78.0	-	78.0
	[92.7; 92.7]		[0.1; 78.6]			[78.0; 78.0]		[78.0; 78.6]
Para-aminosalicylic acid	n=0	n=0	n=2	n=2	n=0	n=0	n=0	n=4
	-	-	49.9	24.6	-	-	-	24.6
			[12.6; 87.3]	[21.3; 27.9]				[16.9; 57.6]

^ Shorter all-oral bedaquiline-containing treatment regimen of 9-12 months, as recommended by WHO (2019 rapid communication, 2020 consolidated guidelines)

* Amikacin, Capreomycin, Kanamycin

** not reported for shorter 9-12 regimen and early treatment discontinuations of longer regimens

Table S14. Distribution of endTB Control Group Regimens by WHO Guideline Period and Conformity with Guidelines

Latest WHO Guidelines to which regimen conformed	WHO recommendations used to guide composition of the control arm by time of randomization			
	**Period 1 (n=31)**	**Period 2 (n=40)**	**Period 3 (n=48)**	**All periods (n=119)**
**2022 Guidelines**				**97 (81.5%)**
3 Group A/1-2 Group B/± Group C	4 (12.9%)	38 (95.0%)	43 (89.6%)	85 (71.4%)
Shorter regimen	0 (0.0%)	1 (2.5%)	4 (8.3%)	5 (4.2%)
2 Group A/1-2 Group B/+ Group C	5 (16.1%)	1 (2.5%)	1 (2.1%)	7 (5.9%)
**earlier WHO Guidelines**				**22 (18.5%)**
Injectable/FQ/+ Group C	22 (71.0%)	0 (0.0%)	0 (0.0%)	22 (18.5%)

WHO Drug Group Classification
Group A: Fluoroquinolone (FQ): levofloxacin, moxifloxacin; bedaquiline, linezolid
Group B: Clofazimine, cycloserine/terizidone
Group C: Ethambutol, isoniazid, pyrazinamide, delamanid, ethionamide-prothionamide, PAS, capreomycin, kanamycin

Although the trial completed randomization before the 2022 WHO Guidelines were released, interpretation of endTB results in light of the current Guidelines is of interest. Table S14 summarizes the distribution of regimens conforming with current (or prior) WHO Guideline by randomization period. The period was defined by the release date of the (amended) endTB SOP that guided regimen composition. Period 1 was informed by 2016 Guidelines and lasted until 17 Dec 2018.(5) Period 2 was informed by the 2018 Rapid Communication (17) and started on 18 Dec 2018 and ended on 30 Mar 2020. Period 3 was informed by the 2019 Rapid Communication (18) from 31 March 2020 through the end of randomization. All control-arm participants received regimens that conformed to WHO Guidelines in force during their randomization. Most (97, 81.5%) participants started on regimens that conform with 2022 Guidance (19) while 22 (18.5%) participants started on regimens that conformed with 2016 Guidance and do not conform to 2022 Guidance.

Table S15. Primary efficacy outcomes at Week 73 in the per-protocol population

Outcome	9BLMZ (N = 98)	9BCLLfxZ (N = 95)	9BDLLfxZ (N = 103)	9DCLLfxZ (N = 96)	9DCMZ (N = 96)	Control (N = 74)	Total (N = 562)
**Favorable**							
Participants - no. (%)	94 (95.9%)	91 (95.8%)	97 (94.2%)	82 (85.4%)	82 (85.4%)	71 (95.9%)	517 (92.0%)
Absolute difference from control (%, 95% CI)	0.0% (-6.0%;5.9%)	-0.2% (-6.2%;5.9%)	-1.8% (-8.1%;4.6%)	-10.5% (-18.9%;-2.2%)	-10.5% (-18.9%;-2.2%)	--	--
Participants with negative culture results, Week 65 and 73 – no. (%)	93 (94.9%)	88 (92.6%)	95 (92.2%)	80 (83.3%)	81 (84.4%)	69 (93.2%)	506 (90.0%)
Participants with favorable bacteriological, clinical and radiological evolution^^ – no. (%)	1 (1.0%)	3 (3.2%)	2 (1.9%)	2 (2.1%)	1 (1.0%)	2 (2.7%)	11 (2.0%)
**Unfavorable**							
Participants – no. (%)	4 (4.1%)	4 (4.2%)	6 (5.8%)	14 (14.6%)	14 (14.6%)	3 (4.1%)	45 (8.0%)
Death, all cause – no. (%)££	2 (2.0%)	0 (0.0%)	3 (2.9%)	3 (3.1%)	2 (2.1%)	2 (2.7%)	12 (2.1%)
Participants with positive culture results** – no. (%)	1 (1.0%)	3 (3.2%)	1 (1.0%)	10 (10.4%)	8 (8.3%)	1 (1.4%)	24 (4.3%)
Participants with recurrence§§ – no. (%)	0 (0.0%)	0 (0.0%)	0 (0.0%)	0 (0.0%)	2 (2.1%)	0 (0.0%)	2 (0.4%)
Participants with permanent treatment discontinuation due to adverse event – no. (%)	0 (0.0%)	0 (0.0%)	0 (0.0%)	0 (0.0%)	0 (0.0%)	0 (0.0%)	0 (0.0%)
Participants with poor treatment adherence/lost to follow-up – no. (%)	0 (0.0%)	0 (0.0%)	0 (0.0%)	0 (0.0%)	1 (1.0%)	0 (0.0%)	1 (0.2%)
Participants who withdrew consent – no. (%)	0 (0.0%)	0 (0.0%)	1 (1.0%)	0 (0.0%)	0 (0.0%)	0 (0.0%)	1 (0.2%)
Participants with other unfavorable outcome## – no. (%)	1 (1.0%)	1 (1.1%)	1 (1.0%)	1 (1.0%)	1 (1.0%)	0 (0.0%)	5 (0.9%)

A protocol-consistent course of treatment was 80% of expected doses taken within 120% of the intended regimen duration. Participants who received more than 7 days of either a prohibited concomitant medication or a study drug not prescribed according to protocol were also excluded from the per-protocol population. ^^participants without culture results between Week 65 and Week 73; ££12 PP participants experienced death as a treatment outcome, 1 participant in the safety population who was excluded from the mITT population also experienced death. 1 participant in the

mITT population was assigned positive culture result as unfavorable outcome at 73 weeks and later died; 1 death occurred in a participant in the mITT population, who was excluded from PP population. Exclusion occurred because the participant did not complete a protocol-consistent course of treatment. Death occurred after treatment was stopped and was not the reason for receiving less than 80% of doses. **participants who permanently discontinued treatment because of a positive sputum culture at Week 16 or later, or who had a positive sputum culture between Week 65 and Week 73; §§participants who, after treatment completion, had a positive sputum culture or started a new treatment regimen; ##participants with other unfavorable outcome: not assessable after completing treatment (n=5).

Table S16. Unadjusted Analyses of Efficacy of endTB Trial Regimens at 39 Weeks (mITT Population)

Week 39 treatment outcome	9BLMZ (n= 118)	9BCLLfxZ (n= 115)	9BDLLfxZ (n= 122)	9DCLLFxZ (n= 118)	9DCMZ (n= 107)	Control (n= 119)	Total (N = 699)
**Total Favorable**	106 (89.8%)	105 (91.3%)	103 (84.4%)	94 (79.7%)	87 (81.3%)	104 (87.4%)	599 (85.7%)
95% CI	[82.9%;94.6%]	[84.6%;95.8%]	[76.8%;90.4%]	[71.3%;86.5%]	[72.6%;88.2%]	[80.1%;92.8%]	[82.9%;88.2%]
**Absolute difference from control**	2.4%	3.9%	-3.0%	-7.7%	-6.1%	-	
95% CI around the difference	[-5.6%;10.5%]	[-4.0%;11.8%]	[-11.7%;5.8%]	[-17.1%;1.7%]	[-15.6%;3.4%]		

Note: Confidence intervals widths have not been adjusted for multiplicity and the intervals should not be used in place of hypothesis testing for secondary outcomes. They are only presented for precision purposes.

Table S17. Unadjusted Analyses of Efficacy of endTB Trial Regimens at 39 Weeks (PP Population)

Week 39 treatment outcome	9BLMZ (n = 98)	9BCLLfxZ (n = 95)	9BDLLfxZ (n = 103)	9DCLLfxZ (n = 96)	9DCMZ (n = 96)	Control (n = 74)	Total (N = 562)
**Total Favorable^**	94 (95.9%)	92 (96.8%)	96 (93.2%)	85 (88.5%)	80 (83.3%)	71 (95.9%)	518 (92.2%)
95% CI	[89.9%;98.9%]	[91.0%;99.3%]	[86.5%;97.2%]	[80.4%;94.1%]	[74.4%;90.2%]	[88.6%;99.2%]	[89.6%;94.3%]
**Absolute difference from control**	0.0%	0.9%	-2.7%	-7.4%	-12.6%	-	
95% CI around the difference	[-6.0%;5.9%]	[-4.8%;6.6%]	[-9.4%;3.9%]	[-15.2%;0.4%]	[-21.3%;-3.9%]		

^ All cultures negative

Note: Confidence intervals widths have not been adjusted for multiplicity and the intervals should not be used in place of hypothesis testing for secondary outcomes. They are only presented for precision purposes.

Table S18. Unadjusted Analyses of Efficacy of endTB Trial Regimens at 104 Weeks (mITT Population)

Week 104 treatment outcome	9BLMZ (n = 118)	9BCLLfxZ (n = 115)	9BDLLfxZ (n = 122)	9DCLLfxZ (n = 118)	9DCMZ (n = 107)	Control (n = 119)	Total (N = 699)
**Total Favorable**	101 (85.6%)	102 (88.7%)	103 (84.4%)	88 (74.6%)	88 (82.2%)	92 (77.3%)	574 (82.1%)
95% CI	[77.9%;91.4%]	[81.4%;93.8%]	[76.8%;90.4%]	[65.7%;82.1%]	[73.7%;89.0%]	[68.7%;84.5%]	[79.1%;84.9%]
**Absolute difference from control**	**8.3%**	**11.4%**	**7.1%**	**-2.7%**	**4.9%**		
95% CI around the difference	[-1.6%;18.1%]	[1.9%;20.9%]	[-2.8%;17.0%]	[-13.6%;8.1%]	[-5.5%;15.4%]		

Note: Confidence intervals widths have not been adjusted for multiplicity and the intervals should not be used in place of hypothesis testing. They are only presented for precision purposes.

Table S19. Unadjusted Analyses of Efficacy of endTB Trial Regimens at 104 Weeks (PP Population)

Week 104 treatment outcome	9BLMZ (n = 98)	9BCLLfxZ (n = 95)	9BDLLfxZ (n = 103)	9DCLLfxZ (n = 96)	9DCMZ (n = 96)	Control (n = 74)	Total (N = 562)
**Total Favorable**	91 (92.9%)	90 (94.7%)	97 (94.2%)	79 (82.3%)	81 (84.4%)	69 (93.2%)	507 (90.2%)
95% CI	[85.8%;97.1%]	[88.1%;98.3%]	[87.8%;97.8%]	[73.2%;89.3%]	75.5%;91.0%]	[84.9%;97.8%]	[87.5%;92.5%]
**Absolute difference from control**	**-0.4%**	**1.5%**	**0.9%**	**-11.0%**	**-8.9%**		
95% CI around the difference	[-8.0%;7.3%]	[-5.8%;8.8%]	[-6.4%;8.2%]	[-20.5%;-1.4%]	[-18.1%;0.4%]		

Note: Confidence intervals widths have not been adjusted for multiplicity and the intervals should not be used in place of hypothesis testing. They are only presented for precision purposes.

Table S20. Adjusted Analyses of Efficacy of endTB Trial Regimens at 73 Weeks (mITT Population)

Week 73 treatment outcome	9BLMZ (n = 118)	9BCLLfxZ (n = 115)	9BDLLfxZ (n = 122)	9DCLLfxZ (n = 118)	9DCMZ (n = 107)	Control (n = 119)	Total (N = 699)
**Total Favorable**	105 (89.0%)	104 (90.4%)	104 (85.2%)	93 (78.8%)	89 (83.2%)	96 (80.7%)	591 (84.5%)
95% CI	[81.9%;94.0%]	[83.5%;95.1%]	[77.7%;91.0%]	[70.3%;85.8%]	[74.7%;89.7%]	[72.4%;87.3%]	[82.0%;87.5%]
**Adjusted risk differences-pre-specified analysis*^**	**8.8%**	**9.5%**	**3.9%**	**-1.9%**	**1.9%**	**-**	
95% CI	[-0.6%;18.2%]	[0.4%;18.6%]	[-5.8%;13.6%]	[-12.5%;8.7%]	[-8.4%;12.3%]		

Backward selection: initial model includes pre-specified baseline variables that were significant in univariate analyses at p<0.10: BMI, age, hepatitis C, extent of disease

* significant (p<0.05) covariates in final model: hepatitis C, extent of disease. (n=694)

^No convergence: Poisson regression

Confidence intervals widths have not been adjusted for multiplicity and the intervals should not be used in place of hypothesis testing. They are only presented for precision purposes.

Week 73 treatment outcome	9BLMZ (n = 118)	9BCLLfxZ (n = 115)	9BDLLfxZ (n = 122)	9DCLLfxZ (n = 118)	9DCMZ (n = 107)	Control (n = 119)	Total (N = 699)
**Adjusted risk differences-post-hoc analysis[#]**	**9.9%**	**10.3%**	**5.0%**	**-0.9%**	**4.1%**	**-**	
95% CI	[0.7%;19.0%]	[1.2%;19.4%]	[-4.9%;14.9%]	[-11.4%;9.5%]	[-6.2%;14.5%]		

[#]Final, post-hoc model includes: PZA resistance, HIV, smear result, cavitation (n=689)

Confidence intervals widths have not been adjusted for multiplicity and the intervals should not be used in place of hypothesis testing. They are only presented for precision purposes.

Table S21. Adjusted Analyses of Efficacy of endTB Trial Regimens at 73 Weeks (PP Population)

Week 73 treatment outcome	9BLMZ (n = 98)	9BCLLfxZ (n = 95)	9BDLLfxZ (n = 103)	9DCLLfxZ (n = 96)	9DCMZ (n = 96)	Control (n = 74)	Total (N = 562)
**Total Favorable**	94 (95.9%)	91 (95.8%)	97 (94.2%)	82 (85.4%)	82 (85.4%)	71 (95.9%)	517 (92.0%)
95% CI	[89.9%;98.9%]	[89.6%;98.8%]	[87.8%;97.8%]	[76.7%;91.8%]	[76.7%;91.8%]	[88.6%;99.2%]	[89.4%;94.1%]
**Adjusted risk differences-pre-specified analysis*^**	**0.1%**	**0.3%**	**-2.9%**	**-12.1%**	**-10.8%**	**-**	
95% CI	[-6.2%; 6.4%]	[-6.1%; 6.7%]	[-10.2%; 4.4%]	[-21.0%; -3.2%]	[-19.3%;-2.2%]		

Backward selection: initial model includes pre-specified baseline variables that were significant in univariate analyses at p<0.10: BMI, age, Injectable resistance, smear result, extent of disease.

* significant covariates (p<0.05) in final model: age, BMI. (n=562)

^No convergence: Poisson regression

Confidence intervals widths have not been adjusted for multiplicity and the intervals should not be used in place of hypothesis testing. They are only presented for precision purposes.

**Adjusted risk differences-post-hoc analyses#^**	**-0.2%**	**-1.0%**	**-2.6%**	**-10.3%**	**-11.0%**	**-**
95% CI	[-6.4%;6.0%]	[-7.5%;5.4%]	[-9.4%;4.3%]	[-19.1%;-1.4%]	[-19.7%;-2.3%]	

#Final, post-hoc model includes: PZA resistance, HIV, smear result, cavitation (n=554)

^No convergence: Poisson regression

Confidence intervals widths have not been adjusted for multiplicity and the intervals should not be used in place of hypothesis testing. They are only presented for precision purposes.

Table S22. Adjusted (pre-specified) Analyses of Efficacy of endTB Trial Regimens at 39 Weeks (mITT Population)

Week 39 treatment outcome	9BLMZ (n= 118)	9BCLLfxZ (n= 115)	9BDLLfxZ (n= 122)	9DCLLFxZ (n= 118)	9DCMZ (n= 107)	Control (n= 119)	Total (N = 699)
**Total Favorable**	106 (89.8%)	105 (91.3%)	103 (84.4%)	94 (79.7%)	87 (81.3%)	104 (87.4%)	599 (85.7%)
95% CI	[82.9%;94.6%]	[84.6%;95.8%]	[76.8%;90.4%]	[71.3%;86.5%]	[72.6%;88.2%]	[80.1%;92.8%]	[82.9%;88.2%]
Adjusted risk differences*	4.1%	5.3%	-2.2%	-6.8%	-4.6%	-	
95% CI around the difference	[-3.9%;12.1%]	[-2.1%;12.7%]	[-10.8%;6.4%]	[-16.0%;2.5%]	[-14.1%;4.9%]		

Note: Confidence intervals widths have not been adjusted for multiplicity and the intervals should not be used in place of hypothesis testing. They are only presented for precision purposes.

Backward selection: initial model includes baseline variables: BMI, hepatitis C, extent of disease.

* covariate in final model: BMI. (n=699);

Table S23. Adjusted (pre-specified) Analyses of Efficacy of endTB Trial Regimens at 39 Weeks (PP Population)

**Week 39 treatment outcome**	**9BLMZ (n = 98)**	**9BCLLfxZ (n = 95)**	**9BDLLfxZ (n = 103)**	**9DCLLfxZ (n = 96)**	**9DCMZ (n = 96)**	**Control (n = 74)**	**Total (N = 562)**
**Total Favorable**	94 (95.9%)	92 (96.8%)	96 (93.2%)	85 (88.5%)	80 (83.3%)	71 (95.9%)	518 (92.2%)
95% CI	[89.9%;98.9%]	[91.0%;99.3%]	[86.5%;97.2%]	[80.4%;94.1%]	[74.4%;90.2%]	[88.6%;99.2%]	[89.6%;94.3%]
Adjusted risk differences*^	0.0%	1.2%	-4.1%	-8.9%	-13.0%	-	
95% CI around the difference	[-6.3%;6.2%]	[-4.9%;7.2%]	[-11.5%;3.3%]	[-17.1%;-0.6%]	[-21.9%;-4.1%]		

Note: Confidence intervals widths have not been adjusted for multiplicity and the intervals should not be used in place of hypothesis testing. They are only presented for precision purposes.
Backward selection: initial model includes baseline variables: BMI, age, HIV, smear result, extent of disease.
* covariates in final model: BMI, age. (n=562)
^*No convergence: Poisson regression*

Table S24. Adjusted (pre-specified) Analyses of Efficacy of endTB Trial Regimens at 104 Weeks (mITT Population)

**W104 treatment outcome**	**9BLMZ (n = 118)**	**9BCLLfxZ (n = 115)**	**9BDLLfxZ (n = 122)**	**9DCLLfxZ (n = 118)**	**9DCMZ (n = 107)**	**Control (n = 119)**	**Total (N = 699)**
**Total Favorable**	101 (85.6%)	102 (88.7%)	103 (84.4%)	88 (74.6%)	88 (82.2%)	92 (77.3%)	574 (82.1%)
95% CI	[77.9%;91.4%]	[81.4%;93.8%]	[76.8%;90.4%]	[65.7%;82.1%]	[73.7%;89.0%]	[68.7%;84.5%]	[79.1%;84.9%]
**Adjusted risk differences-pre-specified analysis*^**	**9.0%**	**11.1%**	**6.6%**	**-2.6%**	4.3%	**-**	
95% CI	[-1.2%;19.2%]	[1.4%;20.8%]	[-3.5%;16.7%]	[-13.9%;8.6%]	[-6.4%;15.1%]		

Note: Confidence intervals widths have not been adjusted for multiplicity and the intervals should not be used in place of hypothesis testing. They are only presented for precision purposes.
Backward selection: initial model includes baseline variables: BMI, pyrazinamide resistance, hepatitis C, extent of disease.
* covariates in final model: hepatitis C, extent of disease. (n=694)
^*No convergence: Poisson regression*

Table S25. Adjusted (pre-specified) Analyses of Efficacy of endTB Trial Regimens at 104 Weeks (PP Population)

**Week 104 treatment outcome**	**9BLMZ (n = 98)**	**9BCLLfxZ (n = 95)**	**9BDLLfxZ (n = 103)**	**9DCLLfxZ (n = 96)**	**9DCMZ (n = 96)**	**Control (n = 74)**	**Total (N = 562)**
**Total Favorable**	91 (92.9%)	90 (94.7%)	97 (94.2%)	79 (82.3%)	81 (84.4%)	69 (93.2%)	507 (90.2%)
95% CI	[85.8%;97.1%]	[88.1%;98.3%]	[87.8%;97.8%]	[73.2%;89.3%]	[75.5%;91.0%]	[84.9%;97.8%]	[87.5%;92.5%]
**Adjusted risk differences-pre-specified analysis*^**	**-0.4%**	**1.6%**	**-0.3%**	**-12.7%**	**-9.0%**	**-**	
95% CI	[-8.3%;7.6%]	[-6.0%;9.3%]	[-8.5%;7.8%]	[-22.7%;-2.8%]	[-18.4%;0.5%]		

Note: Confidence intervals widths have not been adjusted for multiplicity and the intervals should not be used in place of hypothesis testing. They are only presented for precision purposes.

Backward selection: initial model includes baseline variables: BMI, age, Injectable resistance, smear, extent of disease.

*covariates in final model: age, BMI. (n=562)

^*No convergence: Poisson regression*

Table S26. Sensitivity Analyses of Efficacy of endTB Trial Regimens at 73 Weeks (Assessable, all-culture mITT, all-DST mITT, and Assessable Reclassified populations)

Population	W73 treatment outcome	9BLMZ	9BCLLfxZ	9BDLLfxZ	9DCLLfxZ	9DCMZ	Control	Total
**1. Assessable**	**Total in population**	97 (100.0%)	94 (100.0%)	101 (100.0%)	94 (100.0%)	94 (100.0%)	74 (100.0%)	554 (100.0%)
	**Total Favorable**	94 (96.9%)	91 (96.8%)	97 (96.0%)	82 (87.2%)	82 (87.2%)	71 (95.9%)	517 (93.3%)
	95% CI	[91.2%; 99.4%]	[91.0%; 99.3%]	[90.2%; 98.9%]	[78.8%; 93.2%]	[78.8%; 93.2%]	[88.6%; 99.2%]	[90.9%; 95.3%]
	Difference from control	1.0%	0.9%	0.1%	-8.7%	-8.7%	-	
	95% CI of the difference	[-4.7%; 6.6%]	[-4.9 ; 6.6%]	[-5.8%; 6.0%]	[-16.8%; -0.6%]	[-16.8%; -0.6%]		
**2. all-culture mITT**	**Total in population**	124 (100.0%)	119 (100.0%)	125 (100.0%)	121 (100.0%)	116 (100.0%)	121 (100.0%)	726 (100.0%)
	**Total Favorable**	110 (88.7%)	107 (89.9%)	107 (85.6%)	96 (79.3%)	96 (82.8%)	98 (81.0%)	614 (84.6%)
	95% CI	[81.8%; 93.7%]	[83.0%; 94.7%]	[78.2%; 91.2%]	[71.0%; 86.2%]	[74.6%; 89.1%]	[72.9%; 87.6%]	[81.7%; 87.1%]
	Difference from control	7.7%	8.9%	4.6%	-1.7%	1.8%	-	
	95% CI of the difference	[-1.2%;16.7%]	[0.1; 17.8%]	[-4.7%; 13.9%]	[-11.7%; 8.4%]	[-8.0%; 11.6%]		
**3. all-DST mITT**	**Total in population**	118 (100.0%)	115 (100.0%)	122 (100.0%)	118 (100.0%)	108 (100.0%)	119 (100.0%)	700 (100.0%)
	**Total Favorable**	105 (89.0%)	104 (90.4%)	104 (85.3%)	93 (78.8%)	90 (83.3%)	96 (80.7%)	592 (84.6%)
	95% CI	[81.9%; 94.0%]	[83.5%; 95.1%]	[77.7%; 91.0%]	[70.3%; 85.8%]	[74.9%; 89.8%]	[72.4%; 87.3%]	[81.7%; 87.2%]
	Difference from control	8.3%	9.8%	4.6%	-1.9%	2.7%	-	
	95% CI of the difference	[-0.8%;17.4%]	[0.9; 18.7%]	[-4.9%; 14.1%]	[-12.1%; 8.4%]	[-7.3%; 12.6%]		
**4. Assessable reclassifying "Unfavorable because unassessable" to Week 39 outcome**	**Total in population**	98 (100.0%)	95 (100.0%)	103 (100.0%)	96 (100.0%)	96 (100.0%)	74 (100.0%)	562 (100.0%)
	**Total Favorable**	95 (96.9%)	92 (96.8%)	98 (95.2%)	83 (86.5%)	82 (85.4%)	71 (96.0%)	521 (92.7%)
	95% CI	[91.3%; 99.4%]	[91.0%; 99.3%]	[89.0%; 98.4%]	[78.0%; 92.6%]	[79.7%; 91.8%]	[88.6%; 99.2%]	[90.2%; 94.7%]
	Difference from control	1.0%	0.9%	-0.8%	-9.5%	-10.5%	-	
	95% CI of the difference	[-4.6%; 6.6%]	[-4.8 ; 6.6%]	[-6.9%; 5.3%]	[-17.7%; -1.3%]	[-18.9%; -0.2%]		

Note: Sensitivity analysis excluding participants from the control arm who received a shortened regimen, if the proportion exceeds 10% was not performed as the proportion of shortened regimen received in the control is 4.8% (6/126). Multiple imputation to impute missing data was not performed as missing values in the final multivariable model reduce the relevant analysis population by only 0.7% (<20%). Confidence intervals widths have not been adjusted for multiplicity and the intervals should not be used in place of hypothesis testing. They are only presented for precision purposes.

Table S27. Post-hoc sensitivity analysis of efficacy of endTB Trial Regimens at 73 Weeks (mITT population): reclassification of W73 treatment outcome of participants in control arm with longer regimen with addition or change of one or more investigational drug in their regimen as unfavourable outcome (as in experimental arms)

Population	W73 treatment outcome	9BLMZ	9BCLLfxZ	9BDLLfxZ	9DCLLfxZ	9DCMZ	Control	Total
**mITT reclassifying outcomes « favorable" to "Unfavorable" in participants from control arm, longer regimen with addition or replacement of one drug**	Total in population	118 (100.0%)	115 (100.0%)	122 (100.0%)	118 (100.0%)	107 (100.0%)	119 (100.0%)	699 (100.0%)
	Total Favorable	105 (89.0%)	104 (90.4%)	104 (85.2%)	93 (78.8%)	89 (83.2%)	81 (68.1%)	576 (82.4%)
	95% CI	[81.9%;94.0%]	[83.5%;95.1%]	[77.7%;91.0%]	[70.3%;85.8%]	[74.7%;89.7%]	[58.9%;76.3%]	[79.4%;85.2%]
	Difference from	20.9%	22.4%	17.2%	10.7%	15.1%	-	
	95% CI of the	[10.8%;31.0%]	[12.4%;32.3%]	[6.7%;27.7%]	[-0.4%;21.9%]	[4.1%;26.1%]		

Note: Confidence intervals widths have not been adjusted for multiplicity and the intervals should not be used in place of hypothesis testing. They are only presented for precision purposes.

Table S28. Sensitivity Analyses of Efficacy of endTB Trial Regimens at 104 Weeks (Assessable, all-culture mITT, all-DST mITT, and Assessable Reclassified populations)

N°	W104 treatment outcome	9BLMZ	9BCLLfxZ	9BDLLfxZ	9DCLLfxZ	9DCMZ	Control	Total
**Table S1. Assessable**	**Total in population**	94 (100.0%)	92 (100.0%)	101 (100.0%)	91 (100.0%)	91 (100.0%)	73 (100.0%)	542 (100.0%)
	**Total Favorable**	91 (96.8%)	90 (97.8%)	97 (96.0%)	79 (86.8%)	81 (89.0%)	69 (94.5%)	507 (93.5%)
	95% CI	[91.0%; 99.3%]	[92.4%; 99.7%]	[90.2%; 98.9%]	[78.1%; 93.0%]	[80.7%; 94.6%]	[86.6%; 98.5%]	[91.1%; 95.5%]
	Difference from control	2.3%	3.3%	1.5%	-7.7%	-5.5%	-	
	95% CI of the difference	[-4.0%; 8.6%]	[-2.7%; 9.3%]	[-4.9%; 8.0%]	[-16.4%; 1.0%]	[-13.8%; 2.8%]		
**Table S2. all-culture mITT**	**Total in population**	124 (100.0%)	119 (100.0%)	125 (100.0%)	121 (100.0%)	116 (100.0%)	121 (100.0%)	726 (100.0%)
	**Total Favorable**	106 (85.5%)	105 (88.2%)	106 (84.8%)	91 (75.2%)	95 (81.9%)	94 (77.7%)	597 (82.2%)
	95% CI	[78.0%; 91.2%]	[81.0%; 93.4%]	[77.3%; 90.6%]	[66.5%; 82.6%]	[73.7%; 88.4%]	[69.2%; 84.8%]	[79.3%; 84.9%]
	Difference from control	7.8%	10.5%	7.1%	-2.5%	4.2%	-	
	95% CI of the difference	[-1.9%; 17.5%]	[1.1%; 20.0%]	[-2.6%; 16.8%]	[-13.2%; 8.2%]	[-6.0%; 14.4%]		
**Table S3. all-DST mITT**	**Total in population**	118 (100.0%)	115 (100.0%)	122 (100.0%)	118 (100.0%)	108 (100.0%)	119 (100.0%)	700 (100.0%)
	**Total Favorable**	101 (85.6%)	102 (88.7%)	103 (84.4%)	88 (74.6%)	89 (82.4%)	92 (77.3%)	575 (82.1%)
	95% CI	[77.9%; 91.4%]	[81.4%; 93.8%]	[76.8%; 90.4%]	[65.7%; 82.1%]	[73.9%; 89.1%]	[68.7%; 84.5%]	[79.1%; 84.9%]
	Difference from control	8.3%	11.4%	7.1%	-2.7%	5.1%	-	
	95% CI of the difference	[-1.6%;18.1%]	[1.9; 20.9%]	[-2.8%; 17.0%]	[-13.6%; 8.1%]	[-5.3%; 15.5%]		

N°	W104 treatment outcome	9BLMZ	9BCLLfxZ	9BDLLfxZ	9DCLLfxZ	9DCMZ	Control	Total
**Table S4. Assessable reclassifying “LTFU” or “unfavorable because unassessable” to Week 73 outcome**	**Total in population**	98 (100.0%)	95 (100.0%)	103 (100.0%)	96 (100.0%)	96 (100.0%)	74 (100.0%)	562 (100.0%)
	**Total Favorable**	91 (92.9%)	90 (94.7%)	97 (94.2%)	79 (82.3%)	81 (84.4%)	69 (93.2%)	507 (90.2%)
	95% CI	[85.8%; 97.1%]	[88.1%; 98.3%]	[87.8%; 97.8%]	[73.2%; 89.3%]	[75.5%; 91.0%]	[84.9%; 97.8%]	[87.5%; 92.5%]
	Difference from control	-0.4%	1.5%	0.9%	-11.0%	-8.9%	-	
	95% CI of the difference	[-8.0%; 7.3%]	[-5.8%; 8.8%]	[-6.4% ; 8.2%]	[-20.5%; -0.1%]	[-18.1%; 0.4%]		

Note: Sensitivity analysis excluding participants from the control arm who received a shortened regimen, if the proportion exceeds 10% was not performed as the proportion of shortened regimen received in the control is 4.8% (6/126). Multiple imputation to impute missing data was not performed as missing values in the final multivariable model reduce the relevant analysis population by only 0.7% (<20%). Confidence intervals widths have not been adjusted for multiplicity and the intervals should not be used in place of hypothesis testing for secondary outcomes. They are only presented for precision purposes.

Table S29. Line Listing of Deaths in endTB Trial by Week 104 (Safety population)

N	endTB arm	Age Range	Sex	Preferred term	Causality according to investigators	Causality according to the sponsor
1	9BLMZ	30-39	M	*Pneumocystis jirovecii* pneumonia	Not related	Not related
2	9BLMZ	50-59	M	Disease progression	Not related	Not related
3	9BLMZ	50-59	F	Septic shock	Not related	Not related
4	9BCLLfxZ	60-69	M	Alcohol poisoning	Not related	Not related
5	9BDLLfxZ	30-39	F	Respiratory failure	Not related	Not related
6	9BDLLfxZ	70+	F	Respiratory failure	Not related	Not related
7	9BDLLfxZ	50-59	M	Acute abdomen and acute respiratory failure	Not related	Not related
8	9DCLLfxZ	40-49	M	Respiratory failure	Not related	Not related
9	9DCLLfxZ	20-29	M	Disease progression	Not related	Not related
10	9DCLLfxZ	60-69	M	Sepsis	Not related	Not related
11	9DCLLfxZ	50-59	M	Death (unknown cause)	Not related	Not related
12	9DCMZ	50-59	M	Sepsis	Not related	Not related
13	9DCMZ	30-39	M	Death (unknown cause)	Not related	Related
14	9DCMZ	50-59	M	Pancreatic carcinoma	Not related	Not related
15	Control	30-39	M	Alcohol poisoning	Not related	Not related
16	Control	40-49	M	Pneumonia	Not related	Not related
17	Control	50-59	M	Pancreatitis acute	Not related	Not related
18	Control	30-39	M	Cardiomyopathy alcoholic	Not related	Not related

Table S30. Number (%) of participants with adverse events (AEs) by Week 73 (Safety Population)

**Adverse events (AEs)**	**9BLMZ (N = 126)**	**9BCLLfxZ (N = 122)**	**9BDLLfxZ (N = 127)**	**9DCLLfxZ (N = 124)**	**9DCMZ (N = 120)**	**Control (N = 126)**	**Total (N = 745)**
Grade 3 or higher AE – no. (%)	69 (54.8%)	68 (55.7%)	78 (61.4%)	75 (60.5%)	72 (60.0%)	79 (62.7%)	441 (59.2%)
Risk difference from control (95% CI)	-7.9 (-20.1;4.2)	-7.0 (-19.2;5.2)	-1.3 (-13.2;10.7)	-2.2 (-14.3;9.8)	-2.7 (-14.9;9.5)	-	
Serious adverse events	18 (14.3%)	16 (13.1%)	20 (15.8%)	18 (14.5%)	20 (16.7%)	21 (16.7%)	113 (15.2%)
Risk difference from control (95% CI)	-2.4 (-11.3;6.5)	-3.6 (-12.4;5.3)	-0.9 (-10.0;8.2)	-2.2 (-11.1;6.8)	0.0 (-9.3;9.3)	-	
Death from any cause	3 (2.4%)	1 (0.8%)	3 (2.4%)	4 (3.2%)	2 (1.7%)	2 (1.6%)	15 (2.0%)
Risk difference from control (95% CI)	0.8 (-2.6;4.2)	-0.8 (-3.5;1.9)	0.8 (-2.7;4.2)	1.6 (-2.2;5.4)	0.1 (-3.1;3.2)	-	
Participants with at least one AESI	35 (27.8%)	33 (27.1%)	25 (19.7%)	33 (26.6%)	26 (21.7%)	26 (20.6%)	178 (23.9%)
Risk difference from control (95% CI)	7.1 (-3.4;17.7)	6.4 (-4.2;17.0)	-0.9 (-10.8;8.9)	6.0 (-4.5;16.5)	1.0 (-9.2;11.2)	-	

Note: Confidence intervals widths have not been adjusted for multiplicity and the intervals should not be used in place of hypothesis testing. They are only presented for precision purposes.

Table S31. Number (%) of participants experiencing adverse events (AEs) of grade 3 or higher by SOC and PT by Week 73 (Safety Population)

**Number (%) of participants experiencing adverse events (AEs) of grade 3 or higher by SOC and PT by Week 73 (Safety Population**							
Event<br>**System Organ Class (SOC)**<br>- Preferred Term (PT)	**9BLMZ (n = 126)**	**9BCLLfxZ (n = 122)**	**9BDLLfxZ (n = 127)**	**9DCLLfxZ (n = 124)**	**9DCMZ (n = 120)**	**Control (n = 126)**	**Total* (N = 745)**
**Investigations**	**43 (34.1%)**	**43 (35.2%)**	**39 (30.7%)**	**46 (37.1%)**	**50 (41.7%)**	**39 (31.0%)**	**260 (34.9%)**
- Creatinine renal clearance decreased	21 (16.7%)	23 (18.9%)	29 (22.8%)	37 (29.8%)	36 (30.0%)	23 (18.3%)	169 (22.7%)
- Alanine aminotransferase increased	14 (11.1%)	10 (8.2%)	5 (3.9%)	8 (6.5%)	7 (5.8%)	3 (2.4%)	47 (6.3%)
- Blood magnesium increased	6 (4.8%)	6 (4.9%)	4 (3.1%)	1 (0.8%)	5 (4.2%)	3 (2.4%)	25 (3.4%)
- Aspartate aminotransferase increased	4 (3.2%)	7 (5.7%)	4 (3.1%)	3 (2.4%)	3 (2.5%)	4 (3.2%)	25 (3.4%)
- Bilirubin conjugated increased	2 (1.6%)	3 (2.5%)	3 (2.4%)	1 (0.8%)	1 (0.8%)	4 (3.2%)	14 (1.9%)
- Lymphocyte count decreased	4 (3.2%)	1 (0.8%)	2 (1.6%)	0 (0.0%)	2 (1.7%)	1 (0.8%)	10 (1.3%)
- Electrocardiogram QT prolonged	0 (0.0%)	4 (3.3%)	0 (0.0%)	0 (0.0%)	5 (4.2%)	0 (0.0%)	9 (1.2%)
- Transaminases increased	1 (0.8%)	1 (0.8%)	1 (0.8%)	3 (2.4%)	3 (2.5%)	0 (0.0%)	9 (1.2%)
- Gamma-glutamyltransferase increased	1 (0.8%)	1 (0.8%)	0 (0.0%)	1 (0.8%)	1 (0.8%)	2 (1.6%)	6 (0.8%)
- Neutrophil count decreased	1 (0.8%)	0 (0.0%)	0 (0.0%)	0 (0.0%)	1 (0.8%)	2 (1.6%)	4 (0.5%)
- Blood bilirubin increased	0 (0.0%)	1 (0.8%)	1 (0.8%)	0 (0.0%)	1 (0.8%)	1 (0.8%)	4 (0.5%)
- Glycosylated haemoglobin increased	2 (1.6%)	1 (0.8%)	0 (0.0%)	0 (0.0%)	1 (0.8%)	0 (0.0%)	4 (0.5%)
- Hepatic enzyme increased	2 (1.6%)	1 (0.8%)	0 (0.0%)	0 (0.0%)	0 (0.0%)	0 (0.0%)	3 (0.4%)
- Blood potassium increased	0 (0.0%)	0 (0.0%)	0 (0.0%)	0 (0.0%)	2 (1.7%)	1 (0.8%)	3 (0.4%)
- White blood cell count decreased	0 (0.0%)	0 (0.0%)	0 (0.0%)	0 (0.0%)	1 (0.8%)	2 (1.6%)	3 (0.4%)
- Weight increased	0 (0.0%)	0 (0.0%)	0 (0.0%)	0 (0.0%)	0 (0.0%)	2 (1.6%)	2 (0.3%)
- Haemoglobin increased	2 (1.6%)	0 (0.0%)	0 (0.0%)	0 (0.0%)	0 (0.0%)	0 (0.0%)	2 (0.3%)
- Eosinophil count increased	0 (0.0%)	1 (0.8%)	0 (0.0%)	0 (0.0%)	0 (0.0%)	0 (0.0%)	1 (0.1%)
- Blood creatinine decreased	0 (0.0%)	0 (0.0%)	0 (0.0%)	0 (0.0%)	1 (0.8%)	0 (0.0%)	1 (0.1%)
- Blood creatinine increased	0 (0.0%)	0 (0.0%)	1 (0.8%)	0 (0.0%)	0 (0.0%)	0 (0.0%)	1 (0.1%)
- Blood calcium decreased	0 (0.0%)	1 (0.8%)	0 (0.0%)	0 (0.0%)	0 (0.0%)	0 (0.0%)	1 (0.1%)
- Electrocardiogram QRS complex prolonged	0 (0.0%)	0 (0.0%)	0 (0.0%)	0 (0.0%)	0 (0.0%)	1 (0.8%)	1 (0.1%)
- Blood uric acid increased	0 (0.0%)	1 (0.8%)	0 (0.0%)	0 (0.0%)	0 (0.0%)	0 (0.0%)	1 (0.1%)
- Blood urea increased	0 (0.0%)	0 (0.0%)	1 (0.8%)	0 (0.0%)	0 (0.0%)	0 (0.0%)	1 (0.1%)
- Psychiatric evaluation abnormal	0 (0.0%)	0 (0.0%)	0 (0.0%)	0 (0.0%)	1 (0.8%)	0 (0.0%)	1 (0.1%)
**Nervous system disorders**	**8 (6.3%)**	**11 (9.0%)**	**16 (12.6%)**	**11 (8.9%)**	**4 (3.3%)**	**14 (11.1%)**	**64 (8.6%)**
- Neuropathy peripheral	3 (2.4%)	5 (4.1%)	7 (5.5%)	2 (1.6%)	1 (0.8%)	6 (4.8%)	24 (3.2%)
- Paraesthesia	2 (1.6%)	3 (2.5%)	4 (3.1%)	4 (3.2%)	1 (0.8%)	6 (4.8%)	20 (2.7%)
- Areflexia	0 (0.0%)	0 (0.0%)	1 (0.8%)	2 (1.6%)	3 (2.5%)	1 (0.8%)	7 (0.9%)
- Polyneuropathy	1 (0.8%)	0 (0.0%)	2 (1.6%)	1 (0.8%)	2 (1.7%)	0 (0.0%)	6 (0.8%)

Number (%) of participants experiencing adverse events (AEs) of grade 3 or higher by SOC and PT by Week 73 (Safety Population<br><br>*Event*<br>**System Organ Class (SOC)**<br>- Preferred Term (PT)	**9BLMZ (n = 126)**	**9BCLLfxZ (n = 122)**	**9BDLLfxZ (n = 127)**	**9DCLLfxZ (n = 124)**	**9DCMZ (n = 120)**	**Control (n = 126)**	**Total* (N = 745)**
- Optic neuritis	0 (0.0%)	0 (0.0%)	0 (0.0%)	1 (0.8%)	0 (0.0%)	1 (0.8%)	2 (0.3%)
- Hyporeflexia	2 (1.6%)	0 (0.0%)	0 (0.0%)	0 (0.0%)	0 (0.0%)	0 (0.0%)	2 (0.3%)
- Headache	0 (0.0%)	1 (0.8%)	1 (0.8%)	0 (0.0%)	0 (0.0%)	0 (0.0%)	2 (0.3%)
- Sciatica	0 (0.0%)	0 (0.0%)	1 (0.8%)	0 (0.0%)	0 (0.0%)	0 (0.0%)	1 (0.1%)
- Facial paralysis	0 (0.0%)	0 (0.0%)	1 (0.8%)	0 (0.0%)	0 (0.0%)	0 (0.0%)	1 (0.1%)
- Idiopathic intracranial hypertension	0 (0.0%)	0 (0.0%)	0 (0.0%)	0 (0.0%)	0 (0.0%)	1 (0.8%)	1 (0.1%)
- Syncope	0 (0.0%)	0 (0.0%)	0 (0.0%)	1 (0.8%)	0 (0.0%)	0 (0.0%)	1 (0.1%)
- Epilepsy	0 (0.0%)	1 (0.8%)	0 (0.0%)	0 (0.0%)	0 (0.0%)	0 (0.0%)	1 (0.1%)
- Transient ischaemic attack	0 (0.0%)	1 (0.8%)	0 (0.0%)	0 (0.0%)	0 (0.0%)	0 (0.0%)	1 (0.1%)
**Metabolism and nutrition disorders**	**10 (7.9%)**	**6 (4.9%)**	**10 (7.9%)**	**8 (6.5%)**	**10 (8.3%)**	**11 (8.7%)**	**55 (7.4%)**
- Hypermagnesaemia	6 (4.8%)	3 (2.5%)	5 (3.9%)	3 (2.4%)	3 (2.5%)	7 (5.6%)	27 (3.6%)
- Hyperglycaemia	2 (1.6%)	2 (1.6%)	0 (0.0%)	2 (1.6%)	2 (1.7%)	1 (0.8%)	9 (1.2%)
- Hyperkalaemia	2 (1.6%)	0 (0.0%)	0 (0.0%)	2 (1.6%)	1 (0.8%)	1 (0.8%)	6 (0.8%)
- Hypoalbuminaemia	1 (0.8%)	1 (0.8%)	0 (0.0%)	0 (0.0%)	1 (0.8%)	0 (0.0%)	3 (0.4%)
- Hyperuricaemia	0 (0.0%)	0 (0.0%)	1 (0.8%)	0 (0.0%)	1 (0.8%)	0 (0.0%)	2 (0.3%)
- Obesity	0 (0.0%)	0 (0.0%)	0 (0.0%)	0 (0.0%)	1 (0.8%)	1 (0.8%)	2 (0.3%)
- Diabetes mellitus inadequate control	0 (0.0%)	0 (0.0%)	0 (0.0%)	0 (0.0%)	2 (1.7%)	0 (0.0%)	2 (0.3%)
- Hypomagnesaemia	0 (0.0%)	0 (0.0%)	1 (0.8%)	0 (0.0%)	0 (0.0%)	1 (0.8%)	2 (0.3%)
- Hypercalcaemia	0 (0.0%)	0 (0.0%)	1 (0.8%)	0 (0.0%)	0 (0.0%)	0 (0.0%)	1 (0.1%)
- Hypokalaemia	0 (0.0%)	0 (0.0%)	1 (0.8%)	0 (0.0%)	0 (0.0%)	0 (0.0%)	1 (0.1%)
- Hypertriglyceridaemia	0 (0.0%)	0 (0.0%)	0 (0.0%)	0 (0.0%)	1 (0.8%)	0 (0.0%)	1 (0.1%)
- Decreased appetite	0 (0.0%)	0 (0.0%)	1 (0.8%)	0 (0.0%)	0 (0.0%)	0 (0.0%)	1 (0.1%)
- Hyponatraemia	0 (0.0%)	0 (0.0%)	0 (0.0%)	1 (0.8%)	0 (0.0%)	0 (0.0%)	1 (0.1%)
- Hypocalcaemia	0 (0.0%)	0 (0.0%)	0 (0.0%)	1 (0.8%)	0 (0.0%)	0 (0.0%)	1 (0.1%)
**Eye disorders**	**4 (3.2%)**	**12 (9.8%)**	**10 (7.9%)**	**8 (6.5%)**	**7 (5.8%)**	**11 (8.7%)**	**52 (7.0%)**
- Visual acuity reduced	3 (2.4%)	6 (4.9%)	7 (5.5%)	4 (3.2%)	4 (3.3%)	7 (5.6%)	31 (4.2%)
- Visual impairment	0 (0.0%)	2 (1.6%)	3 (2.4%)	2 (1.6%)	2 (1.7%)	2 (1.6%)	11 (1.5%)
- Cataract	1 (0.8%)	1 (0.8%)	0 (0.0%)	1 (0.8%)	0 (0.0%)	2 (1.6%)	5 (0.7%)
- Refraction disorder	0 (0.0%)	1 (0.8%)	0 (0.0%)	1 (0.8%)	0 (0.0%)	0 (0.0%)	2 (0.3%)
- Retinopathy	0 (0.0%)	1 (0.8%)	0 (0.0%)	0 (0.0%)	1 (0.8%)	0 (0.0%)	2 (0.3%)
- Myopia	0 (0.0%)	0 (0.0%)	0 (0.0%)	0 (0.0%)	1 (0.8%)	0 (0.0%)	1 (0.1%)
- Presbyopia	0 (0.0%)	1 (0.8%)	0 (0.0%)	0 (0.0%)	0 (0.0%)	0 (0.0%)	1 (0.1%)
- Vision blurred	0 (0.0%)	0 (0.0%)	0 (0.0%)	0 (0.0%)	0 (0.0%)	1 (0.8%)	1 (0.1%)

Number (%) of participants experiencing adverse events (AEs) of grade 3 or higher by SOC and PT by Week 73 (Safety Population							
Event<br>**System Organ Class (SOC)**<br>- Preferred Term (PT)	**9BLMZ (n = 126)**	**9BCLLfxZ (n = 122)**	**9BDLLfxZ (n = 127)**	**9DCLLfxZ (n = 124)**	**9DCMZ (n = 120)**	**Control (n = 126)**	**Total* (N = 745)**
- Optic neuropathy	0 (0.0%)	1 (0.8%)	0 (0.0%)	0 (0.0%)	0 (0.0%)	0 (0.0%)	1 (0.1%)
- Hypermetropia	0 (0.0%)	1 (0.8%)	0 (0.0%)	0 (0.0%)	0 (0.0%)	0 (0.0%)	1 (0.1%)
- Eye haemorrhage	0 (0.0%)	1 (0.8%)	0 (0.0%)	0 (0.0%)	0 (0.0%)	0 (0.0%)	1 (0.1%)
**Blood and lymphatic system disorders**	**6 (4.8%)**	**8 (6.6%)**	**9 (7.1%)**	**13 (10.5%)**	**5 (4.2%)**	**10 (7.9%)**	**51 (6.8%)**
- Anaemia	4 (3.2%)	6 (4.9%)	9 (7.1%)	9 (7.3%)	3 (2.5%)	7 (5.6%)	38 (5.1%)
- Lymphopenia	1 (0.8%)	0 (0.0%)	0 (0.0%)	4 (3.2%)	1 (0.8%)	3 (2.4%)	9 (1.2%)
- Neutropenia	0 (0.0%)	1 (0.8%)	0 (0.0%)	1 (0.8%)	1 (0.8%)	1 (0.8%)	4 (0.5%)
- Leukopenia	0 (0.0%)	1 (0.8%)	0 (0.0%)	1 (0.8%)	0 (0.0%)	1 (0.8%)	3 (0.4%)
- Bicytopenia	0 (0.0%)	0 (0.0%)	0 (0.0%)	0 (0.0%)	1 (0.8%)	1 (0.8%)	2 (0.3%)
- Thrombocytopenia	1 (0.8%)	0 (0.0%)	0 (0.0%)	0 (0.0%)	0 (0.0%)	1 (0.8%)	2 (0.3%)
- Pancytopenia	0 (0.0%)	0 (0.0%)	0 (0.0%)	1 (0.8%)	0 (0.0%)	0 (0.0%)	1 (0.1%)
- Bone marrow failure	0 (0.0%)	1 (0.8%)	0 (0.0%)	0 (0.0%)	0 (0.0%)	0 (0.0%)	1 (0.1%)
- Leukocytosis	0 (0.0%)	0 (0.0%)	0 (0.0%)	0 (0.0%)	0 (0.0%)	1 (0.8%)	1 (0.1%)
- Myelosuppression	0 (0.0%)	0 (0.0%)	0 (0.0%)	1 (0.8%)	0 (0.0%)	0 (0.0%)	1 (0.1%)
**Ear and labyrinth disorders**	**10 (7.9%)**	**11 (9.0%)**	**6 (4.7%)**	**4 (3.2%)**	**9 (7.5%)**	**9 (7.1%)**	**49 (6.6%)**
- Hypoacusis	8 (6.3%)	9 (7.4%)	4 (3.1%)	4 (3.2%)	7 (5.8%)	8 (6.3%)	40 (5.4%)
- Deafness	2 (1.6%)	2 (1.6%)	1 (0.8%)	0 (0.0%)	2 (1.7%)	1 (0.8%)	8 (1.1%)
- Deafness unilateral	0 (0.0%)	0 (0.0%)	1 (0.8%)	0 (0.0%)	0 (0.0%)	0 (0.0%)	1 (0.1%)
- Tinnitus	0 (0.0%)	1 (0.8%)	0 (0.0%)	0 (0.0%)	0 (0.0%)	0 (0.0%)	1 (0.1%)
**Infections and infestations**	**12 (9.5%)**	**3 (2.5%)**	**6 (4.7%)**	**6 (4.8%)**	**6 (5.0%)**	**4 (3.2%)**	**37 (5.0%)**
- Pneumonia	4 (3.2%)	0 (0.0%)	3 (2.4%)	0 (0.0%)	1 (0.8%)	2 (1.6%)	10 (1.3%)
- COVID-19	2 (1.6%)	0 (0.0%)	0 (0.0%)	0 (0.0%)	1 (0.8%)	1 (0.8%)	4 (0.5%)
- Hepatitis A	1 (0.8%)	0 (0.0%)	0 (0.0%)	2 (1.6%)	0 (0.0%)	0 (0.0%)	3 (0.4%)
- COVID-19 pneumonia	0 (0.0%)	2 (1.6%)	0 (0.0%)	0 (0.0%)	1 (0.8%)	0 (0.0%)	3 (0.4%)
- Sepsis	0 (0.0%)	1 (0.8%)	0 (0.0%)	1 (0.8%)	1 (0.8%)	0 (0.0%)	3 (0.4%)
- Pneumocystis jirovecii pneumonia	1 (0.8%)	0 (0.0%)	0 (0.0%)	1 (0.8%)	0 (0.0%)	0 (0.0%)	2 (0.3%)
- Pneumonia bacterial	0 (0.0%)	0 (0.0%)	1 (0.8%)	0 (0.0%)	0 (0.0%)	0 (0.0%)	1 (0.1%)
- Infective glossitis	0 (0.0%)	0 (0.0%)	0 (0.0%)	1 (0.8%)	0 (0.0%)	0 (0.0%)	1 (0.1%)
- Pulmonary tuberculoma	0 (0.0%)	0 (0.0%)	1 (0.8%)	0 (0.0%)	0 (0.0%)	0 (0.0%)	1 (0.1%)
- Respiratory tract infection	0 (0.0%)	0 (0.0%)	1 (0.8%)	0 (0.0%)	0 (0.0%)	0 (0.0%)	1 (0.1%)
- Wound infection	1 (0.8%)	0 (0.0%)	0 (0.0%)	0 (0.0%)	0 (0.0%)	0 (0.0%)	1 (0.1%)
- Tonsillitis	0 (0.0%)	0 (0.0%)	0 (0.0%)	0 (0.0%)	0 (0.0%)	1 (0.8%)	1 (0.1%)
- Abscess soft tissue	0 (0.0%)	0 (0.0%)	0 (0.0%)	0 (0.0%)	1 (0.8%)	0 (0.0%)	1 (0.1%)

Number (%) of participants experiencing adverse events (AEs) of grade 3 or higher by SOC and PT by Week 73 (Safety Population							
Event<br>**System Organ Class (SOC)**<br>- Preferred Term (PT)	**9BLMZ (n = 126)**	**9BCLLfxZ (n = 122)**	**9BDLLfxZ (n = 127)**	**9DCLLfxZ (n = 124)**	**9DCMZ (n = 120)**	**Control (n = 126)**	**Total* (N = 745)**
- Pyelonephritis	0 (0.0%)	0 (0.0%)	0 (0.0%)	0 (0.0%)	1 (0.8%)	0 (0.0%)	1 (0.1%)
- Tuberculosis	1 (0.8%)	0 (0.0%)	0 (0.0%)	0 (0.0%)	0 (0.0%)	0 (0.0%)	1 (0.1%)
- Appendicitis	0 (0.0%)	0 (0.0%)	0 (0.0%)	0 (0.0%)	0 (0.0%)	1 (0.8%)	1 (0.1%)
- Gastroenteritis	0 (0.0%)	0 (0.0%)	0 (0.0%)	1 (0.8%)	0 (0.0%)	0 (0.0%)	1 (0.1%)
- Herpes zoster	0 (0.0%)	0 (0.0%)	0 (0.0%)	0 (0.0%)	1 (0.8%)	0 (0.0%)	1 (0.1%)
- Skin infection	1 (0.8%)	0 (0.0%)	0 (0.0%)	0 (0.0%)	0 (0.0%)	0 (0.0%)	1 (0.1%)
- HIV infection	0 (0.0%)	0 (0.0%)	0 (0.0%)	0 (0.0%)	0 (0.0%)	1 (0.8%)	1 (0.1%)
- Cellulitis	0 (0.0%)	0 (0.0%)	1 (0.8%)	0 (0.0%)	0 (0.0%)	0 (0.0%)	1 (0.1%)
- Septic shock	1 (0.8%)	0 (0.0%)	0 (0.0%)	0 (0.0%)	0 (0.0%)	0 (0.0%)	1 (0.1%)
- Pneumonia staphylococcal	0 (0.0%)	0 (0.0%)	1 (0.8%)	0 (0.0%)	0 (0.0%)	0 (0.0%)	1 (0.1%)
- Post procedural sepsis	0 (0.0%)	0 (0.0%)	0 (0.0%)	1 (0.8%)	0 (0.0%)	0 (0.0%)	1 (0.1%)
- Dengue fever	1 (0.8%)	0 (0.0%)	0 (0.0%)	0 (0.0%)	0 (0.0%)	0 (0.0%)	1 (0.1%)
**Hepatobiliary disorders**	**6 (4.8%)**	**4 (3.3%)**	**2 (1.6%)**	**7 (5.6%)**	**5 (4.2%)**	**3 (2.4%)**	**27 (3.6%)**
- Hepatotoxicity	5 (4.0%)	0 (0.0%)	1 (0.8%)	3 (2.4%)	1 (0.8%)	1 (0.8%)	11 (1.5%)
- Drug-induced liver injury	1 (0.8%)	3 (2.5%)	1 (0.8%)	3 (2.4%)	0 (0.0%)	0 (0.0%)	8 (1.1%)
- Hepatitis	0 (0.0%)	1 (0.8%)	0 (0.0%)	0 (0.0%)	2 (1.7%)	2 (1.6%)	5 (0.7%)
- Hyperbilirubinaemia	0 (0.0%)	0 (0.0%)	0 (0.0%)	1 (0.8%)	1 (0.8%)	0 (0.0%)	2 (0.3%)
- Acute on chronic liver failure	0 (0.0%)	0 (0.0%)	0 (0.0%)	0 (0.0%)	1 (0.8%)	0 (0.0%)	1 (0.1%)
**Musculoskeletal and connective tissue disorders**	**1 (0.8%)**	**6 (4.9%)**	**4 (3.1%)**	**4 (3.2%)**	**2 (1.7%)**	**5 (4.0%)**	**22 (3.0%)**
- Arthralgia	0 (0.0%)	4 (3.3%)	3 (2.4%)	2 (1.6%)	1 (0.8%)	5 (4.0%)	15 (2.0%)
- Myalgia	0 (0.0%)	2 (1.6%)	1 (0.8%)	0 (0.0%)	0 (0.0%)	1 (0.8%)	4 (0.5%)
- Back pain	1 (0.8%)	0 (0.0%)	0 (0.0%)	1 (0.8%)	0 (0.0%)	1 (0.8%)	3 (0.4%)
- Rotator cuff syndrome	0 (0.0%)	1 (0.8%)	0 (0.0%)	1 (0.8%)	0 (0.0%)	0 (0.0%)	2 (0.3%)
- Osteoarthritis	0 (0.0%)	0 (0.0%)	0 (0.0%)	0 (0.0%)	1 (0.8%)	0 (0.0%)	1 (0.1%)
- Arthropathy	0 (0.0%)	1 (0.8%)	0 (0.0%)	0 (0.0%)	0 (0.0%)	0 (0.0%)	1 (0.1%)
**Gastrointestinal disorders**	**1 (0.8%)**	**2 (1.6%)**	**3 (2.4%)**	**5 (4.0%)**	**2 (1.7%)**	**3 (2.4%)**	**16 (2.1%)**
- Vomiting	0 (0.0%)	1 (0.8%)	0 (0.0%)	3 (2.4%)	0 (0.0%)	3 (2.4%)	7 (0.9%)
- Nausea	0 (0.0%)	1 (0.8%)	2 (1.6%)	1 (0.8%)	1 (0.8%)	0 (0.0%)	5 (0.7%)
- Gastritis	0 (0.0%)	0 (0.0%)	0 (0.0%)	0 (0.0%)	1 (0.8%)	0 (0.0%)	1 (0.1%)
- Dyspepsia	0 (0.0%)	0 (0.0%)	1 (0.8%)	0 (0.0%)	0 (0.0%)	0 (0.0%)	1 (0.1%)
- Acute abdomen	0 (0.0%)	0 (0.0%)	1 (0.8%)	0 (0.0%)	0 (0.0%)	0 (0.0%)	1 (0.1%)
- Pancreatitis	0 (0.0%)	0 (0.0%)	0 (0.0%)	0 (0.0%)	0 (0.0%)	1 (0.8%)	1 (0.1%)
- Abdominal pain	0 (0.0%)	0 (0.0%)	0 (0.0%)	1 (0.8%)	0 (0.0%)	0 (0.0%)	1 (0.1%)

Number (%) of participants experiencing adverse events (AEs) of grade 3 or higher by SOC and PT by Week 73 (Safety Population<br><br>*Event*<br>**System Organ Class (SOC)**<br>- Preferred Term (PT)	**9BLMZ (n = 126)**	**9BCLLfxZ (n = 122)**	**9BDLLfxZ (n = 127)**	**9DCLLfxZ (n = 124)**	**9DCMZ (n = 120)**	**Control (n = 126)**	**Total* (N = 745)**
- Small intestinal obstruction	0 (0.0%)	0 (0.0%)	0 (0.0%)	1 (0.8%)	0 (0.0%)	0 (0.0%)	1 (0.1%)
- Upper gastrointestinal haemorrhage	0 (0.0%)	0 (0.0%)	0 (0.0%)	0 (0.0%)	1 (0.8%)	0 (0.0%)	1 (0.1%)
- Abdominal hernia	1 (0.8%)	0 (0.0%)	0 (0.0%)	0 (0.0%)	0 (0.0%)	0 (0.0%)	1 (0.1%)
**Respiratory, thoracic and mediastinal disorders**	**0 (0.0%)**	**2 (1.6%)**	**4 (3.1%)**	**2 (1.6%)**	**3 (2.5%)**	**4 (3.2%)**	**15 (2.0%)**
- Haemoptysis	0 (0.0%)	0 (0.0%)	0 (0.0%)	0 (0.0%)	1 (0.8%)	2 (1.6%)	3 (0.4%)
- Pneumothorax	0 (0.0%)	0 (0.0%)	0 (0.0%)	1 (0.8%)	1 (0.8%)	1 (0.8%)	3 (0.4%)
- Dyspnoea	0 (0.0%)	1 (0.8%)	1 (0.8%)	1 (0.8%)	0 (0.0%)	0 (0.0%)	3 (0.4%)
- Cough	0 (0.0%)	0 (0.0%)	1 (0.8%)	0 (0.0%)	2 (1.7%)	0 (0.0%)	3 (0.4%)
- Respiratory failure	0 (0.0%)	0 (0.0%)	1 (0.8%)	0 (0.0%)	0 (0.0%)	1 (0.8%)	2 (0.3%)
- Acute pulmonary oedema	0 (0.0%)	1 (0.8%)	0 (0.0%)	0 (0.0%)	0 (0.0%)	0 (0.0%)	1 (0.1%)
- Pulmonary cavitation	0 (0.0%)	0 (0.0%)	0 (0.0%)	0 (0.0%)	0 (0.0%)	1 (0.8%)	1 (0.1%)
- Acute respiratory failure	0 (0.0%)	0 (0.0%)	1 (0.8%)	0 (0.0%)	0 (0.0%)	0 (0.0%)	1 (0.1%)
**Skin and subcutaneous tissue disorders**	**1 (0.8%)**	**2 (1.6%)**	**2 (1.6%)**	**3 (2.4%)**	**3 (2.5%)**	**1 (0.8%)**	**12 (1.6%)**
- Rash	0 (0.0%)	1 (0.8%)	1 (0.8%)	2 (1.6%)	0 (0.0%)	0 (0.0%)	4 (0.5%)
- Pruritus	0 (0.0%)	1 (0.8%)	0 (0.0%)	0 (0.0%)	2 (1.7%)	0 (0.0%)	3 (0.4%)
- Drug reaction with eosinophilia and systemic symptoms	0 (0.0%)	0 (0.0%)	0 (0.0%)	1 (0.8%)	1 (0.8%)	0 (0.0%)	2 (0.3%)
- Dermatitis exfoliative	0 (0.0%)	0 (0.0%)	0 (0.0%)	0 (0.0%)	1 (0.8%)	0 (0.0%)	1 (0.1%)
- Eczema	0 (0.0%)	0 (0.0%)	0 (0.0%)	0 (0.0%)	0 (0.0%)	1 (0.8%)	1 (0.1%)
- Fixed eruption	1 (0.8%)	0 (0.0%)	0 (0.0%)	0 (0.0%)	0 (0.0%)	0 (0.0%)	1 (0.1%)
- Dry skin	0 (0.0%)	0 (0.0%)	1 (0.8%)	0 (0.0%)	0 (0.0%)	0 (0.0%)	1 (0.1%)
**General disorders and administration site conditions**	**1 (0.8%)**	**1 (0.8%)**	**2 (1.6%)**	**2 (1.6%)**	**2 (1.7%)**	**4 (3.2%)**	**12 (1.6%)**
- Fatigue	0 (0.0%)	1 (0.8%)	0 (0.0%)	0 (0.0%)	0 (0.0%)	2 (1.6%)	3 (0.4%)
- Chest pain	0 (0.0%)	0 (0.0%)	1 (0.8%)	0 (0.0%)	0 (0.0%)	1 (0.8%)	2 (0.3%)
- Malaise	0 (0.0%)	1 (0.8%)	0 (0.0%)	0 (0.0%)	0 (0.0%)	1 (0.8%)	2 (0.3%)
- Pyrexia	0 (0.0%)	0 (0.0%)	0 (0.0%)	0 (0.0%)	1 (0.8%)	1 (0.8%)	2 (0.3%)
- Death	0 (0.0%)	0 (0.0%)	0 (0.0%)	1 (0.8%)	0 (0.0%)	0 (0.0%)	1 (0.1%)
- Sudden death	0 (0.0%)	0 (0.0%)	0 (0.0%)	0 (0.0%)	1 (0.8%)	0 (0.0%)	1 (0.1%)
- Generalised oedema	1 (0.8%)	0 (0.0%)	0 (0.0%)	0 (0.0%)	0 (0.0%)	0 (0.0%)	1 (0.1%)
- Disease progression	0 (0.0%)	0 (0.0%)	0 (0.0%)	1 (0.8%)	0 (0.0%)	0 (0.0%)	1 (0.1%)
- Asthenia	0 (0.0%)	0 (0.0%)	0 (0.0%)	0 (0.0%)	0 (0.0%)	1 (0.8%)	1 (0.1%)
- Mucosal inflammation	0 (0.0%)	0 (0.0%)	1 (0.8%)	0 (0.0%)	0 (0.0%)	0 (0.0%)	1 (0.1%)
**Injury, poisoning and procedural complications**	**0 (0.0%)**	**1 (0.8%)**	**2 (1.6%)**	**2 (1.6%)**	**2 (1.7%)**	**4 (3.2%)**	**11 (1.5%)**
- Alcohol poisoning	0 (0.0%)	1 (0.8%)	0 (0.0%)	0 (0.0%)	0 (0.0%)	2 (1.6%)	3 (0.4%)

Number (%) of participants experiencing adverse events (AEs) of grade 3 or higher by SOC and PT by Week 73 (Safety Population							
Event<br>**System Organ Class (SOC)**<br>- Preferred Term (PT)	**9BLMZ (n = 126)**	**9BCLLfxZ (n = 122)**	**9BDLLfxZ (n = 127)**	**9DCLLfxZ (n = 124)**	**9DCMZ (n = 120)**	**Control (n = 126)**	**Total* (N = 745)**
- Femur fracture	0 (0.0%)	0 (0.0%)	0 (0.0%)	0 (0.0%)	1 (0.8%)	0 (0.0%)	1 (0.1%)
- Thermal burn	0 (0.0%)	0 (0.0%)	1 (0.8%)	0 (0.0%)	0 (0.0%)	0 (0.0%)	1 (0.1%)
- Abortion induced incomplete	0 (0.0%)	0 (0.0%)	1 (0.8%)	0 (0.0%)	0 (0.0%)	0 (0.0%)	1 (0.1%)
- Hip fracture	0 (0.0%)	0 (0.0%)	0 (0.0%)	0 (0.0%)	1 (0.8%)	0 (0.0%)	1 (0.1%)
- Ankle fracture	0 (0.0%)	0 (0.0%)	0 (0.0%)	0 (0.0%)	0 (0.0%)	1 (0.8%)	1 (0.1%)
- Stab wound	0 (0.0%)	0 (0.0%)	0 (0.0%)	0 (0.0%)	0 (0.0%)	1 (0.8%)	1 (0.1%)
- Thoracic vertebral fracture	0 (0.0%)	0 (0.0%)	0 (0.0%)	1 (0.8%)	0 (0.0%)	0 (0.0%)	1 (0.1%)
- Hand fracture	0 (0.0%)	0 (0.0%)	0 (0.0%)	1 (0.8%)	0 (0.0%)	0 (0.0%)	1 (0.1%)
**Psychiatric disorders**	**0 (0.0%)**	**2 (1.6%)**	**1 (0.8%)**	**1 (0.8%)**	**3 (2.5%)**	**3 (2.4%)**	**10 (1.3%)**
- Suicide attempt	0 (0.0%)	1 (0.8%)	0 (0.0%)	0 (0.0%)	3 (2.5%)	1 (0.8%)	5 (0.7%)
- Depression	0 (0.0%)	1 (0.8%)	0 (0.0%)	1 (0.8%)	0 (0.0%)	2 (1.6%)	4 (0.5%)
- Anxiety	0 (0.0%)	0 (0.0%)	0 (0.0%)	1 (0.8%)	0 (0.0%)	1 (0.8%)	2 (0.3%)
- Anxiety disorder	0 (0.0%)	0 (0.0%)	1 (0.8%)	0 (0.0%)	0 (0.0%)	0 (0.0%)	1 (0.1%)
- Drug dependence	0 (0.0%)	1 (0.8%)	0 (0.0%)	0 (0.0%)	0 (0.0%)	0 (0.0%)	1 (0.1%)
**Vascular disorders**	**1 (0.8%)**	**1 (0.8%)**	**1 (0.8%)**	**2 (1.6%)**	**0 (0.0%)**	**0 (0.0%)**	**5 (0.7%)**
- Hypotension	0 (0.0%)	0 (0.0%)	1 (0.8%)	1 (0.8%)	0 (0.0%)	0 (0.0%)	2 (0.3%)
- Diabetic vascular disorder	1 (0.8%)	0 (0.0%)	0 (0.0%)	0 (0.0%)	0 (0.0%)	0 (0.0%)	1 (0.1%)
- Hypertension	0 (0.0%)	1 (0.8%)	0 (0.0%)	0 (0.0%)	0 (0.0%)	0 (0.0%)	1 (0.1%)
- Deep vein thrombosis	0 (0.0%)	0 (0.0%)	0 (0.0%)	1 (0.8%)	0 (0.0%)	0 (0.0%)	1 (0.1%)
**Cardiac disorders**	**1 (0.8%)**	**2 (1.6%)**	**0 (0.0%)**	**2 (1.6%)**	**0 (0.0%)**	**0 (0.0%)**	**5 (0.7%)**
- Acute myocardial infarction	0 (0.0%)	1 (0.8%)	0 (0.0%)	2 (1.6%)	0 (0.0%)	0 (0.0%)	3 (0.4%)
- Cardiac failure congestive	1 (0.8%)	1 (0.8%)	0 (0.0%)	0 (0.0%)	0 (0.0%)	0 (0.0%)	2 (0.3%)
- Cardiac failure	0 (0.0%)	1 (0.8%)	0 (0.0%)	1 (0.8%)	0 (0.0%)	0 (0.0%)	2 (0.3%)
**Renal and urinary disorders**	**1 (0.8%)**	**0 (0.0%)**	**1 (0.8%)**	**0 (0.0%)**	**0 (0.0%)**	**1 (0.8%)**	**3 (0.4%)**
- Proteinuria	1 (0.8%)	0 (0.0%)	1 (0.8%)	0 (0.0%)	0 (0.0%)	0 (0.0%)	2 (0.3%)
- Chronic kidney disease	0 (0.0%)	0 (0.0%)	0 (0.0%)	0 (0.0%)	0 (0.0%)	1 (0.8%)	1 (0.1%)
- Acute kidney injury	1 (0.8%)	0 (0.0%)	0 (0.0%)	0 (0.0%)	0 (0.0%)	0 (0.0%)	1 (0.1%)
**Immune system disorders**	**0 (0.0%)**	**0 (0.0%)**	**2 (1.6%)**	**0 (0.0%)**	**0 (0.0%)**	**0 (0.0%)**	**2 (0.3%)**
- Hypersensitivity	0 (0.0%)	0 (0.0%)	2 (1.6%)	0 (0.0%)	0 (0.0%)	0 (0.0%)	2 (0.3%)
**Pregnancy, puerperium and perinatal conditions**	**0 (0.0%)**	**0 (0.0%)**	**1 (0.8%)**	**0 (0.0%)**	**0 (0.0%)**	**1 (0.8%)**	**2 (0.3%)**
- Abortion threatened	0 (0.0%)	0 (0.0%)	1 (0.8%)	0 (0.0%)	0 (0.0%)	0 (0.0%)	1 (0.1%)
- Abortion spontaneous	0 (0.0%)	0 (0.0%)	0 (0.0%)	0 (0.0%)	0 (0.0%)	1 (0.8%)	1 (0.1%)

Number (%) of participants experiencing adverse events (AEs) of grade 3 or higher by SOC and PT by Week 73 (Safety Population							
Event<br>**System Organ Class (SOC)**<br>- Preferred Term (PT)	**9BLMZ (n = 126)**	**9BCLLfxZ (n = 122)**	**9BDLLfxZ (n = 127)**	**9DCLLfxZ (n = 124)**	**9DCMZ (n = 120)**	**Control (n = 126)**	**Total* (N = 745)**
**Neoplasms benign, malignant and unspecified (incl cysts and polyps)**	**0 (0.0%)**	**0 (0.0%)**	**0 (0.0%)**	**0 (0.0%)**	**0 (0.0%)**	**1 (0.8%)**	**1 (0.1%)**
- Lung neoplasm malignant	0 (0.0%)	0 (0.0%)	0 (0.0%)	0 (0.0%)	0 (0.0%)	1 (0.8%)	1 (0.1%)

**Total by preferred term (PT) represents the sum of participants who experienced at least one event corresponding to that PT. If they experienced more than one event of the same PT, it is counted only once. If a participant experienced more than one unique PT within a system organ class (SOC), each unique PT counts toward the PT total, but only one event counts toward the SOC.*

Table S32. Number (%) of participants experiencing adverse events (AEs) of grade 3 or higher by SOC and PT by Week 104 (Safety Population)

Number (%) of participants experiencing adverse events (AEs) of grade 3 or higher by SOC and PT by Week 104 (Safety Population)							
Event<br>**System Organ Class (SOC)**<br>- Preferred Term (PT)	**9BLMZ (n = 126)**	**9BCLLfxZ (n = 122)**	**9BDLLfxZ (n = 127)**	**9DCLLfxZ (n = 124)**	**9DCMZ (n = 120)**	**Control (n = 126)**	**Total* (N = 745)**
**Investigations**	**43 (34.1%)**	**47 (38.5%)**	**43 (33.9%)**	**47 (37.9%)**	**51 (42.5%)**	**40 (31.7%)**	**271 (36.4%)**
- Creatinine renal clearance decreased	21 (16.7%)	24 (19.7%)	30 (23.6%)	37 (29.8%)	37 (30.8%)	23 (18.3%)	172 (23.1%)
- Alanine aminotransferase increased	14 (11.1%)	10 (8.2%)	5 (3.9%)	8 (6.5%)	7 (5.8%)	3 (2.4%)	47 (6.3%)
- Aspartate aminotransferase increased	4 (3.2%)	7 (5.7%)	5 (3.9%)	3 (2.4%)	3 (2.5%)	6 (4.8%)	28 (3.8%)
- Blood magnesium increased	6 (4.8%)	6 (4.9%)	4 (3.1%)	1 (0.8%)	5 (4.2%)	3 (2.4%)	25 (3.4%)
- Bilirubin conjugated increased	2 (1.6%)	3 (2.5%)	3 (2.4%)	1 (0.8%)	1 (0.8%)	4 (3.2%)	14 (1.9%)
- Transaminases increased	1 (0.8%)	2 (1.6%)	2 (1.6%)	3 (2.4%)	3 (2.5%)	0 (0.0%)	11 (1.5%)
- Lymphocyte count decreased	4 (3.2%)	1 (0.8%)	2 (1.6%)	0 (0.0%)	2 (1.7%)	1 (0.8%)	10 (1.3%)
- Electrocardiogram QT prolonged	0 (0.0%)	4 (3.3%)	0 (0.0%)	0 (0.0%)	5 (4.2%)	0 (0.0%)	9 (1.2%)
- Gamma-glutamyltransferase increased	1 (0.8%)	1 (0.8%)	1 (0.8%)	1 (0.8%)	1 (0.8%)	2 (1.6%)	7 (0.9%)
- Glycosylated haemoglobin increased	2 (1.6%)	2 (1.6%)	0 (0.0%)	1 (0.8%)	1 (0.8%)	0 (0.0%)	6 (0.8%)
- Neutrophil count decreased	1 (0.8%)	1 (0.8%)	0 (0.0%)	0 (0.0%)	1 (0.8%)	3 (2.4%)	6 (0.8%)
- Blood bilirubin increased	0 (0.0%)	1 (0.8%)	1 (0.8%)	0 (0.0%)	1 (0.8%)	1 (0.8%)	4 (0.5%)
- Hepatic enzyme increased	2 (1.6%)	1 (0.8%)	1 (0.8%)	0 (0.0%)	0 (0.0%)	0 (0.0%)	4 (0.5%)
- Weight increased	0 (0.0%)	0 (0.0%)	1 (0.8%)	0 (0.0%)	0 (0.0%)	2 (1.6%)	3 (0.4%)
- White blood cell count decreased	0 (0.0%)	0 (0.0%)	0 (0.0%)	0 (0.0%)	1 (0.8%)	2 (1.6%)	3 (0.4%)
- Blood potassium increased	0 (0.0%)	0 (0.0%)	0 (0.0%)	0 (0.0%)	2 (1.7%)	1 (0.8%)	3 (0.4%)
- Haemoglobin increased	2 (1.6%)	0 (0.0%)	0 (0.0%)	0 (0.0%)	0 (0.0%)	0 (0.0%)	2 (0.3%)

Number (%) of participants experiencing adverse events (AEs) of grade 3 or higher by SOC and PT by Week 104 (Safety Population)							
Event<br>**System Organ Class (SOC)**<br>- Preferred Term (PT)	**9BLMZ (n = 126)**	**9BCLLfxZ (n = 122)**	**9BDLLfxZ (n = 127)**	**9DCLLfxZ (n = 124)**	**9DCMZ (n = 120)**	**Control (n = 126)**	**Total* (N = 745)**
- Blood uric acid increased	0 (0.0%)	1 (0.8%)	0 (0.0%)	0 (0.0%)	0 (0.0%)	0 (0.0%)	1 (0.1%)
- Eosinophil count increased	0 (0.0%)	1 (0.8%)	0 (0.0%)	0 (0.0%)	0 (0.0%)	0 (0.0%)	1 (0.1%)
- Eastern Cooperative Oncology Group performance status worsened	0 (0.0%)	0 (0.0%)	0 (0.0%)	1 (0.8%)	0 (0.0%)	0 (0.0%)	1 (0.1%)
- Blood creatinine increased	0 (0.0%)	0 (0.0%)	1 (0.8%)	0 (0.0%)	0 (0.0%)	0 (0.0%)	1 (0.1%)
- Blood creatinine decreased	0 (0.0%)	0 (0.0%)	0 (0.0%)	0 (0.0%)	1 (0.8%)	0 (0.0%)	1 (0.1%)
- Psychiatric evaluation abnormal	0 (0.0%)	0 (0.0%)	0 (0.0%)	0 (0.0%)	1 (0.8%)	0 (0.0%)	1 (0.1%)
- Blood urea increased	0 (0.0%)	0 (0.0%)	1 (0.8%)	0 (0.0%)	0 (0.0%)	0 (0.0%)	1 (0.1%)
- Electrocardiogram QRS complex prolonged	0 (0.0%)	0 (0.0%)	0 (0.0%)	0 (0.0%)	0 (0.0%)	1 (0.8%)	1 (0.1%)
- Blood calcium decreased	0 (0.0%)	1 (0.8%)	0 (0.0%)	0 (0.0%)	0 (0.0%)	0 (0.0%)	1 (0.1%)
**Nervous system disorders**	**8 (6.3%)**	**11 (9.0%)**	**17 (13.4%)**	**13 (10.5%)**	**4 (3.3%)**	**15 (11.9%)**	**68 (9.1%)**
- Neuropathy peripheral	3 (2.4%)	5 (4.1%)	7 (5.5%)	2 (1.6%)	1 (0.8%)	6 (4.8%)	24 (3.2%)
- Paraesthesia	2 (1.6%)	3 (2.5%)	4 (3.1%)	4 (3.2%)	1 (0.8%)	7 (5.6%)	21 (2.8%)
- Areflexia	0 (0.0%)	0 (0.0%)	1 (0.8%)	2 (1.6%)	3 (2.5%)	1 (0.8%)	7 (0.9%)
- Polyneuropathy	1 (0.8%)	0 (0.0%)	2 (1.6%)	1 (0.8%)	2 (1.7%)	0 (0.0%)	6 (0.8%)
- Hyporeflexia	2 (1.6%)	0 (0.0%)	1 (0.8%)	0 (0.0%)	0 (0.0%)	0 (0.0%)	3 (0.4%)
- Transient ischaemic attack	0 (0.0%)	1 (0.8%)	0 (0.0%)	1 (0.8%)	0 (0.0%)	0 (0.0%)	2 (0.3%)
- Headache	0 (0.0%)	1 (0.8%)	1 (0.8%)	0 (0.0%)	0 (0.0%)	0 (0.0%)	2 (0.3%)
- Optic neuritis	0 (0.0%)	0 (0.0%)	0 (0.0%)	1 (0.8%)	0 (0.0%)	1 (0.8%)	2 (0.3%)
- Facial paralysis	0 (0.0%)	0 (0.0%)	1 (0.8%)	0 (0.0%)	0 (0.0%)	0 (0.0%)	1 (0.1%)
- Sciatica	0 (0.0%)	0 (0.0%)	1 (0.8%)	0 (0.0%)	0 (0.0%)	0 (0.0%)	1 (0.1%)
- Syncope	0 (0.0%)	0 (0.0%)	0 (0.0%)	1 (0.8%)	0 (0.0%)	0 (0.0%)	1 (0.1%)
- Brain injury	0 (0.0%)	0 (0.0%)	0 (0.0%)	1 (0.8%)	0 (0.0%)	0 (0.0%)	1 (0.1%)
- Idiopathic intracranial hypertension	0 (0.0%)	0 (0.0%)	0 (0.0%)	0 (0.0%)	0 (0.0%)	1 (0.8%)	1 (0.1%)
- Epilepsy	0 (0.0%)	1 (0.8%)	0 (0.0%)	0 (0.0%)	0 (0.0%)	0 (0.0%)	1 (0.1%)
**Metabolism and nutrition disorders**	**11 (8.7%)**	**7 (5.7%)**	**11 (8.7%)**	**10 (8.1%)**	**10 (8.3%)**	**13 (10.3%)**	**62 (8.3%)**
- Hypermagnesaemia	7 (5.6%)	3 (2.5%)	5 (3.9%)	3 (2.4%)	3 (2.5%)	8 (6.3%)	29 (3.9%)
- Hyperglycaemia	2 (1.6%)	2 (1.6%)	0 (0.0%)	3 (2.4%)	2 (1.7%)	1 (0.8%)	10 (1.3%)
- Hyperkalaemia	2 (1.6%)	0 (0.0%)	0 (0.0%)	2 (1.6%)	1 (0.8%)	1 (0.8%)	6 (0.8%)
- Hypocalcaemia	0 (0.0%)	1 (0.8%)	0 (0.0%)	2 (1.6%)	0 (0.0%)	1 (0.8%)	4 (0.5%)
- Diabetes mellitus inadequate control	0 (0.0%)	0 (0.0%)	1 (0.8%)	0 (0.0%)	2 (1.7%)	0 (0.0%)	3 (0.4%)
- Hypoalbuminaemia	1 (0.8%)	1 (0.8%)	0 (0.0%)	0 (0.0%)	1 (0.8%)	0 (0.0%)	3 (0.4%)
- Hyperuricaemia	0 (0.0%)	0 (0.0%)	1 (0.8%)	0 (0.0%)	1 (0.8%)	0 (0.0%)	2 (0.3%)

Number (%) of participants experiencing adverse events (AEs) of grade 3 or higher by SOC and PT by Week 104 (Safety Population)							
Event<br>**System Organ Class (SOC)**<br>- Preferred Term (PT)	**9BLMZ (n = 126)**	**9BCLLfxZ (n = 122)**	**9BDLLfxZ (n = 127)**	**9DCLLfxZ (n = 124)**	**9DCMZ (n = 120)**	**Control (n = 126)**	**Total* (N = 745)**
- Hypomagnesaemia	0 (0.0%)	0 (0.0%)	1 (0.8%)	0 (0.0%)	0 (0.0%)	1 (0.8%)	2 (0.3%)
- Obesity	0 (0.0%)	0 (0.0%)	0 (0.0%)	0 (0.0%)	1 (0.8%)	1 (0.8%)	2 (0.3%)
- Hyponatraemia	0 (0.0%)	0 (0.0%)	0 (0.0%)	1 (0.8%)	0 (0.0%)	0 (0.0%)	1 (0.1%)
- Hypokalaemia	0 (0.0%)	0 (0.0%)	1 (0.8%)	0 (0.0%)	0 (0.0%)	0 (0.0%)	1 (0.1%)
- Decreased appetite	0 (0.0%)	0 (0.0%)	1 (0.8%)	0 (0.0%)	0 (0.0%)	0 (0.0%)	1 (0.1%)
- Hypercalcaemia	0 (0.0%)	0 (0.0%)	1 (0.8%)	0 (0.0%)	0 (0.0%)	0 (0.0%)	1 (0.1%)
- Hypertriglyceridaemia	0 (0.0%)	0 (0.0%)	0 (0.0%)	0 (0.0%)	1 (0.8%)	0 (0.0%)	1 (0.1%)
**Eye disorders**	**5 (4.0%)**	**13 (10.7%)**	**10 (7.9%)**	**10 (8.1%)**	**8 (6.7%)**	**12 (9.5%)**	**58 (7.8%)**
- Visual acuity reduced	4 (3.2%)	7 (5.7%)	7 (5.5%)	5 (4.0%)	5 (4.2%)	7 (5.6%)	35 (4.7%)
- Visual impairment	0 (0.0%)	2 (1.6%)	3 (2.4%)	2 (1.6%)	2 (1.7%)	2 (1.6%)	11 (1.5%)
- Cataract	1 (0.8%)	1 (0.8%)	0 (0.0%)	1 (0.8%)	0 (0.0%)	2 (1.6%)	5 (0.7%)
- Refraction disorder	0 (0.0%)	1 (0.8%)	0 (0.0%)	1 (0.8%)	0 (0.0%)	0 (0.0%)	2 (0.3%)
- Retinopathy	0 (0.0%)	1 (0.8%)	0 (0.0%)	0 (0.0%)	1 (0.8%)	0 (0.0%)	2 (0.3%)
- Hypermetropia	0 (0.0%)	1 (0.8%)	0 (0.0%)	0 (0.0%)	0 (0.0%)	0 (0.0%)	1 (0.1%)
- Myopia	0 (0.0%)	0 (0.0%)	0 (0.0%)	0 (0.0%)	1 (0.8%)	0 (0.0%)	1 (0.1%)
- Cataract subcapsular	0 (0.0%)	0 (0.0%)	0 (0.0%)	0 (0.0%)	0 (0.0%)	1 (0.8%)	1 (0.1%)
- Vision blurred	0 (0.0%)	0 (0.0%)	0 (0.0%)	0 (0.0%)	0 (0.0%)	1 (0.8%)	1 (0.1%)
- Optic neuropathy	0 (0.0%)	1 (0.8%)	0 (0.0%)	0 (0.0%)	0 (0.0%)	0 (0.0%)	1 (0.1%)
- Visual field defect	0 (0.0%)	0 (0.0%)	0 (0.0%)	1 (0.8%)	0 (0.0%)	0 (0.0%)	1 (0.1%)
- Presbyopia	0 (0.0%)	1 (0.8%)	0 (0.0%)	0 (0.0%)	0 (0.0%)	0 (0.0%)	1 (0.1%)
- Eye haemorrhage	0 (0.0%)	1 (0.8%)	0 (0.0%)	0 (0.0%)	0 (0.0%)	0 (0.0%)	1 (0.1%)
**Ear and labyrinth disorders**	**10 (7.9%)**	**14 (11.5%)**	**6 (4.7%)**	**4 (3.2%)**	**11 (9.2%)**	**9 (7.1%)**	**54 (7.2%)**
- Hypoacusis	8 (6.3%)	12 (9.8%)	4 (3.1%)	4 (3.2%)	9 (7.5%)	8 (6.3%)	45 (6.0%)
- Deafness	2 (1.6%)	2 (1.6%)	1 (0.8%)	0 (0.0%)	2 (1.7%)	1 (0.8%)	8 (1.1%)
- Deafness unilateral	0 (0.0%)	0 (0.0%)	1 (0.8%)	0 (0.0%)	0 (0.0%)	0 (0.0%)	1 (0.1%)
- Tinnitus	0 (0.0%)	1 (0.8%)	0 (0.0%)	0 (0.0%)	0 (0.0%)	0 (0.0%)	1 (0.1%)
**Blood and lymphatic system disorders**	**6 (4.8%)**	**8 (6.6%)**	**9 (7.1%)**	**13 (10.5%)**	**5 (4.2%)**	**10 (7.9%)**	**51 (6.8%)**
- Anaemia	4 (3.2%)	6 (4.9%)	9 (7.1%)	9 (7.3%)	3 (2.5%)	7 (5.6%)	38 (5.1%)
- Lymphopenia	1 (0.8%)	0 (0.0%)	0 (0.0%)	4 (3.2%)	1 (0.8%)	3 (2.4%)	9 (1.2%)
- Neutropenia	0 (0.0%)	1 (0.8%)	0 (0.0%)	0 (0.0%)	1 (0.8%)	1 (0.8%)	3 (0.4%)
- Leukopenia	0 (0.0%)	1 (0.8%)	0 (0.0%)	0 (0.0%)	0 (0.0%)	1 (0.8%)	2 (0.3%)
- Bicytopenia	0 (0.0%)	0 (0.0%)	0 (0.0%)	0 (0.0%)	1 (0.8%)	1 (0.8%)	2 (0.3%)
- Thrombocytopenia	1 (0.8%)	0 (0.0%)	0 (0.0%)	0 (0.0%)	0 (0.0%)	1 (0.8%)	2 (0.3%)

Number (%) of participants experiencing adverse events (AEs) of grade 3 or higher by SOC and PT by Week 104 (Safety Population)							
Event<br>**System Organ Class (SOC)**<br>- Preferred Term (PT)	**9BLMZ (n = 126)**	**9BCLLfxZ (n = 122)**	**9BDLLfxZ (n = 127)**	**9DCLLfxZ (n = 124)**	**9DCMZ (n = 120)**	**Control (n = 126)**	**Total* (N = 745)**
- Pancytopenia	0 (0.0%)	0 (0.0%)	0 (0.0%)	1 (0.8%)	0 (0.0%)	0 (0.0%)	1 (0.1%)
- Leukocytosis	0 (0.0%)	0 (0.0%)	0 (0.0%)	0 (0.0%)	0 (0.0%)	1 (0.8%)	1 (0.1%)
- Bone marrow failure	0 (0.0%)	1 (0.8%)	0 (0.0%)	0 (0.0%)	0 (0.0%)	0 (0.0%)	1 (0.1%)
- Myelosuppression	0 (0.0%)	0 (0.0%)	0 (0.0%)	1 (0.8%)	0 (0.0%)	0 (0.0%)	1 (0.1%)
**Infections and infestations**	**13 (10.3%)**	**6 (4.9%)**	**6 (4.7%)**	**6 (4.8%)**	**7 (5.8%)**	**4 (3.2%)**	**42 (5.6%)**
- Pneumonia	4 (3.2%)	1 (0.8%)	3 (2.4%)	0 (0.0%)	1 (0.8%)	2 (1.6%)	11 (1.5%)
- COVID-19	2 (1.6%)	0 (0.0%)	0 (0.0%)	0 (0.0%)	2 (1.7%)	1 (0.8%)	5 (0.7%)
- COVID-19 pneumonia	0 (0.0%)	4 (3.3%)	0 (0.0%)	0 (0.0%)	1 (0.8%)	0 (0.0%)	5 (0.7%)
- Sepsis	0 (0.0%)	1 (0.8%)	0 (0.0%)	1 (0.8%)	1 (0.8%)	0 (0.0%)	3 (0.4%)
- Hepatitis A	1 (0.8%)	0 (0.0%)	0 (0.0%)	2 (1.6%)	0 (0.0%)	0 (0.0%)	3 (0.4%)
- Appendicitis	1 (0.8%)	0 (0.0%)	0 (0.0%)	0 (0.0%)	0 (0.0%)	1 (0.8%)	2 (0.3%)
- Pneumocystis jirovecii pneumonia	1 (0.8%)	0 (0.0%)	0 (0.0%)	1 (0.8%)	0 (0.0%)	0 (0.0%)	2 (0.3%)
- Post procedural sepsis	0 (0.0%)	0 (0.0%)	0 (0.0%)	1 (0.8%)	0 (0.0%)	0 (0.0%)	1 (0.1%)
- Pyelonephritis	0 (0.0%)	0 (0.0%)	0 (0.0%)	0 (0.0%)	1 (0.8%)	0 (0.0%)	1 (0.1%)
- Diabetic foot infection	1 (0.8%)	0 (0.0%)	0 (0.0%)	0 (0.0%)	0 (0.0%)	0 (0.0%)	1 (0.1%)
- Osteomyelitis	1 (0.8%)	0 (0.0%)	0 (0.0%)	0 (0.0%)	0 (0.0%)	0 (0.0%)	1 (0.1%)
- Infective glossitis	0 (0.0%)	0 (0.0%)	0 (0.0%)	1 (0.8%)	0 (0.0%)	0 (0.0%)	1 (0.1%)
- Tonsillitis	0 (0.0%)	0 (0.0%)	0 (0.0%)	0 (0.0%)	0 (0.0%)	1 (0.8%)	1 (0.1%)
- Herpes zoster	0 (0.0%)	0 (0.0%)	0 (0.0%)	0 (0.0%)	1 (0.8%)	0 (0.0%)	1 (0.1%)
- Septic shock	1 (0.8%)	0 (0.0%)	0 (0.0%)	0 (0.0%)	0 (0.0%)	0 (0.0%)	1 (0.1%)
- Pulmonary tuberculoma	0 (0.0%)	0 (0.0%)	1 (0.8%)	0 (0.0%)	0 (0.0%)	0 (0.0%)	1 (0.1%)
- HIV infection	0 (0.0%)	0 (0.0%)	0 (0.0%)	0 (0.0%)	0 (0.0%)	1 (0.8%)	1 (0.1%)
- Dengue fever	1 (0.8%)	0 (0.0%)	0 (0.0%)	0 (0.0%)	0 (0.0%)	0 (0.0%)	1 (0.1%)
- Pneumonia bacterial	0 (0.0%)	0 (0.0%)	1 (0.8%)	0 (0.0%)	0 (0.0%)	0 (0.0%)	1 (0.1%)
- Cellulitis	0 (0.0%)	0 (0.0%)	1 (0.8%)	0 (0.0%)	0 (0.0%)	0 (0.0%)	1 (0.1%)
- Pneumonia staphylococcal	0 (0.0%)	0 (0.0%)	1 (0.8%)	0 (0.0%)	0 (0.0%)	0 (0.0%)	1 (0.1%)
- Respiratory tract infection	0 (0.0%)	0 (0.0%)	1 (0.8%)	0 (0.0%)	0 (0.0%)	0 (0.0%)	1 (0.1%)
- Tuberculosis	1 (0.8%)	0 (0.0%)	0 (0.0%)	0 (0.0%)	0 (0.0%)	0 (0.0%)	1 (0.1%)
- Skin infection	1 (0.8%)	0 (0.0%)	0 (0.0%)	0 (0.0%)	0 (0.0%)	0 (0.0%)	1 (0.1%)
- Abscess soft tissue	0 (0.0%)	0 (0.0%)	0 (0.0%)	0 (0.0%)	1 (0.8%)	0 (0.0%)	1 (0.1%)
- Wound infection	1 (0.8%)	0 (0.0%)	0 (0.0%)	0 (0.0%)	0 (0.0%)	0 (0.0%)	1 (0.1%)
- Gastroenteritis	0 (0.0%)	0 (0.0%)	0 (0.0%)	1 (0.8%)	0 (0.0%)	0 (0.0%)	1 (0.1%)
**Hepatobiliary disorders**	**6 (4.8%)**	**5 (4.1%)**	**2 (1.6%)**	**7 (5.6%)**	**5 (4.2%)**	**3 (2.4%)**	**28 (3.8%)**

Number (%) of participants experiencing adverse events (AEs) of grade 3 or higher by SOC and PT by Week 104 (Safety Population)							
Event<br>**System Organ Class (SOC)**<br>- Preferred Term (PT)	**9BLMZ (n = 126)**	**9BCLLfxZ (n = 122)**	**9BDLLfxZ (n = 127)**	**9DCLLfxZ (n = 124)**	**9DCMZ (n = 120)**	**Control (n = 126)**	**Total* (N = 745)**
- Hepatotoxicity	5 (4.0%)	1 (0.8%)	1 (0.8%)	3 (2.4%)	1 (0.8%)	1 (0.8%)	12 (1.6%)
- Drug-induced liver injury	1 (0.8%)	3 (2.5%)	1 (0.8%)	3 (2.4%)	0 (0.0%)	0 (0.0%)	8 (1.1%)
- Hepatitis	0 (0.0%)	1 (0.8%)	0 (0.0%)	0 (0.0%)	2 (1.7%)	2 (1.6%)	5 (0.7%)
- Hyperbilirubinaemia	0 (0.0%)	0 (0.0%)	0 (0.0%)	1 (0.8%)	1 (0.8%)	0 (0.0%)	2 (0.3%)
- Acute on chronic liver failure	0 (0.0%)	0 (0.0%)	0 (0.0%)	0 (0.0%)	1 (0.8%)	0 (0.0%)	1 (0.1%)
**Musculoskeletal and connective tissue disorders**	**1 (0.8%)**	**6 (4.9%)**	**4 (3.1%)**	**4 (3.2%)**	**2 (1.7%)**	**5 (4.0%)**	**22 (3.0%)**
- Arthralgia	0 (0.0%)	4 (3.3%)	3 (2.4%)	2 (1.6%)	1 (0.8%)	5 (4.0%)	15 (2.0%)
- Myalgia	0 (0.0%)	2 (1.6%)	1 (0.8%)	0 (0.0%)	0 (0.0%)	1 (0.8%)	4 (0.5%)
- Back pain	1 (0.8%)	0 (0.0%)	0 (0.0%)	1 (0.8%)	0 (0.0%)	1 (0.8%)	3 (0.4%)
- Rotator cuff syndrome	0 (0.0%)	1 (0.8%)	0 (0.0%)	1 (0.8%)	0 (0.0%)	0 (0.0%)	2 (0.3%)
- Osteoarthritis	0 (0.0%)	0 (0.0%)	0 (0.0%)	0 (0.0%)	1 (0.8%)	0 (0.0%)	1 (0.1%)
- Arthropathy	0 (0.0%)	1 (0.8%)	0 (0.0%)	0 (0.0%)	0 (0.0%)	0 (0.0%)	1 (0.1%)
**Gastrointestinal disorders**	**1 (0.8%)**	**2 (1.6%)**	**3 (2.4%)**	**5 (4.0%)**	**3 (2.5%)**	**5 (4.0%)**	**19 (2.6%)**
- Vomiting	0 (0.0%)	1 (0.8%)	0 (0.0%)	3 (2.4%)	0 (0.0%)	3 (2.4%)	7 (0.9%)
- Nausea	0 (0.0%)	1 (0.8%)	2 (1.6%)	1 (0.8%)	1 (0.8%)	0 (0.0%)	5 (0.7%)
- Mallory-Weiss syndrome	0 (0.0%)	0 (0.0%)	0 (0.0%)	0 (0.0%)	0 (0.0%)	1 (0.8%)	1 (0.1%)
- Dyspepsia	0 (0.0%)	0 (0.0%)	1 (0.8%)	0 (0.0%)	0 (0.0%)	0 (0.0%)	1 (0.1%)
- Upper gastrointestinal haemorrhage	0 (0.0%)	0 (0.0%)	0 (0.0%)	0 (0.0%)	1 (0.8%)	0 (0.0%)	1 (0.1%)
- Abdominal hernia	1 (0.8%)	0 (0.0%)	0 (0.0%)	0 (0.0%)	0 (0.0%)	0 (0.0%)	1 (0.1%)
- Abdominal pain	0 (0.0%)	0 (0.0%)	0 (0.0%)	1 (0.8%)	0 (0.0%)	0 (0.0%)	1 (0.1%)
- Pancreatitis	0 (0.0%)	0 (0.0%)	0 (0.0%)	0 (0.0%)	0 (0.0%)	1 (0.8%)	1 (0.1%)
- Abdominal pain upper	0 (0.0%)	0 (0.0%)	0 (0.0%)	0 (0.0%)	1 (0.8%)	0 (0.0%)	1 (0.1%)
- Pancreatitis acute	0 (0.0%)	0 (0.0%)	0 (0.0%)	0 (0.0%)	0 (0.0%)	1 (0.8%)	1 (0.1%)
- Acute abdomen	0 (0.0%)	0 (0.0%)	1 (0.8%)	0 (0.0%)	0 (0.0%)	0 (0.0%)	1 (0.1%)
- Small intestinal obstruction	0 (0.0%)	0 (0.0%)	0 (0.0%)	1 (0.8%)	0 (0.0%)	0 (0.0%)	1 (0.1%)
- Gastritis	0 (0.0%)	0 (0.0%)	0 (0.0%)	0 (0.0%)	1 (0.8%)	0 (0.0%)	1 (0.1%)
**Psychiatric disorders**	**1 (0.8%)**	**4 (3.3%)**	**2 (1.6%)**	**2 (1.6%)**	**4 (3.3%)**	**5 (4.0%)**	**18 (2.4%)**
- Depression	0 (0.0%)	3 (2.5%)	0 (0.0%)	1 (0.8%)	1 (0.8%)	3 (2.4%)	8 (1.1%)
- Anxiety	0 (0.0%)	1 (0.8%)	1 (0.8%)	2 (1.6%)	0 (0.0%)	2 (1.6%)	6 (0.8%)
- Suicide attempt	0 (0.0%)	1 (0.8%)	0 (0.0%)	0 (0.0%)	3 (2.5%)	1 (0.8%)	5 (0.7%)
- Drug dependence	0 (0.0%)	1 (0.8%)	0 (0.0%)	0 (0.0%)	0 (0.0%)	0 (0.0%)	1 (0.1%)
- Brief psychotic disorder without marked stressors	1 (0.8%)	0 (0.0%)	0 (0.0%)	0 (0.0%)	0 (0.0%)	0 (0.0%)	1 (0.1%)
- Anxiety disorder	0 (0.0%)	0 (0.0%)	1 (0.8%)	0 (0.0%)	0 (0.0%)	0 (0.0%)	1 (0.1%)

Number (%) of participants experiencing adverse events (AEs) of grade 3 or higher by SOC and PT by Week 104 (Safety Population)							
Event<br>**System Organ Class (SOC)**<br>- Preferred Term (PT)	**9BLMZ (n = 126)**	**9BCLLfxZ (n = 122)**	**9BDLLfxZ (n = 127)**	**9DCLLfxZ (n = 124)**	**9DCMZ (n = 120)**	**Control (n = 126)**	**Total* (N = 745)**
**Respiratory, thoracic and mediastinal disorders**	**0 (0.0%)**	**3 (2.5%)**	**4 (3.1%)**	**2 (1.6%)**	**3 (2.5%)**	**4 (3.2%)**	**16 (2.1%)**
- Pneumothorax	0 (0.0%)	0 (0.0%)	0 (0.0%)	1 (0.8%)	1 (0.8%)	1 (0.8%)	3 (0.4%)
- Haemoptysis	0 (0.0%)	0 (0.0%)	0 (0.0%)	0 (0.0%)	1 (0.8%)	2 (1.6%)	3 (0.4%)
- Dyspnoea	0 (0.0%)	1 (0.8%)	1 (0.8%)	1 (0.8%)	0 (0.0%)	0 (0.0%)	3 (0.4%)
- Cough	0 (0.0%)	0 (0.0%)	1 (0.8%)	0 (0.0%)	2 (1.7%)	0 (0.0%)	3 (0.4%)
- Respiratory failure	0 (0.0%)	0 (0.0%)	1 (0.8%)	0 (0.0%)	0 (0.0%)	1 (0.8%)	2 (0.3%)
- Pulmonary cavitation	0 (0.0%)	0 (0.0%)	0 (0.0%)	0 (0.0%)	0 (0.0%)	1 (0.8%)	1 (0.1%)
- Acute pulmonary oedema	0 (0.0%)	1 (0.8%)	0 (0.0%)	0 (0.0%)	0 (0.0%)	0 (0.0%)	1 (0.1%)
- Pulmonary haemorrhage	0 (0.0%)	1 (0.8%)	0 (0.0%)	0 (0.0%)	0 (0.0%)	0 (0.0%)	1 (0.1%)
- Acute respiratory failure	0 (0.0%)	0 (0.0%)	1 (0.8%)	0 (0.0%)	0 (0.0%)	0 (0.0%)	1 (0.1%)
**Skin and subcutaneous tissue disorders**	**1 (0.8%)**	**2 (1.6%)**	**2 (1.6%)**	**3 (2.4%)**	**3 (2.5%)**	**1 (0.8%)**	**12 (1.6%)**
- Rash	0 (0.0%)	1 (0.8%)	1 (0.8%)	2 (1.6%)	0 (0.0%)	0 (0.0%)	4 (0.5%)
- Pruritus	0 (0.0%)	1 (0.8%)	0 (0.0%)	0 (0.0%)	2 (1.7%)	0 (0.0%)	3 (0.4%)
- Drug reaction with eosinophilia and systemic symptoms	0 (0.0%)	0 (0.0%)	0 (0.0%)	1 (0.8%)	1 (0.8%)	0 (0.0%)	2 (0.3%)
- Eczema	0 (0.0%)	0 (0.0%)	0 (0.0%)	0 (0.0%)	0 (0.0%)	1 (0.8%)	1 (0.1%)
- Dry skin	0 (0.0%)	0 (0.0%)	1 (0.8%)	0 (0.0%)	0 (0.0%)	0 (0.0%)	1 (0.1%)
- Fixed eruption	1 (0.8%)	0 (0.0%)	0 (0.0%)	0 (0.0%)	0 (0.0%)	0 (0.0%)	1 (0.1%)
- Dermatitis exfoliative	0 (0.0%)	0 (0.0%)	0 (0.0%)	0 (0.0%)	1 (0.8%)	0 (0.0%)	1 (0.1%)
**General disorders and administration site conditions**	**1 (0.8%)**	**1 (0.8%)**	**2 (1.6%)**	**2 (1.6%)**	**2 (1.7%)**	**4 (3.2%)**	**12 (1.6%)**
- Fatigue	0 (0.0%)	1 (0.8%)	0 (0.0%)	0 (0.0%)	0 (0.0%)	2 (1.6%)	3 (0.4%)
- Malaise	0 (0.0%)	1 (0.8%)	0 (0.0%)	0 (0.0%)	0 (0.0%)	1 (0.8%)	2 (0.3%)
- Chest pain	0 (0.0%)	0 (0.0%)	1 (0.8%)	0 (0.0%)	0 (0.0%)	1 (0.8%)	2 (0.3%)
- Pyrexia	0 (0.0%)	0 (0.0%)	0 (0.0%)	0 (0.0%)	1 (0.8%)	1 (0.8%)	2 (0.3%)
- Generalised oedema	1 (0.8%)	0 (0.0%)	0 (0.0%)	0 (0.0%)	0 (0.0%)	0 (0.0%)	1 (0.1%)
- Asthenia	0 (0.0%)	0 (0.0%)	0 (0.0%)	0 (0.0%)	0 (0.0%)	1 (0.8%)	1 (0.1%)
- Mucosal inflammation	0 (0.0%)	0 (0.0%)	1 (0.8%)	0 (0.0%)	0 (0.0%)	0 (0.0%)	1 (0.1%)
- Death	0 (0.0%)	0 (0.0%)	0 (0.0%)	1 (0.8%)	0 (0.0%)	0 (0.0%)	1 (0.1%)
- Sudden death	0 (0.0%)	0 (0.0%)	0 (0.0%)	0 (0.0%)	1 (0.8%)	0 (0.0%)	1 (0.1%)
- Disease progression	0 (0.0%)	0 (0.0%)	0 (0.0%)	1 (0.8%)	0 (0.0%)	0 (0.0%)	1 (0.1%)
**Injury, poisoning and procedural complications**	**0 (0.0%)**	**1 (0.8%)**	**2 (1.6%)**	**2 (1.6%)**	**2 (1.7%)**	**4 (3.2%)**	**11 (1.5%)**
- Alcohol poisoning	0 (0.0%)	1 (0.8%)	0 (0.0%)	0 (0.0%)	0 (0.0%)	2 (1.6%)	3 (0.4%)
- Thermal burn	0 (0.0%)	0 (0.0%)	1 (0.8%)	0 (0.0%)	0 (0.0%)	0 (0.0%)	1 (0.1%)
- Hand fracture	0 (0.0%)	0 (0.0%)	0 (0.0%)	1 (0.8%)	0 (0.0%)	0 (0.0%)	1 (0.1%)

Number (%) of participants experiencing adverse events (AEs) of grade 3 or higher by SOC and PT by Week 104 (Safety Population)							
Event<br>**System Organ Class (SOC)**<br>- Preferred Term (PT)	**9BLMZ (n = 126)**	**9BCLLfxZ (n = 122)**	**9BDLLfxZ (n = 127)**	**9DCLLfxZ (n = 124)**	**9DCMZ (n = 120)**	**Control (n = 126)**	**Total* (N = 745)**
- Thoracic vertebral fracture	0 (0.0%)	0 (0.0%)	0 (0.0%)	1 (0.8%)	0 (0.0%)	0 (0.0%)	1 (0.1%)
- Femur fracture	0 (0.0%)	0 (0.0%)	0 (0.0%)	0 (0.0%)	1 (0.8%)	0 (0.0%)	1 (0.1%)
- Stab wound	0 (0.0%)	0 (0.0%)	0 (0.0%)	0 (0.0%)	0 (0.0%)	1 (0.8%)	1 (0.1%)
- Hip fracture	0 (0.0%)	0 (0.0%)	0 (0.0%)	0 (0.0%)	1 (0.8%)	0 (0.0%)	1 (0.1%)
- Abortion induced incomplete	0 (0.0%)	0 (0.0%)	1 (0.8%)	0 (0.0%)	0 (0.0%)	0 (0.0%)	1 (0.1%)
- Ankle fracture	0 (0.0%)	0 (0.0%)	0 (0.0%)	0 (0.0%)	0 (0.0%)	1 (0.8%)	1 (0.1%)
**Cardiac disorders**	**1 (0.8%)**	**2 (1.6%)**	**0 (0.0%)**	**2 (1.6%)**	**0 (0.0%)**	**1 (0.8%)**	**6 (0.8%)**
- Acute myocardial infarction	0 (0.0%)	1 (0.8%)	0 (0.0%)	2 (1.6%)	0 (0.0%)	0 (0.0%)	3 (0.4%)
- Cardiac failure congestive	1 (0.8%)	1 (0.8%)	0 (0.0%)	0 (0.0%)	0 (0.0%)	0 (0.0%)	2 (0.3%)
- Cardiac failure	0 (0.0%)	1 (0.8%)	0 (0.0%)	1 (0.8%)	0 (0.0%)	0 (0.0%)	2 (0.3%)
- Cardiomyopathy alcoholic	0 (0.0%)	0 (0.0%)	0 (0.0%)	0 (0.0%)	0 (0.0%)	1 (0.8%)	1 (0.1%)
**Vascular disorders**	**1 (0.8%)**	**1 (0.8%)**	**1 (0.8%)**	**2 (1.6%)**	**0 (0.0%)**	**0 (0.0%)**	**5 (0.7%)**
- Hypotension	0 (0.0%)	0 (0.0%)	1 (0.8%)	1 (0.8%)	0 (0.0%)	0 (0.0%)	2 (0.3%)
- Deep vein thrombosis	0 (0.0%)	0 (0.0%)	0 (0.0%)	1 (0.8%)	0 (0.0%)	0 (0.0%)	1 (0.1%)
- Diabetic vascular disorder	1 (0.8%)	0 (0.0%)	0 (0.0%)	0 (0.0%)	0 (0.0%)	0 (0.0%)	1 (0.1%)
- Hypertension	0 (0.0%)	1 (0.8%)	0 (0.0%)	0 (0.0%)	0 (0.0%)	0 (0.0%)	1 (0.1%)
**Renal and urinary disorders**	**1 (0.8%)**	**0 (0.0%)**	**1 (0.8%)**	**0 (0.0%)**	**0 (0.0%)**	**1 (0.8%)**	**3 (0.4%)**
- Proteinuria	1 (0.8%)	0 (0.0%)	1 (0.8%)	0 (0.0%)	0 (0.0%)	0 (0.0%)	2 (0.3%)
- Acute kidney injury	1 (0.8%)	0 (0.0%)	0 (0.0%)	0 (0.0%)	0 (0.0%)	0 (0.0%)	1 (0.1%)
- Chronic kidney disease	0 (0.0%)	0 (0.0%)	0 (0.0%)	0 (0.0%)	0 (0.0%)	1 (0.8%)	1 (0.1%)
**Immune system disorders**	**0 (0.0%)**	**0 (0.0%)**	**2 (1.6%)**	**0 (0.0%)**	**0 (0.0%)**	**0 (0.0%)**	**2 (0.3%)**
- Hypersensitivity	0 (0.0%)	0 (0.0%)	2 (1.6%)	0 (0.0%)	0 (0.0%)	0 (0.0%)	2 (0.3%)
**Pregnancy, puerperium and perinatal conditions**	**0 (0.0%)**	**0 (0.0%)**	**1 (0.8%)**	**0 (0.0%)**	**0 (0.0%)**	**1 (0.8%)**	**2 (0.3%)**
- Abortion spontaneous	0 (0.0%)	0 (0.0%)	0 (0.0%)	0 (0.0%)	0 (0.0%)	1 (0.8%)	1 (0.1%)
- Abortion threatened	0 (0.0%)	0 (0.0%)	1 (0.8%)	0 (0.0%)	0 (0.0%)	0 (0.0%)	1 (0.1%)
**Neoplasms benign, malignant and unspecified (incl cysts and polyps)**	**0 (0.0%)**	**0 (0.0%)**	**0 (0.0%)**	**0 (0.0%)**	**1 (0.8%)**	**1 (0.8%)**	**2 (0.3%)**
- Lung neoplasm malignant	0 (0.0%)	0 (0.0%)	0 (0.0%)	0 (0.0%)	0 (0.0%)	1 (0.8%)	1 (0.1%)
- Pancreatic carcinoma	0 (0.0%)	0 (0.0%)	0 (0.0%)	0 (0.0%)	1 (0.8%)	0 (0.0%)	1 (0.1%)

**Total by preferred term (PT) represents the sum of participants who experienced at least one event corresponding to that PT. If they experienced more than one event of the same PT, it is counted only once. If a participant experienced more than one unique PT within a system organ class (SOC), each unique PT counts toward the PT total, but only one event counts toward the SOC.*

Table S33. Number (%) of participants experiencing serious adverse events (SAEs) by SOC and PT by Week 73 (Safety Population)

Number (%) of participants experiencing serious adverse events (SAEs) by SOC and PT by Week 73 (Safety Population)							
Event<br>**System Organ Class (SOC)**<br>- Preferred Term (PT)	**9BLMZ**<br>**(n = 126)**	**9BCLLfxZ**<br>**(n = 122)**	**9BDLLfxZ**<br>**(n = 127)**	**9DCLLfxZ**<br>**(n = 124)**	**9DCMZ**<br>**(n = 120)**	**Control**<br>**(n = 126)**	**Total***<br>**(N = 745)**
**Infections and infestations**	**11 (8.7%)**	**3 (2.5%)**	**6 (4.7%)**	**3 (2.4%)**	**6 (5.0%)**	**4 (3.2%)**	**33 (4.4%)**
- Pneumonia	4 (3.2%)	0 (0.0%)	3 (2.4%)	0 (0.0%)	1 (0.8%)	2 (1.6%)	10 (1.3%)
- COVID-19	2 (1.6%)	0 (0.0%)	0 (0.0%)	0 (0.0%)	1 (0.8%)	1 (0.8%)	4 (0.5%)
- COVID-19 pneumonia	0 (0.0%)	2 (1.6%)	0 (0.0%)	0 (0.0%)	1 (0.8%)	0 (0.0%)	3 (0.4%)
- Sepsis	0 (0.0%)	1 (0.8%)	0 (0.0%)	1 (0.8%)	1 (0.8%)	0 (0.0%)	3 (0.4%)
- Pneumocystis jirovecii pneumonia	1 (0.8%)	0 (0.0%)	0 (0.0%)	1 (0.8%)	0 (0.0%)	0 (0.0%)	2 (0.3%)
- Abscess soft tissue	0 (0.0%)	0 (0.0%)	0 (0.0%)	0 (0.0%)	1 (0.8%)	0 (0.0%)	1 (0.1%)
- Skin infection	1 (0.8%)	0 (0.0%)	0 (0.0%)	0 (0.0%)	0 (0.0%)	0 (0.0%)	1 (0.1%)
- Tonsillitis	0 (0.0%)	0 (0.0%)	0 (0.0%)	0 (0.0%)	0 (0.0%)	1 (0.8%)	1 (0.1%)
- Pneumonia staphylococcal	0 (0.0%)	0 (0.0%)	1 (0.8%)	0 (0.0%)	0 (0.0%)	0 (0.0%)	1 (0.1%)
- Appendicitis	0 (0.0%)	0 (0.0%)	0 (0.0%)	0 (0.0%)	0 (0.0%)	1 (0.8%)	1 (0.1%)
- Gastroenteritis	0 (0.0%)	0 (0.0%)	0 (0.0%)	1 (0.8%)	0 (0.0%)	0 (0.0%)	1 (0.1%)
- Septic shock	1 (0.8%)	0 (0.0%)	0 (0.0%)	0 (0.0%)	0 (0.0%)	0 (0.0%)	1 (0.1%)
- Cellulitis	0 (0.0%)	0 (0.0%)	1 (0.8%)	0 (0.0%)	0 (0.0%)	0 (0.0%)	1 (0.1%)
- Herpes zoster	0 (0.0%)	0 (0.0%)	0 (0.0%)	0 (0.0%)	1 (0.8%)	0 (0.0%)	1 (0.1%)
- Wound infection	1 (0.8%)	0 (0.0%)	0 (0.0%)	0 (0.0%)	0 (0.0%)	0 (0.0%)	1 (0.1%)
- Pulmonary tuberculoma	0 (0.0%)	0 (0.0%)	1 (0.8%)	0 (0.0%)	0 (0.0%)	0 (0.0%)	1 (0.1%)
- Respiratory tract infection	0 (0.0%)	0 (0.0%)	1 (0.8%)	0 (0.0%)	0 (0.0%)	0 (0.0%)	1 (0.1%)
- Pneumonia bacterial	0 (0.0%)	0 (0.0%)	1 (0.8%)	0 (0.0%)	0 (0.0%)	0 (0.0%)	1 (0.1%)
- Post procedural sepsis	0 (0.0%)	0 (0.0%)	0 (0.0%)	1 (0.8%)	0 (0.0%)	0 (0.0%)	1 (0.1%)
- Tuberculosis	1 (0.8%)	0 (0.0%)	0 (0.0%)	0 (0.0%)	0 (0.0%)	0 (0.0%)	1 (0.1%)
- Dengue fever	1 (0.8%)	0 (0.0%)	0 (0.0%)	0 (0.0%)	0 (0.0%)	0 (0.0%)	1 (0.1%)
**Blood and lymphatic system disorders**	**1 (0.8%)**	**3 (2.5%)**	**3 (2.4%)**	**3 (2.4%)**	**2 (1.7%)**	**4 (3.2%)**	**16 (2.1%)**
- Anaemia	1 (0.8%)	3 (2.5%)	3 (2.4%)	2 (1.6%)	2 (1.7%)	4 (3.2%)	15 (2.0%)
- Pancytopenia	0 (0.0%)	0 (0.0%)	0 (0.0%)	1 (0.8%)	0 (0.0%)	0 (0.0%)	1 (0.1%)
**Investigations**	**3 (2.4%)**	**4 (3.3%)**	**3 (2.4%)**	**0 (0.0%)**	**2 (1.7%)**	**3 (2.4%)**	**15 (2.0%)**
- Alanine aminotransferase increased	3 (2.4%)	1 (0.8%)	2 (1.6%)	0 (0.0%)	0 (0.0%)	0 (0.0%)	6 (0.8%)
- Aspartate aminotransferase increased	1 (0.8%)	1 (0.8%)	2 (1.6%)	0 (0.0%)	0 (0.0%)	1 (0.8%)	5 (0.7%)
- Electrocardiogram QT prolonged	0 (0.0%)	2 (1.6%)	0 (0.0%)	0 (0.0%)	2 (1.7%)	0 (0.0%)	4 (0.5%)
- Bilirubin conjugated increased	0 (0.0%)	0 (0.0%)	1 (0.8%)	0 (0.0%)	0 (0.0%)	1 (0.8%)	2 (0.3%)

**Number (%) of participants experiencing serious adverse events (SAEs) by SOC and PT by Week 73 (Safety Population)**							
Event<br>**System Organ Class (SOC)**<br>- Preferred Term (PT)	**9BLMZ**<br>**(n = 126)**	**9BCLLfxZ**<br>**(n = 122)**	**9BDLLfxZ**<br>**(n = 127)**	**9DCLLfxZ**<br>**(n = 124)**	**9DCMZ**<br>**(n = 120)**	**Control**<br>**(n = 126)**	**Total***<br>**(N = 745)**
- Lymphocyte count decreased	0 (0.0%)	0 (0.0%)	1 (0.8%)	0 (0.0%)	0 (0.0%)	0 (0.0%)	1 (0.1%)
- Neutrophil count decreased	0 (0.0%)	0 (0.0%)	0 (0.0%)	0 (0.0%)	0 (0.0%)	1 (0.8%)	1 (0.1%)
- Transaminases increased	0 (0.0%)	1 (0.8%)	0 (0.0%)	0 (0.0%)	0 (0.0%)	0 (0.0%)	1 (0.1%)
**Respiratory, thoracic and mediastinal disorders**	**0 (0.0%)**	**1 (0.8%)**	**3 (2.4%)**	**2 (1.6%)**	**2 (1.7%)**	**5 (4.0%)**	**13 (1.7%)**
- Haemoptysis	0 (0.0%)	0 (0.0%)	0 (0.0%)	0 (0.0%)	1 (0.8%)	3 (2.4%)	4 (0.5%)
- Pneumothorax	0 (0.0%)	0 (0.0%)	0 (0.0%)	1 (0.8%)	1 (0.8%)	1 (0.8%)	3 (0.4%)
- Respiratory failure	0 (0.0%)	0 (0.0%)	1 (0.8%)	0 (0.0%)	0 (0.0%)	1 (0.8%)	2 (0.3%)
- Dyspnoea	0 (0.0%)	0 (0.0%)	1 (0.8%)	1 (0.8%)	0 (0.0%)	0 (0.0%)	2 (0.3%)
- Pulmonary cavitation	0 (0.0%)	0 (0.0%)	0 (0.0%)	0 (0.0%)	0 (0.0%)	1 (0.8%)	1 (0.1%)
- Acute respiratory failure	0 (0.0%)	0 (0.0%)	1 (0.8%)	0 (0.0%)	0 (0.0%)	0 (0.0%)	1 (0.1%)
- Acute pulmonary oedema	0 (0.0%)	1 (0.8%)	0 (0.0%)	0 (0.0%)	0 (0.0%)	0 (0.0%)	1 (0.1%)
**Gastrointestinal disorders**	**1 (0.8%)**	**1 (0.8%)**	**1 (0.8%)**	**4 (3.2%)**	**1 (0.8%)**	**3 (2.4%)**	**11 (1.5%)**
- Vomiting	0 (0.0%)	1 (0.8%)	0 (0.0%)	3 (2.4%)	0 (0.0%)	2 (1.6%)	6 (0.8%)
- Small intestinal obstruction	0 (0.0%)	0 (0.0%)	0 (0.0%)	1 (0.8%)	0 (0.0%)	0 (0.0%)	1 (0.1%)
- Acute abdomen	0 (0.0%)	0 (0.0%)	1 (0.8%)	0 (0.0%)	0 (0.0%)	0 (0.0%)	1 (0.1%)
- Pancreatitis	0 (0.0%)	0 (0.0%)	0 (0.0%)	0 (0.0%)	0 (0.0%)	1 (0.8%)	1 (0.1%)
- Gastritis	0 (0.0%)	0 (0.0%)	0 (0.0%)	0 (0.0%)	1 (0.8%)	0 (0.0%)	1 (0.1%)
- Upper gastrointestinal haemorrhage	0 (0.0%)	0 (0.0%)	0 (0.0%)	0 (0.0%)	1 (0.8%)	0 (0.0%)	1 (0.1%)
- Abdominal hernia	1 (0.8%)	0 (0.0%)	0 (0.0%)	0 (0.0%)	0 (0.0%)	0 (0.0%)	1 (0.1%)
- Nausea	0 (0.0%)	0 (0.0%)	0 (0.0%)	1 (0.8%)	0 (0.0%)	0 (0.0%)	1 (0.1%)
**Injury, poisoning and procedural complications**	**0 (0.0%)**	**1 (0.8%)**	**2 (1.6%)**	**1 (0.8%)**	**2 (1.7%)**	**4 (3.2%)**	**10 (1.3%)**
- Alcohol poisoning	0 (0.0%)	1 (0.8%)	0 (0.0%)	0 (0.0%)	0 (0.0%)	2 (1.6%)	3 (0.4%)
- Hip fracture	0 (0.0%)	0 (0.0%)	0 (0.0%)	0 (0.0%)	1 (0.8%)	0 (0.0%)	1 (0.1%)
- Stab wound	0 (0.0%)	0 (0.0%)	0 (0.0%)	0 (0.0%)	0 (0.0%)	1 (0.8%)	1 (0.1%)
- Thoracic vertebral fracture	0 (0.0%)	0 (0.0%)	0 (0.0%)	1 (0.8%)	0 (0.0%)	0 (0.0%)	1 (0.1%)
- Thermal burn	0 (0.0%)	0 (0.0%)	1 (0.8%)	0 (0.0%)	0 (0.0%)	0 (0.0%)	1 (0.1%)
- Ankle fracture	0 (0.0%)	0 (0.0%)	0 (0.0%)	0 (0.0%)	0 (0.0%)	1 (0.8%)	1 (0.1%)
- Abortion induced incomplete	0 (0.0%)	0 (0.0%)	1 (0.8%)	0 (0.0%)	0 (0.0%)	0 (0.0%)	1 (0.1%)
- Femur fracture	0 (0.0%)	0 (0.0%)	0 (0.0%)	0 (0.0%)	1 (0.8%)	0 (0.0%)	1 (0.1%)
**Psychiatric disorders**	**0 (0.0%)**	**3 (2.5%)**	**1 (0.8%)**	**0 (0.0%)**	**3 (2.5%)**	**2 (1.6%)**	**9 (1.2%)**
- Suicide attempt	0 (0.0%)	1 (0.8%)	0 (0.0%)	0 (0.0%)	3 (2.5%)	1 (0.8%)	5 (0.7%)
- Depression	0 (0.0%)	0 (0.0%)	0 (0.0%)	0 (0.0%)	0 (0.0%)	1 (0.8%)	1 (0.1%)
- Anxiety disorder	0 (0.0%)	0 (0.0%)	1 (0.8%)	0 (0.0%)	0 (0.0%)	0 (0.0%)	1 (0.1%)

Number (%) of participants experiencing serious adverse events (SAEs) by SOC and PT by Week 73 (Safety Population)							
Event<br>**System Organ Class (SOC)**<br>- Preferred Term (PT)	**9BLMZ**<br>**(n = 126)**	**9BCLLfxZ**<br>**(n = 122)**	**9BDLLfxZ**<br>**(n = 127)**	**9DCLLfxZ**<br>**(n = 124)**	**9DCMZ**<br>**(n = 120)**	**Control**<br>**(n = 126)**	**Total***<br>**(N = 745)**
- Drug dependence	0 (0.0%)	1 (0.8%)	0 (0.0%)	0 (0.0%)	0 (0.0%)	0 (0.0%)	1 (0.1%)
- Psychotic disorder	0 (0.0%)	1 (0.8%)	0 (0.0%)	0 (0.0%)	0 (0.0%)	0 (0.0%)	1 (0.1%)
**Hepatobiliary disorders**	**1 (0.8%)**	**1 (0.8%)**	**0 (0.0%)**	**1 (0.8%)**	**2 (1.7%)**	**2 (1.6%)**	**7 (0.9%)**
- Hepatotoxicity	1 (0.8%)	0 (0.0%)	0 (0.0%)	0 (0.0%)	0 (0.0%)	1 (0.8%)	2 (0.3%)
- Drug-induced liver injury	0 (0.0%)	1 (0.8%)	0 (0.0%)	1 (0.8%)	0 (0.0%)	0 (0.0%)	2 (0.3%)
- Hepatitis	0 (0.0%)	0 (0.0%)	0 (0.0%)	0 (0.0%)	0 (0.0%)	1 (0.8%)	1 (0.1%)
- Acute on chronic liver failure	0 (0.0%)	0 (0.0%)	0 (0.0%)	0 (0.0%)	1 (0.8%)	0 (0.0%)	1 (0.1%)
- Hyperbilirubinaemia	0 (0.0%)	0 (0.0%)	0 (0.0%)	0 (0.0%)	1 (0.8%)	0 (0.0%)	1 (0.1%)
**Nervous system disorders**	**0 (0.0%)**	**2 (1.6%)**	**2 (1.6%)**	**2 (1.6%)**	**0 (0.0%)**	**1 (0.8%)**	**7 (0.9%)**
- Seizure	0 (0.0%)	1 (0.8%)	0 (0.0%)	1 (0.8%)	0 (0.0%)	0 (0.0%)	2 (0.3%)
- Idiopathic intracranial hypertension	0 (0.0%)	0 (0.0%)	0 (0.0%)	0 (0.0%)	0 (0.0%)	1 (0.8%)	1 (0.1%)
- Facial paralysis	0 (0.0%)	0 (0.0%)	1 (0.8%)	0 (0.0%)	0 (0.0%)	0 (0.0%)	1 (0.1%)
- Optic neuritis	0 (0.0%)	0 (0.0%)	0 (0.0%)	1 (0.8%)	0 (0.0%)	0 (0.0%)	1 (0.1%)
- Neuropathy peripheral	0 (0.0%)	0 (0.0%)	1 (0.8%)	0 (0.0%)	0 (0.0%)	0 (0.0%)	1 (0.1%)
- Transient ischaemic attack	0 (0.0%)	1 (0.8%)	0 (0.0%)	0 (0.0%)	0 (0.0%)	0 (0.0%)	1 (0.1%)
**General disorders and administration site conditions**	**2 (1.6%)**	**0 (0.0%)**	**0 (0.0%)**	**2 (1.6%)**	**2 (1.7%)**	**0 (0.0%)**	**6 (0.8%)**
- Sudden death	0 (0.0%)	0 (0.0%)	0 (0.0%)	0 (0.0%)	1 (0.8%)	0 (0.0%)	1 (0.1%)
- Generalised oedema	1 (0.8%)	0 (0.0%)	0 (0.0%)	0 (0.0%)	0 (0.0%)	0 (0.0%)	1 (0.1%)
- Pyrexia	1 (0.8%)	0 (0.0%)	0 (0.0%)	0 (0.0%)	0 (0.0%)	0 (0.0%)	1 (0.1%)
- Disease progression	0 (0.0%)	0 (0.0%)	0 (0.0%)	1 (0.8%)	0 (0.0%)	0 (0.0%)	1 (0.1%)
- Chest pain	0 (0.0%)	0 (0.0%)	0 (0.0%)	0 (0.0%)	1 (0.8%)	0 (0.0%)	1 (0.1%)
- Death	0 (0.0%)	0 (0.0%)	0 (0.0%)	1 (0.8%)	0 (0.0%)	0 (0.0%)	1 (0.1%)
**Cardiac disorders**	**1 (0.8%)**	**2 (1.6%)**	**0 (0.0%)**	**2 (1.6%)**	**0 (0.0%)**	**0 (0.0%)**	**5 (0.7%)**
- Cardiac failure congestive	1 (0.8%)	1 (0.8%)	0 (0.0%)	0 (0.0%)	0 (0.0%)	0 (0.0%)	2 (0.3%)
- Cardiac failure	0 (0.0%)	1 (0.8%)	0 (0.0%)	1 (0.8%)	0 (0.0%)	0 (0.0%)	2 (0.3%)
- Acute myocardial infarction	0 (0.0%)	1 (0.8%)	0 (0.0%)	1 (0.8%)	0 (0.0%)	0 (0.0%)	2 (0.3%)
**Metabolism and nutrition disorders**	**1 (0.8%)**	**2 (1.6%)**	**0 (0.0%)**	**0 (0.0%)**	**2 (1.7%)**	**0 (0.0%)**	**5 (0.7%)**
- Diabetes mellitus inadequate control	0 (0.0%)	0 (0.0%)	0 (0.0%)	0 (0.0%)	2 (1.7%)	0 (0.0%)	2 (0.3%)
- Hyperglycaemia	0 (0.0%)	2 (1.6%)	0 (0.0%)	0 (0.0%)	0 (0.0%)	0 (0.0%)	2 (0.3%)
- Hypoalbuminaemia	1 (0.8%)	0 (0.0%)	0 (0.0%)	0 (0.0%)	0 (0.0%)	0 (0.0%)	1 (0.1%)
**Skin and subcutaneous tissue disorders**	**0 (0.0%)**	**0 (0.0%)**	**0 (0.0%)**	**1 (0.8%)**	**3 (2.5%)**	**0 (0.0%)**	**4 (0.5%)**
- Drug reaction with eosinophilia and systemic symptoms	0 (0.0%)	0 (0.0%)	0 (0.0%)	1 (0.8%)	1 (0.8%)	0 (0.0%)	2 (0.3%)
- Swelling face	0 (0.0%)	0 (0.0%)	0 (0.0%)	0 (0.0%)	1 (0.8%)	0 (0.0%)	1 (0.1%)

Number (%) of participants experiencing serious adverse events (SAEs) by SOC and PT by Week 73 (Safety Population)							
Event **System Organ Class (SOC)** - Preferred Term (PT)	**9BLMZ (n = 126)**	**9BCLLfxZ (n = 122)**	**9BDLLfxZ (n = 127)**	**9DCLLfxZ (n = 124)**	**9DCMZ (n = 120)**	**Control (n = 126)**	**Total* (N = 745)**
- Dermatitis exfoliative	0 (0.0%)	0 (0.0%)	0 (0.0%)	0 (0.0%)	1 (0.8%)	0 (0.0%)	1 (0.1%)
**Musculoskeletal and connective tissue disorders**	**0 (0.0%)**	**1 (0.8%)**	**0 (0.0%)**	**1 (0.8%)**	**2 (1.7%)**	**0 (0.0%)**	**4 (0.5%)**
- Arthralgia	0 (0.0%)	0 (0.0%)	0 (0.0%)	1 (0.8%)	1 (0.8%)	0 (0.0%)	2 (0.3%)
- Arthropathy	0 (0.0%)	1 (0.8%)	0 (0.0%)	0 (0.0%)	0 (0.0%)	0 (0.0%)	1 (0.1%)
- Osteoarthritis	0 (0.0%)	0 (0.0%)	0 (0.0%)	0 (0.0%)	1 (0.8%)	0 (0.0%)	1 (0.1%)
**Eye disorders**	**1 (0.8%)**	**2 (1.6%)**	**0 (0.0%)**	**0 (0.0%)**	**0 (0.0%)**	**0 (0.0%)**	**3 (0.4%)**
- Optic neuropathy	1 (0.8%)	1 (0.8%)	0 (0.0%)	0 (0.0%)	0 (0.0%)	0 (0.0%)	2 (0.3%)
- Eye haemorrhage	0 (0.0%)	1 (0.8%)	0 (0.0%)	0 (0.0%)	0 (0.0%)	0 (0.0%)	1 (0.1%)
**Vascular disorders**	**1 (0.8%)**	**1 (0.8%)**	**0 (0.0%)**	**1 (0.8%)**	**0 (0.0%)**	**0 (0.0%)**	**3 (0.4%)**
- Deep vein thrombosis	0 (0.0%)	0 (0.0%)	0 (0.0%)	1 (0.8%)	0 (0.0%)	0 (0.0%)	1 (0.1%)
- Hypertension	0 (0.0%)	1 (0.8%)	0 (0.0%)	0 (0.0%)	0 (0.0%)	0 (0.0%)	1 (0.1%)
- Diabetic vascular disorder	1 (0.8%)	0 (0.0%)	0 (0.0%)	0 (0.0%)	0 (0.0%)	0 (0.0%)	1 (0.1%)
**Renal and urinary disorders**	**1 (0.8%)**	**0 (0.0%)**	**1 (0.8%)**	**0 (0.0%)**	**0 (0.0%)**	**0 (0.0%)**	**2 (0.3%)**
- Proteinuria	0 (0.0%)	0 (0.0%)	1 (0.8%)	0 (0.0%)	0 (0.0%)	0 (0.0%)	1 (0.1%)
- Acute kidney injury	1 (0.8%)	0 (0.0%)	0 (0.0%)	0 (0.0%)	0 (0.0%)	0 (0.0%)	1 (0.1%)
**Immune system disorders**	**0 (0.0%)**	**0 (0.0%)**	**2 (1.6%)**	**0 (0.0%)**	**0 (0.0%)**	**0 (0.0%)**	**2 (0.3%)**
- Hypersensitivity	0 (0.0%)	0 (0.0%)	2 (1.6%)	0 (0.0%)	0 (0.0%)	0 (0.0%)	2 (0.3%)
**Pregnancy, puerperium and perinatal conditions**	**0 (0.0%)**	**0 (0.0%)**	**1 (0.8%)**	**0 (0.0%)**	**0 (0.0%)**	**0 (0.0%)**	**1 (0.1%)**
- Abortion threatened	0 (0.0%)	0 (0.0%)	1 (0.8%)	0 (0.0%)	0 (0.0%)	0 (0.0%)	1 (0.1%)
**Neoplasms benign, malignant and unspecified (incl cysts and polyps)**	**0 (0.0%)**	**0 (0.0%)**	**0 (0.0%)**	**0 (0.0%)**	**0 (0.0%)**	**1 (0.8%)**	**1 (0.1%)**
- Lung neoplasm malignant	0 (0.0%)	0 (0.0%)	0 (0.0%)	0 (0.0%)	0 (0.0%)	1 (0.8%)	1 (0.1%)
**Product issues**	**1 (0.8%)**	**0 (0.0%)**	**0 (0.0%)**	**0 (0.0%)**	**0 (0.0%)**	**0 (0.0%)**	**1 (0.1%)**
- Device dislocation	1 (0.8%)	0 (0.0%)	0 (0.0%)	0 (0.0%)	0 (0.0%)	0 (0.0%)	1 (0.1%)

**Total by preferred term (PT) represents the sum of participants who experienced at least one event corresponding to that PT. If they experienced more than one event of the same PT, it is counted only once. If a participant experienced more than one unique PT within a system organ class (SOC), each unique PT counts toward the PT total, but only one event counts toward the SOC.*

Table S34. Number (%) of participants experiencing serious adverse events (SAEs) by SOC and PT by Week 104 (Safety Population)

**Number (%) of participants experiencing serious adverse events (SAEs) by SOC and PT by Week 104 (Safety Population)**							
Event<br>**System Organ Class (SOC)**<br>- Preferred Term (PT)	**9BLMZ**<br>**(n = 126)**	**9BCLLfxZ**<br>**(n = 122)**	**9BDLLfxZ**<br>**(n = 127)**	**9DCLLfxZ**<br>**(n = 124)**	**9DCMZ**<br>**(n = 120)**	**Control**<br>**(n = 126)**	**Total***<br>**(N = 745)**
**Infections and infestations**	**12 (9.5%)**	**6 (4.9%)**	**6 (4.7%)**	**3 (2.4%)**	**7 (5.8%)**	**4 (3.2%)**	**38 (5.1%)**
- Pneumonia	4 (3.2%)	1 (0.8%)	3 (2.4%)	0 (0.0%)	1 (0.8%)	2 (1.6%)	11 (1.5%)
- COVID-19	2 (1.6%)	0 (0.0%)	0 (0.0%)	0 (0.0%)	2 (1.7%)	1 (0.8%)	5 (0.7%)
- COVID-19 pneumonia	0 (0.0%)	4 (3.3%)	0 (0.0%)	0 (0.0%)	1 (0.8%)	0 (0.0%)	5 (0.7%)
- Sepsis	0 (0.0%)	1 (0.8%)	0 (0.0%)	1 (0.8%)	1 (0.8%)	0 (0.0%)	3 (0.4%)
- Appendicitis	1 (0.8%)	0 (0.0%)	0 (0.0%)	0 (0.0%)	0 (0.0%)	1 (0.8%)	2 (0.3%)
- Pneumocystis jirovecii pneumonia	1 (0.8%)	0 (0.0%)	0 (0.0%)	1 (0.8%)	0 (0.0%)	0 (0.0%)	2 (0.3%)
- Pneumonia bacterial	0 (0.0%)	0 (0.0%)	1 (0.8%)	0 (0.0%)	0 (0.0%)	0 (0.0%)	1 (0.1%)
- Respiratory tract infection	0 (0.0%)	0 (0.0%)	1 (0.8%)	0 (0.0%)	0 (0.0%)	0 (0.0%)	1 (0.1%)
- Post procedural sepsis	0 (0.0%)	0 (0.0%)	0 (0.0%)	1 (0.8%)	0 (0.0%)	0 (0.0%)	1 (0.1%)
- Gastroenteritis	0 (0.0%)	0 (0.0%)	0 (0.0%)	1 (0.8%)	0 (0.0%)	0 (0.0%)	1 (0.1%)
- Diabetic foot infection	1 (0.8%)	0 (0.0%)	0 (0.0%)	0 (0.0%)	0 (0.0%)	0 (0.0%)	1 (0.1%)
- Pneumonia staphylococcal	0 (0.0%)	0 (0.0%)	1 (0.8%)	0 (0.0%)	0 (0.0%)	0 (0.0%)	1 (0.1%)
- Tonsillitis	0 (0.0%)	0 (0.0%)	0 (0.0%)	0 (0.0%)	0 (0.0%)	1 (0.8%)	1 (0.1%)
- Skin infection	1 (0.8%)	0 (0.0%)	0 (0.0%)	0 (0.0%)	0 (0.0%)	0 (0.0%)	1 (0.1%)
- Dengue fever	1 (0.8%)	0 (0.0%)	0 (0.0%)	0 (0.0%)	0 (0.0%)	0 (0.0%)	1 (0.1%)
- Herpes zoster	0 (0.0%)	0 (0.0%)	0 (0.0%)	0 (0.0%)	1 (0.8%)	0 (0.0%)	1 (0.1%)
- Septic shock	1 (0.8%)	0 (0.0%)	0 (0.0%)	0 (0.0%)	0 (0.0%)	0 (0.0%)	1 (0.1%)
- Cellulitis	0 (0.0%)	0 (0.0%)	1 (0.8%)	0 (0.0%)	0 (0.0%)	0 (0.0%)	1 (0.1%)
- Wound infection	1 (0.8%)	0 (0.0%)	0 (0.0%)	0 (0.0%)	0 (0.0%)	0 (0.0%)	1 (0.1%)
- Tuberculosis	1 (0.8%)	0 (0.0%)	0 (0.0%)	0 (0.0%)	0 (0.0%)	0 (0.0%)	1 (0.1%)
- Pulmonary tuberculoma	0 (0.0%)	0 (0.0%)	1 (0.8%)	0 (0.0%)	0 (0.0%)	0 (0.0%)	1 (0.1%)
- Abscess soft tissue	0 (0.0%)	0 (0.0%)	0 (0.0%)	0 (0.0%)	1 (0.8%)	0 (0.0%)	1 (0.1%)
- Osteomyelitis	1 (0.8%)	0 (0.0%)	0 (0.0%)	0 (0.0%)	0 (0.0%)	0 (0.0%)	1 (0.1%)
**Blood and lymphatic system disorders**	**1 (0.8%)**	**3 (2.5%)**	**3 (2.4%)**	**4 (3.2%)**	**2 (1.7%)**	**4 (3.2%)**	**17 (2.3%)**
- Anaemia	1 (0.8%)	3 (2.5%)	3 (2.4%)	3 (2.4%)	2 (1.7%)	4 (3.2%)	16 (2.1%)
- Pancytopenia	0 (0.0%)	0 (0.0%)	0 (0.0%)	1 (0.8%)	0 (0.0%)	0 (0.0%)	1 (0.1%)
**Investigations**	**3 (2.4%)**	**4 (3.3%)**	**3 (2.4%)**	**0 (0.0%)**	**2 (1.7%)**	**3 (2.4%)**	**15 (2.0%)**
- Alanine aminotransferase increased	3 (2.4%)	1 (0.8%)	2 (1.6%)	0 (0.0%)	0 (0.0%)	0 (0.0%)	6 (0.8%)
- Aspartate aminotransferase increased	1 (0.8%)	1 (0.8%)	2 (1.6%)	0 (0.0%)	0 (0.0%)	1 (0.8%)	5 (0.7%)
- Electrocardiogram QT prolonged	0 (0.0%)	2 (1.6%)	0 (0.0%)	0 (0.0%)	2 (1.7%)	0 (0.0%)	4 (0.5%)

Number (%) of participants experiencing serious adverse events (SAEs) by SOC and PT by Week 104 (Safety Population)							
Event<br>**System Organ Class (SOC)**<br>- Preferred Term (PT)	**9BLMZ**<br>**(n = 126)**	**9BCLLfxZ**<br>**(n = 122)**	**9BDLLfxZ**<br>**(n = 127)**	**9DCLLfxZ**<br>**(n = 124)**	**9DCMZ**<br>**(n = 120)**	**Control**<br>**(n = 126)**	**Total***<br>**(N = 745)**
- Bilirubin conjugated increased	0 (0.0%)	0 (0.0%)	1 (0.8%)	0 (0.0%)	0 (0.0%)	1 (0.8%)	2 (0.3%)
- Lymphocyte count decreased	0 (0.0%)	0 (0.0%)	1 (0.8%)	0 (0.0%)	0 (0.0%)	0 (0.0%)	1 (0.1%)
- Transaminases increased	0 (0.0%)	1 (0.8%)	0 (0.0%)	0 (0.0%)	0 (0.0%)	0 (0.0%)	1 (0.1%)
- Neutrophil count decreased	0 (0.0%)	0 (0.0%)	0 (0.0%)	0 (0.0%)	0 (0.0%)	1 (0.8%)	1 (0.1%)
**Respiratory, thoracic and mediastinal disorders**	**0 (0.0%)**	**2 (1.6%)**	**3 (2.4%)**	**2 (1.6%)**	**2 (1.7%)**	**5 (4.0%)**	**14 (1.9%)**
- Haemoptysis	0 (0.0%)	0 (0.0%)	0 (0.0%)	0 (0.0%)	1 (0.8%)	3 (2.4%)	4 (0.5%)
- Pneumothorax	0 (0.0%)	0 (0.0%)	0 (0.0%)	1 (0.8%)	1 (0.8%)	1 (0.8%)	3 (0.4%)
- Respiratory failure	0 (0.0%)	0 (0.0%)	1 (0.8%)	0 (0.0%)	0 (0.0%)	1 (0.8%)	2 (0.3%)
- Dyspnoea	0 (0.0%)	0 (0.0%)	1 (0.8%)	1 (0.8%)	0 (0.0%)	0 (0.0%)	2 (0.3%)
- Pulmonary haemorrhage	0 (0.0%)	1 (0.8%)	0 (0.0%)	0 (0.0%)	0 (0.0%)	0 (0.0%)	1 (0.1%)
- Pulmonary cavitation	0 (0.0%)	0 (0.0%)	0 (0.0%)	0 (0.0%)	0 (0.0%)	1 (0.8%)	1 (0.1%)
- Acute respiratory failure	0 (0.0%)	0 (0.0%)	1 (0.8%)	0 (0.0%)	0 (0.0%)	0 (0.0%)	1 (0.1%)
- Acute pulmonary oedema	0 (0.0%)	1 (0.8%)	0 (0.0%)	0 (0.0%)	0 (0.0%)	0 (0.0%)	1 (0.1%)
**Gastrointestinal disorders**	**1 (0.8%)**	**1 (0.8%)**	**1 (0.8%)**	**4 (3.2%)**	**2 (1.7%)**	**5 (4.0%)**	**14 (1.9%)**
- Vomiting	0 (0.0%)	1 (0.8%)	0 (0.0%)	3 (2.4%)	0 (0.0%)	2 (1.6%)	6 (0.8%)
- Pancreatitis	0 (0.0%)	0 (0.0%)	0 (0.0%)	0 (0.0%)	0 (0.0%)	1 (0.8%)	1 (0.1%)
- Abdominal hernia	1 (0.8%)	0 (0.0%)	0 (0.0%)	0 (0.0%)	0 (0.0%)	0 (0.0%)	1 (0.1%)
- Acute abdomen	0 (0.0%)	0 (0.0%)	1 (0.8%)	0 (0.0%)	0 (0.0%)	0 (0.0%)	1 (0.1%)
- Abdominal pain upper	0 (0.0%)	0 (0.0%)	0 (0.0%)	0 (0.0%)	1 (0.8%)	0 (0.0%)	1 (0.1%)
- Mallory-Weiss syndrome	0 (0.0%)	0 (0.0%)	0 (0.0%)	0 (0.0%)	0 (0.0%)	1 (0.8%)	1 (0.1%)
- Gastritis	0 (0.0%)	0 (0.0%)	0 (0.0%)	0 (0.0%)	1 (0.8%)	0 (0.0%)	1 (0.1%)
- Upper gastrointestinal haemorrhage	0 (0.0%)	0 (0.0%)	0 (0.0%)	0 (0.0%)	1 (0.8%)	0 (0.0%)	1 (0.1%)
- Pancreatitis acute	0 (0.0%)	0 (0.0%)	0 (0.0%)	0 (0.0%)	0 (0.0%)	1 (0.8%)	1 (0.1%)
- Nausea	0 (0.0%)	0 (0.0%)	0 (0.0%)	1 (0.8%)	0 (0.0%)	0 (0.0%)	1 (0.1%)
- Small intestinal obstruction	0 (0.0%)	0 (0.0%)	0 (0.0%)	1 (0.8%)	0 (0.0%)	0 (0.0%)	1 (0.1%)
**Injury, poisoning and procedural complications**	**0 (0.0%)**	**1 (0.8%)**	**2 (1.6%)**	**1 (0.8%)**	**2 (1.7%)**	**4 (3.2%)**	**10 (1.3%)**
- Alcohol poisoning	0 (0.0%)	1 (0.8%)	0 (0.0%)	0 (0.0%)	0 (0.0%)	2 (1.6%)	3 (0.4%)
- Thermal burn	0 (0.0%)	0 (0.0%)	1 (0.8%)	0 (0.0%)	0 (0.0%)	0 (0.0%)	1 (0.1%)
- Hip fracture	0 (0.0%)	0 (0.0%)	0 (0.0%)	0 (0.0%)	1 (0.8%)	0 (0.0%)	1 (0.1%)
- Ankle fracture	0 (0.0%)	0 (0.0%)	0 (0.0%)	0 (0.0%)	0 (0.0%)	1 (0.8%)	1 (0.1%)
- Thoracic vertebral fracture	0 (0.0%)	0 (0.0%)	0 (0.0%)	1 (0.8%)	0 (0.0%)	0 (0.0%)	1 (0.1%)
- Stab wound	0 (0.0%)	0 (0.0%)	0 (0.0%)	0 (0.0%)	0 (0.0%)	1 (0.8%)	1 (0.1%)
- Femur fracture	0 (0.0%)	0 (0.0%)	0 (0.0%)	0 (0.0%)	1 (0.8%)	0 (0.0%)	1 (0.1%)

Number (%) of participants experiencing serious adverse events (SAEs) by SOC and PT by Week 104 (Safety Population)							
Event<br>**System Organ Class (SOC)**<br>- Preferred Term (PT)	**9BLMZ**<br>**(n = 126)**	**9BCLLfxZ**<br>**(n = 122)**	**9BDLLfxZ**<br>**(n = 127)**	**9DCLLfxZ**<br>**(n = 124)**	**9DCMZ**<br>**(n = 120)**	**Control**<br>**(n = 126)**	**Total***<br>**(N = 745)**
- Abortion induced incomplete	0 (0.0%)	0 (0.0%)	1 (0.8%)	0 (0.0%)	0 (0.0%)	0 (0.0%)	1 (0.1%)
**Nervous system disorders**	**0 (0.0%)**	**2 (1.6%)**	**2 (1.6%)**	**5 (4.0%)**	**0 (0.0%)**	**1 (0.8%)**	**10 (1.3%)**
- Seizure	0 (0.0%)	1 (0.8%)	0 (0.0%)	2 (1.6%)	0 (0.0%)	0 (0.0%)	3 (0.4%)
- Transient ischaemic attack	0 (0.0%)	1 (0.8%)	0 (0.0%)	1 (0.8%)	0 (0.0%)	0 (0.0%)	2 (0.3%)
- Idiopathic intracranial hypertension	0 (0.0%)	0 (0.0%)	0 (0.0%)	0 (0.0%)	0 (0.0%)	1 (0.8%)	1 (0.1%)
- Facial paralysis	0 (0.0%)	0 (0.0%)	1 (0.8%)	0 (0.0%)	0 (0.0%)	0 (0.0%)	1 (0.1%)
- Neuropathy peripheral	0 (0.0%)	0 (0.0%)	1 (0.8%)	0 (0.0%)	0 (0.0%)	0 (0.0%)	1 (0.1%)
- Optic neuritis	0 (0.0%)	0 (0.0%)	0 (0.0%)	1 (0.8%)	0 (0.0%)	0 (0.0%)	1 (0.1%)
- Brain injury	0 (0.0%)	0 (0.0%)	0 (0.0%)	1 (0.8%)	0 (0.0%)	0 (0.0%)	1 (0.1%)
**Psychiatric disorders**	**1 (0.8%)**	**3 (2.5%)**	**1 (0.8%)**	**0 (0.0%)**	**3 (2.5%)**	**2 (1.6%)**	**10 (1.3%)**
- Suicide attempt	0 (0.0%)	1 (0.8%)	0 (0.0%)	0 (0.0%)	3 (2.5%)	1 (0.8%)	5 (0.7%)
- Brief psychotic disorder without marked stressors	1 (0.8%)	0 (0.0%)	0 (0.0%)	0 (0.0%)	0 (0.0%)	0 (0.0%)	1 (0.1%)
- Depression	0 (0.0%)	0 (0.0%)	0 (0.0%)	0 (0.0%)	0 (0.0%)	1 (0.8%)	1 (0.1%)
- Drug dependence	0 (0.0%)	1 (0.8%)	0 (0.0%)	0 (0.0%)	0 (0.0%)	0 (0.0%)	1 (0.1%)
- Psychotic disorder	0 (0.0%)	1 (0.8%)	0 (0.0%)	0 (0.0%)	0 (0.0%)	0 (0.0%)	1 (0.1%)
- Anxiety disorder	0 (0.0%)	0 (0.0%)	1 (0.8%)	0 (0.0%)	0 (0.0%)	0 (0.0%)	1 (0.1%)
**Hepatobiliary disorders**	**1 (0.8%)**	**1 (0.8%)**	**0 (0.0%)**	**1 (0.8%)**	**2 (1.7%)**	**2 (1.6%)**	**7 (0.9%)**
- Hepatotoxicity	1 (0.8%)	0 (0.0%)	0 (0.0%)	0 (0.0%)	0 (0.0%)	1 (0.8%)	2 (0.3%)
- Drug-induced liver injury	0 (0.0%)	1 (0.8%)	0 (0.0%)	1 (0.8%)	0 (0.0%)	0 (0.0%)	2 (0.3%)
- Hepatitis	0 (0.0%)	0 (0.0%)	0 (0.0%)	0 (0.0%)	0 (0.0%)	1 (0.8%)	1 (0.1%)
- Acute on chronic liver failure	0 (0.0%)	0 (0.0%)	0 (0.0%)	0 (0.0%)	1 (0.8%)	0 (0.0%)	1 (0.1%)
- Hyperbilirubinaemia	0 (0.0%)	0 (0.0%)	0 (0.0%)	0 (0.0%)	1 (0.8%)	0 (0.0%)	1 (0.1%)
**Cardiac disorders**	**1 (0.8%)**	**2 (1.6%)**	**0 (0.0%)**	**2 (1.6%)**	**0 (0.0%)**	**1 (0.8%)**	**6 (0.8%)**
- Cardiac failure congestive	1 (0.8%)	1 (0.8%)	0 (0.0%)	0 (0.0%)	0 (0.0%)	0 (0.0%)	2 (0.3%)
- Acute myocardial infarction	0 (0.0%)	1 (0.8%)	0 (0.0%)	1 (0.8%)	0 (0.0%)	0 (0.0%)	2 (0.3%)
- Cardiac failure	0 (0.0%)	1 (0.8%)	0 (0.0%)	1 (0.8%)	0 (0.0%)	0 (0.0%)	2 (0.3%)
- Cardiomyopathy alcoholic	0 (0.0%)	0 (0.0%)	0 (0.0%)	0 (0.0%)	0 (0.0%)	1 (0.8%)	1 (0.1%)
**General disorders and administration site conditions**	**2 (1.6%)**	**0 (0.0%)**	**0 (0.0%)**	**2 (1.6%)**	**2 (1.7%)**	**0 (0.0%)**	**6 (0.8%)**
- Pyrexia	1 (0.8%)	0 (0.0%)	0 (0.0%)	0 (0.0%)	0 (0.0%)	0 (0.0%)	1 (0.1%)
- Disease progression	0 (0.0%)	0 (0.0%)	0 (0.0%)	1 (0.8%)	0 (0.0%)	0 (0.0%)	1 (0.1%)
- Death	0 (0.0%)	0 (0.0%)	0 (0.0%)	1 (0.8%)	0 (0.0%)	0 (0.0%)	1 (0.1%)
- Chest pain	0 (0.0%)	0 (0.0%)	0 (0.0%)	0 (0.0%)	1 (0.8%)	0 (0.0%)	1 (0.1%)
- Sudden death	0 (0.0%)	0 (0.0%)	0 (0.0%)	0 (0.0%)	1 (0.8%)	0 (0.0%)	1 (0.1%)

**Number (%) of participants experiencing serious adverse events (SAEs) by SOC and PT by Week 104 (Safety Population)**							
Event<br>**System Organ Class (SOC)**<br>- Preferred Term (PT)	**9BLMZ**<br>**(n = 126)**	**9BCLLfxZ**<br>**(n = 122)**	**9BDLLfxZ**<br>**(n = 127)**	**9DCLLfxZ**<br>**(n = 124)**	**9DCMZ**<br>**(n = 120)**	**Control**<br>**(n = 126)**	**Total***<br>**(N = 745)**
- Generalised oedema	1 (0.8%)	0 (0.0%)	0 (0.0%)	0 (0.0%)	0 (0.0%)	0 (0.0%)	1 (0.1%)
**Metabolism and nutrition disorders**	**1 (0.8%)**	**2 (1.6%)**	**0 (0.0%)**	**0 (0.0%)**	**2 (1.7%)**	**0 (0.0%)**	**5 (0.7%)**
- Diabetes mellitus inadequate control	0 (0.0%)	0 (0.0%)	0 (0.0%)	0 (0.0%)	2 (1.7%)	0 (0.0%)	2 (0.3%)
- Hyperglycaemia	0 (0.0%)	2 (1.6%)	0 (0.0%)	0 (0.0%)	0 (0.0%)	0 (0.0%)	2 (0.3%)
- Hypoalbuminaemia	1 (0.8%)	0 (0.0%)	0 (0.0%)	0 (0.0%)	0 (0.0%)	0 (0.0%)	1 (0.1%)
**Skin and subcutaneous tissue disorders**	**0 (0.0%)**	**0 (0.0%)**	**0 (0.0%)**	**1 (0.8%)**	**3 (2.5%)**	**0 (0.0%)**	**4 (0.5%)**
- Drug reaction with eosinophilia and systemic symptoms	0 (0.0%)	0 (0.0%)	0 (0.0%)	1 (0.8%)	1 (0.8%)	0 (0.0%)	2 (0.3%)
- Dermatitis exfoliative	0 (0.0%)	0 (0.0%)	0 (0.0%)	0 (0.0%)	1 (0.8%)	0 (0.0%)	1 (0.1%)
- Swelling face	0 (0.0%)	0 (0.0%)	0 (0.0%)	0 (0.0%)	1 (0.8%)	0 (0.0%)	1 (0.1%)
**Eye disorders**	**1 (0.8%)**	**2 (1.6%)**	**0 (0.0%)**	**0 (0.0%)**	**0 (0.0%)**	**1 (0.8%)**	**4 (0.5%)**
- Optic neuropathy	1 (0.8%)	1 (0.8%)	0 (0.0%)	0 (0.0%)	0 (0.0%)	0 (0.0%)	2 (0.3%)
- Eye haemorrhage	0 (0.0%)	1 (0.8%)	0 (0.0%)	0 (0.0%)	0 (0.0%)	0 (0.0%)	1 (0.1%)
- Cataract subcapsular	0 (0.0%)	0 (0.0%)	0 (0.0%)	0 (0.0%)	0 (0.0%)	1 (0.8%)	1 (0.1%)
**Musculoskeletal and connective tissue disorders**	**0 (0.0%)**	**1 (0.8%)**	**0 (0.0%)**	**1 (0.8%)**	**2 (1.7%)**	**0 (0.0%)**	**4 (0.5%)**
- Arthralgia	0 (0.0%)	0 (0.0%)	0 (0.0%)	1 (0.8%)	1 (0.8%)	0 (0.0%)	2 (0.3%)
- Arthropathy	0 (0.0%)	1 (0.8%)	0 (0.0%)	0 (0.0%)	0 (0.0%)	0 (0.0%)	1 (0.1%)
- Osteoarthritis	0 (0.0%)	0 (0.0%)	0 (0.0%)	0 (0.0%)	1 (0.8%)	0 (0.0%)	1 (0.1%)
**Vascular disorders**	**1 (0.8%)**	**1 (0.8%)**	**0 (0.0%)**	**1 (0.8%)**	**0 (0.0%)**	**0 (0.0%)**	**3 (0.4%)**
- Deep vein thrombosis	0 (0.0%)	0 (0.0%)	0 (0.0%)	1 (0.8%)	0 (0.0%)	0 (0.0%)	1 (0.1%)
- Diabetic vascular disorder	1 (0.8%)	0 (0.0%)	0 (0.0%)	0 (0.0%)	0 (0.0%)	0 (0.0%)	1 (0.1%)
- Hypertension	0 (0.0%)	1 (0.8%)	0 (0.0%)	0 (0.0%)	0 (0.0%)	0 (0.0%)	1 (0.1%)
**Renal and urinary disorders**	**1 (0.8%)**	**0 (0.0%)**	**1 (0.8%)**	**0 (0.0%)**	**0 (0.0%)**	**0 (0.0%)**	**2 (0.3%)**
- Acute kidney injury	1 (0.8%)	0 (0.0%)	0 (0.0%)	0 (0.0%)	0 (0.0%)	0 (0.0%)	1 (0.1%)
- Proteinuria	0 (0.0%)	0 (0.0%)	1 (0.8%)	0 (0.0%)	0 (0.0%)	0 (0.0%)	1 (0.1%)
**Neoplasms benign, malignant and unspecified (incl cysts and polyps)**	**0 (0.0%)**	**0 (0.0%)**	**0 (0.0%)**	**0 (0.0%)**	**1 (0.8%)**	**1 (0.8%)**	**2 (0.3%)**
- Pancreatic carcinoma	0 (0.0%)	0 (0.0%)	0 (0.0%)	0 (0.0%)	1 (0.8%)	0 (0.0%)	1 (0.1%)
- Lung neoplasm malignant	0 (0.0%)	0 (0.0%)	0 (0.0%)	0 (0.0%)	0 (0.0%)	1 (0.8%)	1 (0.1%)
**Immune system disorders**	**0 (0.0%)**	**0 (0.0%)**	**2 (1.6%)**	**0 (0.0%)**	**0 (0.0%)**	**0 (0.0%)**	**2 (0.3%)**
- Hypersensitivity	0 (0.0%)	0 (0.0%)	2 (1.6%)	0 (0.0%)	0 (0.0%)	0 (0.0%)	2 (0.3%)
**Product issues**	**1 (0.8%)**	**0 (0.0%)**	**0 (0.0%)**	**0 (0.0%)**	**0 (0.0%)**	**0 (0.0%)**	**1 (0.1%)**
- Device dislocation	1 (0.8%)	0 (0.0%)	0 (0.0%)	0 (0.0%)	0 (0.0%)	0 (0.0%)	1 (0.1%)
**Pregnancy, puerperium and perinatal conditions**	**0 (0.0%)**	**0 (0.0%)**	**1 (0.8%)**	**0 (0.0%)**	**0 (0.0%)**	**0 (0.0%)**	**1 (0.1%)**

Number (%) of participants experiencing serious adverse events (SAEs) by SOC and PT by Week 104 (Safety Population)							
Event	**9BLMZ**	**9BCLLfxZ**	**9BDLLfxZ**	**9DCLLfxZ**	**9DCMZ**	**Control**	**Total***
**System Organ Class (SOC)**<br>- Preferred Term (PT)	**(n = 126)**	**(n = 122)**	**(n = 127)**	**(n = 124)**	**(n = 120)**	**(n = 126)**	**(N = 745)**
- Abortion threatened	0 (0.0%)	0 (0.0%)	1 (0.8%)	0 (0.0%)	0 (0.0%)	0 (0.0%)	1 (0.1%)

**Total by preferred term (PT) represents the sum of participants who experienced at least one event corresponding to that PT. If they experienced more than one event of the same PT, it is counted only once. If a participant experienced more than one unique PT within a system organ class (SOC), each unique PT counts toward the PT total, but only one event counts toward the SOC.*

Table S35. Number (%) of participants with adverse events of special interest (AESI) of grade 3 or higher and time to onset by Week 73 (Safety population)

**Adverse events (AEs)**	**9BLMZ (n = 126)**	**9BCLLfxZ (n = 122)**	**9BDLLfxZ (n = 127)**	**9DCLLfxZ (n = 124)**	**9DCMZ (n = 120)**	**Control (n = 126)**	**Total (N = 745)**
- AESI: ≥ grade 3 hematologic toxicity	11 (8.7%)	9 (7.4%)	10 (7.9%)	13 (10.5%)	9 (7.5%)	13 (10.3%)	65 (8.7%)
Risk difference from control (95% CI)	-1.6 (-8.8;5.7)	-2.9 (-10.0;4.1)	-2.4 (-9.5;4.6)	0.2 (-7.4;7.7)	-2.8 (-9.9;4.3)	-	
Days to event, median [IQR]	*29 [26-109]*	*80 [56-171]*	*67.5 [28-82]*	*83 [61.0-98.0]*	*145 [88-205]*	*112 [28-269]*	*83.0 [28-171.0]*
- AESI: ≥ grade 3 peripheral neuropathy	4 (3.2%)	5 (4.1%)	9 (7.1%)	3 (2.4%)	3 (2.5%)	6 (4.8%)	30 (4.0%)
Risk difference from control (95% CI)	-1.6 (-6.4;3.2)	-0.7 (-5.8;4.5)	2.3 (-3.5;8.1)	-2.3 (-6.9;2.3)	-2.3 (-6.9;2.4)	-	
Days to event, median [IQR]	*162.5 [144-220]*	*83 [50-137]*	*166 [125-220]*	*119 [112-329]*	*57 [34-139]*	*218.5 [178-248]*	*162 [112-220]*
- AESI: ≥ grade 3 hepatotoxicity	23 (18.3%)	17 (13.9%)	8 (6.3%)	18 (14.5%)	12 (10.0%)	9 (7.1%)	87 (11.7%)
Risk difference from control (95% CI)	11.1 (3.0;19.2)	6.8 (-0.8;14.4)	-0.8 (-7.0;5.3)	7.4 (-0.3;15.0)	2.9 (-4.1;9.9)	-	
Days to event, median [IQR]	*56 [41-154]*	*82 [55-139]*	*41 [25.5-56]*	*81.5 [40-200]*	*59.5 [26-167]*	*84 [40-269]*	*58 [40-166]*
- AESI: ≥ grade 3 optic neuropathy	0 (0.0%)	1 (0.8%)	0 (0.0%)	1 (0.8%)	0 (0.0%)	2 (1.6%)	4 (0.5%)
Days to event, median [IQR]	-	*65 [65-65]*	-	*106 [106-106]*	-	*106.5 [57-156]*	*85.5 [61-131]*
- AESI: ≥ grade 3 QT prolongation	0 (0.0%)	4 (3.3%)	0 (0.0%)	0 (0.0%)	5 (4.2%)	0 (0.0%)	9 (1.2%)
Days to event, median [IQR]	-	*169 [119-244.5]*	-	-	*222.0 [145-223]*	-	*177 [145-223]*

Confidence intervals widths have not been adjusted for multiplicity and the intervals should not be used in place of hypothesis testing. They are only presented for precision purposes

Table S36. Adverse events of special interest (AESI) of grade 3 or higher and time to onset by Week 104 (Safety population)

**Adverse events (AEs)**	**9BLMZ (n = 126)**	**9BCLLfxZ (n = 122)**	**9BDLLfxZ (n = 127)**	**9DCLLfxZ (n = 124)**	**9DCMZ (n = 120)**	**Control (n = 126)**	**Total (N = 745)**
- AESI: ≥ grade 3 hematologic toxicity	11 (8.7%)	10 (8.2%)	10 (7.9%)	13 (10.5%)	9 (7.5%)	14 (11.1%)	67 (9.0%)
Days to event, median [IQR]	*29 [26-109]*	*123 [56-194]*	*67.5 [28-82]*	*83 [61.0-98.0]*	*145 [88-205]*	*148 [28-286]*	*83 [28-193]*
- AESI: ≥ grade 3 peripheral neuropathy	4 (3.2%)	5 (4.1%)	9 (7.1%)	3 (2.4%)	3 (2.5%)	6 (4.8%)	30 (4.0%)
Days to event, median [IQR]	*162.5 [144-220]*	*83 [50-137]*	*166 [125-220]*	*119 [112-329]*	*57 [34-139]*	*218.5 [178-248]*	*162 [112-220]*
- AESI: ≥ grade 3 hepatotoxicity	23 (18.3%)	19 (15.6%)	11 (8.7%)	18 (14.5%)	12 (10.0%)	9 (7.1%)	92 (12.4%)
Days to event, median [IQR]	*56 [41-154]*	*110 [55-221]*	*56 [26-509]*	*81.5 [40-200]*	*59.5 [26-167]*	*84 [40-269]*	*71 [40.5-185]*
- AESI: ≥ grade 3 optic neuropathy	0 (0.0%)	1 (0.8%)	0 (0.0%)	1 (0.8%)	0 (0.0%)	2 (1.6%)	4 (0.5%)
Days to event, median [IQR]	-	*65 [65-65]*	-	*106 [106-106]*	-	*106.5 [57-156]*	*85.5 [61-131]*
- AESI: ≥ grade 3 QT prolongation	0 (0.0%)	4 (3.3%)	0 (0.0%)	0 (0.0%)	5 (4.2%)	0 (0.0%)	9 (1.2%)
Days to event, median [IQR]	-	*169 [119-244.5]*	-	-	*222.0 [145-223]*	-	*177 [145-223]*

Table S37. Frequency of endTB trial Participants Experiencing Adverse Events Leading to (and Time to) Permanent Discontinuation of at Least One Study Drug by Treatment Group by Week 73 (Safety Population)

**Adverse events (AEs)**	**9BLMZ (n = 126)**	**9BCLLfxZ (n = 122)**	**9BDLLfxZ (n = 127)**	**9DCLLfxZ (n = 124)**	**9DCMZ (n = 120)**	**Control (n = 126)**	**Total (N = 745)**
Participants with at least one AE leading to permanent discontinuation ≥1 drug	26 (20.6%)	32 (26.2%)	35 (27.6%)	29 (23.4%)	19 (15.8%)	51 (40.5%)	192 (25.8%)
Days to drug discontinuation, median [IQR]	101 [82-225]	134 [76-217]	127 [61-197]	134 [77-178]	103 [41-217]	160.0 [60-315]	133.5 [65.5-217.5]
- **Pyrazinamide**	**22 (17.5%)**	**23 (18.9%)**	**21 (16.5%)**	**16 (12.9%)**	**15 (12.5%)**	**11 (8.7%)**	**108 (14.5%)**
Days to drug discontinuation, median [IQR]	101 [82-226]	112 [72-199]	103 [61-181]	85 [47-172.5]	63 [41-164]	58 [38-145]	92 [57.5-184.5]
- **Linezolid**	**7 (5.6%)**	**10 (8.2%)**	**15 (11.8%)**	**15 (12.1%)**	**-**	**19 (15.1%)**	**66 (8.9%)**
Days to drug discontinuation, median [IQR]	131 [73-184]	208.5 [112-239]	145 [69-201]	134 [78-195]	-	195 [141-319]	164 [112-225]
- **Clofazimine**	**-**	**5 (4.1%)**	**-**	**5 (4.0%)**	**5 (4.2%)**	**7 (5.6%)**	**22 (3.0%)**
Days to drug discontinuation, median [IQR]	-	192 [163-259]	-	168 [49-273]	103 [50-242]	366.0 [284-440]	250.5 [103-315]
- **Levofloxacin**	**-**	**4 (3.3%)**	**1 (0.8%)**	**5 (4.0%)**	**1 (0.8%)**	**12 (9.5%)**	**23 (3.1%)**
Days to drug discontinuation, median [IQR]	-	232.5 [155-287.5]	69 [69-69]	178 [49-273]	83 [83-83]	245 [164.5-439]	213 [83-323]
- **Bedaquiline**	**4 (3.2%)**	**3 (2.5%)**	**1 (0.8%)**	**-**	**-**	**6 (4.8%)**	**14 (1.9%)**
Days to drug discontinuation, median [IQR]	123.5 [72-190.5]	210 [97-323]	69 [69-69]	-	-	427 [169-440]	189.5 [91-414]
- **Moxifloxacin**	**5 (4.0%)**	**-**	**-**	**-**	**5 (4.2%)**	**1 (0.8%)**	**11 (1.5%)**
Days to drug discontinuation, median [IQR]	112 [90-156]	-	-	-	103 [50-261]	23 [23-23]	103 [50-225]
- **Delamanid**	**-**	**-**	**1 (0.8%)**	**4 (3.2%)**	**3 (2.5%)**	**0 (0.0%)**	**8 (1.1%)**
Days to drug discontinuation, median [IQR]	-	-	69 [69-69]	161 [40-290]	50 [41-103]	-	59.5 [45.0-188]

Table S38. Participant pregnancies, exposure information, and treatment decision during the endTB Trial

N	Arm	Age Range	Exposure information & timing	Participant treatment/study decision	Time-to-onset days)
1	9BDLLfxZ	20-29	Maternal exposure during pregnancy from 1st trimester	Early discontinued	103
2	9BLMZ	20-29	Maternal exposure during pregnancy from 1st trimester	Early discontinued	224
3	9BDLLfxZ	20-29	Maternal exposure during pregnancy from 1st trimester	Maintained in the study	127
4	9BDLLfxZ	20-29	Maternal exposure during pregnancy from 1st trimester	Maintained in the study	77
5	9BDLLfxZ	20-29	Maternal exposure during pregnancy from 1st trimester	Maintained in the study	63
6	9BDLLfxZ	30-39	Maternal exposure before pregnancy	Maintained in the study	497
7	Control	30-39	Maternal exposure during pregnancy from 1st trimester	Maintained in the study	66
8	9BLMZ	30-39	Maternal exposure before pregnancy	Study completed	~665
9	9DCMZ	30-39	Maternal exposure before pregnancy	Maintained in the study	566
10	9BCLlfxZ	20-29	Maternal exposure during pregnancy from 1st trimester	Maintained in the study	280
11	Control	30-39	Maternal exposure during pregnancy from 1st trimester	Maintained in the study	129
12	9DCLLfxZ	30-39	Maternal exposure before pregnancy	Maintained in the study	701
13	9DCLLfxZ	20-29	Maternal exposure during pregnancy from 1st trimester	Maintained in the study	150
14	9DCLLfxZ	20-29	Maternal exposure before pregnancy	Maintained in the study	607
15	9DCMZ	20-29	Maternal exposure before pregnancy	Maintained in the study	418
16	Control	20-29	Maternal exposure before pregnancy	Maintained in the study	350
17	Control	20-29	Maternal exposure before pregnancy	Maintained in the study	473
18	Control	40-49	Maternal exposure during pregnancy from 1st trimester	Maintained in the study	422
19	9DCMZ	20-29	Maternal exposure before pregnancy	Maintained in the study	569
20	9BDLLfxZ	20-29	Maternal exposure before pregnancy	Maintained in the study	487
21	9BDLLfxZ	20-29	Maternal exposure before pregnancy	Maintained in the study	582
22	Control	20-29	Paternal exposure before pregnancy	Maintained in the study	374
23	9DCLLfxZ	Unknown	Paternal exposure before pregnancy	Maintained in the study	Unknown
24	9BDLLfxZ	Unknown	Paternal exposure before pregnancy	Maintained in the study	Unknown
25	9DCMZ	Unknown	Paternal exposure before pregnancy	Maintained in the study	652
26	9DCLLfxZ	15-19	Paternal exposure before pregnancy	Maintained in the study	388
27	9DCLLfxZ	15-19	Paternal exposure before pregnancy	Maintained in the study	146
28	9BLMZ	Unknown	Paternal exposure before pregnancy	Maintained in the study	256
29	Control	Unknown	Paternal exposure before pregnancy	Maintained in the study	78
30	9BDLLfxZ	Unknown	Paternal exposure before pregnancy	Maintained in the study	11
31	Control	Unknown	Paternal exposure before pregnancy	Maintained in the study	444
32	9DCLLfxZ	20-29	Paternal exposure before pregnancy	Maintained in the study	403
33	9BLMZ	Unknown	Paternal exposure before pregnancy	Maintained in the study	582

4 References

1. WHO. Global Tuberculosis Programme [Available from: *https://www.who.int/teams/global-tuberculosis-programme/data*.
2. WHO. Global tuberculosis report 2023. Geneva2023.
3. WHO. Web Annexes. In: WHO consolidated guidelines on tuberculosis. Module 4: treatment - drug-resistant tuberculosis treatment, 2022 update. Geneva: World Health Organization; 2022. p. 408.
4. Wason JM, Trippa L. A comparison of Bayesian adaptive randomization and multi-stage designs for multi-arm clinical trials. Stat Med. 2014;33(13):2206-21.
5. WHO. WHO treatment guidelines for drug-resistant tuberculosis 2016 update. Geneva: World Health Organization; 2016.
6. Lanoix JP, Chaisson RE, Nuermberger EL. Shortening Tuberculosis Treatment With Fluoroquinolones: Lost in Translation? Clin Infect Dis. 2016;62(4):484-90.
7. Tasneen R, Li SY, Peloquin CA, Taylor D, Williams KN, Andries K, et al. Sterilizing activity of novel TMC207- and PA-824-containing regimens in a murine model of tuberculosis. Antimicrob Agents Chemother. 2011;55(12):5485-92.
8. Upton AM, Cho S, Yang TJ, Kim Y, Wang Y, Lu Y, et al. In vitro and in vivo activities of the nitroimidazole TBA-354 against Mycobacterium tuberculosis. Antimicrob Agents Chemother. 2015;59(1):136-44.
9. Tasneen R, Williams K, Amoabeng O, Minkowski A, Mdluli KE, Upton AM, et al. Contribution of the nitroimidazoles PA-824 and TBA-354 to the activity of novel regimens in murine models of tuberculosis. Antimicrob Agents Chemother. 2015;59(1):129-35.
10. Dooley KE, Rosenkranz SL, Conradie F, Moran L, Hafner R, von Groote-Bidlingmaier F, et al. QT effects of bedaquiline, delamanid, or both in patients with rifampicin-resistant tuberculosis: a phase 2, open-label, randomised, controlled trial. Lancet Infect Dis. 2021;21(7):975-83.
11. Nunn AJ, Phillips PP, Mitchison DA. Timing of relapse in short-course chemotherapy trials for tuberculosis. Int J Tuberc Lung Dis. 2010;14(2):241-2.
12. Diacon AH, Pym A, Grobusch MP, de los Rios JM, Gotuzzo E, Vasilyeva I, et al. Multidrug-resistant tuberculosis and culture conversion with bedaquiline. N Engl J Med. 2014;371(8):723-32.
13. von Groote-Bidlingmaier F, Patientia R, Sanchez E, Balanag V, Jr., Ticona E, Segura P, et al. Efficacy and safety of delamanid in combination with an optimised background regimen for treatment of multidrug-resistant tuberculosis: a multicentre, randomised, double-blind, placebo-controlled, parallel group phase 3 trial. Lancet Respir Med. 2019;7(3):249-59.
14. Nyang'wa BT, Berry C, Kazounis E, Motta I, Parpieva N, Tigay Z, et al. A 24-Week, All-Oral Regimen for Rifampin-Resistant Tuberculosis. N Engl J Med. 2022;387(25):2331-43.
15. Tweed CD, Wills GH, Crook AM, Amukoye E, Balanag V, Ban AYL, et al. A partially randomised trial of pretomanid, moxifloxacin and pyrazinamide for pulmonary TB. Int J Tuberc Lung Dis. 2021;25(4):305-14.
16. Paton NI, Cousins C, Suresh C, Burhan E, Chew KL, Dalay VB, et al. Treatment Strategy for Rifampin-Susceptible Tuberculosis. N Engl J Med. 2023;388(10):873-87.
17. WHO. Rapid communication: key changes to treatment of multidrug- and rifampicin-resistant tuberculosis (MDR/RR-TB). August 2018.
18. WHO. Rapid communication: key changes to treatment of drug-resistant tuberculosis. Geneva: World Health Organization; December 2019.
19. WHO. WHO consolidated guidelines on tuberculosis. Module 4: treatment - drug-resistant tuberculosis treatment, 2022 update. Geneva: World Health Organization; 2022.
